# Clinicians’ perspectives on incidentally discovered silent brain infarcts – A qualitative study

**DOI:** 10.1371/journal.pone.0194971

**Published:** 2018-03-29

**Authors:** Lester Y. Leung, Paul K. J. Han, Christine Lundquist, Gene Weinstein, David E. Thaler, David Kent

**Affiliations:** 1 Division of Stroke and Cerebrovascular Diseases, Department of Neurology, Tufts Medical Center, Boston, Massachusetts, United States of America; 2 Center for Outcomes Research & Evaluation, Maine Medical Center Research Institute, Portland, Maine, United States of America; 3 Predictive Analytics and Comparative Effectiveness Center, Institute for Clinical Research and Health Policy Studies, Tufts Medical Center, Boston, Massachusetts, United States of America; 4 Department of Radiology, Massachusetts General Hospital, Boston, Massachusetts, United States of America; Cleveland Clinic, UNITED STATES

## Abstract

**Background:**

While silent brain infarcts (SBIs) in screened cohorts are associated with risk of symptomatic stroke and dementia, the clinical significance of incidentally discovered SBIs (id-SBIs) is unknown. Detection may offer an opportunity to initiate prevention measures, but uncertainties about id-SBIs may impede clinicians from addressing them and complicate further study of this condition.

**Methods and results:**

This study used semi-structured interviews of practicing clinicians. Interviews were audio recorded, transcribed, and analyzed using a grounded theory approach. A constant comparative method was used to organize emergent themes and examine new themes. Purposeful sampling was employed to achieve participant diversity. Fifteen clinicians were interviewed. Emergent themes centered on uncertainty about id-SBIs, clinical decision making in response to uncertainty, and evidence needed to resolve uncertainty. All clinicians reported uncertainty about id-SBIs: diagnostic, prognostic, or therapeutic. Differential responses to uncertainties resulted in practice variation within and between specialties. Diagnostic and prognostic uncertainty discouraged disclosure of imaging findings to patients. Vascular neurologists viewed the prognostic significance of id-SBIs as similar to symptomatic stroke. Therapeutic uncertainty was common, but most participants endorsed using stroke secondary prevention strategies. Regarding future research, all internists indicated they would consider changing practices in response to observational studies, whereas half of the neurologists expressed reluctance to modify practices based on non-randomized data. Several expressed concerns about clinical trial feasibility and lack of equipoise.

**Conclusions:**

id-SBIs are a focus of uncertainty for clinicians, leading to practice variation. Future studies must address diagnostic and prognostic uncertainty to facilitate implementation of prevention strategies.

## Introduction

Silent brain infarcts (SBIs) are common and have important consequences, but optimal management strategies have not been established. SBIs occur without overt symptoms and remain undetected until discovered on neuroimaging. In screened cohorts, SBIs are associated with a two-to-three fold increased risk of symptomatic brain infarction and dementia, independent of vascular risk factors [1–4]. SBIs are more common than ischemic stroke: for U.S. adults over age 50, the estimated prevalence of SBIs is 20%, and the annual incidence may be 11 times greater than for symptomatic infarcts [5]. However, without standard screening, SBIs are only detected if discovered incidentally on neuroimaging performed for other indications: it is unknown if these incidentally discovered SBIs (id-SBIs) resemble those studied in screened cohorts with respect to frequency and outcomes.

To guide research on SBIs, the American Heart Association/American Stroke Association (AHA/ASA) refined definitions for silent cerebrovascular diseases and released a scientific statement highlighting potential harms following SBIs, the lack of randomized clinical trial (RCT) data, the probable value of stroke primary prevention strategies following SBIs, and the probable inappropriateness of population screening [6–7]. However, as the data informing this statement were drawn from studies on SBIs in neuroimaging screened cohorts, it is not clear how clinicians should approach id-SBIs, the only SBIs currently relevant to patient care. There are no studies describing clinical practices for id-SBIs or identifying factors that influence clinical decision making, especially barriers to addressing these SBIs. Furthermore, it is unknown how readily clinicians will incorporate evidence from future treatment studies (such as RCTs and observational comparative effectiveness research (CER)) into their clinical practices.

To assess potential barriers for clinicians to address this condition or study the effectiveness of prevention measures, we assessed perspectives of internists and neurologists encountering patients with id-SBIs to 1) identify areas of uncertainty that need to be addressed by future studies and guidelines, and 2) inform the design of future studies to optimize dissemination and acceptance of study findings.

## Methods

### Study design, participants, and data collection

This study was approved by the Tufts Medical Center IRB. This study used individual, semi-structured qualitative interviews of internists (IN), general neurologists (GN), and vascular neurologists (VN), the clinicians thought most likely to encounter patients with SBIs and alter clinical practices. Participants were recruited through direct contact from greater Boston area practices. To facilitate recruitment, physicians at practices or centers other than those of the investigators were approached first if they were potentially familiar with the investigators’ names through shared patients or academic affiliations (e.g. conferences, training programs); physicians at the investigators’ centers were selected partly at random in conjunction with purposeful selection to identify clinicians varying by sex, specialty, practice setting, and experience. Clinician specialty was anticipated to be a major factor influencing responses to interview questions, so recruitment was divided in three phases (five interviews each) with each specialty represented in each recruitment phase. Participants were required to have active clinical practices, exposure to patients with SBIs, non-involvement in SBI research, and no prior communications with the investigators about SBIs. Participants were asked to estimate the average number of patients with id-SBIs they encounter annually. Interviews were conducted by one of the investigators (LYL, a male attending vascular neurologist with clinical equipoise regarding SBIs and no prior publications on SBIs at the time of the study) and lasted 30–60 minutes (duration dependent on the length of the participants’ responses and audio quality). These were audio-recorded with participant consent and transcribed verbatim by a professional transcription service (S1 File). This study did not employ field notes, participant data correction, or participant checking.

### Interview content

The interview guide was developed by the multidisciplinary team of investigators including internists, vascular neurologists, a radiologist, and a qualitative methodologist: an initial draft was produced by LYL and subsequently reviewed and edited by the other investigators to maximize understandability and minimize leading questions. The interview guide included open-ended questions and clarifying probes to explore clinicians’ perceptions of the nature of SBIs, potential harms, current practices, and approaches to new evidence (S2 File). Participants were required to respond to all “exploratory” open-ended questions and were asked as many “focused” clarifying questions as possible within the time frame of the interviews, understanding that some participants may have limited time to participate due to clinical responsibilities. The guide was modified between phases to further explore unanticipated themes and themes related to clinical decision making.

### Data analysis

Line-by-line, software-assisted coding of anonymised transcripts was performed by two investigators (LYL, CL) using NVivo (V.11; QSR International, Melbourne, Australia). An initial codebook was developed by three investigators (LYL, PH, CL) through independent review and team-based reconciliation of the first two coded transcripts, using a “grounded theory” approach to categorize thematic content in a hierarchical, logically coherent conceptual schema [8–9]. The reconciled codebook was used by two investigators (LYL, CL) to code the remaining transcripts using a “constant comparative” method [10]. In the absence of field notes, both investigators had access to the audio recordings and could review the audio alongside the transcribed interviews to clarify the context of participant statements. The three investigators met at the end of each phase to discuss coding and recruitment decisions, resolve disagreements, and revise the interview guide and codebook. This process continued until thematic saturation was achieved (i.e. no major new themes or first order codes emerged in the final interviews of each clinician specialty).

## Results

Interviews were conducted with 15 participants: seven internists, four general neurologists, and four vascular neurologists. No clinicians declined to be interviewed, and all participants responded to all interview questions. Participant characteristics are described in Table 1.

**Table 1:** Characteristics of clinicians.

Characteristic	Subcategory	n or median (IQR)
Sex	Men	9
	Women	6
Specialty	Internal medicine	7
	General neurology	4
	Vascular neurology	4
Practice Setting	Inpatient	1
	Outpatient	0
	Both	14
Institution	Academic	12
	Community	3
Years of experience	< 5 years	2
	5–10 years	6
	> 10 years	7
Estimated encounters with patients with id-SBIs (annual)	Internal medicine	10 (5–15)
	General neurology	22.5 (15–35)
	Vascular neurology	20 (13.75–27.5)

All specialties were represented in academic and community practices. Emergent themes fell into three main categories: 1) uncertainty about SBIs, 2) clinical decision making in response to uncertainty, and 3) evidence needed to resolve uncertainty. Regarding scenarios in which clinicians encountered SBIs (Table 2), the most frequent neuroimaging indications were symptoms not specific to stroke.

**Table 2:** Scenarios leading to incidental discovery of SBIs.

Asymptomatic, abnormal neurologic examination finding
Post-operative assessment of new neurologic symptoms (with detection of unrelated SBIs)
Symptomatic stroke or transient ischemic attack (with detection of unrelated SBIs)
Symptoms not specific to stroke
Altered mental status (confusion, lethargy)
Cognitive decline
Dizziness
Gait difficulty
Generalized weakness
Headaches
Lightheadedness
Memory loss
Seizures
Syncope
Trauma

### Uncertainty about SBIs

*Uncertainty about SBIs* focused on diagnostic, prognostic, and therapeutic uncertainties and their sources (Table 3).

**Table 3:** Uncertainty about SBIs–types and sources.

Categories	Subcategories	Subcategories	Representative quotations
(Level 1)	(Level 2)	(Level 3)	
Types of Uncertainty	Diagnostic	“Silent” nature of SBIs	“Is this a different subset of stroke patients in terms of etiologies?” (VN2)
		Relationship to leukoaraiosis	“I think the size and shape and location probably suggests there’s a difference between them (SBI). But again, I wouldn’t profess to have much certainty there.” (VN4)
		Causal uncertainty	
	Prognostic	Risk of future stroke	“I think it’s really hard to implicate silent strokes for any individual patient as the cause of the problem.” (IN1)
		Risk of direct harm	
	Therapeutic	Approach to management	“The truth is I don’t have an algorithm yet.” (GN1)
Sources of Uncertainty	Limited awareness and dissemination of available evidence	Prevalence	"I have no idea how common they are." (IN2)
		Outcomes	“I think it is not very well recognized that these patients are at risk of harm… That there is an urgency to treat… That they need to be treated like any other stroke patients in terms of secondary prophylaxis.” (VN2)
	
	Lack of treatment studies and guidelines	Benefit of specialist	“When would a referral be beneficial? What would a neurologist add?” (IN1)
		Benefit of treatment	“I’d like to know whether others are treating them the same way I am.” (GN2)
		Consensus	
		Guidelines	
		Testing	

#### Uncertainty about SBIs: Diagnostic

**The “silent” nature of SBIs.** Most participants expressed uncertainty about diagnosing SBIs.

“I think the biggest question for me would be what exactly you consider a silent stroke.”–general neurologist #2

In the absence of symptoms, participants attempted to define SBIs based on neuroimaging interpretation and patient characteristics (i.e. vascular risk factors) that might increase the likelihood of the finding representing infarction rather than non-vascular pathologies. A few questioned whether SBIs were always “silent” or if they sometimes had subtle, unrecognized manifestations, particularly in elderly or cognitively impaired individuals—minimizing any purported distinction between SBIs and symptomatic strokes. Neurologists defined SBIs with more certainty, citing small size and location in subcortical or “non-eloquent” parts of the brain as the primary causes of clinical silence. Terminology used to describe SBIs was variable: “silent stroke” and “asymptomatic stroke” were the most commonly used and were considered the most useful in communication with patients and other clinicians.

**Relationship to leukoaraiosis.** Several participants identified knowledge gaps in differentiating between SBIs and leukoaraiosis.

“Should I assume all white matter disease is a silent stroke outside of people who have multiple sclerosis or something like that?”–internist #5

Several suggested that both are on a spectrum where SBIs are more clinically significant and leukoaraiosis is less significant. Most felt less compelled to respond to leukoaraiosis as aggressively as SBIs, but they cited a lack of published studies guiding this practice.

**Causal uncertainty about SBIs.** Most participants hypothesized that SBIs have similar mechanisms of infarction as symptomatic strokes: a majority cited thrombotic mechanisms, whereas a minority (primarily vascular neurologists) also cited embolic mechanisms. Several participants suggested that the proportions of mechanisms might differ between SBIs and symptomatic stroke. Several participants across all specialties suggested that subtyping SBIs by presumed mechanism was useful.

#### Uncertainty about SBIs: Prognostic

**Risk of future stroke.** Even with accompanying diagnostic uncertainty, several participants expressed very strong beliefs regarding the prognostic equivalence of SBIs and symptomatic stroke. For example, two internists, three general neurologists, and three vascular neurologists expressed similar notions that:

“A stroke is a stroke.”–vascular neurologist #2

“I would say, once you’ve had one, regardless of your clinical manifestations of it, you’re a stroke victim.”–internist #3

Most participants believed that the presence of SBIs indicated an increased risk for symptomatic stroke, regardless of the presence or absence of symptoms or neurologic deficits related to the present infarct. However, one participant expressed uncertainty regarding the frequency or severity of symptomatic stroke following SBIs.

**Risk of direct harm from a “silent” condition.** In contrast to beliefs about stroke risk, internists and neurologists had different perspectives regarding the potential for direct harm from SBIs. Internists expressed more uncertainty about specific health consequences beyond stroke risk, whereas neurologists more readily suggested cognitive decline as an important direct sequelae.

#### Uncertainty about SBIs: Therapeutic

**Uncertainty in management.** While neurologists expressed greater certainty in defining SBIs, both internists and neurologists were uncertain about optimal strategies for managing patients with SBIs. Most described “probable” steps they would take on a “case by case” basis, and a few clarified that they would be less aggressive with SBIs than symptomatic strokes.

“Would I put someone on a Holter and look for afib for a silent stroke and then commit someone to a lifetime of warfarin? Those are things that I would do as part of a stroke workup. Would I do that for a silent stroke? I don’t know that I would.”–internist #2

Notably, a few expressed strong opinions that there are different types of SBIs that might warrant different tests and treatments.

“Treating everyone as if its thrombotic is probably inappropriate if the proportion (thrombotic versus embolic) is different for silent versus non-silent.”–internist #3

#### Uncertainty about SBIs: Sources of uncertainty

**Limited awareness and dissemination of available evidence.** Several participants expressed a lack of awareness of any published studies on the frequency, risk factors, or outcomes of SBIs. In particular, internists and neurologists described different impressions regarding frequency: internists assumed SBIs were uncommon, and neurologists assumed they occur frequently.

**Lack of treatment studies and guidelines.** Participants described that a lack of available evidence or guidelines fostered uncertainty in managing patients with SBIs. Several participants cited concerns about undertreatment.

“I’ve noticed that a lot of patients with these findings are not aware of them, and they are not being informed by previous doctors, so I’m not sure whether we are undertreating these silent stroke patients.”–internist #6

### Managing uncertainty

*Managing uncertainty* focused on clinical practice variation in response to different types of uncertainty (Table 4).

**Table 4:** Managing uncertainty.

Categories	Subcategories	Representative quotations
(Level 1)	(Level 2)	
Managing diagnostic uncertainty	Emphasizing the “incidental” nature of SBIs	“I equate them to silent MI (myocardial infarction): still an MI.” (IN3)
	Influence of radiologists’ language	“I might initially say they had an incidental stroke, but then eventually that becomes a different assessment… We have to work up… It goes from ‘incidental’ to all of sudden me, ‘clinically’ doing something about it.” (GN2)
Managing prognostic uncertainty	Obligation to take action	“In situations where they come up in the hospital, we haven’t usually (addressed SBI). In the inpatient setting, we’re dealing with the presenting problem. If we don’t think it’s related, I haven’t thought too much about it.” (IN2)
	Disclosing SBIs to patients	
Managing therapeutic uncertainty	Individualizing care	"They have to be aggressively managed, as you'd manage any other stroke patient." (VN2)
	Etiologic testing	"I'll use the finding of the silent stroke as an impetus to motivate them to stop smoking." (GN3)
	Lifestyle modification	"If it is a silent infarction that I see on a CT scan, I will probably do a full work up, just like how I treat a symptomatic stroke." (IN6)
	Medication management	
	Specialty referral	

#### Managing uncertainty: Diagnostic

**Emphasizing the “incidental” nature of SBIs.** Despite often viewing SBIs as equivalent in significance to symptomatic strokes, a few participants described that their approach to SBIs was shaped by the “incidental” nature of their discovery. Two internists expressed similar beliefs that incidental findings in general should be ignored unless there is a strong guideline recommendation to take action.

“In the case of silent stroke, more often than not I do nothing… In terms of other incidental findings, unless it’s a cancer risk, I tend to ignore them… I try to minimize the incidental findings as much as possible.”–internist #1

Several acknowledged that incidental findings could be clinically important (e.g. occult malignancy) or could represent opportunities to prevent related diseases.

In some cases, the incidental nature did not generate the same sense of urgency as symptomatic stroke.

“For incidental findings, I will manage it in a non-urgent way. Instead of getting a workup within a week, I feel like I can take time.”–internist #6

One general neurologist described no longer perceiving SBIs as “incidental” once they were interpreted as warranting a response, emphasizing that the term connotes a lack of need for action.

**Influence of radiologists’ language.** All neurologists reviewed neuroimaging directly and were not substantially influenced by radiologist language, whereas all internists only reviewed imaging reports and were dependent on the radiologist's certainty in determination of infarction. Internists cited that uncertainty or lack of specificity in the radiologists’ terminology would influence their actions, making them less likely to respond to possible SBIs.

"If they say infarction (I would respond). If they say white matter disease or chronic microangiopathy … (I might not)."–internist #6

#### Managing uncertainty: Prognostic

**Obligation to take action.** Participants had different views regarding their obligation to respond to SBIs discovered in routine care. Possibly due to high prognostic certainty and specialty focus, vascular neurologists uniformly felt obligated to respond aggressively to SBIs. Internists and general neurologists described being more conservative in their approaches, including some who felt no obligation to take action.

**Disclosing SBIs to patients.** In this sample, most participants described usually or always reporting SBIs to patients. Two internists with high prognostic uncertainty avoided disclosure, citing difficulty in explaining SBIs to their patients and fearing negative patient responses.

"It's just going to be hard for them to hear news from me and not view it as significant and it's hard for me to communicate, 'Oh, I found this thing but it doesn't matter.‴–internist #1

Several described their strategies for describing the findings, emphasizing the opportunity for prevention of adverse health consequences. Several participants emphasized the perceived chronicity and size of the infarcts as a means of reassuring patients.

"I tell them it looks like in the past they had a small stroke they were likely unaware of. . . . we should work this up, then we can prevent larger strokes in the future."–general neurologist #4

#### Managing uncertainty: Therapeutic

**Individualizing care.** In the absence of treatment studies, participants described several overlapping strategies for patients with SBIs: tailoring approaches to the suspected mechanism of infarction, and individualizing care (particularly with regards to age, infarct chronicity, and vascular risk profile). Most internists and general neurologists emphasized risk factor modification as their most consistent response to SBIs.

Several participants across all specialties described similarities between their approaches to SBIs and symptomatic strokes. This approach was most uniform among vascular neurologists who uniformly viewed stroke prevention after detection of SBIs as “secondary prevention.” For example, they described pursuing tests for stroke etiology similar to those used for symptomatic stroke but with variable degrees of comprehensiveness.

“I would look for afib. I would do an echo. I would do the vascular studies if they were not done. I really try to understand why they had the stroke.”–vascular neurologist #3

Participants expressed inconsistency in their referral practices, with internists sometimes referring to neurologists and general neurologists sometimes referring to vascular specialists.

Most participants described using the discovery of SBIs as an opportunity to counsel on lifestyle changes, focusing on tobacco cessation, followed by exercise, diet, weight loss, and treatment compliance.

Several participants across all specialties described initiating or modifying treatments including antiplatelets, anticoagulants, antihypertensives, statins, and antiglycemic medications (extrapolated from guidelines for stroke secondary prevention). Of these, antiplatelets and statins were the mostly commonly cited.

"If they have a silent stroke and are not on an antiplatelet, at least I'll give them aspirin."–internist #6

### Resolving uncertainty

*Resolving uncertainty* focused on evaluation of new evidence and anticipated translation into practice (Table 5).

**Table 5:** Resolving uncertainty–evaluating evidence.

Categories (Level 1)	Subcategories (Level 2)	Subcategories (Level 3)	Representative quotations
Evaluating evidence	Accepting new evidence	Observational CER RCT	“Definitely. Yes. Absolutely. I think I would still love to see a randomized trial, but I think if there was a large enough observational study that demonstrated (a treatment effect), it would be enough for me to change my practice.” (GN3) "I think an all-comers observational study probably wouldn’t change my management." (GN2)
	Devaluing observational studies	Generalizability	“The main thing I'd want to know about the methods section is how were patients selected for asymptomatic stroke. In the end, I think these people get scans for many reasons. Those reasons are heterogeneous.” (VN4)
	Skepticism about RCT feasibility	Feasibility	“I think it (an RCT) is actually impossible." (VN3)
		Equipoise	"I would have a little trouble telling someone not to take aspirin and a statin when I found a stroke on their head CT." (IN3)
		Recruitment	

#### Resolving uncertainty: Evaluating evidence

**Accepting new evidence.** Participants differed by specialty regarding their willingness to alter practices based on findings from CER and RCTs. All internists expressed willingness to incorporate findings from observational studies into their practices without requiring RCTs. Half of the general neurologists and half of the vascular neurologists cited the need for data from randomized studies to alter practices.

"It's really difficult to convince people to do something, just based on observational data if the data were not randomized."–vascular neurologist #3

**Devaluing observational studies.** When asked about concerns regarding observational studies, a few participants cited specific concerns. One internist and one general neurologist emphasized that observational studies are unable to prove causality. One vascular neurologist raised concerns about generalizability with regards to heterogeneous indications for the scans detecting SBIs.

**Skepticism about RCT feasibility.** Several participants, including those describing that they would need RCTs to alter their practices, cited concerns about the feasibility of conducting RCTs.

"I don't think of silent stroke as something that I identify often on an anecdotal basis. I definitely worry about recruitment. Also, like an effect size, we're talking about a treatment effect. I would imagine you'd have to have a pretty big n. I have concerns about feasibility."–vascular neurologist #4

Despite uncertainty about the management of SBIs, lack of equipoise at the individual clinician level was described by a few participants across all specialties as a major barrier to the conduct of RCTs. These participants described an unwillingness to enroll patients with SBIs in trials testing the efficacy of aspirin for stroke prevention.

"If they have known vascular risk factors, to not give an aspirin is probably malpractice. I wouldn't recommend that in the study."–general neurologist #4

## Discussion

Despite prior research on SBIs in screened cohorts, clinicians in our diverse sample expressed uncertain and conflicting views regarding id-SBIs [1–4]. Even with the groundwork laid by these prior studies and the AHA/ASA scientific statement, we found considerable lingering diagnostic and prognostic uncertainty that could impede clinicians from addressing this condition [7]. Practice variation and discordant views on incorporation of new evidence described in this study highlight the need to improve our understanding of id-SBIs—an unstudied condition that may differ from SBIs in screened cohorts in etiology and risk of adverse outcomes—before addressing treatment efficacy.

In this study, diagnostic and prognostic uncertainties were the primary barriers to addressing id-SBIs, including inhibiting the essential first step in management: disclosure to patients. The main areas of diagnostic uncertainty were difficulty defining id-SBIs, uncertainty about underlying causes, the incidental nature of their discovery, and difficulty interpreting neuroimaging. Difficulties with establishing a radiologic diagnosis in the absence of symptoms were particularly important for internists in our study: they uniformly cited reliance on certainty in radiologists’ language in reporting neuroimaging findings. However, radiologists may not consistently identify or emphasize these findings—reinforcing lack of awareness of id-SBIs or their clinical significance among internists. Consensus radiologic criteria have been proposed, but it is not known if radiologists routinely follow them [11]. Considering that internists far outnumber neurologists, most patients with id-SBIs are likely encountered first by internists. It would be infeasible for all patients with id-SBIs to be referred to neurologists for further management given their probable high prevalence. Accordingly, efforts to improve reporting of id-SBIs by radiologists with standardized definitions and greater certainty will be essential for the identification of patients with id-SBIs, the conduct of treatment studies, and the management of id-SBIs by internists.

With or without diagnostic uncertainty, a few participants endorsing considerable prognostic uncertainty actively ignored id-SBIs, defaulting to assuming low risk of harm. By contrast, several viewed id-SBIs as having prognostic significance (e.g. stroke risk) and proceeded with disclosure to patients. However, both assumptions of risk are unproven since outcomes after id-SBIs have not been studied. A precise estimate of risk will be difficult to obtain as there is likely heterogeneity in the reasons for which these patients undergo neuroimaging. Nonetheless, excepting perioperative id-SBIs, some id-SBIs may portend similar risks as SBIs detected in screened cohorts. Based on the responses in this study, a stroke prevention eligible group of patients clearly exists. Even if screening were implemented in the future, SBIs eligible for stroke prevention will continue to be discovered incidentally. For all of these reasons, further research is needed to understand the factors that influence prognosis and treatment benefit for these patients.

**Fig 1:**
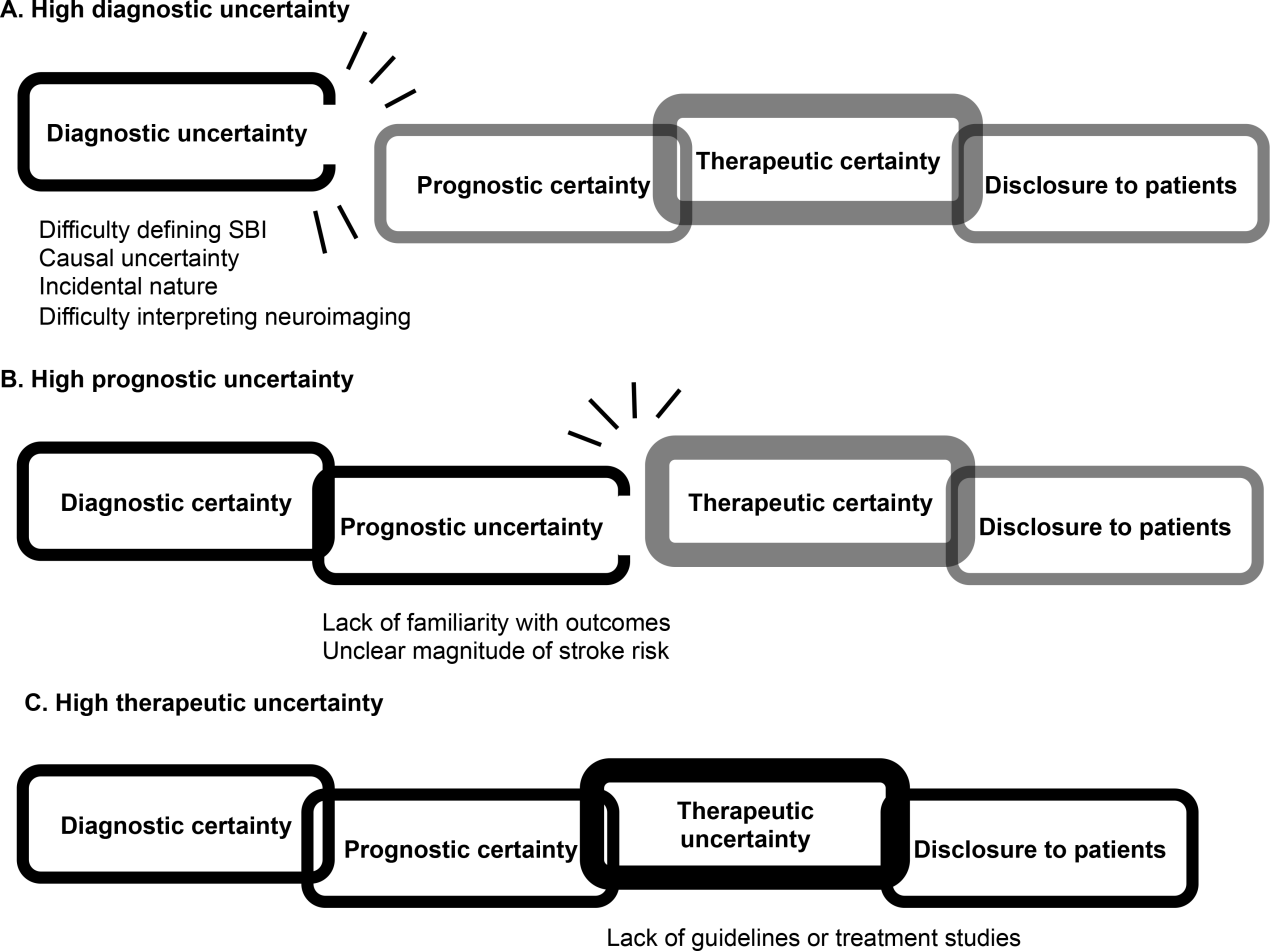
The broken chain: A theoretical model for the influence of different types of uncertainty on disclosure of id-SBIs to patients. The “chain of links” indicates a sequence of clinical reasoning, starting from diagnosis and ending in disclosure of the neuroimaging findings to patients (i.e. to disclose incidental findings to patients, clinicians generally want a degree of certainty about the precision of diagnosis, the potential for adverse health outcomes, and the utility of medical therapies). A, B, and C indicate three scenarios where uncertainty predominates in diagnostic, prognostic, or therapeutic domains. Uncertainty in a specific domain is indicated by an incomplete oval (link) and a description of specific foci of uncertainty below the figure. Black outlines on the links indicates the progression of clinical reasoning to a point where clinicians are halted by uncertainty. The thick-walled link highlights the ability of clinicians to manage and tolerate their own therapeutic uncertainty (unlike diagnostic or prognostic uncertainty) and proceed with disclosure of the neuroimaging findings to patients despite this uncertainty. In other words, the process of disclosure to patients is more vulnerable to clinician diagnostic and prognostic uncertainty.

Unlike diagnostic and prognostic uncertainties, therapeutic uncertainty did not affect disclosure to patients or the decision to alter care. The differential effects of diagnostic and prognostic uncertainties versus therapeutic uncertainty on the process of disclosure to patients is depicted in a theoretical model in Fig 1. Most participants appeared comfortable practicing in the absence of treatment studies or guidelines: they described practices extrapolated from stroke prevention—i.e., primary prevention, secondary prevention, or an intermediate approach. Importantly, secondary stroke prevention is more aggressive, involving more medications and testing [12–13]. In this study, vascular neurologists uniformly endorsed secondary prevention strategies whereas internists and general neurologists often endorsed a variety of less aggressive approaches. Aggressive management was linked to a belief in the prognostic equivalence of id-SBIs and symptomatic strokes. This strong belief is incompatible with the relatively conservative AHA/ASA suggestion to implement primary prevention strategies for patients with SBIs [7]. Consequently, this study suggests that some clinicians in each specialty may continue to offer more aggressive care. Furthermore, this belief is also expressed in the lack of individual clinician equipoise where a few of the participants expressed reluctance to enroll patients in clinical trials assessing treatments considered to be the standard of care for secondary stroke prevention (e.g. aspirin). This practice variation is suboptimal for patients, particularly those encountering multiple clinicians, and the assumption of prognostic equivalence may impede proper assessments of the efficacy and risks of prevention therapies for these patients.

This study had several limitations. Because the interviews are not anonymous, some participants may have misrepresented their practices, including disclosure to patients. The study sample was relatively small and restricted to a limited geographic area: this is a universal limitation of qualitative interview studies. Nonetheless, the sample size is comparable to similar qualitative interview studies and was sufficient to achieve thematic saturation [14–15]. Future survey studies in a larger clinician sample will be useful to determine if the findings of this study are generalizable. Finally, this study assessed clinicians’ beliefs but did not measure clinical practices: a quantitative analysis of clinician practices for patients with id-SBIs can better assess this. Nevertheless, this study also had several strengths. Our multidisciplinary research team included internists, vascular neurologists, and a radiologist with a broad range of clinical experience and perspectives. The participants represented a diverse group of clinicians in academic and community practices, seven hospitals, three specialties, and inpatient and outpatient settings. All participants had experience providing care for patients with SBIs, enhancing the validity of this study’s findings.

## Conclusions

id-SBIs are understudied and involve several uncertainties that foster practice variation and complicate the development of a standard of care. Further research is needed to reduce uncertainties about this form of SBI in order to facilitate treatment studies and evidence based clinical practices. Until then, disclosure to patients and acknowledgment of uncertainty may be useful practices in the individualized care of patients with id-SBIs.

## Supporting information

S1 File Interview transcripts. Transcribed interview transcripts for fifteen clinician participants.
(DOCX)

S2 File Interview guide. The final version of the interview guide.
(DOCX)
