# Correction: Clinicians’ perspectives on incidentally discovered silent brain infarcts – A qualitative study

**DOI:** 10.1371/journal.pone.0197706

**Published:** 2018-05-16

**Authors:** Lester Y. Leung, Paul K. J. Han, Christine Lundquist, Gene Weinstein, David E. Thaler, David M. Kent

The sixth author’s name is missing the middle initial. The correct name is: David M. Kent. The correct citation is: Leung LY, Han PKJ, Lundquist C, Weinstein G, Thaler DE, Kent DM (2018) Clinicians’ perspectives on incidentally discovered silent brain infarcts—A qualitative study. PLoS ONE 13(3): e0194971. https://doi.org/10.1371/journal.pone.0194971
