# Supplementary material for: Clinicians’ perspectives on incidentally discovered silent brain infarcts – A qualitative study
Source: PLoS One. 2018 Mar 29;13(3):e0194971. doi: 10.1371/journal.pone.0194971 (PMC5875806; doi:10.1371/journal.pone.0194971)
Supplement: S1 File — Transcribed interview transcripts for fifteen clinician participants. (DOCX) [file pone.0194971.s001.docx]

**S1 Interview Transcripts.**

Order of Interviews:

1. Internist 1 (IN1)
2. General Neurologist 1 (GN1)
3. Vascular Neurologist 1 (VN1)
4. Vascular Neurologist 2 (VN2)
5. Internist 2 (IN2)
6. General Neurologist 2 (GN2)
7. Internist 3 (IN3)
8. Internist 4 (IN4)
9. Vascular Neurologist 3 (VN3)
10. Internist 5 (IN5)
11. General Neurologist 3 (GN3)
12. Internist 6 (IN6)
13. General Neurologist 4 (GN4)
14. Internist 7 (IN7)
15. Vascular Neurologist 4 (VN4)

Internist 1

Study ID: IN1

LL: So this is internist number one. And it’ll take about-there are about ten questions.  And there will be some potential side questions as well depending on how you answer.

LL: So the first question is just tell me what you know about silent strokes so far.

IN1: Everything possible.

LL: Okay.

IN1: No no, like everything, so you want me to say everything I know about silent strokes so far.

LL: Yeah. Whatever you know. In summary.

IN1: This… this includes white matter disease.  Just like white matter dots that you see on MRI.

LL: Sure. If you’d like.

IN1: Okay. So I guess my understanding of silent strokes is that there's a range from just microvascular disease that comes from chronic hypertension and maybe, other subclinical thromboembolic disease… ranging to unrecognized a-fib and full-on, you know, kind of anatomically obvious strokes that occur without a patient’s knowledge and can happen for a variety of reasons.  Probably the most common cause would be hypertension, atrial fibrillation, much less commonly going to be other causes of arterial thrombotic disease and probably more likely in folks with many vascular risk factors like diabetes.  Migraines.  What else. Other neurologic-other neurological issues.  It's also obviously- it’s increasingly common with age and I think there really aren’t that many folks over the age of seventy who don't have at least some low level burden of some kind of microvascular disease, whether it's clinical or subclinical.  Smoking.  Of course, also a big risk factor.  And you know, I think the clinical relevance of them is highly variable.  And usually where it comes up most is in folks where we’re trying to come up with a differential diagnosis for possible dementia. And if it’s vascular and we think if they have enough silent strokes that would implicate vascular dementia.  And then that would be, a sort of a different, it may have different implications and a treatment strategy than if we think they have Alzheimers or Parkinsons or something ... Something else.

LL: Oh, sure.

IN1:  So that is most of what I know about silent strokes.

LL: Ok good, no, that's a good start. So it's a diagnosis that has several names that have been described in the literature including silent stroke, silent brain infarction, silent cerebral infarction, covert stroke, covered brain infarction, subclinical stroke, subtle stroke, asymptomatic stroke. Of these terms, which are the ones that you've heard used and which do you use most often?

IN1: I don't really know. Can I see the list again?

LL: Sure. Up hear.

IN1: I think I may say subclinical or asymptomatic.

LL: OK, good. Are there others you've heard in reference to this phenomenon?

IN1: I don't think so. I think it sort of in that kind of asymptomatic-subclinical area. I think it's mostly.  And then I think... I think it's whether or not the... I think the only other thing is you know just the sort of white matter disease you see on MRI almost ubiquitously in older folks. You know just kind of refer to it as radiologists do it just as age related white matter disease or as microvascular white matter disease.

LL: And for you, of these terms what do you think is the most appropriate or useful term to use considering that there's a whole plethora of different ways that people are referring to this? 

IN1: Probably subclinical is the most helpful because it makes it clear to me that somebody has had a stroke but the clinical manifestations are not obvious, whereas if somebody had a stroke where the clinical manifestations were important, then like, then that would just simply be a stroke. Whereas if they have a subclinical stroke that means that there is some unrecognized or previously undertreated-potentially undertreated-vascular risk factor.  And it kind of makes me worried that they have other vascular disease elsewhere that I need to think about. Heart. Elsewhere. Kidneys.

LL: Why do you think covert strokes or silent stroke occur?

IN1: I think mostly what I covered earlier. I think like most vascular risk factors like hypertension, smoking, diabetes.  Anything that causes, you know, like intimal thickening.  You know, atherosclerotic disease.  Plaque rupture.  Afib is probably, I imagine Afib is probably responsible for, like, the lion's share of these, as well as hypertension-I would guess-are probably at least seventy-eighty percent.  And then I think there's probably other, you know, bizarre causes.  Maybe kind of vascular disease related to migraines.  Cancer.  What else.  Some other kind of hypercoagulable-other other hypercoagulable states which are, which tend to be relatively uncommon, particularly in the arterial circulation.

LL: Sure. So that covers-

IN1: Lupus, autoimmune disease, things like that.  You know, lupus is probably a cause, but if someone with lupus had strokes, like, you would probably, I mean, I guess, you would mostly suspect their lupus.

LL: That covers the risk factors and mechanisms of disease end of that question. It's actually sort of a two part question cause, depending on how your interpret it, but the other side of it is why-why are these silent, why are these subclinical?

IN1: Oh. Well, I think because... you know, I happen to like and read a lot about the brain. And have a lot...

LL: Sure, sure.

IN1: You know, I think the reason why is because: A, there's a lot of redundancy, I think, throughout the brain, so it's not like, I think, I think, the construct like there's a single area that's responsible for any single thing.  I think also a lot of these strokes happen. Don't necessarily, you know, go all the way up the cortex where a lot of the kind of most essential functions are.  And I think it's going to be hard for people to notice a lot of these changes because of the nature of just how consciousness works so if you have, like, if you have occipital silent stroke which is probably pretty uncommon, but you did have that people aren't going to be aware of, like, a small visual field defect.  Particularly it doesn't affect things like driving or something.  You can just, you can compensate by just looking around. There are probably many ways of people compensate in ways they're completely unaware of.  And it wouldn't be a silent stroke if it that happened in your motor strip or if it happened in your, you know, your primary sensory regions and then you notice you can't feel anything.  And I think a lot of these a lot of these are probably affecting more subtle, more subtle areas of cognition like memory or reasoning or emotions, things that you can easily attribute changes in those as you get older to a variety of things.  And so they don't, it's not like one day you wake up and suddenly you can't move your pinky and you're like,"What happened?" It's just that you're a little more irritable.  Or you're a little more forgetful. Or over time you get a little clumsier. And I think those things are hard to point to any one thing. So that's, I think, generally how I think about it. Though every now and then you run into someone who has, like, a really big stroke. Like a big parietal stroke, and it's just, like. (laugh) How did this never-how did this never come up? And it is kind of.  You know, this kind of.  You know, it is kind of impressive. But people are really able to. I think people can really adapt to a lot of things and come up with rationalizations for what's going on that particularly when It's something that you're not-doesn't cause any pain and you're not necessarily conscious of.  You don't feel a difference because it's your brain; that's how you perceive the world. And if it's gone, you don't perceive that's gone.  It's just...you... you don't have that function anymore.  Then the rest the brain is not aware of it.

LL: Fair enough. I think you've already covered a lot of ground in terms of answering the next question.  The next one is from your perspective in what ways are silent or subclinical strokes the same or different in terms of their pathophysiology as compared to more typical strokes that present with symptoms or deficits?

IN1: Right.  So I think it's just the territory size that, you know, if you have a big MCA... if you have a blockage of a major branch of one of the cerebral vessels, you are immediately disabled and clearly in extremis, you know, from anyone who can observe you. And most likely yourself and you probably have a change in your state of consciousness.  Whereas for these small strokes, unless they hit a very choice area, like the brainstem or some... it's pretty... you have a lacunar stroke or something... then it's probably just not going to be obvious.  So I think it's probably mostly just a function of the size and severity and also maybe the extent to which it causes edema in the surrounding structure so that kind of it becomes sort of a global intracranial problem... which is going to be much more symptomatic that if just have a little small area of inflammation.  Like people have brain mets. Many many brain mets and be completely asymptomatic because they grow slowly over time and they only effect very small areas of the brain at any individual moment.

LL: So, if I'm hearing you correctly, it's mostly a matter of location and size. It sounds like the mechanisms are pretty much the same. My next question is actually going to be about risk factors but I think you already talked about that in terms of things like hypertension and atrial fibrillation being... taking the lion's share in terms of the major causes of these strokes.  But also other things like you mentioned.  Tobacco and migraine and hypercoagulability and cancer and so on. In terms of settings in which you've encountered the diagnosis... What have those settings been like?

IN1: Well, I mean, I'm not going to discover it unless I get brain imaging on someone because we're calling them subclinical. So I don't... my... you know I think usually... you know, it's really hard.  The neurological exam is particularly... the way a PCP does it is gonna... we're going to very hard time picking this stuff up.  Or we'll just notice what probably is a false positive but it will trigger a, you know, brain imaging that, you know we're finding a silent stroke but not because it actually had an exam finding but just because I thought there was an exam finding. So usually it's in the setting of head imaging for something else like... they fell and we thought they had their head. Or they have... or we're doing the headache workup.. or it's a de... memory loss, or dementia workup. Or they got admitted to the hospital for some other reason or they had some kinda of trauma.  So I think it's almost always in the setting of.. or unfortunately sometimes... someone gets gets to the hospital, they get delirious.  Just like something else that or other... or syncope. They have common causes for head imaging.  And then we just see that it's there.  But it rarely comes up that I'm looking... that I'm looking for them.  I think probably the most relevant clinical situation would be the dementia workup and I want to do head imaging.  And the thing we probably most likely learn from that is what's the contribution of vascular dementia.

LL: Seems like memory loss, dementia, falls, syncope, headache.

IN1:  Headache... that's going to be uncommon.

LL: Ok.

IN1: Yeah, I think that's most... syncope, or, any TIA. Of course, if we're worried someone had a stroke or a TIA, then you know, we would do that. And sometimes what the reveals is that maybe it wasn't a TIA at all but they actually do have, they have old strokes.

LL: In these cases, do you end up connecting whatever their original symptoms were to these findings on the MRIs?

IN1: Almost never. 

LL: OK.

IN1: Except in the setting of vascular dementia or maybe someone who's failing to thrive at home and they're living alone and they're not doing very well. And then we will... and that will just be one part of the explanation of why they're not doing very well.  It's not, it's not never but it's pretty uncommon. I think, I can think of a few examples where maybe somebody came in with TIA-like symptoms and then they had actually untreated AFib and it looked like they had evidence of old prior strokes and that was you know an important finding.  And so that's maybe happened... it's maybe happened a couple times that I can think of.

LL: Good.  So these subclinical or covert strokes are often encountered incidentally, just like you're describing when physicians are investigating a symptom or a medical problem that doesn't overtly seem to be related to stroke. But what does it mean for you as a clinician for something to be incidental?

IN1: Anything or a silent stroke?

LL:  Just anything actually.  And then I'll ask you specifically about this issue.

IN1: I mean it depends on what it is. In the case of a silent stroke, probably more often than not I do nothing.  Maybe, I think in the presence of better guidelines, and I think, I feel like I'm reading a little more about we should be more aggressive at screening for Afib. But if someone is in sinus rhythm every time you see them, it's a little hard.  We don't, we don't really have a lot of guidance right now to monitor everyone for thirty days. How long do you need to monitor somebody and what cost and inconvenience to them before we can confidently rule out Afib when the option for many people is still Coumadin and that's a very big commitment for people.  So we don't really have a lot of guidance there. In terms of other incidental findings, I mean, if they are a cancer risk, then you have to continue to follow them. Otherwise I tend to ignore them... like everybody... so many people have renal cysts or adrenal nodules or liver cysts and I tend to really just ignore them. Or like fibroids in their uterus.  If there's something the radiologist says needs to be worked up or followed like a lung nodule then I will do my best to follow those.  But besides that, I tend to... I try to minimize the incidental findings as much as possible.  I tend to mention them to patients but depends on the patient, because I feel like some patients... they just... it's going to be hard for them to hear news from me and not view it as significant and it's hard for me to communicate, "Oh I found this thing but it doesn't matter." Because if I mention it to them, then it must matter. And they don't have the sophistication to understand that sometimes you find things that don't matter.  But trying to be... a good doctor, I'm... I want them just to know what's there.  But sometimes I think that backfires 'cause then you kind of go down a rabbit hole of trying to have a whole conversation about something that doesn't matter.  But I just didn't want them to be told by somebody else and then think that I'm a bad doctor 'cause I didn't tell them what was on their CAT scan.  So it's a very hard communication challenge.  It's often easiest to just simply ignore it because I feel like my job as a PCP is to give my patient the most important medical information... what's the big picture that's important.  Like, you should lose weight. Not worry about this renal cyst that is almost certainly absolutely nothing and if I tell you about it and you're going to tell everyone forever that you have a renal test and like think about it as a diagnosis when really it's nothing.

LL: The next four sub questions you can answer briefly if you want to because I think you've already applied the framework of how how you respond or not respond to these. But, I'm going to ask you about treatments, testing, referrals, and also recommendations for lifestyle changes or behavioral modifications. So the first is, do you generally start or modify treatments for patients with silent strokes?

IN1: I don't think so. I probably do... I probably do a mental checklist and see if they're on an aspirin or not.  I think whenever I find it it's almost always in somebody who already has... some kind of vascular disease and is already on a treatment regimen.  But I think that's what I would think of.

LL: Do you order additional testing?

IN1: Basically never. 

LL: Do you refer these patients to anybody?

IN1: No.

LL: And... do you recommend any particular behavioral changes or lifestyle modifications?

IN1: I think it's usually not.. I think it's usually, it doesn't necessarily... It's rare. It's rare that it will ever be something I find in isolation where it's not clear that they need to work on their diet for diabetes or quit smoking or... maybe they smoked for forty years and now they quit and that's their major risk factor and they've already made the change.  And so I think it's.. I think it's relatively uncommon that I would... that anything... that there's something new that I would advocate for with that information that I can think of.

LL: Do you think... so we've talked about this as being subclinical disease, but do you think that these subclinical or covert strokes have caused any harm to patients?

IN1: I think it's probably on the spectrum for vascular dementia. So a lot of my older patients are probably more likely to fall and have a poor outcomes that they have more of these strokes, so I'm sure. But I don't, I don't know if it's causal or not because they're probably more likely to do poorly for the reasons that cause the stroke as well.  So I don't know how causal they are.  So I mean I don't, I don't know and it's an... I think it's an open research question and unless probably there has been some work on it but it's really hard to tell. I think the only... like the only difference is just that if someone's brain is riddled with small strokes then you know they probably have dementia, but you probably knew that something was wrong, anyway, before you had that happen. So I think it's really, really hard to implicate silent strokes for any individual patient as the cause of the problem.  I think without some... better understanding of where does it cross the line from being subclinical to actually affecting outcomes.

LL: Do you think that these covert strokes or subclinical strokes have... do they put patients at greater risk for other health issues, and if so, how much risk?

IN1: Like I said I don't... I don't know that the strokes themselves put people at greater risk for things. I think it's more a symptom of their risk for overall vascular disease. But, I think probably it's most relevant in geriatrics where probably these strokes put older folks at a higher risk for falls, for frailty, for disability. Kind of all of the things that start older patients on the trajectory of... dwindling... quality of life and overall status.

LL: This is an extension of this past question and I'm basically going to give you a laundry list of things things that could potentially be things that silent strokes could people put people at risk for. They're certainly... the answers are not all of yes or all no. So you can just say Yes or No or give a, if you want, a qualified answer for a particular item. But it's just... I'm going to go down the list. So, again the question is do you think that covert strokes placed your patients at risk for any of the following?

LL: The first is stroke.

IN1: Yes.  

LL: Intracerebral hemorrhage.

IN1: Probably

LL: Myocardial infarction.  

IN1: What do you.. what are you implying about... you're not implying causation here, right? It's just that the presence of silent stroke indicates an elevated risk for these things, right?

LL: Sure.  

IN1: I don't think.  I don't think having a silent stroke is a causal relationship. But it means you have vascular disease in general so you have a higher risk of MI.

LL: Sounds good. Heart failure.

IN1: Yeah.  

LL: High blood pressure.

IN1: Right. Yes, in the opposite direction.

LL: High cholesterol.

IN1: Yeah.

LL: Diabetes.

IN1: Yeah.

LL: Headache.

IN1: I don't think so.

LL: Vertigo.

IN1: Probably not.

LL: Seizures.

IN1: I don't think so.

LL: Falls.

IN1: Yes.

LL: Memory loss.

IN1: Yes.

LL: Dementia.

IN1: Yes.

LL: Anxiety.

IN1: I don't think so

LL: Depression.

IN1: Probably

LL: Schizophrenia.

IN1: No.

LL: OK. How comfortable do you feel treating patients with subclinical or covert strokes?

IN1: I mean, I think I feel pretty comfortable... I think it's not clear what to do about them so it's not like there's anything to feel comfortable or uncomfortable about as we've discussed before. So I don't think it's something that makes me that worried.

LL: You've already answered the next part of this question which is: Do you follow any practice guidelines?  And I think you already alluded earlier that you don't think that there are any practice guidelines.

IN1: I don't think there really are any. And like I said, I think... and they're probably... I think the things to think about are... overall vascular risk management like statin and aspirin and then I think there's screening for Afib.  But I think there's really no clear guidelines on how to do that right now.

LL: What do you think are the major knowledge gaps in this area?

IN1: I mean, I think they've come out. I think...  Is there is a.. it is there a pathway to follow? I think screening for Afib is probably the biggest one.  Because if somebody has silent strokes then Afib could be the cause and that needs to be treated before they have a devastating stroke.  And so, to what extent... how aggressively should we screen these folks for Afib... cause they may only have Afib once a month maybe.  But do you try to catch that? Or they could have less often.  It could only be in the setting of stress... So what you do about that?  I think... I don't know that anybody knows what to do for folks who have silent strokes in the absence of other vascular risk factors? How do you manage that?  I don't know. Because a lot of people silent strokes are going to have diabetes and they're going to have coronary artery disease. They could have other vascular disease.  And so it doesn't really change the management from the primary care perspective.  And then I think the other thing is I don't know what the definition of silent stroke is. So you know... how big does a blip need to be on an M.R.I. or a C.T. scan before that becomes kind of a clinical condition that needs to be managed. I don't think anybody... I don't think there's any consensus on that.  And then...I think that's probably pretty good start. Those are a lot of questions.

LL: What would... are there other things that you would want to know more about to help you in your practice?

IN1: I mean, I think the questions I just, I think... basically the questions I just had.  I think maybe when would a referral be beneficial?  And what would a neurologist add in that situation because it's not clear to me there's really much to do? Except you're add an aspirin or a statin and just stick to geriatric best practice... best practices. In terms of fall risk assessments and doing regular cognitive assessment, things like that.  So I think, those are the big things.

LL: This last question... which has a couple parts is a little bit of a thought experiment.

IN1: OK.

LL: But let's say there is a well designed, rigorous, observational study that's a comparative effectiveness study. And you've actually described I think that in most the most of these cases you don't really change too much in terms of the medical management of these patients. But let's say that this comparative effectiveness study was looking at patients with silent strokes in order to prevent late term consequences of silent strokes and they found that it was actually useful to start people on an antiplatelet and a statin, just sort of, out of course. Everyone should be on an antiplatelet and a statin, including people who don't have other vascular risk factors. Would that be something that would actually convince you to change your practice if such a study were done?

IN1: Well, I mean, it depends on what... what's the outcome in the study?

LL: Let's say the outcome is recurrent stroke.  So stroke with symptoms and deficits. So, just like you're describing how aggressively we should screen patients for atrial fibrillation, let's say the outcome you are trying to prevent is having a stroke with symptoms or deficits after a silent stroke?

IN1: I mean I think, I think it, I think it might change my practice some.  But I think it again begs the question of... then do we screen everyone for silent stroke and do we use M.R.I. or C.T. scans?  And then how many other incidental finding do those bring up that then cause other unnecessary work ups?  So I think that may be it's a first step but it's not the... it's not... that wouldn't be the only piece of evidence I would need. I think you really need to get to kind of a real quality number that incorporates the cost of the incidentalomas, the imaging, along with the actual benefit because the marginal population that it benefits is potentially pretty narrow. And to implement it effectively you need to do a lot more imaging.  And so that's pretty costly.  So I think that's... I would really need to see that. I'd really need to see that before I start sending my patients. I mean, if a patient gets imaging and then I see a silent stroke, so I'm like, "Well you're not on an aspirin, so like, you might as well be like you." It could affect me there but that's going to be a pretty uncommon occurrence.

LL: OK. Do you think that there would have to be randomized control trials to try and look at patients with silent stroke? Let's say that there was a rationale for screening that was designed and it was reasonable to do so. Would you need an R.C.T. to convince you in terms of what medications you should start people on?

IN1: No, I mean, I think for this, it would be more about the cost effectiveness. What I really want to know is... because it's actually not about the medication. It's about the screening for the silent strokes which is what I would be most worried about because you know many many many people end up being on a statin and an aspirin.  And I feel like they're pretty well tolerated and they're not drugs that I feel particularly disinclined to prescribe and I feel it's a relatively narrow slice of the population that needs this. But I need to... but then I need to add yet another thing in my checklist and we need to have, kind of, another thing to potentially screen our older patients for which is an enormous cost the system, inconvenient to our patients, adds more time to our visit.  It's kind of an enormous.. it would be an enormous extra burden on the primary care system and I want to make sure that the benefit is worth the cost. Just like mammograms, PSAs, and colonoscopies.  But we know that those find and prevent things before they... you know... turn into life threatening issues. PSAs and mammograms, not necessarily.  So I think that's... so an RCT... I mean I guess, "Sure," might be beneficial. If you can identify the high risk population. But then you have to design the inclusion criteria for the RCT very carefully, and that's what I care about is really "what are the inclusion criteria?"

LL: In any studies for patients who have these, are there particular outcomes that you would find important for your patients?

IN1: I think... think disability. Quality of life.  I think stroke... I think stroke is a very important outcome. Mortality, obviously, but that's hard. I think it's really about stroke and disability.  I think if we... if we're not preventing poor neurological problems and disability or falls, things that we want to avoid in our older patients, if it doesn't affect quality of life then I'm not necessarily that interested in it.  But I think... I think stroke could be a surrogate measure there because we know strokes are terrible. So... nobody needs to be convinced that strokes are... something that's important to... nobody needs to be convinced that strokes are something to avoid. We treat Afib very aggressively for that reason.

LL: Let me give you just a statistic. There's been a number of large prospective covert studies like the Cardiovascular Health Study, the Framingham Offspring Study, Rotterdam, NOMAS... that have actually looked at silent stroke as one of the outcomes.  And they found that over age fifty the prevalence of having a silent stroke is about twenty percent, including these populations of people who are actually often when they enter these cohorts are actually relatively healthy. And so knowing... so thinking about that number, twenty percent as the prevalence, and it's certainly something that increases with age, so people start on the lower end of the spectrum and then it ramps up pretty quickly after that. How, both in your own practice as a primary care physician but also thinking on broader scale.... how would you approach dealing with all those patients who have this diagnosis if you are to find there are guidelines or there are studies that say... that describe that there is an effective way of preventing stroke or disability following silent stroke. How would you deal with all those people who you ready know have this problem?

IN1: So you're talking about population management?

LL: Both in your own practice but also in terms of the overall population.

IN1: Yeah I mean... so that's a big issue for health systems across the country... for anything.  I mean, it's not like we figured out everything else in terms of... nobody... I don't know that anybody has a one hundred percent effective strategy for colonoscopy or lung cancer screening or AAA screening or screening for dementia.  You know, all these things.  So I mean... I think every practice does it differently, for better or for worse.  And there does need to be a strategy implement these population wide screen screening strategies. Right now our health system does it pretty poorly and you just need to devote resources to it. And you see, something that's important enough to health systems. This is something that probably health systems would like because they get to make money off of it but it's hard to get patients to show up for these things. So I'd say it's no different than what we would do for colonoscopies where every year there are hundreds or thousands of patients in many practices, well maybe hundreds of patients, who are due for their colonoscopies and you need to call them and nag them to get... to come in and show up and get, make this happen. And every year people will fall through the cracks and you always have overdue people and people who are due.  And it just takes resources with people calling and having a reminder system and making sure you follow up on abnormal results and all those things.  And it's not easy. And it's public health.  So, but I think, I think you can understand my resistance a little.  I mean, I just, I would need a fairly high burden of proof before I accept a new process of screening and treating these... this condition... like we do for other things, without a really strong level of evidence that it benefits certain people. Which we don't have right now, but we should look at. Just like we know the AAA is cost effective for people who have smoked who are over fifty.  But it's only once. How often should we do screening? At what age? When do we do it again?  What counts as a subclinical and what counts as relevant?  You know we don't have any of those definitions.  And so before we even think about population screening we need to decide what's actually important and who's a population that we screen.

LL: Great. That actually brings us to the end of the interview. I just want to leave it open to see if you have any additional questions or thoughts now that we've come to the end?

IN1: No, that's great. Sounds like an interesting research area, lots to do.

LL: Thanks. I'm going to stop the recording right now.

END OF INTERVIEW

General Neurologist 1

Study ID: GN1

**Q:** Okay, so we're going to get started with general neurologist #1. So the first question is tell me what you know about silent or covert strokes so far?

**A:** Silent or covert strokes? I'm not sure what you mean, what do I know about it?

**Q:** Just what are your thoughts about it?

**A:** So my thoughts are generally that a silent or covert stroke would be an incidentally found evidence of prior infarcts on brain imaging in a patient who does not recall any history of symptoms that would refer to that stroke.

**Q:** Exactly, yeah. So I think we probably have a similar definition in that respect. Anything else that comes to mind?

**A:** Gosh. I mean, I would guess that you would typically see a variety of types of covert strokes. You could see what looks like an old lacunar infarct or you could see something that looks like embolic, like did they at some point shower emboli and didn't notice that?

**Q:** Fair enough.

**A:** Or, is there evidence of maybe-- I guess I don't know if you would group into that like amyloid angiopathy? Like people who have micro hemorrhages in the context of Alzheimer's dementia or--

**Q:** Sure, yeah.

**A:** I suppose that could count also as clinically silent.

**Q:** Yeah, some people would. I'll just mention for the purposes of the studies that we're pursuing, we're mostly focusing on brain infarction. But there are people who consider micro hemorrhages to be a type of stroke. It’s just of the hemorrhagic variety.

**A:** Right. Or, like, I suppose you could also potentially count like asymptomatic or clinically silent sinus thrombosis with evidence of infarction due to venous congestion. I don't know if people count that as well.

**Q:** No, that could essentially count.

**A:** Okay.

**Q:** The next question is an extension of this first one, but this diagnosis, it comes with several names that people have used in the literature, which include silent stroke, silent brain infarction, silent cerebral infarction, covert stroke, covert brain infarction, sub clinical stroke, subtle stroke, asymptomatic stroke. Which of these terms have you heard used before?

**A:** I think I generally personally have used the term either asymptomatic or sub clinical stroke or infarct.

**Q:** And are there other terms besides the ones that we've mentioned that you've heard used to describe this phenomenon?

**A:** Not that I can think of.

**Q:** Okay. Of these, what do you think is the most appropriate or useful term to use? If we were to try and get everyone to use the same wording, what would be the one that makes the most sense to you?

**A:** I think that I personally like sub clinical or asymptomatic because I think with covert, it is not really clear what the implication is. It could be that, like, it doesn't outwardly manifest in some way, but the patient could have some symptoms, whereas asymptomatic or silent could do this as well. I think asymptomatic and sub clinical imply most strongly to me that the patient was totally unaware that anything happened.

**Q:** Sure. So, why do you think silent strokes occur?

**A:** I think that it could occur because you have somebody who has vascular risk factors for either embolic or in situ thrombosis and they could have just a small enough infarct or an infarct in an area that is not like high real estate enough that they could be unaware. Or, it could be in somebody who already has some neurological compromise. Let’s say that they have some difficulties walking or they have some cognitive impairment, and so it’s not as noticeable that they have deficits due to an infarct.

**Q:** Fair enough. What do you think are the major risk factors for silent strokes?

**A:** So, the various risk factors just for small vessel ischemic disease, so hypertension, smoking, diabetes, and then I suppose for embolic infarcts, it could be if they have carotid atherosclerotic disease, or less likely some distal source of emboli.

**Q:** Sure. In what settings have you encountered this diagnosis or this issue?

**A:** So I feel like I probably most commonly encountered it in the inpatient setting. So you would often see that, for example, in a patient who was post-op like from some vascular procedure and they develop some deficits and you go to scan them and they have like a shower of emboli that doesn’t necessarily explain what happened. It’s like they could be delirious and you scanned them for just further workup of the delirium and it was not totally clear whether-- I guess that isn't strictly silent. But I feel like we would scan people for one reason, not expecting to find anything. And then you see like a corona radiata infarct and you're like, well what does that mean?

**Q:** Right, fair enough. In these settings, what were the typical symptoms that patients presented with?

**A:** So I feel like altered mental status was a common one where we would scan people and not expecting to find infarcts or just some kind of transient symptoms that didn't sound very convincing for infarct, whether it was like sensory symptoms or something. Sorry, I'm trying to think back on examples.

**Q:** No problem. Take your time.

**A:** I feel like in my line of work, because I see neuromuscular patients, we get a lot of people who come to us who've had brain scans or scans of their spine, or whatever, as part of workup to try to rule out kind of other complicating conditions when they have something that looks like peripheral weakness just because people often in the community are trying to be extra conscientious in working it up. And then you find something like an infarct and then you're stuck wondering what to do about it. And it pretty clearly doesn’t explain their symptoms.

**Q:** Sure. I think you answered this next question already for the second scenario. I was going to ask you, in those encounters, did you think that the symptoms that the patients presented with were somehow tied to the abnormalities on the scans?

**A:** No.

**Q:** Generally no?

**A:** No.

**Q:** Okay. The next question is these-- silent strokes are often encountered incidentally, just as you're describing when physicians are investigating a symptom or medical problem that doesn't overtly seem to be related to stroke as a potential mechanism. So, just in general, what does it mean for you for something to be incidental?

**A:** For something to be incidental, then generally it’s something-- it’s an abnormality that I found while looking for something else and I didn't expect to see it. And then you have to sort of decide how much weight you ascribe to it and what you want to do with it. So, I feel like it comes up always with aneurysms. It’s just you're looking for something and then you find an incidental aneurysm and it sets you down some kind of flow-charty kind of path.

**Q:** Sure. And as you kind of alluded to there, there's some thought process in terms of figuring out how much of a risk you're attributing to these incidental findings, depending on what it is. So for this particular one, for these silent strokes, do you feel obligated to respond to them?

**A:** Yes, that's an interesting question. I do feel obligated to at least think about why they happen and how to optimize the patient’s vascular risk factors to the extent that it feels appropriate for me in my particular specialty. Because this happens a lot, I feel like in sub specialty neurology, that you want to address all of their problems. But it actually makes more sense to just focus in on what they came for.

But, in the one or two examples I can think of where a patient had like an incidental stroke, I tried to just get a basic sense of their vascular risk factors. Oftentimes, patients are on aspirin anyway, or if they're not, then I say you might want to consider being on an aspirin and talk to them about that. And just making sure that they're kind of plugged in to primary care. There's one situation where I inherited a patient where they had found incidental strokes and actually just made a referral to the stroke center for optimization of secondary risk factors because we have an outpatient stroke neurologist who’s very interested in seeing those patients. So it makes it kind of easy.

**Q:** Great. So you mentioned aspirin as an example. Have there been situations that you've actually started it for patients or made modifications to antiplatelet therapy?

**A:** So I did start somebody on aspirin once, but it was not a silent stroke. They described a pretty good presentation of amaurosis fugax and so they said that they had just had surgery the week before, and then they had a 20 minute period of monocular blindness. And this was a few years prior, but I said, “Have you ever been on an aspirin daily?” And they said, “Yeah, I have, and I stopped and I'm not sure why.” And I said, “Well, do you have any problems with bleeding, blobbity blah,” and I said, “why don’t you start taking an aspirin?”

**Q:** Fair enough. Have you started or modified any other types of medications?

**A:** I haven't, like, changed anybody’s anti-platelet therapy or anything like that.

**Q:** Or like antihypertensives, statins, anything like that?

**A:** No.

**Q:** Okay.

**A:** I have started people-- this wasn't for silent strokes, but I feel like I start a lot of people back on their statin because when they're getting neuromuscular referral, oftentimes primary care doctors have freaked out because of, like, nonspecific muscle aches. And I'm like, “Start it back up.”

**Q:** Fair enough. I think you've also alluded to this, but do you, when you encounter silent strokes, do you order any additional testing?

**A:** So I haven't personally done that, and again I'm pretty early in my practice. But I would consider doing that maybe down the line. Or, I think more realistically, what I would do is I would probably make recommendations to the primary care physician.

**Q:** What would you recommend to the PCP?

**A:** So I think like in the case of this woman with amaurosis fugax, I would recommend a fasting lipid panel and A1c and I'd probably recommend that they do carotid ultrasounds. And if there were abnormalities in the carotid ultrasounds to consider like having her see like a stroke specialist or a vascular specialist of some kind.

**Q:** Sure.

**A:** The truth is I don’t have an algorithm yet. [laughter] I'm sort of figuring it out on a case by case basis what feels appropriate. And with this one with amaurosis, she had some other things that prompted me to just refer her to see one of my colleagues in the stroke center on a one-time basis, which she did.

**Q:** And there might not be an algorithm. I think what you're implying is that it might depend on the patient and what you might think could be an underlying cause of that particular stroke, whether it’s silent or subtle or sub clinical. And so it sounds like for most of these patients, because of being a neuromuscular specialist, you might feel compelled to refer them to stroke specialists?

**A:** Yeah. I forgot to say event monitoring. Go on. [laughter]

**Q:** Sure, sure. Did you want to explain?

**A:** Oh, just that like another thing for-- especially if it sounds embolic, it might be worth it to set them up with a Zio Patch or something like that just to rule out paroxysmal afib.

**Q:** Okay, good. Besides stroke specialists, are there other specialists that you would potentially get involved?

**A:** I mean, not specialists, but I try to get the primary care doctor kind of looped in if I'm seeing something concerning for silent stroke, yeah.

**Q:** Definitely, okay.

[side remarks]

**Q:** When you have encountered these, have you asked-- or do you ask patients to make any lifestyle changes, behavioral modifications?

**A:** I have definitely counseled patients on quitting smoking, if that counts?

**Q:** Yeah, absolutely.

**A:** And if they have pre-diabetes, I've talked to them about nutrition counseling.

**Q:** Good. This next question is thinking a little bit about the idea of risk and harm. But do you think that these silent strokes have actually caused harm to patients?

**A:** Not that I can think of in my case, but I can certainly imagine circumstances where it could cause harm. I don't know if that's worth talking about or if you just mean in my-- I don't think I've ever directly caused harm as a result of it.

**Q:** I mean, the strokes themselves, these silent strokes, these sub clinical strokes. Have those caused harm to the patients?

**A:** Oh, I see. I mean, it’s hard to say, right? I could certainly imagine in somebody who has like some mild cognitive impairment or some very mild parkinsonism, it’s like is it possible that sub clinical strokes are playing a role in that? I think it’s really hard to say.

**Q:** Okay, fair enough. From your perspective, do you think that silent strokes put patients at greater risk for other health issues?

**A:** I don't know if it’s so much that the strokes put them at risk. But I think that the strokes are an indicator of their-- should I talk louder?

**Q:** Maybe a little.

**A:** I don't think that the strokes themselves necessarily place them at higher risk unless they have a really high burden of sub clinical strokes. But, they are sort of an indicator of other things that are amiss.

**Q:** Sure. This is an extension of this question, but I'm actually going to read a laundry list of just potential things, conditions or symptoms. And the question is do you think silent strokes place your patients at a risk for any of the following. So you can just say yes or no or give some qualified answer if you want to. But, no pressure to do any of those in particular. So again, the question is silent strokes place your patients at risk for any of the following. So first one is stroke?

**A:** Yes, I guess. Or indicates a higher risk of stroke.

**Q:** Okay. Intracerebral hemorrhage?

**A:** Depends.

**Q:** Okay. Heart attack or myocardial infarction?

**A:** Could indicate a higher risk of that.

**Q:** Okay. Heart failure?

**A:** No.

**Q:** High blood pressure?

**A:** Could indicate a higher risk of that. Is that like what you mean when you say--

**Q:** You can interpret however you want.

**A:** All right, I'll keep giving my qualified answers.

**Q:** That's fine. High cholesterol?

**A:** Could indicate that.

**Q:** Okay. Diabetes?

**A:** Could be related.

**Q:** Okay. Headache?

**A:** No.

**Q:** Vertigo?

**A:** Probably not.

**Q:** Seizures?

**A:** Depending on the quantity and characteristics of the sub clinical infarcts.

**Q:** Okay. Falls?

**A:** Depending.

**Q:** Memory loss?

**A:** Yeah, yes.

**Q:** Dementia?

**A:** Yes.

**Q:** Anxiety?

**A:** No.

**Q:** Depression?

**A:** Maybe.

**Q:** And schizophrenia?

**A:** No.

**Q:** Okay. They're not like all right answers or wrong answers to this.

**A:** No, it’s interesting. I've never really thought about this, so.

**Q:** Sure. So the next question is how comfortable do you feel treating patients with silent strokes?

**A:** Moderately.

**Q:** Okay. An extension of that question is do you follow any practice guidelines? And if so, which ones?

**A:** Not currently, just because I feel like the few situations I've encountered have all been very different. So I've sort of tackled them on a case by case basis. And if I've been uncomfortable it’s more like I've asked one of my colleagues in stroke for advice on what they would do.

**Q:** Okay. What do you think are the major knowledge gaps in this area?

**A:** I think the part of it is that guidelines for stroke management are sort of evolving constantly. And so, part of it is just having access to the most up to date information in a very obvious location.

**Q:** Sure. What would you want to know more about this particular issue, people with silent strokes?

**A:** I mean, I think that what would be the most helpful if there was just like some kind of more formulaic guideline for what are some initial steps that are appropriate for the physician to take on their own without any additional input. So like for X type of sub clinical stroke, just make sure they're on an anti-platelet agent, LDL below whatever, and then in what circumstances it’s worth a referral to a specialist. Because I also appreciate that you guys are busy and I don’t want to overburden with, like, you know, with referrals, basically.

**Q:** Okay. So this next question is kind of a thought experiment and it’s coming towards the end of the interview. So, let’s imagine that there's a rigorous observational comparative effectiveness study that's performed and the purpose of the study is to look at effective prevention therapies following silent stroke. So basically, preventing things that might be bad things that would occur as a result of silent stroke.

And let’s say that the study finds that what you might normally do, let's say it’s starting an aspirin, or telling people to go back on their aspirin, what if it’s found like that that actually is increasing harm rather than providing much benefit? So maybe the bleeding risks outweigh the benefits of stopping further stroke? So would this type of study, like a big, sort of observational, well designed study, a comparative effectiveness study, be enough to convince you to change your practice?

**A:** I mean, I think it would depend. You said it was a fairly large--

**Q:** Let’s say it’s a large, well-designed study. It is an observational study. And it’s an effectiveness study rather than an efficacy study. So it’s sort of looking at all comers and taking into account a lot of things like adherence to treatments and other variable patient factors.

**A:** Right. Hmm. That's a good question. I don't think so.

**Q:** Okay, that's fine. If not, what would you need to convince you to change what you're doing right now?

**A:** I mean, I think that the data in support of anti-platelet therapy has been based on, like, randomized controlled trials that have been pretty rigorous, so I would probably want some kind of randomized controlled study, not just like an observational study. I would want to know a little bit more about the patient populations that were examined, if it was, like, predominantly elderly people who maybe have like a higher risk of bleeding in general. I don't know, I think I would want some kind of control in place, yeah.

**Q:** Sure. On that topic, or that idea of a randomized controlled trial potentially guiding your practice, I'm thinking a little bit about this scenario of people with sort of incidentally found abnormality on a scan that might not be tied to clinical symptoms or how they present. If such an RCT were performed, how do you think patients should be recruited into this type of study?

**A:** I mean, it’s a good question, actually, now that I'm thinking about it. It's like if we say that the standard of care is to treat people with an anti-platelet agent, is that okay to actually not start them on an anti-platelet agent? But I guess we're--

**Q:** You're wondering about equipoise, whether or not there's equipoise?

**A:** Well, yeah, because at this point we're talking more about somebody who’s had a known infarct. We start them on anti-platelet therapy because that prevents-- that helps prevent further infarcts, but an incidentally discovered one, is that treated the same and are we actually-- is that reasonable for us to not treat them with anti-platelet therapy? How would they be recruited? I mean, I think-- sorry, I'm having trouble with this one.

**Q:** No, this is a difficult question.

**A:** I think if it’s felt reasonable that we should design this trial, based on like the questions I just brought up, I think I would probably just recruit patients where I incidentally found a stroke and said, “Your imaging shows that you have-- that in the past, it looks as though you may have had a small stroke that you were not aware of. We are trying to understand whether anti-platelet agents like aspirin are a safe and effective treatment to help prevent further strokes.”

Are you asking about recruitment, like how I would--?

**Q:** Yes.

**A:** Yeah, and so I would-- yeah, I’d probably like for a randomized trial, like randomize them to get either aspirin or placebo and that would probably be-- yeah. And then I guess you just-- you're not asking about the design of the study? Okay.

**Q:** No, just that initial phase, the idea of bringing people into study, like how would you approach them or who are the people you'd approach?

**A:** Yeah. I mean, I think it would have to be patients who-- in whom there was not a suspicion of a stroke. They had a brain scan to look for something else, incidentally found stroke from which they deny any symptoms that would refer to that infarct and then just asking them if they'd be willing to participate in a trial.

**Q:** Makes sense. Let’s say that trial, or some other study, is able to find a good, effective treatment and we're able to prevent other complications that might follow silent strokes. Let’s say more strokes, for example. So, this is actually a-- let's say this is a fairly common issue. Like, what I'm going to quote to you as a statistic is for people above age 50, the prevalence of silent stroke is about 20 percent.

**A:** Okay. That's true?

**Q:** It is.

**A:** Okay.

**Q:** Based on the best evidence that we have so far, large cohort studies in many places around the world.

**A:** And this is beyond, like, small vessel ischemic disease changes, or does it include that?

**Q:** These are using a pretty rigorous imaging definition where the radiologists are convinced this is a-- this would be called an infarct, even-- well, if somebody came in with the symptoms that would be the same thing, if they had symptoms that are referable to that part of the brain. They would still say that it’s an infarct. So, you use the same sort of criteria.

And so how-- this is kind of a question thinking about your practice, but also thinking about the whole country or the world, but how would you think it’s-- how do you approach the patients who already have these in that now we have a treatment for this, 20 percent of people over age 50 have a silent stroke.

**A:** I see. So is the question like should we be screening for silent strokes and starting people on treatment?

**Q:** Sure. How would you approach it in your practice, how do you think things should be approached on a larger scale?

**A:** Yeah. I mean, I think that's a tricky question. I think that, like, I would really need to be convinced about the risk/benefit ratio before subjecting people to random MRIs or to screening MRIs because, A, that's very expensive. And B, you're liable to discover other incidental findings and that gets back to your question of, like, silent strokes causing harm, which actually maybe we can come back to it because I was just thinking about it while you were talking before, sorry. [laughter] But, yeah, I think that while I would certainly be liable to start somebody on treatment where I incidentally discovered a stroke, I would not want to implement some kind of screening procedure, similar to how I feel about aneurysms. I think that's the best comparison I can come up with.

That I will do a CTA or an MRA if I feel like there's something that refers to that, and then I will refer patients on to, like, interventional for incidentally discovered aneurysm. But I'm like, crap, [laughter] and yeah, rather than subject-- I think that's-- yeah, I think I've given you basically my answer. I would certainly counsel people and start them on a treatment if it was felt appropriate in the event that I discovered one incidentally. But I wouldn't go looking for it.

**Q:** So that's it. That's actually the end of the interview. Now, I'll kind of talk to you a little bit for a minute to debrief after this. But I'm just wondering if you had any additional thoughts or questions you wanted to put out there before we close?

**A:** Yeah. So, I think the thing I was just thinking about, you asked about silent strokes causing harm. And I think that the two things I can think about, one is similar to incidentally discovered pulmonary nodules. It can sometimes subject people to increased imaging down the line which if it’s MRI it’s not as big of a deal. But I don't think these things are totally free from harm.

**Q:** Sure.

**A:** And then the other thing that I've seen in my specialty, people who get referred to me and they had some symptoms that were really not referable to a sub clinical infarct that was found on imaging, but they were told that they had a stroke and that this was the cause of their symptoms.

So, I had a patient who had a missed diagnosis of ALS for many months because her symptoms “started after she fell,” and then she was told that she had a stroke. And so it made it very difficult for her to accept the diagnosis. And I think that that was actually an example of that, doing harm. When, like, she had some vascular risk factors. She probably did have a sub clinical stroke at some point, but it was completely unrelated. So I think that's a good example of it causing more harm than good, to find those. Anything else?

Yeah. I mean, I think in general, I think of sub clinical infarcts as kind of like it gives you a little window into somebody’s vascular health or other potential medical conditions. So it can be a nice kind of sentinel, like maybe we need to do a little more digging to look for other vascular risk factors. And it can be a nice launching point for things like counseling on smoking cessation. Like, “Here you have this evidence that you had maybe a tiny stroke. We can prevent further strokes. You need to stop smoking.”

So it can be kind of nice in that way. Or, it can help you look for something like paroxysmal afib, which might not have been discovered, and can potentially be treated. So, yeah, I don't know. My views are evolving.

**Q:** Sounds good. Well, thank you very much. Let me just stop the recording right now.

END OF INTERVIEW

Vascular Neurologist 1

Study ID: VN1

**Q:** So we’ll get started with the interview with our first vascular neurologist. And just to let you know, there are about 10 questions but there's some side questions that I might add on, too, and depending on how the conversation goes. Feel free to answer with as much detail or lack of detail as you would like.

**A:** Okay.

**Q:** And so the topic is on covert or silent stroke. And so the first question is tell me what you know about silent or covert stroke so far, just in terms of your general knowledge, what you think about it?

**A:** I think they are much more than we think they are. And evidently, we find a lot more of them now than in the past with MRI techniques. And I don't know if you would consider clinically “TIAs” that have MRI findings of stroke as covert strokes. But I think that's a big component of what we see. Sometimes, patients have no symptoms whatsoever, you do an MRI, you find a stroke. And I think they're much more likely to happen in older patients and that's because they have other issues that sometimes-- they neglect the symptoms or don’t recognize they have the symptoms.

Or, the stroke itself is lost in a whole neuro degenerative process, vascular degenerative process. And sometimes, they can actually be heralds to worst things that can happen in the future. And if we find them and catch them in time, we could actually intervene earlier and prevent a disaster from happening. Now, whether we should screen people for that, that's another question.

**Q:** Fair enough. I'm standing kind of a little bit closer to make sure that--

**A:** Yes.

**Q:** We're [00:02:02]. So the follow-up to that is that this diagnosis has a number of different names that people have used which include silent stroke, silent brain infarction, silent cerebral infarction, covert stroke, covert brain infarction, sub clinical stroke, subtle stroke, asymptomatic stroke. Of these, which are the ones you've heard most often? Or of these, which have you heard used, rather?

**A:** It’s definitely silent stroke or silent infarction or sub clinical stroke. Covert is less frequently used, at least where I practice.

**Q:** Which do you use most often?

**A:** I would say probably silent, silent stroke or silent infarction, actually.

**Q:** Besides the ones that I mentioned, are there any other ones that you've heard used to describe this phenomenon?

**A:** Yes, sometimes we describe them more descriptively as strokes, present only in MRI or imaging only present strokes. And that's the only other authoritative etymology I've heard used in the literature.

**Q:** And among these, the ones that I mentioned, the ones that you mentioned, what would you say is the most appropriate or useful term, in your opinion?

**A:** That is an excellent question. I think-- actually, I think from a scientific perspective, the most accurate would be the latter one I used, which is strokes present noticed only on MRI or on imaging. And the reason I'm saying this is because sometimes there's recalled bias or misinterpretation of symptoms by the patients. They have had a stroke, but they never recognize it. And that's why they develop-- not necessarily silent, they were shouting at the patient, they never realized it was happening, especially posterior circulation stroke. So they have nausea, vomiting. They think they have a GI bug and you end up having a [00:04:04] medulized stroke. So I think the most accurate would be this, to say it’s present only on MRI without any clinical manifestations.

**Q:** Good. Let me just do an audio check to make sure that we're catching the volume.

**A:** Yeah.

**Q:** All right. So the next question is why do you think these covert strokes occur?

**A:** Why do they occur? [laughter] You mean, why they remain covert or why they happen in the first place?

**Q:** Both?

**A:** Well, why they happen in the first place, it depends on the underlying-- you know, epidemiology characteristics of the patient. So, my suspicion, I don’t have any evidence to back this up, is that many of them are perhaps cardio embolic in nature. But this is really not coming from any informed literature search. Now, why they sometimes are clinically silent? They can be one of two things, or a combination of two things, the first one being that they actually affect less eloquent areas of the brain, and therefore they do not necessarily cause symptoms.

And the second one is that they're small and, again, they don’t really, you know, cause a clinical death of this [00:05:30] by the patient. There's that.

**Q:** Okay, good. This is, in some ways, a related question. But from your perspective, in what ways are these silent or covert strokes the same or different in pathophysiology as compared to more typical strokes, those which present with neurologic symptoms or deficits?

**A:** To be honest, I don't think there's necessarily any difference. That's my understanding. I mean, a stroke is a stroke. It's probably a matter of luck. With the caveat that I have a suspicion that posterior circulation strokes may many times be misinterpreted by the patients, and therefore [00:06:14] bias I mentioned. And then I also suspect sub cortical strokes in, say, the right hemisphere. They may be less clinical manifest than others just because it’s a non-eloquent area of the brain. And therefore, they don’t really cause that many symptoms.

So, one could make an argument that some small sub cortical or [00:06:39] strokes, especially deep in the hemispheres, not so close to the basal ganglia or the thalami [?] or other areas may not manifest themselves. And I think we do see that many times. We see small [00:06:52] and we tell the patient they’ve had a stroke and they're like, “I never had any symptoms.”

And the other thing can be some cerebellar infarcts that never really cause any symptoms or anything like that. And those are more likely to be embolic in nature.

**Q:** What do you think are the major risk factors for covert or silent stroke?

**A:** Well, I think it’s the same risk factors as, you know, any other stroke; that is hypertension, is the most likely perpetrator, diabetes, hyperlipidemia, atrial fibrillation. Now coming back to the discussion we just had, if you were to pick up one of the risk factors, I would say hypertension and atrial fibrillation are probably the two more likely indicated factors there.

**Q:** Fair enough. During the times that you've seen this entity, what are the types of settings in which you've encountered the diagnosis?

**A:** That is a very good question, what is the type. I would say two. Usually it’s either at admissions for possible stroke or TIA, whatever we find, you know, infarcts in territories that don’t match the symptoms that the patient has reported. And in those cases, usually you have an embolic source that has caused some clinically manifested and some clinically non-manifested strokes. And oftentimes, you know, consultations, neurological consultations for other reasons that end up getting an MRI of the brain and we see strokes, chronic sub acute or even acute sometimes.

**Q:** Right. Just thinking off the top of your head, can you remember what those consults were for?

**A:** Oh, the most common one is, you know, the non-otherwise specified confusion or altered mental state. For whatever it’s worth, usually it ends up being a cephalopathy. But oftentimes, patients get imaging for that. So I would say that's by and large the most common.

**Q:** Good. For the patients-- maybe focusing a little bit on the patients that you saw in terms of your clinic or your inpatient service, what were the-- when a covert stroke is found, what were the symptoms or issues that they presented with? You mentioned some might present with a question stroke or TIA, but what are the types of symptoms that--

**A:** You know what? I don't think that I can really put a tag to this because it could be pretty much anything, you know. And as I said, many times as people have cardio embolism as a cause, which may cause, for instance, you know, seems [00:09:56] to the left hemisphere or left frontal lobe. And then you do an MRI and you find something in the right occipital lobe that doesn't really cause any symptoms. And nobody knew about it.

So I think, you know, you could have the opposite combination. You could have symptoms from the right occipital lobe and you find a stroke in the left frontal lobe. I guess I'm focusing a little bit on heat [?] stroke, but I'm sure you mean chronic sub acute or acute-- any [00:10:26] strokes?

**Q:** Any time point, yeah.

**A:** Yes, exactly. Yeah, so I don't think I can put a specific tag on this question.

**Q:** Okay, fair enough. So, one of the ideas around silent strokes or covert strokes or imaging defined strokes is that they're often encountered incidentally when doctors are investigating symptoms or medical problems that most of the time are not overtly thought to be related to stroke. So when you hear that term, what does it mean to you for something to be incidental in terms of how you respond to it or not respond to it, what it means, the significance?

**A:** Okay. Well, I guess it depends on at least a couple of things. First thing being what is the risk factor profile of the patient? And if they are otherwise high risk of stroke, should not be alerted and intervene, do something about it. That's definitely, you know, to gain a more prompt response on my side.

Now, the other thing is, you know, why was it asked in the first place why the patient have an MI in the first place, right? And for instance, I'm sure you've seen that yourself. People get MIs for dementia, cognitive decline, and we know that there's a link between vascular-- several vascular diseases and cognitive decline. And sometimes you see strokes. And you're like, you know what? Your father or your mother is so much worse lately because they just had a stroke. So sometimes they have an explanatory value.

But I think in my mind, stroke is stroke. So it does not matter to me whether it seems chronic or very acute. Obviously, an acute stroke probably carries a risk, high risk, of another stroke happening soon. But I would be sure that this patient is appropriately addressed in terms of secondary prevention. So it really doesn’t matter very much.

**Q:** If I'm hearing you correctly, it sounds like you do some sort of estimation or calculation of what you perceive to be that patient’s risk. And so regardless of whether-- if you're faced with something that's incidental, that prompts another question for you, which is assuming this is a stroke, how at risk are they for health [OVERLAPPING VOICES].

**A:** For another--

**Q:** Related to that, another--

**A:** For another stroke.

**Q:** For another stroke, okay. So, it sounds like if someone’s high risk you feel obligated to do something about that. If you estimate that they're low risk, would you also do something or would not feel obligated--

**A:** That would depend on how well I feel the secondary risk factors are being addressed or investigated. So for instance, so if I see someone that has a stroke that to me looks like it may be coming from carotid disease, I want to make sure that someone has looked into the carotids, you know. If they haven't, I'll either look myself or notify a primary care physician or vascular surgeon, whoever is involved in the patient’s care. And, of course, sometimes, you know, you see a stroke that seems incidental to you and the patient’s like, “Yeah, you know, I've been on aspirin and Plavix and what not.” Then you figure out the patient’s actually appropriately treated so you don’t need to do anything about it. But I would make sure that, you know, key questions have been answered about what the cause of the stroke was and if there's something to be addressed, it has been addressed appropriately.

**Q:** Fair enough. So, it sounds like probably at least in some of these scenarios in the past, you may have made a change in that patient’s management?

**A:** Yes.

**Q:** And so--

**A:** Absolutely.

**Q:** Have you in such an experience ended up starting a new treatment or modifying a treatment?

**A:** Yes.

**Q:** And if so, what sort of changes did you make?

**A:** I've certainly changed things such as just anti-platelet management. I start someone on anti-platelet or change them from anti-platelet to something else when I found something, i.e., atrial fibrillation, or on occasions I've found out that patient has guarded disease, which you know, ends up leading to a vacularization or something like that. So, this has happened several times in the past. I find something and you start digging in and realize there's something underlying it and you need to intervene.

Now, whether your question is whether I do something right away when I see a stroke or I initiate a cascade of events that leads to a change?

**Q:** I think that leads to my next question which is-- it sounds like you probably-- you're the type of person who might do additional testing or additional investigations if you had the suspicion that there's something awry. And you kind of alluded to these, but what are the types of tests that you would do?

**A:** So, yeah. I mean, first thing is you look at the stroke and having some knowledge about strokes, you kind of stratify patients as to regarding what's the possible etiology of the stroke. So if the stroke seems like it’s a lacunar infarct, I would not necessarily start looking for carotid disease or heart disease or stuff like that. I would focus on hypertension, diabetes and make sure they're well treated and they're taking their anti-platelet and they don’t smoke and stuff like that.

Now, if a stroke looks like it may be cardio embolic or artery to artery embolism, and then I would definitely make sure that the patient has some sort of vessel imaging. You know, being a vascular neurologist, I like to have the entire vasculature imaged, so a CT of the head and neck would be my go-to test. But at least the carotid ultrasound if that's in that territory. And/or cardiac evaluation with a transferacic [?] cardiogram and possibly a continuous heart monitor to see if there's some this atrial fibrillation.

The underlying, you know, thing being whether there is something that can be treated that would drastically change the chance of this patient having another event, basically.

**Q:** The next question might seem a little funny, but since we're the specialist as stroke neurologists, often looking into these issues, but would you enlist the help of any other specialists? Or have you enlisted the help of other specialists in this setting?

**A:** Yes, I have enlisted the help of vascular surgeons when it comes down to carotid interventions. And cardiologists, especially when I have high suspicions there might be atrial fibrillation. And I can find it, so either do a TEE with [00:17:32] studies, not for afib but for other unidentified cardio embolism. Or do an implantable monitor. And I've actually found a few patients with afib that was only found after a couple of months of monitoring. So definitely cardiology and vascular surgery are the two most common services I go to.

**Q:** Besides medical treatments, do you ask these patients, the covert or silent brain infarcts, to make any lifestyle changes or behavioral modifications in response to that finding?

**A:** Certainly. Certainly, you know, I'm a big proponent of exercise and diet, although to be honest with you, I'm a little pessimistic about how much people are willing to make these changes. Which I think have a very big effect on their chances of having another stroke. So, I always tell them to exercise and I usually am more specific about it. So walking, if they can do running. But usually, I tell people that they should be walking X amount of minutes per day, X amount of times per week. Sometimes people have very specific questions about what kind of exercise they can be doing, so we have to address that. And then diet is another thing. Weight loss. Commonsense things that people should be doing.

**Q:** I think you've already answered this question in several different ways, but the next question is do you think the covert strokes cause harm to the patients?

**A:** I think the cause in at least a couple of ways. One is that I think they probably contribute to, you know, a weakening or lessening of the so-called [00:19:20] reserve or, you know, brain reserve, which means that they make their brains susceptible to future injuries, strokes, accelerate dementia if dementia’s going to happen. So certainly I think although they are clinically silent when they happy, I think they have a cost, which is hidden. And I think that's the most important aspect of it.

And the second thing is obviously seizures. You know, especially if the stroke is close to the cortex, it results in gliocosis [?] and can-- I think it’s the most common cause of seizures in older individuals. So those are the two major things I think when I see someone with a silent stroke.

**Q:** Okay. You've described a couple of these additional health issues which have some greater risk, but thinking about the magnitude of risk, so from your perspective how much do covert strokes put patients at greater risk for these other health issues?

**A:** I can't say that I have a personal experience that's long enough and big enough to have formed a personal opinion. I don't think I have followed these people long enough anyways to see how they do. But my reading of the literature certainly tells me that there is a lot of vascular contribution to cognitive impairment and dementia. So I think those silent “strokes” do play a significant role in overall cognitive decline. So that I think unquestionably in my mind, they play an important role there.

I cannot say that I have seen that many people with, you know, seizures because they had a silent stroke. But I do know that theoretically it’s a risk and it sometimes happens. So I would definitely say cognition is the most important thing.

**Q:** I'm actually going to give you a list, that I'm not going to ask of everyone, in terms of what you think-- whether or not you think patients are more at risk of these because of having the silent strokes.

**A:** Okay.

**Q:** And you can just say yes or no, if you want to give a graded answer, you can. So, first is stroke?

**A:** Yes, certainly.

**Q:** Intracerebral hemorrhage?

**A:** Yes.

**Q:** Heart attack, or myocardial infarction?

**A:** Not directly.

**Q:** Heart failure?

**A:** Not directly, but they share many of the same risk factors.

**Q:** High blood pressure?

**A:** No, not directly.

**Q:** High cholesterol?

**A:** Again, not directly.

**Q:** Distribute?

**A:** Not directly.

**Q:** Headache?

**A:** Unlikely.

**Q:** Vertigo?

**A:** Yes.

**Q:** Seizures?

**A:** Yes.

**Q:** Falls?

**A:** Yes.

**Q:** Memory loss?

**A:** Yes.

**Q:** Dementia?

**A:** Absolutely.

**Q:** Anxiety?

**A:** Possible.

**Q:** Depression?

**A:** Sure.

**Q:** Schizophrenia?

**A:** No.

**Q:** Okay.

**A:** I hope I have understood the question and I'm not answering it in a very concrete way. But when you ask about hypertension, diabetes, I thought of the question as is the silent stroke a causative factor for those?

**Q:** Yes, that is the nature of the question. So you understood it correctly.

**A:** All right.

**Q:** For you, how comfortable do you feel treating patients with the silent strokes?

**A:** Well, that's what I do for a living so I feel fairly-- as comfortable as I feel with non-silent strokes. I think they're important, so I do treat them the same way in many ways. Lot are very [00:23:14].

**Q:** Do you follow any practice guidelines? And if so, which ones?

**A:** In general or regarding silent strokes? I can follow--

**Q:** Regarding silent strokes?

**A:** Yeah. I mean, I go to the American Heart Association primary, secondary prevention and other relevant guidelines for the most part. I want to think that I actually know the literature well enough to kind of know the granularity and the subtleties behind the guidelines, so I don’t necessarily take the guideline and follow it to the iota. But I do use them as a compass.

**Q:** Which ones do you apply for these patients with covert strokes, the primary or the secondary guidelines?

**A:** Excellent question. Secondary, for sure.

**Q:** Secondary?

**A:** Oh, I think they’ve had already a stroke so, you know, in essence you're looking at secondary prevention.

**Q:** Fair enough. What do you think from experiencing these patients and taking care of them and asking questions and thinking about things, what do you think are the major knowledge gaps in this area?

**A:** I think the biggest and foremost is the fact that people, because they never experience clinical systems, they do not think of it as the same-- as a clinical manifested stroke. So, stroke many times rings as a very loud bell for many people and it makes them at least take some things more seriously. And I find that a silent stroke may not be nearly as loud of a bell as a clinically manifested stroke. So I think that's as big knowledge gap for patients.

And I think when it comes to non-neurology, non-stroke physicians, I think there's also a misconception sometimes that because the stroke did not cause any symptoms or same [00:25:16] for TIA, actually, that it’s less risky than a clinical manifested stroke. And what I tell people is that the only difference is one gives you symptoms and the other doesn’t. Otherwise, they're the same thing and they should have been treated the same way.

**Q:** What would you want to know more about?

**A:** That is an excellent question. I think I would like to know at least a couple of things. One would be how frequent they actually are, because I don't think anybody knows that. I don't know that anybody can answer that unless we go to population cohort based studies. So that's one thing. A second thing is following the first point is if there's anything we can do to prevent them before they happen, and I guess there's nothing better than just trying to prevent cardiovascular risk factors from happening anyways.

And the third thing I would like to know is if there are any of them that never get to the attention of a vascular neurologist, they're found and neglected and ignored, and what's the impact of that neglect on the future outcome of these people?

**Q:** Fair enough. The next question’s sort of a thought experiment, okay? So let’s imagine that there's a rigorous observational comparative effectiveness study that's performed and it determines that there's some effective treatments for preventive therapies for covert stroke. Actually, specifically the covert stroke is found and these therapies are proven to be effective at preventing complications of covert stroke, such as, let’s say, ischemic stroke following that.

And let’s say the study-- it’s looking at the effectiveness and actually finds that treatments that you might normally start, whether they're, for example, antiplatelets, actually seem to be unfavorable in terms of the risk/benefit ratio where they're actually-- for example, you're starting all these patients on aspirin, perhaps, and you find that actually they're bleeding more than they're having subsequent strokes. So would that type of study be enough to change your practice, so like an observational comparative effectiveness study?

**A:** It would tentatively give me a pause and make me think a little bit more. I don't know that I have a one size fits all approach to these that would need to change one way or the other. But it would definitely give me pause and make me think why is this happening? Are these people different from other stroke patients in some way that we don’t know? So, really like it to be followed by something more. [laughter]

**Q:** Sure. What sort of things would you want it to be followed by? What type of--

**A:** So we really found this, you know, that if you put these on anti-platelet, instead of doing good you do nothing or you do harm, I want to know how are these people different than people with clinical manifest strokes and if I can find out why they don’t respond the same way to treatments that other patients supposedly respond to. So that would be one thing.

**Q:** So thinking down more of the pathophysiology understanding characteristics?

**A:** Yeah. You know, as you probably figured out in my mind, these people are probably the same. Maybe I'm mistaken, but I think of silent and clinical manifest strokes as the same thing, stroke. But so maybe the biggest, you know, surprise or the biggest change in conceptuals that would happen is to make me think maybe these people are not exactly the same.

Now, would I change my practice? I don't know. Perhaps I would be less enthusiastic about using anti-platelets so liberally in these people, for instance. But, I would certainly want to dig more into it for someone to do it, not me personally. So I don't know if that answers your question?

**Q:** It does, it does, yeah. I guess a follow-up to that is thinking about that type of study, like say a well designed observational study, prospective or retrospective, if that gave you some pause, would you be looking for a randomized controlled trial or meta analysis or something else to follow that to [00:30:23] on top of that data or would that type of information be sufficient to guide you?

**A:** Well, I guess it would depend on what would you find. You know, I think the essence of what you're asking me is, you know, whether you would find something to suggest that, you know, people with silent strokes are different than other people with strokes. And so, I would really want to-- it would depend partially on what the finding of the study is. So, you find a difference, what's the difference? And, you know, the follow-up study to that would depend on the kind of difference you find, I suppose.

So if you find some imaging marker, I guess you could-- there's a cohort of people with imaging available, you could go back and dig more in that direction. If there seems to be more of a medication effect, then you could definitely do something perspective, you know, randomized controlled. So it really depends on the kind of difference you find, in my mind, at least. I don't know if that answers the question.

**Q:** No, it does, yeah. If studies were done prospectively, in terms of the outcomes of the studies, what do you think would be most important to look at?

**A:** That's a very good question. Well, I think assuming that cognitive decline and cognitive impairment and/or current stroke are the two major things we're worried about here. I think those are the two most important end points in my mind. Now, whether there would be more feasible end points to measure, that's a different story. But I think conceptually, it would be the two things that one would want to look into, yeah.

**Q:** Okay. Anything else you could think of that comes to mind?

**A:** Seizure would be one, but I think seizure is going to be relatively infrequent. You would need a very big sample to do anything. So I think cognitive, you know, performance. I should be more specific about this. I mean, one could either find a very global cognitive performance test that kind of is over-inclusive. One could look into gaits, looking at falls, could be looking into, I guess, what kind of strokes again. Or just overall functional performance, something such as one to five ranking scale, you know, going down the road.

This would have to be kind of a longitudinal study, though. You would have to-- enroll people and follow them, follow them, follow them and see what happens.

**Q:** The next question’s a little bit dependent on your style of practice. But let's say we found that there are effective treatments that we should be getting to people with covert strokes or silent strokes and that actually prevents an important outcome. Let’s say it’s recurrent stroke. How do you handle all those patients that you've seen already, or how would you approach that, the people in your practice?

**A:** Would I go back and change something, you mean? Sure, I would have this new finding. I would want to insure that the finding actually applies to my patient. Because as I said, sometimes you define a very specific cause and, you know, for instance you find someone with [00:33:47] or fibrillation and I don't think there's much room for thought there. Maybe there will be. But I think it would, you know, depend to some degree on how applicable the study population would be to my individual patient.

Certainly for patients of mine that would fit your average study patient, I would sit down and talk to them and explain what's happening. And to some degree, would depend on how much they could understand and the value of the literature and findings and have a discussion. But that's what I would do.

**Q:** So we're coming pretty much to the end of the interview right now. But I was wondering if you have any additional thoughts or ideas, things that come to mind, questions that you would want to ask? Any additional thoughts?

**A:** No, to be honest with you, [laughter], as I said, in my mind, these people are-- or should be seen as the same, at least for the time being. I'd need to have a look into the literature if there's anything to suggest that they may be different. But, that would be a very interesting line of thought. But otherwise, I would just treat them one in the same. Conceptually, I would like to know why some people have symptoms whereas some others don’t. And just to follow up, you could keep almost a matched case control study where you have people with singular location and maybe kind of comparable size strokes. Some of them are clinically manifested, some others aren't. And this could probably, you know, bring up to the surface some difference that you're looking at and explain why you can have-- some people have silent and why some others are not silent. But anyhow, that's just a thought.

**Q:** So one final question I'm just going to add on is I think you've described several times that your belief and your impression is that these silent strokes are essentially the same as any other type of stroke. A stroke is a stroke. And so, you also mentioned that you'd be interested to know if there are patients out there who have these findings who never come to the attention of a vascular neurologist.

**A:** Right.

**Q:** So let's say I was to tell you that in the literature it’s described that, as an estimate across several large population-- or large cohorts in different parts of the world, that the average-- say the average prevalence of silent stroke is about 20 percent in people who are age 50 or older. And there's some proportion of people who are younger as well. How would you feel about that, knowing that you just mentioned-- you just kind of expressed a thought that maybe these patients should come to the attention of stroke neurologists [OVERLAPPING VOICES].

**A:** I have two questions. One is how is stroke defined in these studies? Is it any, you know, area of gliosis? I would want to know that.

**Q:** Right, how is it defined in terms of [OVERLAPPING VOICES].

**A:** Yeah. So is it overestimated, underestimated or what? But whatever it is, it sounds like there's a substantial number out there that do have silent strokes and they should be. Well, I guess the other question is do vascular neurologists make a change and see, you know, if patients who never see a vascular neurologist do differently in any other way than people that are actually taken care of by vascular neurologists? So we may not be making any difference at all. [laughter]

**Q:** But you seem to be making changes, right?

**A:** I do make changes, but not all of them. It kind of depends on the individual case. Some people, there is nothing that you can fix, and some others there's nothing specifically you can fix except for treat the blood pressure and cholesterol and diabetes. So, I guess it depends.

But yeah, going back to your question, 20 percent age 50 and older, I think that's a big number. And again I will go back again to cognitive impairment. I'm fairly convinced that vascular cognitive impairment is a big problem. And as we get older, it’s going to get only more and more important.

So, if there was one thing that I would really want to know is what's the burden of, you know, silent strokes, recurrent silent strokes and how they affect cognition. Because I think that's going to be a big, big problem going forward. I don't know if that answers your question.

**Q:** No, it does. I was just sort of asking that final question just to explore that idea.

**A:** No, I think your problem not is that in my mind vascular cognitive impairment and silent strokes are quite linked with each other. And the more you leave it untreated, the more it’s going to continue to happen. And some day, the patient’s going to be demented, you're going to [00:38:58]. [laughter]

**Q:** Thank you very much.

**A:** Absolutely.

**Q:** I'm just going to stop the recording right now.

END OF INTERVIEW

Vascular Neurologist 2

Study ID: VN2

**Q:** So this is the interview for vascular neurologist #2. There'll be ten questions and it might expand a little bit more in terms of some sub questions.

**A:** Okay.

**Q:** So the first question is what do you know about silent strokes or covert strokes, just in terms of your baseline knowledge?

**A:** So, I mean, we like to call them asymptomatic strokes. So we don’t know whether they are, you know-- I mean, it’s still a matter of debate whether they are truly silent or not. I mean, they may have cognitive issues or there are other issues. It all depends on how deeply you look into it. There are studies which show that these people are at a high risk of subsequent strokes, and there is data from-- data all the way going back to massive trials and stuff which have shown-- so, I think they have to be aggressively managed, as you'd manage any other stroke patient.

**Q:** So, I think you alluded to something in terms of the naming of these. So the diagnosis could potentially have several names, which include silent stroke, silent brain infarction, silent cerebral infarction, covert stroke, covert brain infarction, sub clinical stroke, subtle stroke, asymptomatic stroke. So, which of these are terms that you've heard or which ones you think are used most often?

**A:** I mean, silent and asymptomatic I think are the ones that probably are used more often. That's what I think, yeah.

**Q:** Are there other terms besides the ones that I mentioned or you mentioned that you've heard used?

**A:** So, the other thing that you can add to it is hemorrhages. I mean, there are certain hemorrhages that are silent, too. I mean, those are asymptomatic with [00:01:56] yes.

**Q:** Okay. And of these, what do you think is the best term, or the most useful term or appropriate term?

**A:** I would probably favor asymptomatic infarct-- asymptomatic strokes, uh-huh.

**Q:** Okay. So why do you think these occur?

**A:** Oh, there can be a different variety of reasons. I mean, just like you can have reasons-- you know, there are different reasons for any strokes. I think this can have petty much a fairly wide variety of reasons. But you can see these strokes in patients with atrial fibrillation, you see this in patients with BFOs, you see this in large arterial disease. You see this in cryptogenic, you know. So I don't think if the question is is there a particular etiology for these silent strokes, or a group of etiologies different, or is this a different subset of stroke patients in terms of etiologies, I don't think I know the answer. I would say no.

**Q:** It sounds like you think at least some of them probably have the same pathophysiology or mechanisms?

**A:** Right, yes.

**Q:** Why do you think they present without symptoms, then?

**A:** It depends on-- you know, it depends if the stroke involves an eloquent cortex, so a region of the brain which is much more likely to produce symptoms. It also depends on how big these strokes are. So the size and the location.

**Q:** Okay. This is sort of a similar question, asked in a slightly different way. But, from your perspective, in what ways are covert strokes or silent strokes the same or different in terms of the pathophysiology as more typical strokes? So I think you described in terms of differences, maybe the part of the brain that's affected and also the--

**A:** If I were to guess, I mean I would just say that typically these are smaller so you can make a case that, well, you know, cardio embolic strokes such as from atrial fibrillation tend to be larger strokes. So you say maybe they are less likely to be cardio embolic strokes. But, I'm not sure if that really holds out. That's a guess.

**Q:** All right, fair enough. So it seems like size and the part of the brain are kind of what you're alluded to as being differences. And then similarities might be the mechanisms by which they [OVERLAPPING VOICES].

**A:** Yeah, yeah, right. Yeah, yeah.

**Q:** Okay. What do you think are the major risk factors for these--

**A:** And if I can add one more thing, I think in elderly patients, they are much more likely to escape attention.

**Q:** Why is that?

**A:** I think it’s probably because the symptoms may be masked by other things. They may have cognitive deficits and things. They may not be reported, or less likely to be recognized.

**Q:** Sure. So the patients themselves--

**A:** Yeah.

**Q:** --might just be a vulnerable population in terms of their ability to recognize or report the symptoms?

**A:** Yeah, right. Or, to be picked up by a physician, uh-huh.

**Q:** Okay. The next question is what would you identify as being the major risk factors for silent strokes or covert strokes?

**A:** The risk factors are the same. The major risk factors, so if you look at overall the general risk factors are the same; hypertension, diabetes, heart disease, atrial fibrillation, hypercholesterolemia. And then you have the [00:06:02] opathies. Then more proximate causes would be, you know, large vessel disease, you've got-- we talked about atrial fibrillation, yeah. Smoking. Yeah, uh-huh.

**Q:** Okay. In what settings have you encountered this phenomenon of silent strokes?

**A:** So, usually-- I mean, well, you see it both in inpatient and outpatient. I mean, in the inpatient side, it’s not unusual to see patients who come in with a stroke and who've had another stroke which went unreported. You know, I'd characterize the first one as being asymptomatic. But in outpatient settings, sometimes you get a brain scan or a patient undergoes a scan for something totally unrelated and a stroke is picked up.

**Q:** In either setting, so I think for the first setting, you're describing people who've come in with stroke symptoms, presumably, and then you find there's some other brain condition.

**A:** Yeah, right.

**Q:** And so they're essentially presenting with stroke symptoms, but there's another one that doesn't explain the symptoms?

**A:** Right, uh-huh.

**Q:** In the second setting where they're getting scanned for something else, what are some of the symptoms that patients might present with?

**A:** So, for example, headaches. I mean, they get scanned for headaches and they're found to have an infarct. Dizziness. So, and some of these are, you know, routine stuff that you may not yourself initiate the investigation, but people come up and they get referred. So that is what I would-- that's basically what comes to mind. I mean, I have seen things for seizures and stuff. I mean, it will be a debate whether this will be an asymptomatic stroke. But the initial stroke didn't produce any focal deficits, and they developed epilepsy which is secondary to the lesions. So I've seen patients like that, too, yeah, uh-huh.

**Q:** So that's certainly a special scenario in terms of if people develop seizures and then you find a lesion and, like, then like beyond that?

**A:** Right, right.

**Q:** For some of these other things, like you mentioned headache and dizziness, when you encounter these patients, do you end up connecting those initial symptoms and this finding on the scan?

**A:** So, yes. You try to correlate the-- from where the location is given the presentation, whether this was truly incidental or it was related to the presentation, yes.

**Q:** Okay. So you've brought up this term incidental. And so the next question is actually related to this, which is these strokes are often discovered incidentally when physicians are looking or investigating a symptom or medical problem that might not overtly seem to be related to stroke. And so what does it mean to you for something to be incidental?

**A:** So incidental meaning it was recognized-- so the investigation was not designed to pick up-- or the suspicion was less for a stroke. Or there was no suspicion for a stroke. The initial-- the reason for initiating the investigation was something totally unrelated. You picked up a stroke and that is what I mean by incidental. Right, yeah.

**Q:** And when you encounter that, do you feel like you should do something about it? Do you feel obligated to respond?

**A:** Sure, absolutely, uh-huh.

**Q:** So my next three questions are going to be related to that. So for example, let’s say medications or treatments. what types of things do you do? Do you change treatments, do you add new treatments?

**A:** So, yeah, so you would certainly consider, you know, for example, anti-thrombotic or anticoagulant treatment depending again on the risk factors. So some of it will be guided by you will initiate a workup and see, you know, what other things that you discovered along the way. So if somebody has a carotid lesion, you may want to operate on them.

**Q:** You're sort of alluding to some of this already, but what sort of testing would-- might you pursue in this setting?

**A:** So definitely vascular imaging, intracranial-- and depending on, you know, the location of the stroke. So vascular imaging, for sure, intracranial, extra cranial, you want to have a cardiac echo, you want to have some prolonged monitoring of the heart rhythm even with the halter or king of hearts, or something like that. That's an option, yeah.

**Q:** So this is a kind of funny question to ask from me to you, sort of a stroke specialist, but since we're often investigating this issue and the ones that people look to to try to figure out how to do that, but in your investigation for these covert strokes, do you enlist the help of other specialists?

**A:** Again, you may want to do it. I mean, for example if there's a young patient who comes in and you're suspecting a coagulopathy or pro-thrombotic disorder, you may want to get a hematologist on board. The other tests are fairly routine. So for example, if you get an echo, it’s then interpreted by a cardiologist. So I would say that, you know, that is-- and similarly, a radiologist if he’s reading your carotid ultrasound, you're still relying on somebody else’s expertise, uh-huh.

**Q:** When you encounter these patients, do you ask them to do anything differently in terms of their lifestyle or any-- make any behavioral modifications?

**A:** Sure. So, I do the same thing that I would advise any other stroke patient. So smoking cessation, watching diet, increasing fiscal activities. You know, medication compliance. Yeah, uh-huh.

**Q:** For these covert strokes, do you think they’ve actually caused some harm to the patients?

**A:** So, that is a matter o debate. Some people would argue that any stroke is bad. I mean, just because you're not picking up signs and symptoms in your office visit doesn't mean that it doesn't have any consequences for the patient. So, I think one, it puts them at a higher risk category. Secondly, we sometimes don’t know whether it has subtle cognitive deficits. And if these lesions accrue down the line, what it would mean.

**Q:** In your perspective, what are the things that-- well, I guess I'll put this-- this is kind of a two-part question. One is what are the things that you think covert strokes or silent strokes put people at risk for? And how much risk do you think is that magnitude?

**A:** So the magnitude of-- well, so, one, they are at a higher risk. I think the magnitude of risk varies, again, depending on the underlying etiology. So if you've got symptomatic carotid and you don’t intervene and I think the magnitude of risk is higher. And similarly, if you pick up atrial [00:13:50] for atrial fibrillation that you didn't recognize before, you don’t put these people on anticoagulation. Obviously, the magnitude of risk is higher, again like, you know, it depends on the other underlying factors.

So, you know, for afib, if somebody has congestive heart failure on top of it, so the risk stratification would vary depending on the other things that go along with it.

**Q:** And so certainly, you feel like stroke is one of the-- recurrent stroke or--

**A:** Yeah, so recurrent stroke, yes, yes.

**Q:** Is the big risk here?

**A:** Yeah, yeah.

**Q:** Anything else that comes to--

**A:** So, not only-- and these patients are at risk of other ischemic events, coronary artery disease, peripheral vascular disease, especially if you've got large vessel disease in the carotids, more likely to have coronary artery-- coexistent coronary artery disease or peripheral vascular disease. So, yes, uh-huh.

**Q:** Okay. I'm going to actually for the next set of questions, it’s stemming from this, but I'm actually just going to run down a list of things and the question is do you think silent strokes puts your patients at risk for these things? And you can say yes, no, or give some sort of graded answer.

**A:** All right.

**Q:** And it’s not yes or no-- the answer is not yes for everything or no for everything.

**A:** All right, okay.

**Q:** So the first one is stroke?

**A:** So, what, are they risk for stroke? Yes, uh-huh.

**Q:** Intracerebral hemorrhage?

**A:** Yes, uh-huh.

**Q:** Myocardial infarction?

**A:** Yes.

**Q:** Heart failure?

**A:** Well, it depends. [laughter]

**Q:** Okay. High blood pressure?

**A:** So, they may have preexisting hypertension. The stroke is not causing the hypertension, but they may have preexisting hypertension.

**Q:** High cholesterol?

**A:** Again, the same thing. They may have preexisting hypercholesterolemia.

**Q:** Diabetes?

**A:** Same thing, they may have preexisting, uh-huh.

**Q:** Headache?

**A:** Some data to support, not very good that people with headaches have a higher incidence of asymptomatic infarcts.

**Q:** Okay. Vertigo?

**A:** They are at risk for vertigo. Not sure.

**Q:** Seizures?

**A:** Yes.

**Q:** Falls?

**A:** I'd say so, yes.

**Q:** Memory loss?

**A:** Yes.

**Q:** Dementia?

**A:** Yes.

**Q:** Anxiety?

**A:** Don’t know.

**Q:** Depression?

**A:** Not sure.

**Q:** Schizophrenia?

**A:** Probably not.

**Q:** So the next question is how comfortable do you feel treating patients with silent strokes or covert strokes?

**A:** I mean, that's what I do, I treat stroke patients, symptomatic or asymptomatic, I treat them.

**Q:** So you feel pretty comfortable?

**A:** Yeah, right.

**Q:** Okay. Do you follow any particular practice guidelines? And if so, which ones?

**A:** So, I mean, I follow the guidelines with the usual stroke guidelines like you treat any other stroke patients. So, in terms of risk factor modification, in terms of choosing antithrombotic, antiplatelets, anticoagulants.

**Q:** So just to clarify, do you follow the secondary stroke prevention guidelines or the primary prevention guidelines?

**A:** Secondary stroke prevention, yes, uh-huh.

**Q:** What do you think are the major knowledge gaps in this area?

**A:** One, I think one of the things is that it’s not very well recognized that these patients are at risk of harm. I mean, they are at risk of, one, major-- you know, medical events from strokes to the other things that we talked about. That there is an urgency to treat them just like you'd treat any other patients in terms of investigating their underlying risk factors for stroke and for-- that they need to be treated just like any other stroke patients in terms of secondary prophylaxis.

**Q:** So you mentioned risk of harm, people knowing that and also the urgency, which is interesting. What do you mean by that, like just thinking a little bit about that urgency issue. Let’s say somebody has been scanned or something--

**A:** So for example, if you pick up somebody-- which looks like a sub acute stroke, asymptomatic, acute or sub acute, I would treat it urgently. I mean, there is-- so you do the vascular imaging and like you would investigate any other stroke patient because the risk of recurrence is probably higher in the initial few days, weeks after the stroke.

**Q:** How about for the more chronic ones?

**A:** Well, it’s debatable whether the same kind of risk exists but I think overall these patients are still at a high risk of stroke recurrence.

**Q:** Okay. What are things that you would want to know more about, or learn more about in this area? Like, if you can make a wish list of studies to be done, or sort of knowledge to be prioritized, what are the things you'd want to know more about to kind of practice?

**A:** So one of the things that we don’t know, really, is there are not very good prospective studies that I'm aware of which has looked at these patients. And so, lot of things which I'm saying is based on my clinical experience. But we don’t know necessarily whether all these things that I've said were true in terms of-- so we would anticipate them to have, you know, to have worst clinical outcomes from a neurological standpoint, from a systemic standpoint, down the line if they're not managed appropriately. We don’t know whether it’s true.

Secondly, you don’t know what kind of neurological problems that they’ll-- to what is the incidence of recurrent seizures-- recurrent strokes, incidence of seizures in these patients, dementia and stuff, all those things that-- because it also would guide treatment priorities and how you counsel patients and caregivers. So I think it’s a problem.

**Q:** Okay. From your view, because you mentioned the phrase appropriate treatment, sort of distill down, what do you think that means for these patients? What would you consider the appropriate pathway or the appropriate management of these patients?

**A:** So appropriate treatment would be whether these patients need to be treated the way you treat any other stroke patients in terms of vascular-- I mean, you think that that is the right thing to do, but it has implications for how you utilize the resources, for example. I mean, if you see somebody, do you need to admit them, how urgently and what kind of scans you need, what kind of investigations you need to pursue, or whether-- so a lot of those things are unknown.

**Q:** So the next question’s a little bit of a thought experiment. So, let’s say there was a study that was being performed. It's observational, it’s a rigorous, well designed study and it’s looking at comparative effectiveness of certain medications following the discovery of a silent or covert stroke with the intent to prevent long-term complications, let’s say recurrent stroke. And let’s say that, I mean, for example, you might be thinking of putting somebody on an antithrombotic medication, whether it’s anti-platelets or anticoagulants. And this study found that the opposite of what you would normally do might actually be the better option.

**A:** So not to put them?

**Q:** Exactly. Let's say that the bleeding complications outweighs the benefits that might come from the antithrombotic. Would that be enough to sort of guide your management of these patients or sort of alter practice?

**A:** Sure. I think that would be-- I mean, that would be helpful. I think that is really unlikely. [laughter] But, yeah, that may help, uh-huh.

**Q:** Why do you think it’s unlikely?

**A:** Well, it’s unlikely because there's been a lot of-- I mean, there's been several studies which have looked, I mean, at use of antiplatelets in patients who've not had strokes for primary prevention. I mean, we don’t think that there is significant harm in exposing these patients to antithrombotics. Now, if you've had an infarct, you would assume that there is definitely going to be some good.

**Q:** Do you think in terms of thinking about types of studies, in order to guide your practice, you would need a randomized controlled trial to look at what's the right way of preventing things after stroke or would this type of study strictly more pragmatic be enough?

**A:** So the question is going to be whether there is clinical equipoise or a clinical trial and that is the big debate. That is going to be the hardest thing to say. I mean, a lot of people would say, like me, would say no, these people need to be on an aspirin, or something, if you've had a stroke. I'm not yet convinced that, you know, these people are at a high risk of hemorrhage. So, unless there's some studies which-- before, as a prelude to a clinical trial, shows that okay, the risk of hemorrhage is high in these patients, I probably won't be persuaded to enroll any of my patients in such clinical trials. [laughter]

**Q:** Fair enough. What do you think are the-- if future studies are done, prospective, because you mentioned that prospective studies are something that would be important, what are the outcomes that would be really important to look for?

**A:** So, I mean, the stroke is going to be an outcome for sure and what you're looking at. And you can use stroke as a homogenous group. You can look at all strokes. You can look at major, you know, cardiovascular end points, MIs, you can look at-- actually, just to step back. So you can look at stroke, you can look at cognitive outcomes on how these people do, whether they develop vascular dementia and stuff like that down the line. You can look at other systemic cardiovascular end points and those would be the main things.

**Q:** Okay, fair enough. And the next follow-up question--

**A:** So if I may just step back and say one thing.

**Q:** Of course, sure.

**A:** While I was thinking, I would say that I would be curious to know how practices change not just in terms of antithrombotics, but how aggressive you become about controlling for example, hypertension or cholesterol in these patients. I mean, so there are several layers to how you manage these patients.

**Q:** Absolutely.

**A:** And whether, I mean, doing a trial and looking at these-- not so much antithrombotics, but how do you treat them as secondary prevention or as just anybody with primary prevention? And I think that would also be an interesting question to look at.

**Q:** So as a stroke neurologist, and let’s say I was going to do such a study, so you mentioned anti-platelets, sort of aggressiveness so antihypertensive treatment and anti cholesterol treatment. Is there anything else that you would want me to look at?

**A:** Smoking cessation. I think if people are told-- I mean, the people I think if they are told that you've had a stroke, they're more-- they would be much more motivated to quit smoking, for example, lifestyle changes, than just say that your scan shows something. I think it may be an important tool to modify behavior, not just for patients-- not just for physicians, but for patients.

**Q:** Fair enough. The last follow-up is related-- it actually is dependent on how you arrange your practice, especially the outpatient practice. But let’s say there were studies that were done and there's evidence for particular practices that are very effective in preventing stroke after covert or silent stroke, or whatever outcomes end up being important. How would you-- what would you do about all those patients that you've already seen? Or like how do you approach that, that have these covert strokes in the past, are already known to have one?

**A:** Well, it’s difficult. So, if, one, as a specialist, you're not always in touch with these patients. Sometimes you see them and sometimes you don’t. I mean, the assumption is that once you publish a study like this, the physicians who are actually in charge of these patients would make the necessary, you know, or they would refer these patients back to you. But, if there are patients who are still under my car, I would certainly adopt the results from the studies to change practice.

**Q:** Another way of framing the question, thinking on a broader level, with you as a stroke neurologist and leader in the field, there are sentiments amongst the large primarily prospective cohort studies related to silent stroke or covert stroke that above age 50, in adults, the prevalence is probably about 20 percent age 50 and up. And there's, of course, some in younger adults, too. Probably a smaller proportion. It seems to rise with age.

So thinking about that big of prevalence, 20 percent of adults over age 50, how do you think that should be handled for all those people out there walking around with silent strokes?

**A:** So I think they need to be investigated, I mean the reasons-- so, one of the difficulties in research like this is that you don’t know-- so this is not-- in prospective studies, so you're talking about studies where people had a scan, like Framingham study prospective--

**Q:** For example, like the--

**A:** --everybody underwent a scan?

**Q:** Exactly.

**A:** Right. So these are not referred by the physicians for --

**Q:** No, they were enrolled in the cohort and scanned at pre-specified times.

**A:** So I think it tells you that this is a major area that still needs work. I think it tells you that it’s-- I think the morbidity from this kind of lesions is going to be higher as-- and you would assume that these lesions would accrue and these people are more likely to suffer, you know, cognitive impairments and things like that. So I think secondary prevention is really important in these people.

**Q:** Okay, I think that's very helpful. So, we're coming to the end right now and I just wanted to sort of open things up to see if you have any additional thoughts or questions that you would have in mind about this topic?

**A:** So the one thing that will come up with-- with a study like this, if you're proposing, is the question about screening. So should anybody over 50 have an MRI? Would you screen just like you screen for prostate cancer or would you just do the other thing, meaning that you just want to make sure that they're simple things that blood pressure is under control, their sugar is under control? So that is the big question. I mean, it has a lot of implications for using resources. If you're going to-- and you'd say, well, CT scans are not good enough. These patients need MRIs.

**Q:** Great. Well, thank you so much for your time. I'm just going to stop the recording right now.

**A:** And on record-- [laughter] you owe me a beer, right?

**Q:** Yes, I owe you a beer. [laughter]

END OF INTERVIEW

Internist 2

Study ID: IN2

**Q:** So there'll be about ten questions and depending on how you answer, I might expand to a couple of side questions.

**A:** Okay.

**Q:** The first question is just for you to tell me what you know about silent about silent or covert stroke so far?

**A:** Well, I guess what I don't know is so certainly I would imagine a silent stroke would be if there's an area on imaging that clearly looks like it’s been infarcted, but the patient doesn’t recall ever having a stroke on exam, nothing is detectable. Or, I suppose maybe it’s detectable, but no one had noticed it before.

What I don't know if it counts as a covert stroke, and I always assume not was the chronic white matter changes that people have in older age. That I don’t consider a silent stroke, but rather like an area that has encephalomalacia. So that, I guess, would be a definition. And other than that, I have no idea how common they are, if there's an area. I assume they happen to older people with stroke risk factors.

**Q:** Okay. And you certainly don’t have to have any background knowledge on this topic. This is completely exploratory just to get a sense for what you know, what you don’t know. So, you can comment on what you don’t know as well. And yeah, it’s a little bit of a gray zone in terms of what people consider stroke and what people consider white matter disease. Or if that's even on the same spectrum of disease, or if they're completely separate entities. There's a lot of debate about that.

Thinking about just strokes, and I guess we’ll focus more on discreet brain lesions on an MRI or CT, in patients who don’t have clinical symptoms or deficits that at least they were able to recognize, the diagnosis at least in the literature, has a number of different names that people have used including silent stroke, silent brain infarction, silent cerebral infarction, covert stroke, covert brain infarction, sub clinical stroke, subtle stroke, asymptomatic stroke. Of these, which are terms that you've heard used?

**A:** I guess maybe sub clinical stroke. Maybe I'm confusing it with other sub clinical-- you refer to several things as sub clinical, that sounds familiar. Maybe asymptomatic stroke.

**Q:** Have you used any of these terms in describing them to colleagues or patients?

**A:** I don't think so. No particular time stands out in my memory.

**Q:** And are there other terms besides these ones that we've mentioned that you've heard used referring to this phenomenon?

**A:** No.

**Q:** Of these, do you think that because there's a whole plethora of different terms, hopefully we’d want to choose just one so we're all speaking the same language. Is there one that you would think is most appropriate or most useful?

**A:** Yeah, I'm just thinking.

**Q:** And it doesn't have to be one on this sheet of paper.

**A:** Yeah. I think one that uses stroke is probably the most user friendly, whether silent, sub clinical or asymptomatic stroke, those would be my top three. Subtle stroke suggests someone has deficits that are subtle. Maybe silent stroke nicely echoes like a silent MI, which we certainly talk about. Or asymptomatic stroke. Do you want me to pick one?

**Q:** No, that's okay. Yeah, there's no-- there's no requirement that there has to be just one. It was mostly to get a sense for what term seemed reasonable in terms of encompassing what you would put into this category. For these silent strokes, why do you think they occur?

**A:** Presumably the same reason clinical strokes occur. So, I'd imagine more of them, that they're probably smaller areas such that they're sub clinical that more are due to the sort of cardiovascular risk factors; hypertension, hyperlipidemia, than afib, which I would-- might think of, like, affecting a larger area, although I'm not sure. So, I guess that would be like thrombotic disease rather than embolic. That would be my guess for why they occur.

**Q:** Okay. This is sort of--

**A:** So that's what I get.

**Q:** Sure, yeah. This is kind of an extension of that question, but from your perspective, are these silent strokes the same or different from regular garden variety strokes that have symptoms or deficits in terms of their pathophysiology or how they occur?

**A:** In what way? This is all my speculation, so I would imagine that they are the same. But like I said, maybe skewed towards the thrombotic rather than embolic. Does that answer your question?

**Q:** Yeah. No, it does.

**A:** Okay.

**Q:** Yes. And again, this is kind of like a follow-up question for that. I think you've already alluded to a few of them, but what do you think are the major risk factors for these silent strokes?

**A:** Well, I guess I didn't say smoking, but certainly smoking. Hypertension, big one, hyperlipidemia, and probably afib. Maybe even heart failure. What am I missing? Peripheral vascular disease which just goes along with all those other things. So, and then I suppose, I mean, people with hyper coagulable states probably have these things, actually, so I would list that in there.

**Q:** Thinking back, and this could actually be any time during your current practice as a primary care physician or earlier during your training as well, but in what sort of settings did you encounter this diagnosis or this phenomenon of people with a stroke that was described on a scan but it, at least, didn’t seem to be tied to symptoms or deficits?

**A:** So it probably happens most of all with older patients who come into the emergency room for weakness, lethargy, altered mental status, something that prompts a head CT, they fell, something like that. And it’s very clear on the CT they have an old area of encephalomacia that is clearly not a new infarct. And we're like, “Oh, I guess they had a stroke.” And then so that's really the context, it’s done for some other reason.

**Q:** What are some examples of this presenting symptoms or scenarios? You mentioned fall?

**A:** Yeah. I mean, like I said, I can't remember specific cases in which this has come up, but like older people coming in for altered mental status, it’s obviously extremely common in internal medicine admission and whether-- so often they have infection, whether it’s UTI or pneumonia or just viral syndrome, medication effect. So, I don't know how much-- but the presenting problem would be altered mental status. And then just sort of generalized weakness, I think, or like trouble walking that families describe as weakness, something that makes, for me the physician, think well, they could have had a stroke and maybe that's why they can't walk. I'd say those are the big reasons.

**Q:** In those scenarios, did you end up connecting the presenting symptoms to the findings on the scan or were they completely separate?

**A:** I would say they usually don’t end up thinking they're connected. Yeah, I mean often-- we often see people who we think have had a new stroke, but it turns out it’s just sort of recrudescence of their old symptoms because they have an infection or something like that. But usually in that case, they know they’ve had a stroke and they're coming in with symptoms. They're like, “Well, this is like my old stroke,” so that's-- the silent strokes, I think, usually end up being truly incidental and unrelated to why they're initially coming in, yeah.

**Q:** You've alluded to a concept of things being incidental and physicians looking for something, a symptom or some clinical problem and then they find something which doesn’t-- at least initially-- appear to be related to stroke as the underlying cause. So just thinking broadly, what does it mean for you, for something to be incidental?

**A:** I mean, it’s really just that, that you've just said. Usually, we think of it as coming up on imaging studies, although I suppose it doesn't have to be. You could have incidentally found-- such as the patient this weekend-- incidental hypoalbuminemia. I have no idea why. Any time you're ordering a test for another reason and then find something-- usually, we use that term-- it doesn't always mean it’s clinically significant. You can have an incidentally discovered renal cyst that we don’t do anything about and we're like that was incidental. And sometimes, you end up doing things about them, but are-- if you were-- even after you found it and you don’t think it’s related to the initial presentation.

So, like, you might find something you weren't looking for, but you're like, “Oh, I guess that's the reason. I wasn't even thinking about that.” I think I wouldn't consider that incidental, I would consider something incidental if it’s true and unrelated to your-- or like completely unrelated for the reason that you got the test.

**Q:** Sure. For these, for silent strokes, do you feel an obligation to respond to them?

**A:** Well, I think probably in the situations where they come up in the hospital, we haven't usually because as the inpatient setting, we're dealing with their presenting problem. And if we really don’t think it’s related, I haven't thought too much about it. Like I said, I don't think it’s come up for an outpatient of mine where I'm sort of managing the chronic issues.

**Q:** And you mention that your current panel’s relatively young.

**A:** Yeah, relatively young. I mean, I can sort of think-- I can think about whether I would do something.

**Q:** Sure. Let's keep it as a hypothetical for a minute. Assuming that some of your patients in your practice are going to end up aging over time or you're going to-- as you mentioned earlier, you're cross covering for some of your colleagues who have much older patients. Do you think you would respond to these, or ask your colleagues to do something about it or alert them?

**A:** You know, I think-- I've actually been thinking about this. I know you told me not to think in advance of this conversation, but I've been thinking about what would I do. And the question I always like to ask myself, as most people do, what will I do with the results of my workup? So, if most of these risk factors we’ll sort of already know about. Like I said diabetes earlier as a risk factor. But, most of those things we’ll know about, but I suppose if I found known stroke and the patient hadn't had an A1c checked in 15 years, seems unlikely, but if like that would be an easy thing to check and we know what people’s blood pressures are, but they hadn't had a lipid panel in a long time, maybe I would do those things because those are sort of slightly lower hanging fruit and the sort of less dramatic to intervene on.

Like, when I put someone on a halter and look for afib for a silent stroke and then commit someone to a lifetime of warfarin because of the silent stroke? Those are things that I would do as part of a stroke workup. Would I do that for a silent stroke? I don't know that I would. So, I think I might stop the workup. I would, as a-- add those test image and blood pressure, A1c, lipids. Obviously, I think I would use it if they were a smoker, like use that as a motivational interviewing tool for why they should quit smoking, why they should take their blood pressure meds.

I suppose also like carotid artery studies. That's an interesting question. I have to think about that. I would probably ask the neurologist. [laughter] Because I'm an internist. But, you know, it could also be a decision with the patient. “You've had this old stroke. These are some of the workups we can do,” if they're already pretty incapacitated and they're like 80s, I wouldn't-- doesn’t make sense to send that person for a carotid endarterectomy if they have these little--

**Q:** Fair enough.

**A:** But it might prompt a conversation with a-- do you think you want to get a lot of testing with the reason being to prevent future non-silent stroke? But [00:15:45] loud stroke.

**Q:** Right. Do you think you would change any medications or modify any treatments?

**A:** This lady who I now have this opiate [?] contract with also told me that she had a stroke earlier this year. She's like 47. She's like, “I had a stroke, they gave me TPA. I have no problems now.” This was all in Florida. And I was like, “I don't believe this is true, but like, you should take aspirin every day, I guess.” So I suppose if I saw a silent stroke and the patient weren't on aspirin, that would feel like the benefits would outweigh the risks there of starting them on aspirin if they weren't already. And then again, maybe being a little more aggressive with the risk factor control, whether it’s blood pressure, lipids, diabetes, et cetera.

So, I think those would be the main things. And if they weren't on a statin, I don't know. Probably the right thing to do would be to put them on a statin, although I'm sure we don’t actually know what the right thing to do is evidence-wise. But if you were to extrapolate from the clinical stroke literature, you would say they should probably go on a statin. But not having been in that situation, I don't know what I would do.

**Q:** Okay. Do you think encountering these patients, you would refer them to specialists of some sort?

**A:** Again, I think it would depend a little bit on the patient and if they were a younger, functional person that we really want to prevent future strokes, understand why this happened, I mean, I could order the workup myself, but I think I would probably refer them to a neurologist just to sort of get their opinion on the books. If it were a patient with a lot of co morbidities who their life expectancy was not that long and they already had a lot of, I guess, morbidity, I think I probably wouldn't.

**Q:** And you alluded to this, I think, with tobacco use, counseling. But do you think you'd ask patients to make any lifestyle changes or behavioral modifications?

**A:** Always. [laughter] I mean, in some ways, it’s always nice to have a little-- bargaining chip’s the wrong word but I'll just use it because that's what came to mind. For people with hypertension, we always get EKGs and if I can see they have LDH on their EKG I'll say, “Look, you're already having damage to your heart because of your blood pressure. If we can--“ but that's a silent thing that I sort of use to try and motivate patients. And this would be another [00:18:47] these patients are going to be people who need to make some lifestyle changes because they have co morbid disease. So for sure, I love having medical, like clinical, reasons to motivate patients to make lifestyle change in terms of exercise, diet, medication adherence.

**Q:** Right, okay. Do you think these silent strokes have caused harm to patients?

**A:** Hmm. I don't know. It always seems like every time you infarct a little of your brain, it’s probably caused some harm, like-- maybe they haven't. I don't know. I think patients would disagree on whether there's harm caused or not-- caused harm or not, like a lot of patients feel like if they have abnormalities on their studies, like, they have an illness. And some people are like, “I feel fine. Nothing is wrong with me.” And in some ways, they're both right. I don't know. I think the way I talk about it would depend on the patient, actually, again.

Patients who are more likely to brush things off, I might actually try to convince them that this was a harmful thing that happened to them. And patients who are more likely to freak out about everything, I might emphasize, like, this is not causing you any harm but I am worried about potential harm in the future. So, I think it would depend on how I'm trying to motivate the patient.

**Q:** So the interpretation might be patient-specific?

**A:** I think so, yeah. I mean, and if the patient asks me, like, “Am I going to have longer-term-- like does this mean that I'm going to get dementia?” I would probably tell them I didn't know.

**Q:** Okay. Do you think the patient specific characteristics in terms of if they are the type of person who would freak out or the type of person who would brush things off, does that affect whether or not you would tell them about the finding?

**A:** No. I think I would probably tell everyone about it, unless there was like a very sick, older patient, like with some-- like some end stage disease that I didn't want to complicate our conversations at all. I think I would probably always tell the patient, yeah.

**Q:** Fair enough. This is a related question in some ways. But, from your perspective, do you think that the silent strokes put patients at greater risk for other health issues? And if so, how much risk?

**A:** Oh, I mean certainly they're associated with other risks. So if someone’s had a silent stroke, I would say they're probably more likely to have another stroke, if the measure of underlying cardiovascular disease, they'd be more likely to have peripheral disease or an MI. But does that actual stroke put them at risk? I guess it might put them at risk of seizures like other strokes.

Whether it would have any effect on, like, cognition or something, I don't know. Yeah. It would depend on where it is, I guess.

**Q:** Fair enough. I'm **A:** company going to give you a laundry list of things that could potentially be something that silent strokes would put people at risk for. And these are not all right or wrong, so it’s just a list. And you can say to this question of do covert or silent strokes place your patients at risk of anything of the following, you can say yes, no or give some qualified answer.

**A:** Okay.

**Q:** So are you ready?

**A:** Yes.

**Q:** So, again, the question is do you think silent strokes place your patients at risk for any of the following? The first is stroke?

**A:** Say yes.

**Q:** Okay. Intracerebral hemorrhage?

**A:** Oh yeah, probably yes. Yeah.

**Q:** Myocardial infarction?

**A:** A lot of these-- so one thing, I was hearing you guys talk about strengths earlier. We discovered [00:23:53] that I'm really good at reading upside down so I've been looking at this list. So a lot of these I think are yes, but only through like-- because risk factors are associated. So heart attack, yes.

**Q:** You can say that.

**A:** Yes, via association.

**Q:** Heart failure?

**A:** Probably the same. Yes, via association.

**Q:** Sure. High blood pressure?

**A:** Same, and high cholesterol and diabetes, so via association.

**Q:** Sure, fair enough. Headache?

**A:** Everyone has headaches. [laughter] In my three, four months of primary care doctor, everyone has headaches. But, probably yes.

**Q:** Okay. Vertigo?

**A:** That I don't know. I mean, certainly depends on where it is. Although if the stroke’s causing vertigo, it’s not a silent stroke. So, would a silent stroke increase the risk of vertigo? A lot of people have vertigo, too. I'll say yes because so many people get it.

**Q:** Sure. Seizures?

**A:** That I think yes.

**Q:** Falls?

**A:** Again, I don't know. Maybe yes. And then memory loss--

**Q:** Memory loss?

**A:** Memory loss as separate from dementia? Maybe yes for memory loss, like in terms of, like, subtle memory loss. But I think no for dementia.

**Q:** How about anxiety?

**A:** No.

**Q:** Depression?

**A:** I would-- yes.

**Q:** Schizophrenia?

**A:** No to schizophrenia.

**Q:** Okay. In general, how comfortable do you feel treating patients with silent strokes?

**A:** I mean, do you think as comfort as something I've done a lot, so I'm comfortable with that I haven't done, so not comfortable from the perspective of like it’s familiar. But, it’s sort of familiar by association of I've seen plenty of patients who've had strokes. So, I think I could extrapolate what the workup is. So, relatively comfortable.

**Q:** Okay. What practice guidelines would you use to guide your care?

**A:** Oh, I don't know. I'm not-- I don't know, I doubt there's like general internal medicine guidelines about this. I'm not in the habit of looking up any sort of neurology focused guidelines. So I would have to see if there's an up to date article about it, or say there's a Dinamet [?] article about it and see if they reference any practice guidelines.

**Q:** I'll pose a slightly different question, which is actually not on the sheet of paper but it’s related to this. But one thing that is a question is does one follow primary prevention guidelines or secondary prevention guidelines? Because they're actually different for stroke in terms of what you might do for a particular patient. And so if you see something on the scan, do you consider that they’ve already had a stroke and you do secondary prevention or is it somehow different from other strokes and so you would follow primary prevention guidelines?

**A:** I think I would probably follow secondary prevention guidelines. If I, like, really thought about it during my practice. I think that would be my gut reaction, yeah.

**Q:** Okay. What do you think are the major knowledge gaps in this area?

**A:** I mean, the simple question of what's the natural history? What happens to people who have had these silent strokes? So, that would be the sort of baseline. I mean, it’s and all gut for me. I can only tell you what my knowledge gaps are. So, what is the sort of natural history, what are patients truly at risk for? And then presuming they're at risk for strokes in the future, do the usual secondary prevention interventions decrease the risk of that stroke? Those are the general questions I would ask. Yeah, I'll stick with that.

**Q:** This last question is-- it’s actually a bit of a thought experiment. And so, one thing that I'll kind of put as a framework, there's some information about risk factors for silent strokes and there's some information about outcomes. One of the outcomes that follows silent stroke, as you alluded to, is people at more risk for stroke with symptoms and deficits for garden variety stroke.

And so let’s imagine that there's a big study that's done. It's rigorous, well designed observational study that's looking at the comparative effectiveness of different potential treatments for preventing stroke after silent stroke. And let’s say that actually finds maybe the opposite of what you might usually do. So you mentioned, for example, aspirin, statins. Let’s say the study actually finds that the bleeding risk starting aspirin on all these patients actually outweighs the benefit of preventing stroke. Do you think that would be enough to alter your practice or sort of guide it in a particular direction?

**A:** I mean, in general, I like to think that I'm relatively attentive to risks of therapies. You know, of course the question with the observational study would be presumably it’s-- well, let me think about this. You're adding [?] to the patients who are at higher risk of having a stroke if it’s observational. Some treating physician was like, “Oh, this patient has silent stroke, and has hypertension and hyperlipidemia. They should go on the stroke regimen.” And then patients who they're like, “Huh, they had a silent stroke. That's interesting. I don’t really know why. They don't seem high enough risk to go on aspirin, we won't put them on aspirin.”

So, I guess what you're saying the finding is, so people who they thought were-- must have been in a sort of higher risk group to begin with, they got treatment, the aspirin caused more bleeding than-- do we know, did it work to prevent strokes? Did it end up preventing more strokes or does it not actually matter?

**Q:** Let’s say it prevented more ischemic strokes, but the GI bleeding actually ended up putting people in the hospital more. And so that frequency of events and the severity of it was enough to outweigh the benefit of preventing ischemic stroke.

**A:** Yeah, I think it would affect my practice. Yeah.

**Q:** Okay. Another potential hypothetical is, for example, if that's not enough, would you prefer that some sort of randomized controlled trial be done? Because you mentioned you're kind of thinking through before the selection criteria for people in an observational study. And what we would probably try to do is try and match people as best as possible to try and limit bias and selection or confounding by indication. And so it sounds like that could potentially sway your practice if it’s a well designed observational study?

**A:** Yeah.

**Q:** So, I guess I would sort of draw from that that you might not necessarily need a randomized trial to guide you?

**A:** I mean, it’s always nice. [laughter] But is it necessary, like once you have this result? I know there are some people out there who feel like well, unless they’ve showed a randomized control trial, I don't believe the results. I tend to be a little more flexible in my thinking, for better, for worse. I don't think anyone knows.

**Q:** Okay. In terms of studies looking at ways of preventing stroke or other things after silent stroke, what do you think are important outcomes to look at?

**A:** You know, I guess you want to look at incidents of stroke as one, but more importantly is sort of morbidity after stroke-- mortality from stroke, very important. Mortality and sort of serious morbidity after stroke. Like, if someone has-- if these people are high risk of stroke but they're, like, very subtle in their deficits that they tend to be very small, not ideal, of course. So you'd want to get a sense of, like, how-- how morbid are these strokes, sort of people’s long-term functioning. Those are always outcomes and then you listed sort of the side effects from if you're looking at medications and preventing strokes.

So, GI bleed, intracranial bleeds, the therapies you'd be using, I guess those are the big, big things, instances of, I don't know, hypoglycemia and hypotension from over treating risk factors. Quality of life outcomes are always useful.

**Q:** Yeah, no, it is. Definitely.

**A:** Basic quality of life, yeah.

**Q:** Okay. I'll mention a statistic to you. And so there have been a number of large cohort studies, prospective studies that have established some information about incidence and prevalence of these silent strokes. And in studies in the U.S. and in other parts of the world, the sort of average number, the mean across different cohorts for patients age 50 and above, is a prevalence of about 20 percent.

**A:** That's high.

**Q:** And so it starts off on the lower end, like around age 50 and somewhere in the 5, 6, 7 percent range. And then--

**A:** Still high.

**Q:** --dramatically goes higher as people age. And it’s much more common than stroke with symptoms or deficits where, for example, above age 50 in the U.S., it might be somewhere between 2 to 14 percent, just depending on how sick the population is. And so, I guess a question for you as a primary care doctor, this is a twofold question. But one is how do you handle these patients in your own practice in terms of let’s say we found out that everyone should be on an aspirin. How would you manage the patients that you've already seen? And also thinking about it more globally, how would you think from a public health or general population standpoint this issue should be addressed?

**A:** Always aspirins and statins in the water and beta blockers probably, while you're at it. I mean, part of this question is like how do you change practices mid-stream if recommendations and guidelines change. So, I mean, this happens all the time with, like, blood pressure goals.

But I think, you know, if-- right. If recommendations came out that aspirin is very useful for secondary prevention in people who've had silent strokes, you know, it would be another thing to add to the list of things to discuss with the patient. But, you know, a reasonable discussion. I guess it would be a question of if 50 percent of people have it over, say, over age 70, if it’s getting to be, like, that common, well do we just-- two questions. Screening, we should be screening for these with-- I guess you could just read PET CTs. I get really wary about screening for this type of thing.

**Q:** Do you want to go into that a little more?

**A:** It could be-- why I get wary?

**Q:** Yeah, why wary about screening?

**A:** Well, so we don't-- I mean, I guess-- so the question is what-- we're screening for things, we have to know that there is a reason to screen for them. So if we have pretty good evidence that we feel like big studies have been done, that like aspirin is-- truly decreases mortality and morbidity for people with silent strokes, then by-- maybe we screen for it. But, like, have to establish how often do you screen for it? Is it a one time screening when you turn 70 or 60 and then if you have a silent stroke after that, you don’t find it, like, I don’t really want to be scanning people’s heads every five years. Like, I'm pretty nervous about lung CT screenings for lung cancer and, like, all the new incidental findings that are going to come up.

**Q:** Right.

**A:** I think there's a little bit less of a risk for that with head CTs because they're, in my experience and maybe just because we're not screening-- like, there are fewer weird incidental things that come up on brain imaging than chest imaging. So that might not be so much a problem. And, in fact, it would be the things that used to be considered incidental that we’d actually be looking for now, right?

**Q:** Right.

**A:** But it’s just like CTs are not so expensive, but, you know, it’s an added cost to the system. Although caring for people with strokes is very expensive, like after they’ve had a serious stroke is very expensive. So preventing stroke is important. So, I mean, those are the main questions, is do we truly know that if we find something, do we know what to do about it? Do we just say, “Okay, we found a stroke. Aspirin, statin, make sure you don’t have diabetes [00:40:21].” That feels like it wouldn't be that hard to implement, although we don't screen for silent MIs with [00:40:34] and things.

**Q:** Right, yeah.

**A:** It's interesting, I don’t-- I mean, I guess we screen for diabetes mostly. So there is sort of, like, I guess a model for-- those are my scattered thoughts about screening. Or, like, the population level thing that you would think about if the prevalence is truly that high. Or, do we just screw the screening and go back to the day we used to recommend everyone take aspirin anyway. Maybe we go back to those days. Yeah.

**Q:** Okay. So, that's the last question, and I just wanted to open it up to see if you had any additional thoughts or questions that came to mind, or we could call it a day there?

**A:** No. I mean, I guess the questions-- it sounds like there is data to answer some of the things I didn't know about-- like there is data on the prevalence of silent strokes and do they tend to be more associated with the cardiovascular risk factors like--

**Q:** I'll tell you about that, actually, after we complete the recording.

**A:** Okay, sure. No, that's good.

**Q:** We’ll stop the recording right here.

END INTERVIEW

General Neurologist 2

Study ID: GN2

**Q:** So this is our interview with our second neurologist. And there are about 12 questions but they’re relatively short ones. Most of it is open ended. It just depends on how talkative you feel. And depending on how much you say, I might ask additional sort of side questions to get a little bit more detail. Again, this is just exploring what you do or how you feel about it, what your thoughts are at this time.

**A:** Sure.

**Q:** So the first question is just tell me what you know about silent or covert strokes so far.

**A:** I think the biggest question for me would be what exactly you consider a silent stroke. And I think it could be a variety of things. And when I think of them in my practice, one is, certainly there are small, lacunar things that you see on scan, and that’s one piece. And then occasionally you find something that’s much larger that sometimes I wonder how the person was not necessarily aware that something like this necessarily happened. So I guess—does that make sense?

**Q:** No, it does.

**A:** Okay.

**Q:** And actually, that’s something that people debate about in terms of what do we include in that group.

**A:** Sure.

**Q:** So what would you consider a silent stroke to be?

**A:** I’d probably include both of those. Because I think any of those things could require further risk factor modifications. So anything that would seem to be something vascular related that might make me think of how I might prevent that in the future, I would consider, which could be something lacunar and it could be something that appears more embolic in nature.

**Q:** Sounds good. The diagnosis or the condition or finding has been given several names that show up in our parlance as well as in the literature. And I’m going to mention a few of them to you. They include silent stroke, silent brain infarction, silent cerebral infarction, covert stroke, covert brain infarction, subclinical stroke, subtle stroke and asymptomatic stroke. And of these, which ones have you heard used?

**A:** Several of them, probably the most common being silent stroke, asymptomatic stroke, and subclinical stroke probably, are the ones that probably are most commonly used I would say.

**Q:** Are there any that you use or other terms that you might use?

**A:** I would probably use silent stroke more than I would anything else.

**Q:** And this may be the same, but is there a term that you think is most appropriate or most useful in terms unifying everything under the same--

**A:** It’s a great question. And I think in part the answer I gave to the last one and the three that I picked out were mostly because that might be the way I describe it to a patient. I’m not going to say covert, cerebral infarction to a patient. So I think those other terms are terms that I say, use, simply because maybe the patient will understand that a little bit better if you go to describe it to them.

So what was the next question? I’m sorry?

**Q:** Oh, it’s just what term do you think would be most helpful and most appropriate or most useful to use?

**A:** Probably silent stroke.

**Q:** Okay. Sounds good, focusing on what patients can understand.

**A:** Right.

**Q:** Why do you think these occur?

**A:** I think that a lot of people have vascular risk factors. And I think that it’s a sequela of those things that happen. And I think that why are they necessarily not clinical, is that the big question? Are you just basically saying, why do people have strokes as a whole?

**Q:** Both, actually.

**A:** Okay. I think it’s just that there is a lot of vascular risk factors out there that may not be either optimally treated and then you have people who just choose not to treat them. And then they end up with these problems, I guess.

**Q:** And then the second part of the question as to why they are not clinically apparent--

**A:** Yeah. I guess that sometimes, some of the more subtle deficits may be missed or misinterpreted by a patient. So it’s possible that they actually had something happen, similar to other diseases. MS, for example, some people may have never identified an optic neuritis. And then if you really go push them on it, maybe they’ve actually had something like that. So same thing with seizures, that obviously I treat a lot. They may never have identified something in the past that really was until you bring it to their attention.

So I think some of them may actually be something that was clinically, maybe subtly seen and you just weren’t necessarily aware of it. And then I think parts of it probably, maybe are in areas that are not something that people clinically, or that use on a day-to-day basis because if you don’t think of just a language problem or a motor problem, then maybe that’s not something that someone necessarily uses or thinks of or that, [as well][?], I guess.

**Q:** Great. From your perspective do you think that silent strokes and more garden variety, symptomatic strokes have similar or different pathophysiologies?

**A:** My suspicion is some of them. I would assume that some of the strokes that are from atherosclerosis or thromboembolic disease probably carry the same vascular characteristics as any of those—I think, yes. I would say yes.

**Q:** So yes, they are similar.

**A:** Yes, they are similar. I think so. Probably, to one of the mechanisms that we see as a cause of people being clinically admitted for a stroke. Then probably it’s, I’d say they are the same.

**Q:** And do you think there are different types of silent strokes? And if so, how would you break them down or who would you categorize them?

**A:** I would say yes. But I think it just goes back to the mechanism by which we think caused any stroke. So I’d probably categorize them very similarly to the way that we—when I look at a scan and I think about a patient who comes in for a clinically-defined stroke, then I probably question the same thing about those that are silent. So I would say, I would think about them being more microvascular in etiology versus being something thromboembolic versus something proximally embolic, I guess. So I think same breakdown.

**Q:** Do you think that silent infarcts are similar or different from white matter disease or leukoaraiosis?

**A:** It’s a great question. I had this debate on, actually, when the patient who got sent up to me from—this is off topic, probably.

**Q:** Please, go ahead.

**A:** --That got sent up to me from Florida, who I had seen for epilepsy and had a small area in the kind of posterior frontal region. It was larger than the rest of her white matter disease but she did have some punctate things there. And I paused for a little while and said, “What is this? Is this just more white matter disease or is there some other mechanism by which caused this?” I mean I suspect that white matter disease comes about probably from many of the same risk factors that strokes develop from. And so I think probably it’s a spectrum, maybe? So I think it is probably in the spectrum of things. And I think some of the smaller microvascular sorts of strokes that may be seen as silent strokes per se, probably can accumulate to white matter disease.

**Q:** Do you think--

**A:** Does that answer your question?

**Q:** Yeah. It does. It does.

**A:** Okay.

**Q:** Do you think that they have a similar or different clinical significance between silent strokes and white matter disease?

**A:** As far as clinical outcomes, certainly with really severe white matter disease, certainly other pieces from vascular dementias and other problems could be the sequela later on, I guess of this. But if I’m arguing that some of the smaller strokes can eventually lead to, especially the microvascular ones to that, then I guess that they could have the same in sequela if the risk factors aren’t treated correctly. I guess.

But I would say that the majority of times when I think of a silent stroke, yeah, I’m not sure that I—when I—and again, maybe I don’t know what I’m doing but when I see somebody with a lot of white matter disease, you know, I really think in my mind, hey, you really need to—we really need to make sure that your diabetes is under control, your hypertension is under control, your cholesterol is under control. But I think the same thing when I see a small lacuna, which may have been clinically silent. So I guess—okay.

**Q:** I think you may have just answered my next question but—but my next question was going to be, do you react differently to discovering that somebody has a silent infarct versus white matter disease or the same, same or different.

**A:** It’s a great question because I just debated about this with the same patient I was talking about because we had this discussion about aspirin, in particular. And I think my question would be, especially with some of the microvascular things, from a secondary stroke prevention, is it really something that is as helpful as it is treating diabetes and hypertension and cholesterol. So I would say not necessarily. But then I see somebody with what I see as, you know, lacunes down in the deep structures or I see something in the cerebellum and I feel very obligated to have, for some reason, this person on aspirin. And it probably should be no different, really, than the person who has, I guess, white matter disease. I guess.

So maybe or maybe not. I think it’s really an individual thing for me and I’m not sure I have definite, set way [simultaneous conversation] this.

**Q:** Sure. Sure. What do you think are the major risk factors for silent infarcts?

**A:** Smoking, hypertension, diabetes, high cholesterol are probably the four biggest maybe.

**Q:** What are the scenarios or settings in which you’ve encountered patients with this? Just some examples or some typical scenarios.

**A:** Yeah. I think sometimes scanning a patient for some other reason and then ending up with this probably is that number one cause. I would say review of other hospital records from somewhere else, so then looking at it and then seeing and wondering how it was addressed. I think an admission to the hospital, similarly for the same reason, for another stroke maybe, and then you say, they’ve accumulated things over time. So I think most of it is true, incidental findings on—maybe or maybe not it is incidental. But yeah, I guess for the most part it is incidental findings on MRI scan probably is the biggest way.

**Q:** And what were those scans ordered for? Like what were the symptoms or the issues for the patients?

**A:** Yeah. So I would say most of them in my practice have been for seizures or epilepsy and then have had other, have things like this found. So mostly it’s a completely different diagnosis that probably is not even vascular related and then it found something else on their scan.

**Q:** When you encountered these, did you think that the infarcts were somehow related to what the patients had? Or did you think they were unrelated?

**A:** Usually unrelated. I’ll say that occasionally someone with, particularly a focal epilepsy that has maybe a few—for example, this lady the other day, you know, had abnormalities in her frontal, temporal region on EEG. And I questioned, you know, what this area is. Am I right, that this is really just white matter disease? Is it possible this was a little demyelinating something. Is it possible this was some kind of little developmental venous anomaly or something else there.

So I think that probably if I, in the end, decide that this was either white matter, lacuna, or something else, probably not related. Although I’ll definitely say there are definitely patients, I can think of one, you know, a couple of months ago that it had a small right epsia [?] stroke that has developed epilepsy and it probably was a result of that seemingly silent infarct. Although he probably had findings on exam, he just wasn’t aware of those things. So I think that occasionally it turns out that it actually is related. And that guy, for example, this looked very embolic in nature to me. So actually undertook and entire stroke workup in the setting of the stroke. And his epilepsy probably related from that. So occasionally it can be the cause.

**Q:** So we sometimes use this term incidental where--

**A:** [Laughter] Which I used a minute ago and I decided whether that was the right answer or not. [Laughter]

**Q:** [Laughter] Right. –As a way of describing these findings where we’re looking for something else and then we find this, whether it’s on a scan or a test. And so, I was just going to have you, kind of from a philosophical standpoint explore what it means for you for something to be incidental.

**A:** In my mind it would be something that was found and probably has no relation to the process that you’re actually looking at. For example, someone is coming in for seizures and we find a small stroke there. I think that probably is incidental in my mind in that that wasn’t what I was looking for. It probably doesn’t have any clinical relevance with regards to their physical appearance or exam. I think the question becomes, if I decide to do something about it, where that term goes, you know, in that piece.

Because then I don’t say—I might initially say they had an incidental stroke but then eventually that will become something that maybe actually put in a different assessment. It becomes an assessment of its own of have we worked this up? And so then it kind of goes from this incidental to all of a sudden me, clinically, doing something about it.

**Q:** Which actually brings me to my next question, which is, do your feel obligated to respond when you see these?

**A:** I often debate about that myself. Usually, at a minimum, if I see something on the scan, I usually at least bring up to the primary care doctor that maybe there should be an assessment of their vascular risk factors because probably if we found this in the brain, they probably have something that also could affect heart peripheral vessels. So I feel like at least there needs to be some attention brought that this person seems like they’ve had some sort of damage done to vessel that may be able to be modified and prevent future morbidity. So I feel like at a minimum that needs to be brought up.

As to what else I’d do about that, just a little bit depends on, again, what I think may be the mechanism is. So I would say for the most part—and then I also want to know if they had prior—obviously, it doesn’t become silent if they had prior workup for it and knew about. But obviously I find things on scan sometimes and then you go and take the history and they really were worked up in some other place and actually really had, you know, clinical findings of the time of it, too. And then that kind of excludes it from this interview. So, yeah.

**Q:** Do you ever end up changing treatments?

**A:** Yeah, I can say occasionally. There’s a lady that I have that has pretty bad lupus that has what looks to be silent basal ganglia, you know, infarcts. And I’ve suggested she be on aspirin, I guess, forever, essentially in the setting of this. She was definitely not on it before, whether that was the right decision or not. And then we’ve also talked about her other risk factors and things like, too. So I say, absolutely. Yeah.

**Q:** Did you order any tests or do any additional evaluations?

**A:** Sometimes I will, depending, will assess labs, just to see whether the hemoglobin A1c is, what their LDL is, those sorts of things. I can certainly talk to them about smoking if it turns out they’re a smoker. And then if it really looks like to me that I guess some of the smaller, deeper strokes could be embolic as well, but more I think of something that’s cortical, web shaped, you know, something along those lines—then I sometimes will go down the line of ordering a full, what I would consider stroke-up to look for what, at that point in time seems to be, without the workup, something cryptogenic—that needs to be figured out why they had this to prevent others.

So I’ve definitely ordered ECHOs, longer volters [?], vessel imaging, carotid imaging for these. So, yeah, I have.

**Q:** I know that you have some level of comfort treating patients with stroke. But do you bring in other types of specialists, whether in other fields or in similar fields, again, to help you figure this out?

**A:** Depending on what it looks like, possibly. Yeah, I mean I think a little bit depends on—I’m trying to think about any of the ones that I—I’m not sure really that I’ve necessarily referred—I guess unless I found something really atypical and part of the other workup and I was questioning, maybe treatment options there, I’m not sure that I necessarily would, just in the workup kind of stage of things. But maybe later on it may be that that is the case.

**Q:** What type of people would you get involved?

**A:** It may be that I—it could be that I ask one of you guys who really are stroke specialist, you know, to see or take a look at it or to look at an image or, you know, those sorts of things formally or informally. And I’ve sent people to nutrition in the setting of diabetes. I’ve sent people to endocrinology. I’ve sent people to rheumatology at occasional times. I think that is probably the majority.

**Q:** Okay.

**A:** And the primary care doctor.

**Q:** The primary care, of course.

**A:** Okay. Yeah. [Laughter]

**Q:** [Laughter] Do you think that these silent strokes have caused harm to patients?

**A:** In some cases, yes. In all cases there probably has been some, even if it’s not clinically obviously relevant, there has been some change in the brain structure as a result of it. So I guess, yes, in the strictest sense, all have. But whether it’s something—and I also think that just because they’ve have one, they may be at risk for others. And so I think that—yes, I guess you have caused harm to the patient.

**Q:** From your perspective, how much do silent strokes put patients at greater risk for other health issues? And what might those health issues be?

**A:** Yeah. I think that if they can have something vascular happen in the brain, then certainly it could be something from cardiac standpoint. So certainly they would be at risk for MIs. They could be at risk for chronic kidney disease in the setting of hypertension. They could be—or the other risk factors. Peripheral artery disease, so limb-related problems, vessels. And then the same thing, diabetes, certainly retinopathies, neuropathies—so, yeah.

**Q:** Okay. To explore, actually, that a little bit more in terms of the idea of silent strokes putting people at risk for other things, or being a marker of risk potentially, I’m actually going to give you a little bit of a laundry list. I’m just going to name a condition. And the frame of the question is, do you think silent strokes place your patients at risk for any of the following. And you could say, yes, no, or give some qualified answer between that.

**A:** Sure.

**Q:** Okay?

**A:** Okay.

**Q:** So the first one is stroke.

**A:** Yes.

**Q:** Intracerebral hemorrhage.

**A:** Yes.

**Q:** Heart attack.

**A:** Yes.

**Q:** Heart failure.

**A:** Yes.

**Q:** Hypertension.

**A:** Yes. That may be a cause but I guess it could be—I mean any relation you said. No. At risk for.

**Q:** I mean I was describing it as silent strokes putting people at risk for disease. But if you think that it’s a cause of silent strokes, you can say that.

**A:** Okay. Yes.

**Q:** So you think hypertension is a cause of silent strokes.

**A:** Yes.

**Q:** Okay. How about high cholesterol?

**A:** The same as for hypertension.

**Q:** Okay. Diabetes?

**A:** The same as for hypertension.

**Q:** Okay. Headache?

**A:** Probably not.

**Q:** Okay. Vertigo?

**A:** It may have been the result of. Yeah, sure. Yes.

**Q:** Seizures.

**A:** Yes.

**Q:** Falls.

**A:** Yes.

**Q:** Memory loss?

**A:** Yes.

**Q:** Dementia?

**A:** Yes.

**Q:** Anxiety?

**A:** Maybe when I tell them that they’ve had a silent stroke.

**Q:** [Laughter]

**A:** [Laughter]

**Q:** Okay. The sort of active anxiety as a result of receiving the diagnosis.

**A:** Right.

**Q:** Okay. Depression?

**A:** Yes.

**Q:** Schizophrenia?

**A:** Hmm! No.

**Q:** And then the next question is how comfortable do you feel treating patients with silent strokes?

**A:** [Laughter] Given that as you can tell in this interview that I’m not sure what I’m doing necessarily with each patient, I guess I don’t really have any guidelines to say that I’m doing something necessarily the right way or the wrong way. So I just try to treat the individual and essentially go back to kind of the basics of what might have caused this. So I guess I feel okay doing it.

**Q:** Okay. So kind of in the absence of guidelines you’re individualizing care and trying to sort of figure things out on an individual basis what to do.

**A:** And I do have my own little anxiety provoking states of trying to figure out whether I did the right thing or not but, yeah.

**Q:** Fair enough. What do you think are the major knowledge gaps in this area?

**A:** Probably, maybe lack of consensus recommendation on, you know, workup and treatment. Although, again, I guess it just goes back to—yeah, I guess probably just—I guess that’s probably major. Yeah.

**Q:** Is there anything else you would want to know about?

**A:** With regard to silent strokes?

**Q:** Yeah.

**A:** I mean I’d like to know whether others are treating them the same way I am or the docs I guess. Yeah.

**Q:** Okay. Fair enough. This last part, we’re coming close to the end, but the last part is a little bit of a thought experiment. And the idea is to see how your practice might change given information from new studies, studies that haven’t been done yet. So let’s first imagine that there is a big, rigorous, well-done observational, comparative effectiveness study. And that was done to determine the effectiveness of prevention therapies for silent strokes.

So somebody has a silent stroke. You start them on a medication. And you want to prevent—let’s say the outcome is asymptomatic stroke, which we know is an outcome after silent stroke.

**A:** And obviously I’d like to know this going back to the question before, what the obvious end outcome for--

**Q:** Sure.

**A:** Yeah. Of course. So, right.

**Q:** So this is clinically meaningful outcome, one that you would expect. And let’s say that actually the study finds that the opposite of your, let’s say, on average, typical practice is actually better. So let’s say you start a lot of these patients on an aspirin and the study finds that the risk – benefit ratio for aspirin is actually unfavorable because maybe more of these patients are having bleeding complications instead of having fewer ischemic strokes. Would this type of study, in terms of how it’s designed, it being an observational, comparative effectiveness study, would that be enough to convince you to change your practice?

**A:** Probably not.

**Q:** What type of things would you need to help guide you? Like what sort of studies?

**A:** Yes. I’d really like to see a controlled trial on patients that have gone, you know, onto or off of. But I think that, and really treated with kind of more maximal medical therapy versus those that are maybe just treating the risk factors, you know, for—and I think it would have to be well-designed based on different mechanisms as well, just because as you saw I included a lot of different mechanisms that all go into silent strokes. So I mean I think it would just have to be designed there. So I think an all-comers observational study of this probably would not change my management.

**Q:** If a randomized control trial were performed, so looking at these types of treatments for prevention, how do you think that, how do you think patients should be recruited into that type of study? It’s a little bit of a harder question.

**A:** Yeah. I think I would recruit them from general neurology practices in which strokes have been seen, quote, incidentally on scans. And then I think I probably would, again, have to subdivide those into possibly likely mechanism, I guess. [Pause] Maybe.

**Q:** Okay. What do you think would be the important outcome to assess in either type of study?

**A:** Yeah. I think, I guess primary outcome would be—well, I guess a few. One would be clinical stroke because I think that white matter disease and others could potentially lead to morbidity as well. I mean I think silent stroke is another outcome. Then I would also assess other, what I think are related to strokes, from that laundry list of things between MIs, intracerebral hemorrhage and those. So I would want to know those things as well as I would want to know complications of my treatment as well.

So obviously I’d want to know if people really are having increased hemorrhages, you know, as well. So I guess that would be what I would want to look at.

**Q:** Great. Great. This is the last part of it but let me give you a few known statistics.

**A:** Okay.

**Q:** So there have been a number of several, large cohort studies, like the Framingham Heart Study, the Cardiovascular Health Study, the Northern Manhattan Study, Rotterdam Scan Study and so on. And they are mostly looking at people who are on the older side, over age 50. And what they found pulling the data together, or at least on average is that for individuals over age 50 the prevalence of silent infarcts is about 20 percent. It starts off a little lower, towards age 50. Framingham is somewhere in the middle. And it’s around 5 to 7.5 percent in the like 50 to 60 range. And then once you go up a decade then it bumps up dramatically to 15 percent. And then another decade then 25, then 35 and so on.

So knowing that—so that’s sort of the prevalence in the over age-50 population. We also know based on epidemiologic data that silent strokes are associated with a two to fourfold increased risk of symptomatic stroke. And them a similar, it is actually a two to threefold increased risk of dementia with vascular components, vascular dementia, Alzheimer's or mixed vascular Alzheimer's.

So those two pieces of information, let’s say if we were to find and effective prevention strategy, even if it is just modestly effective in terms of preventing these outcomes, do you think it would actually be worth implementing some type of screening strategy for silent strokes?

**A:** Some sort of screening strategy, so similar to us thinking about lung cancers and doing screening CTs or those sorts of things—that’s a great question because I think the things that—I mean you could screen for the risk factors but screening for actually having a stroke is a pretty high cost to society and the health system. That being said I’d like to know the numbers of cost and hospitalization in patients that developed the sequela afterwards because I would assume that those patients have developed dementia and other secondary strokes have a very large cost to society, too.

So, gosh, I think it would have to be a—I think there would have to be a bit of a—I think there has to be a cost analysis on any screening strategy that you—and, again, how effective you’re going to be at preventing those other things once you find it. And that you have some—so you find it. Like I said, I found all these but I really don’t know what the most effective thing is already. And so until I know that some of the answers to the other controlled trial that you did earlier, then I wouldn’t implement something screening--

**Q:** Until there is [simultaneous conversation]

**A:** Exactly. Right. Yeah.

**Q:** Okay.

**A:** I guess.

**Q:** Fair enough. So this is actually the end. I wanted to see if you had any additional questions or thoughts that you want to express on the tape.

**A:** I don’t guess so.

**Q:** No? Okay. So we’ll stop the recording right now.

END OF INTERVIEW

Internist 3

Study ID: IN3

**Q:** So this is the interview with our third internist. And there are about a dozen questions. They’re kind of relatively short questions and you can explore them and explain in as much detail as you would like. I might add a few questions just to pick your brain a little bit more.

**A:** Sure.

**Q:** And so the first question, just very open ended and it’s just tell me what you know about silent stokes so far.

**A:** Okay. I guess it depends on the etiology of silent strokes. So all you know is that it’s an infarct but it could be embolic or thrombogenic. I sort of categorize silent stroke as the same as a non- silent stroke. I think it is just the radiographic versus clinical. Hopefully, all clinical people have radiographic evidence but not necessarily all radiographic evidence means clinical evidence. I think I break it up in the same way that I break up non- silent strokes, either embolic or thromogenic or thromboembolic [?].

**Q:** Okay. There are a number of names that have been used by clinicians, radiologists or in the literature to refer to these. I’m just going to mentions a few of them. Some people have called them silent strokes, silent brain infarction, silent cerebral infarction, covert strokes, covert brain infarction, subclinical stroke, subtle stroke or asymptomatic stroke. Have you heard any of these terms used before?

**A:** Hm-hmm. Yes.

**Q:** Which one have you heard most often?

**A:** Probably most of them. I think silent stroke, asymptomatic stroke, subclinical stroke, those would probably be the top three I guess that basically come across my brain at the moment.

**Q:** Have you used them to described them to somebody else or are there any particular terms that you’ve used?

**A:** I would probably say silent stroke or asymptomatic stroke are probably my two most common verbiage phrases. I guess I equate them to silent MI, which is a similar concept, still an MI.

**Q:** Right.

**A:** The same with strokes.

**Q:** Fair enough. These may be the same ones. But do you think there are terms that are more appropriate or more useful to be using in the literature just because we have a wide variety that people are using?

**A:** I do think it’s helpful to have some kind of a standard nomenclature. I don't know if I have a strong preference over one versus the other. I guess my personal thought would be asymptomatic stroke is probably the best because it sort of implies it is a stroke without symptoms. I feel like the terminology just works the best for my particular brain. But I think it is more of a PR problem than a clinical problem, I guess.

Whatever works. I think in the seventies they came up with heart attack to sort of inspire people to do something about it. I think whatever inspires the most number of people, I’d be happy to support the cause. But I think standardizing it is a good idea.

**Q:** Fair enough. I think you answered my next question already, which was going to be, why do you think silent strokes occur. But it sounded like you were saying that you think that they are sort of the same mechanisms more or less.

**A:** Yeah, I think the same mechanisms. I guess presumably thrombotic is more likely than embolic with silent strokes would be my personal assumption. But having no data to back that up I don't know if that is true or not.

**Q:** Okay. But why do you make the hypothesis?

**A:** Because I think the microangiopathic diseases would be more likely with thrombotic as opposed to embolic. And they are more likely to end up with lack of symptoms because they are slow and subtle and relatively small brain surfaces. Whereas embolic is as likely to be small and large. So presumably the bell-shaped curve is going to have a many symptomatic as non-symptomatic. Whereas the thrombotic almost always is going to start with small vessel and progress to large vessel. So it’s probably not quite the same bell-shaped curve would be my guess.

**Q:** So I think you mentioned, besides pathophysiology, you were kind of exploring why they might be silent. And so you mentioned they might be smaller. It might be the location.

**A:** Correct.

**Q:** You also mentioned that they might be slower. What do you mean by that?

**A:** I don’t remember saying slower but I’m sure I did. [Laughter] So I guess my thought would be that they clinical progression would be slower. So you’d lose a capillary first and then the arterials and then an artery sort of backing up. So the most distal would occlude first or you’ve have watershed thought of the small vessel areas. So the area, the surface area of brain tissue that’s perfused by capillaries is obviously much less than a major artery. So I think you would probably see the microangiopathic changes distally before you would see them centrally.

**Q:** That makes sense. Yeah. Do you think there are any differences otherwise in terms of the pathophysiology of silent stroke versus symptomatic strokes?

**A:** I’d assume yes. But I would probably just be making up potential reasons. And it probably comes to that bell-shaped curve that of symptomatic strokes embolic or thrombotic, given the pathophys behind it, [00:05:25] changes the percentages. Silent strokes, given my previously stated theory that, you know, silent strokes or asymptomatic strokes are less likely to be embolic. Therefore the percentage is obviously going to be proportionally increased in the symptomatic strokes. But that’s a guess.

**Q:** Fair enough. Still a lot to learn.

**A:** Yes.

**Q:** I think you also answered my next question, which was the idea of there being potentially different types of covert strokes. So my question was going to be, do you think there are different types? And how would you characterize them? I know you mentioned embolic and thrombotic.

**A:** Yeah. I guess that’s hard. So this is probably against what I said a minute ago but--

**Q:** That’s fine.

**A:** --You said covert strokes, covert, whatever the term, silent strokes, you are discovering them radiographically, presumably because there is really no other—if they are covert by definition you are not finding them clinically. So there are going to be incidental radiographic findings. So probably the most logical way to categorize them is going to be subradiographic terms would be my thought. So geographically would be an obvious answer, you know, white matter, gray matter, you know, brain stem, cerebrum.

I think probably that would be one aspect of it and then there would have to be some kind of a size criteria. But radiographically as far as I know, you can’t really distinguish embolic versus thrombotic. So I think that would be a supposition that would get to the etiology. That would probably require more workup, which is hopefully going to happen with every stroke that you find. But you wouldn’t be able to name it if you required that of the definition. So I think probably my suggestion would to define it by the radiographic evidence that you get when you find it. Is that what you’re asking?

**Q:** It is. Yeah, it is.

**A:** Okay.

**Q:** In terms of radiographic findings, do you think that these asymptomatic infarcts are similar or different than white mater disease?

**A:** Yes. But I don't know if you would be able to tell that radiographically. And that might be lack of my knowledge as opposed to a radiologist’s knowledge. But, for example, amyloid disease you can get leaky blood vessels that might cause a stroke clinically or subclinically but is very different from my previously thought of embolic versus thrombotic. So it is sort of a different disease pathology but it might lead to the same infarction of brain tissue.

So I don't know if I would call that a stroke or if I would call that whatever the disease process is, progression of amyloid, progression of something else. But when you find it on the radiograph I don't know. I don't know if you would be able to define that.

**Q:** Sure. Do you think they have similar or different clinical significance?

**A:** I think that clinical significance would just matter which part of the brain, how much and which part of the brain died. Why it died probably doesn’t really matter very much. The plasticity of the person whose—the age of the patient would matter in terms of response and recovery afterwards, but probably not. I guess you’re right. The etiology would matter as much in terms of the effect.

**Q:** Would you react the same or differently if you found one versus another?

**A:** I guess yes because there are prognostic implications. So let’s say embolic, for example, I’m going to really be nervous, presume [?] this is a cardiac source or a carotid source. And so I would intervene on the source. If it’s thrombotic there is not much that I would do at the source I would put them on an aspirin or a statin, aspirin and statin. And if it was amyloid or some kind of a leaky blood vessel problem, you know, I might avoid anticoagulation.

So yes, I think the reason would definitely—it probably wouldn’t change their rehab potential or their—like if they had hemiparesis I would still send them to Spaulding rehab, you know, or Kindred or something. But, yeah, the etiology would probably matter a lot.

**Q:** I’m just going to do a sound check, just to make sure we are doing okay.

**A:** Are you doing okay for your answers?

**Q:** Yeah. No, these are great.

**A:** Okay.

**Q:** My next question is what do you think are the major risk factors for silent strokes?

**A:** I think they’re the same as non- silent strokes. So A-fib, carotid disease and then all the atherosclerotic risk factors, smoking, age, male gender, low HDL, family history. I’m sure there are a million others that I can’t think of.

**Q:** Good. I think that gets across the point that you think they are the same.

**A:** Yeah.

**Q:** And are there particular scenarios or setting where you’ve recalled running into this situation where you are evaluating a patient, or they’ve had a scan and you realize that there is this finding that’s of infarction that was discovered incidentally?

**A:** Hm-hmm. I can’t remember any particular patient names but this definitely comes up not infrequently. I mean I can’t say it’s common. But it definitely comes up. I feel a little bit conflicted about them because I feel like there isn’t the recognized clinical entity of this. So in my mind I equate the analogy to the heart attack that I mentioned before. That if I found, you know, suddenly someone’s injection fraction is 40 percent instead of 60 percent and they’ve got regional wall motion abnormalities, even they may not know they had a heart attack in the past but I still tell them they had a heart attack.

I do the same with stroke. I don’t feel quite as evidence based in that. So I feel like it is sort of my own personal philosophy as opposed to literature based. And I do feel like I can get at the etiology question you had before. I don’t have as firm a ground to stand on because there are, obviously, multiple possible etiologies, but the same concept.

So I treat them as if it was a thrombotic. But I think of embolic possibilities. So I’ll get an EKG for A-fib, you know, make sure I check the regularity of their heart a little bit extra each time, listen for bruits in carotids. So I’ll do sort of a complete clinical workup when I find it. But then often I will put them on an aspirin instead as stroke prevention. And I’ll call them secondary prevention as opposed to primary prevention at that point.

**Q:** Great. You’ve answered the next four questions.

**A:** [Laughter] Oh, I’m sorry.

**Q:** No. No, that’s perfectly fine. This is meant to be very fluid in a way.

**A:** Okay.

**Q:** I was wondering if you can think of any sort of symptoms, like presenting symptoms or situations that, for the patients themselves—like what--

**A:** Oh, you mean like what would have prompted my CAT scan or MRI in the first place?

**Q:** Exactly.

**A:** So often it’s a fall that we get these problems but it can be anything . So I work on both inpatient and outpatient. This is supposed to be just outpatient, though, right?

**Q:** No, it could be anyone.

**A:** Anywhere. So inpatient I often will see—I work on the geriatrics medicine service often. And so patients will come in with a fall. And if they have a head strike then they will do a pan-man CT of just about everything they might have hit on their way down to the ground. And that always includes a head. So they’ll get that. Delirium is a very common presenting symptom of just about anything in the old people. So they’ll get either a CT or an MRI for some reason. So they will get imageries based on that.

And then a variety of outpatient complaints, headache that’s been persisting for a while. Obviously, if there is a focal, neurological complaint, I don't know if this would really fall under the asymptomatic stroke category. So it depends, I guess, anatomically where it is.

**Q:** Right.

**A:** But you might have a brain problem that has radiographic evidence of that etiology but another finding in addition. This comes up all the time for all sorts of radiographs, CAT scans with nodules and things like that. So the same for the MS, you know, other strokes, you know, a symptomatic parietal lobe and an asymptomatic frontal lobe and vice versa. So I think, yeah, there are innumerable reasons to get—any reason you get a CAT scan us probably the reason you would find an incidental finding; an MRI I guess even more so.

**Q:** Yeah. This is a little bit of a philosophical question but we often use this term incidental in reference to things what we didn’t expect to find, found on testing for the evaluation of something else. For you, what does that mean? Like do you—is it—I guess a lot of people refer to things as incidental and there might be some variation in terms of whether or not to respond to that.

**A:** Yeah.

**Q:** So what does it mean to you and how do you decide whether or not to respond to something?

**A:** I guess to me it gets to my reason for getting the test in the first place. So it’s sort of two wrong make a right. So let’s take stroke, for example. If they have a stroke on the CAT scan, my getting the scan doesn’t change the existence or non-existence of a stroke. It’s either there or it’s not and I found it or I didn’t. Incidental implies that I didn’t know to look for it, which is probably a bad thing. I guess if it was asymptomatic by definition there was no reason to look for it in the first place. So that’s okay.

But we find incidental things all of the time that we probably should have been looking for. So screening guidelines being what they are, you know, like lung cancer screening trial for example, we should be getting CAT scans on a lot more people then we probably do. And no screening trial is 100 percent [00:15:59] rate. But incidental—I guess would really just get—for me, my reasoning behind getting the test in the first place, not the outcome of the test.

**Q:** You mentioned some things about, if I’m recalling correctly, in terms of how you might respond. So, for example, it sounds like if you thought that something was embolic you might try to, from the clinical standpoint check clinically to see if their rhythm is abnormal or irregular. Or you might listen to their carotids to see if they have a bruit. And then it sounded like you—you mentioned you might start an aspirin and statin if it otherwise doesn’t look like there is an obvious embolic source.

**A:** Yep.

**Q:** Do you pursue any additional testing beyond that? What do you do sort of in the office? You mentioned the EKG. But I wasn’t sure if there other things you might--

**A:** Yeah. So if they’ve had any palpitations, I probably would pursue, you know, rhythm changes with EKG, Holter, either 24 hour or 48 or even like a loop. If they are asymptomatic from a heart perspective, so they don’t ever feel palpitations, I don’t think a loop is as helpful as a Holter. You can get the implantable monitors are well if you really are suspicious. And I’ve had a couple of people that I’ve done this for. You know, if it’s a lot of strokes, for example, that are asymptomatic, that’s really suggestive of embolic rather than thrombotic. And the consequence of missing that is so high that you really want to make sure you ruled out—the most common etiology that I know, aside from maybe the carotids—A-fib and carotid disease are the two, top things that I always think about.

If they have a PFO I guess, low [00:17:45] DVT, is on the list. But that just seems like such a rare cause, DVTs being not so common; PFOs only about 25 percent. So the two together causing a problem seem so little that I don’t typically do that in my workup. But I think carotids and heart, carotids and rhythm of the heart are definitely the two, most common things that I look for when I’m thinking embolic.

**Q:** [00:18:11] End up ordering ultrasounds or angiography?

**A:** Yeah, usually ultrasound just because it’s so easy. And the bruit on exam, I don't know the sensitivity and specificity but it can’t be very high, certainly with my stethoscope is not as high as in studies. And I don’t even know if it is very high in studies, either.

**Q:** Do you ever order any blood tests?

**A:** Probably as a precursor because anyone on aspirin, I want to get a baseline platelets and coags just to make sure. I don’t think I would—I always do because it’s so easy to get blood tests. But I’m trying to think if there is anything that I look at in particular for causes of stroke versus implications for treatment. So I probably get lights, BUN and creatinine because if they have A-fib, they probably have hypertension or they might have LVH. And so I want to be a little bit more aggressive about their blood pressure. So that’s going to involve an ACE inhibitor or a diuretic, something that is going to affect kidneys and electrolytes.

I always get a starting statins. I’ll have a baseline cholesterol, even though the statin treatment will be regardless of their LDL levels. So I don't know if it really influences my management but I get the data anyways.

**Q:** Yeah.

**A:** Yeah, I’d say probably a chem 7, are they diabetic. You know, in terms of sort of flushing out all of the possible cardiovascular atherosclerotic risk factors. Hopefully I’ve already gotten those before this event. But if haven’t, that will be sort of a timeframe to look at it again.

**Q:** Besides antiplatelets, statins and then an anticoagulant, if you find A-fib, are there other treatments that you might recommend to these patients?

**A:** I guess it depends on what I find. So if it’s A-fib, is it because of hypothyroidism? Is it because of caffeine? Is it alcohol is it hypertension and LVH or diabetic cardiomyopathy. Like I said, it would be downstream stuff. I don’t think anything I would have as a lock-step for stroke other than aspirin and statin or antiplatelets and statin, aspirin plus Plavix but not Plavix, Aggrenox or something. No, I think that is about it.

**Q:** Do you ever talk to your patients in this setting about life style modifications?

**A:** Oh, yeah. Okay. That’s a true point, I guess non-pharmacologic stuff. Yes. So I’m a huge proponent of exercise. So any time I see these kinds of things there is always a chance to harp on that. Dietary things I think, I don’t think of anything in particular for stroke prevention other than modification of risk factors. So if a hypertensive, salt avoidance. If they are cholesterol, I am going to try and avoid saturated fats. More vegetables and fiber for things like that. If they’re obese, caloric management in terms of managing all of their other risk factors; diabetics for sugar. Does that answer--?

**Q:** It does.

**A:** Okay.

**Q:** And when you see these, do you ever end up--

**A:** Oh, and then smoking cessation, obviously, I should have mentioned.

**Q:** Of course. Do you ever end up referring these patients to anybody?

**A:** Sometimes. I can’t say it’s 100 percent, particularly if they’re asymptomatic. I guess it depends on what it looks like. So if it’s a single, single focus probably I would just manage that on myself, on my own. If I’m debating, if it’s multiple foci that always brings up embolic for me. And then I might push a little bit more for a neuro workup.

Or if I’m really concerned about irregularity of the heart, I might send straight to EP, like a Tilt-Table Test or something and I do EP study—like what do I really need to do? Particularly if it’s—obviously, I don’t even do implantable monitor. So if it is going to be anything even remotely close to that I’ll send them there. But usually I try to get all of that stuff in advance. I’m trying to think if I have a baseline ECHO for every single person. Probably. Yeah. I’d be hard pressed—I think I would get an ECHO for everybody, too.

I forget which question you asked me. You asked this earlier. I’m sorry.

**Q:** That’s okay. Yeah. You can jump around if you like. [Laughter] That’s fine.

**A:** Yeah. I think I would probably also get an ECHO, I guess, just a TT. I don’t think I would go for transesophageal for every single person but at least a TTE probably for everybody.

**Q:** Do you think that these asymptomatic or silent strokes have caused some harm to the patients?

**A:** Oh, yeah. I don't know if it—I think everything is a spectrum. By definition if you’ve lost a brain neuron, that is harm. Whether or not it has any macroscopic effects, you’ve got microscopic effects on the radiograph already. So, yes.

**Q:** What sort of harm do you think it’s caused?

**A:** So I guess it depends on where it is. If it is in the occipital lobe, it is going to cause some microscopic vision changes that you might not perceive because you’ve either got a plastic enough brain to have compensated. But now your reserve is lower. So another stroke will be more harmful than that other stroke would have been on its own. So I think at the bare minimum you’ve used up reserve. You know, the worst case scenario you’ve got death because the spectrum of stroke is all encompassing.

But I think there is also like a non-clinical knowledge of having had a stroke for some patients is debilitating, the same as heart attacks. Although I think heart attacks are more in the news, strokes are—again, I’d link those two as atherosclerotic disease burden manifestations usually.

A lot of people are really shaken up by the fact that they’ve had a stroke. So I think psychologically there is a huge burden for some people. Some people don’t care, which is probably a problem in and of itself. But a lot of people, it’s almost too much.

**Q:** Do you think the silent strokes puts individuals at risk for other thing?

**A:** Yes, more strokes, certainly. I would put them in the secondary prevention category. I would say, once you’ve had one, regardless of your clinical manifestations of it, I would call you a stroke victim and [00:24:54] secondary prevention. And as far as I know, there isn’t really much primary prevention for strokes other than behavioral modifications and risk factor modifications. But as soon as you’ve got one, regardless of what you look like clinically, I’d call you secondary prevention.

**Q:** Anything else that you think these put people at risk for? Or is stroke the big one?

**A:** I’m sorry?

**Q:** It sounds like for you, stroke is the major risk factor.

**A:** Stroke is a major risk factor for future strokes.

**Q:** Yeah.

**A:** Yes. I guess—you mean silent strokes or strokes?

**Q:** I mean silent strokes, is there anything else besides symptomatic strokes that you’re concerned about?

**A:** Because I think silent strokes by definition don’t have any clinical, visible clinical consequences. So I think it really would just be repetition, I guess, which would then have all of the downstream effects of, if you get another big one, you die, hemiparetic and other things.

**Q:** I’m actually—just to explore that questions a little bit more, I’m basically going to give you a laundry list of different conditions or symptoms and see if you think that the silent strokes put your patients at risk for any of these. So I’ll just give you a list. You can say, yes, no, or you can give some kind of qualified answer for that.

**A:** Okay. Okay.

**Q:** So the first thing is stroke.

**A:** Sure. Yes.

**Q:** And the second is intracerebral hemorrhage.

**A:** Yes.

**Q:** And the next is heart attacks.

**A:** Can I give a qualified answer?

**Q:** Yes, of course.

**A:** Because if I say yes—I guess, so for me atherosclerotic burden is an entity. And where you have that burden manifest is sort of random. And so if you’ve got atherosclerotic burden in your brain you probably have it on your heart. So I don't know if the silent stroke is a risk factor for heart attacks but the disease process that causes silent stroke could also cause a silent MI.

**Q:** So they have a shared etiology.

**A:** Yes. Yes.

**Q:** Fair enough. You can definitely provide that type of answer.

**A:** Okay. [Laughter]

**Q:** The next is heart failure.

**A:** So, same thing. Given a heart attack you get heart failure.

**Q:** High blood pressure.

**A:** I think some of the risk factors, I think maybe shared etiology, although essential hypertension I guess doesn’t have a known etiology at this point. I don’t think it would increase your risk.

**Q:** How about high cholesterol?

**A:** I think that would probably increase your risk of stroke rather than the reverse.

**Q:** So the other way around.

**A:** Yeah.

**Q:** Diabetes?

**A:** Same. So the reverse, pathophys.

**Q:** Headache.

**A:** Yes.

**Q:** Vertigo.

**A:** Yes, depending on where the silent stroke is.

**Q:** Seizures.

**A:** Yes, definitely.

**Q:** Falls.

**A:** Yes. That would be a silent stroke.

**Q:** Fair enough. Memory loss?

**A:** I guess together with the vertigo.

[Phone interruption?]

**Q:** Do you want to—I think we were at memory loss.

**A:** Yes, because vascular dementia is an entity. But again, it wouldn’t be a silent stroke, I guess if that was the case.

**Q:** And next is dementia.

**A:** Same.

**Q:** Anxiety?

**A:** Oh, yeah.

**Q:** Depression.

**A:** Yes.

**Q:** And last is schizophrenia.

**A:** No.

**Q:** How comfortable do you feel treating patients with silent strokes?

**A:** Very.

**Q:** And I think you’ve already answered this question but, I was going to ask you if follow any particular practice guidelines and if so, which ones?

**A:** So the secondary prevention guidelines, aspirin, statin for all strokes, unless it’s been hemorrhagic or has another, known etiology like A-fib, where it would be Coumadin instead of aspirin—and then the etiology of A-fib, so it is sort of tracing all the roots I guess. But for pure stroke, it could be aspirin, statin are my defaults.

**Q:** What do you think are the major knowledge gaps in this area?

**A:** I guess I’d like to prove that silent strokes are the same entity as non-silent strokes because presumably there is a proportional difference. Some of them are thrombotic and embolic just like big strokes. But there probably is a higher percentage that are just amyloidopathy or vascular, you know, leak or some other, not just pure atherosclerotic disease. The percentages probably differ which will impact the prognosis.

A lot of the data for the secondary prevention is based on the thrombotic and not even necessarily embolic because that’s going to be an alternate source. So treating everyone as if its thrombotic is probably inappropriate. And if the proportion is different for silent versus non-silent, that might affect the clinical decision.

Other things that I’d want to know? I guess that the classification and categorizing that you talk about before, you know, if it’s one millimeter is that different from one centimeter? Is one versus ten different, gray matter versus white matter versus brain stem, cerebellum. You know, there are other vascular regions that are different from others. So I feel like it is more just a big, black box as opposed to one or two questions I’ve got left to answer.

**Q:** Sure.

**A:** I feel like I don’t even know what questions to ask yet because I sort of lump it in empirically as silent and non-silent as the same. But I don't even know if that huge branch point is appropriate or not yet. So I feel that every aspect of it needs to be re-studied in the same way that symptomatic strokes have already been studied—not that all of our knowledge is complete about symptomatic strokes either. But if we at least get confirmation that they are the same for what is already known then I could extrapolate more comfortably to all the other aspects, too.

**Q:** This last part is a little bit of a thought experiment.

**A:** Okay.

**Q:** And the idea is to see how your practice might change given certain information from new studies.

**A:** So potential new studies--

**Q:** Potential new studies

**A:** --Not necessarily real studies. Okay.

**Q:** So let’s first imagine that there is a well-designed, rigorous, observational, comparative effectiveness study that is done performed to determine the effectiveness of prevention therapies after silent strokes. And let’s say the outcome is asymptomatic stroke, which we know is an outcome of silent stroke. Let’s say the study actually found the opposite of what your usual practice is right now. So maybe the risk of putting somebody on an aspirin actually outweighs the benefits. Would this type of study, in terms of its design and—would that be enough to convince you to change your practice?

**A:** Potentially. Observational, obviously, never gets to causation. So the whole concept of the study is a bit flawed. But if it was large enough, I am concerned that silent strokes are not concerned as regular strokes. And aspirin for an old person with leaky blood vessels could actually—it’s pretty easy to come up with a mechanism that that would be totally true. So, yeah, I probably would change a little bit of my practice. But for the young, healthy person who I was not worried about anything other than thrombotic disease, I probably would not change it because the causality of an observational study, just I wouldn’t trust it.

**Q:** Okay. Would you prefer like a randomized control trial then to try and answer those questions?

**A:** Hm-hmm. Yeah.

**Q:** If so, let’s say and RCT was performed looking at comparing these treatments, how do you think patients could be recruited into that type of study? What do you think would be the optimal way of doing that?

**A:** For an RCT?

**Q:** Yeah.

**A:** Well—you mean ethically or just like how would you sell it to the patient?

**Q:** All of the above.

**A:** I think once—I don't know if the standard of care for silent strokes is the same as for non-silent strokes. My practice is that I treat them the same but I don’t know that that’s, the body of literature supports that or not. I probably would be a little bit ethically—I would have a little trouble sort of telling someone not to take aspirin and statin when I found a stroke on their head CT. So I guess that is a good point to go back. I don't know if I will ever get the evidence that would help me.

I guess it would depend on—I’d have to be more knowledgeable I guess. So if it was unethical, if the current standard of care is to treat everyone with a silent stroke the same way with aspirin and statin, then you’ll never get an RCT. So you couldn’t definitely recruit at all. And I guess in which case you would be stuck with observational data.

You could try to demonstrate a dose response instead, baby aspirin versus full-dose aspirin, aspirin plus Plavix and try to demonstrate similar differences between symptomatic and non-symptomatic strokes. Yeah. I don’t think you could, I guess, if that’s truly the standard of care.

**Q:** Do you think it’s a problem that these are mostly discovered incidentally in terms of recruiting patients? Or do you think it’s a [00:35:27], it shows up on the scan and then somebody could tell them that it is there and then people could be approached to be recruited into the study?

**A:** Yeah. I don’t think it’s a problem because a lot of things are discovered that people showed up in the ER. They are recruited when they show up with a problem. This is just how the problem is discovered by X-ray as opposed to symptoms. It will obviously create any—whenever you’ve got a non-random selection of patients, you’ve got confounders that you would just have to account for. This is clearly a confounder. But it is probably relatively easy to control for I would assume.

**Q:** Okay. Are there particular outcomes that you would be most interested in, in this type of study?

**A:** I think all of the outcomes that you mentioned previously that I thought were associated, confirmation and not confirmation, so any repeat stroke would be first and foremost, what’s the most—what are the percentages? What’s the timeframe, you know, a repeat stroke within a year, two years? Are you out of the window after ten years if you haven’t had anything else? Do you ever get to revert back to baseline? If it’s embolic versus thrombotic does that matter if you controlled for the embolic risk in the first place?

So I think the outcome would matter on the etiology that you discovered. But, yeah, the risk of recurrence would probably be my first personal thought but all those other ones, seizures, intracranial hemorrhage, all the important--

**Q:** Let me give you a few statistics. So there have been a number of large, cohort studies like the Framingham Heart Study, the Rotterdam Scan Study, the Cardiovascular Study, and so on—and that for the most part have looked at individuals over age 50. And pooling some of that data together looks like the prevalence of silent brain infarcts for people over age 50 in these screen populations is about 20 percent.

**A:** Wow!

**Q:** And so we also know, based on these studies that silent strokes are associated with about a two to fourfold increase in the risk of symptomatic stroke as well as the risk of developing dementia for the types that have some vascular component, whether it’s vascular dementia, Alzheimer's, or a mix between the two.

**A:** Okay.

**Q:** So knowing that, if we were to find even just a sort of modestly effective prevention strategy, do you think that it would actually be worth starting some sort of screening program for silent strokes?

**A:** Yes.

**Q:** Even if it’s that prevalent, so 20 percent or so?

**A:** That would be the reason for it I think. Because, you know, breast cancer is probably one of the most talked about screening strategies people have and that’s a one in nine lifetime risk for people. This is one in five.

**Q:** Right.

**A:** So, yeah, hands down that would be--

**Q:** How would you do that? Or how would you imagine seeing that happen?

**A:** I guess it would get to the sensitivity and specificity of our current imaging technology. It would have to be CT. It would have to be MRI. It would have to be a cost – benefit analysis. You’d have to look at sort of the contrast versus non-contrast risk of allergies. I mean it would be difficult to say the least because screening, I don't know how many of our 330 million Americans are over the age of 50 but it’s got to be a pretty high percentage. So you’re talking about tens if not a hundred million people in this. So you would have to be pretty practical about how this is going to get done.

**Q:** Yeah.

**A:** And it might be like a colonoscopy which is also starting over 50. Mammogram is over 50. And a colonoscopy is a much more involved test that we still recommend for everyone. Uptake is obviously not 100 percent. So it doesn’t have the same effect on the system. But this would be—if, you know, truly it’s 20, if it’s one in five, that increases your risk by two to fourfold, even a modest decreased risk presumably has the same—presumably you’re treating atherosclerotic disease.

And so you would also decrease their heart attacks, vascular dementia, you know, symptomatic strokes. And so you would have a potentially huge quality of life increase from a modest atherosclerotic burden change—that would be far and away one of the most beneficial impacts we could have. So it’s hard to be more emphatic than that I guess.

[Laughter]

**Q:** Fair enough.

**A:** I think it just comes down to the practicalities of it. Someone has just got to work the math and say there are only so many CT scanners in the country. If they worked 24 hours a day, seven days a week, we can get X-number per year. Therefore we can only do screening once every decade. Or is this a one-off between 50 and 60 and never again, just to establish a baseline risk. Lower threshold for testing, you know, anyone with hypertension we do this for, like you could maybe sort of select the high risk of the high risk, you know, not just over 50 but high risk, over-50 men, hypertensive, previous heart attack, you know, other evidence of atherosclerotic disease also look for stroke—or who cares if they already have atherosclerotic disease they should be secondary prevention for stroke, [by rote][?].

So you could fine tune it. But the concept and philosophy—I’m a primary care doctors so screening is sort of my life blood.

**Q:** Right.

**A:** And this is one of the most damaging, mentally and physically, diseases that exists. So--

**Q:** Fair enough. So that’s actually the end. I just wanted to see if you have any additional sort of questions or thoughts--

**A:** Okay.

**Q:** We’ve come to the end of the interview. Anything else that you want to express on the--

**A:** No, it’s great.

**Q:** --Recording?

**A:** No. Thanks. Well done.

**Q:** Great. Thank you. I’m just going to start the recording right here.

END OF INTERVIEW

Internist 4

Study ID: IN4

**Q:** So this is the interview with our fourth internist. I’ll ask you a number of different questions. And actually some of them will have a few sub-questions as well. But they are generally pretty open ended and you can answer in as much detail as you like. So the first question is really just very exploratory but just tell me what you know so far about silent strokes, whatever comes to mind.

**A:** So, that’s a good question, what do I know about them. They are usually subclinical, assuming because they are silent. Right? People usually don’t have symptoms with them. And we usually pick them up on imaging. And I guess that’s all I know.

**Q:** Okay. Fair enough.

**A:** Is that okay?

**Q:** Yeah, of course. The number, actually, in the literature and also in how clinicians describe them to each other and also to patients, there have been a number of terms to describe this. So the diagnosis has a number of different names, which could include silent stroke, silent brain infarction, silent cerebral infarction, covert stroke, covert brain infarction, subclinical stroke, subtle stroke, asymptomatic stroke. And then there are a bunch more.

**A:** Yeah.

**Q:** But of these, which have you heard used?

**A:** Asymptomatic stroke probably most. I haven’t heard silent stroke as much until you said it.

**Q:** Fair enough. That’s fine.

**A:** Are there other terms that you’ve heard used besides the ones that I mentioned?

**Q:** No.

**A:** Okay.

**Q:** And of these, if you were to use it to describe this phenomenon, in terms of terms that you think might be most useful or most appropriate, which one would you pick?

**A:** Silent stroke. Seems reasonable. Okay.

**Q:** Okay. Why do you think these occur?

**A:** Generally they, in my practice, occur in patients who have cardiovascular risk factors. And, you know, it’s interesting. I think the majority of them likely are thrombotic, from vascular disease. But some people may have A-fib and embolic strokes as well that are silent. So I think probably both causes. I mean hemorrhagic, probably not as much because, yeah, I haven’t really seen that as much. But I’m assuming they’re silent—I mean I’m assuming they are just in a part of the brain where it is not as obvious because there is a large part of the brain we don’t use, as you know.

[Laughter]

You should know. So I just assume people just go asymptomatically. A little piece of the brain is infarcted. And it is mainly in my experience, patients who have, you know, cardiovascular risk factors and it just happens.

**Q:** In your opinion, in what ways are the pathophysiology of these asymptomatic or silent strokes similar or different to your garden variety, symptomatic stroke?

**A:** I would think they’re more small vessel, right, event versus like a large infarct that becomes symptomatic.

**Q:** And I think you kind of answered this next question already but I was going to ask you if you think there are different types of silent strokes--

**A:** You mean embolic versus--

**Q:** --If you categorize them in particular ways.

**A:** I don’t really—yeah, is that what you mean?

**Q:** Hm-hmm.

**A:** Thrombotic and embolic?

**Q:** However like you would kind of operationally break them into categories that would be helpful for you.

**A:** Yeah, that’s how I would do it.

**Q:** So thrombotic versus embolic.

**A:** Embolic, yeah.

**Q:** Okay. And you kind of alluded to this before we started the interview. But do you think that silent strokes and white matter disease, so microangiopathic changes, are they similar or different? And if so, how so?

**A:** So they are not—so I don’t read my own MRIs, right?

**Q:** Sure.

**A:** I depend on somebody else reading them. And so they are called different things. Someone who reads the MRIs or CT scans categorizes them differently. So I am to think they’re different. However, if you really think about it, they’re really not different because they are both, you know, low flow events, right. So I guess not.

**Q:** Okay. Fair enough. Does reading about one on an imaging report change how [simultaneous conversation]

**A:** Yeah. So I have to say the micro and the chronic white matter changes are so common that I don’t really—I mean, I make sure that their cardiovascular risk for a file [?] is, you know appropriated addressed, which they are because that’s what we do even without a stroke, right?

**Q:** Of course.

**A:** But beyond that I don’t really get too excited by them, versus if somebody called a real, like said stroke, acute, sub—I’d be like, “Oh, wow! They had a stroke, which maybe, you know, we should think about it more.”

**Q:** Do you think they have the same significance or different significance, the white matter disease versus--

**A:** Right. Right. Right. I would think if it’s called a stroke it would be more significant.

**Q:** Okay. What do you think are the major risk factors for silent strokes?

**A:** Age, diabetes, hypertension, tobacco, hypercholesterolemia and I guess—yeah, I mean that’s what—then you have the more rare, like vasculitis, people with, you know, but that’s probably not as major.

**Q:** And we often find these by accident, right?

**A:** Hm-hmm.

**Q:** What are the types of scenarios or setting in which these show up?

**A:** By accident? Headache. You know, people get—it’s usually from imaging. And most of the time the imaging is done, probably for headache or trauma, where they are found. Or memory, or if it’s a dementia workup, maybe—those are most of the time where you find them, if you’re not looking. You’re asking if you’re not looking specifically--

**Q:** Right.

**A:** --For a stroke. Yeah. Yeah.

**Q:** Although maybe you are sometimes.

**A:** Yeah, maybe I am sometimes like with the dementia. Right? Or a change in a symptom. But I may not be thinking about it.

**Q:** Okay. When you do find these or see the radiologist’s report that somebody’s had and old infarct or a subacute infarct, do you end up drawing some connections between that finding and whatever the initial symptom was, the presenting symptoms?

**A:** Usually not. But again, it would have to be like an individual, like maybe have to be an individual, case-by-case, you know, issue.

**Q:** Right. So thinking a little bit about how these are found, so we often use this term incidental in reference to things that we find on tests, on scans that are detected when we are investigating something else. And so this often comes up as a surprise. So I’m sure as a primary care doctor you see a lot of incidental findings. What does it mean for you for something to be incidental in terms of how you use that terms and how you may or may not respond to things.

**A:** I guess with incidental, means I think more like scarring, lung nodules, things like that. I would—with a stroke, if somebody called it a stroke on scan, I wouldn’t actually call it incidental. I mean the microangio—so, again, this white matter disease it kind of probably think of it more like I think of incidental stuff, like the adrenal adenoma or these things that just are there and everybody has. But with the white matter disease it’s almost become like incidental because a lot of people have them. Although, I have to say, even that it’s case-by-case.

**Q:** Sure.

**A:** Like I have had a young woman who had microangiopathic changes reported, youngish in her forties. And I took that a lot more seriously, right, the maybe your old man or woman or whatever, someone who is older. So age does make a difference in how I think about it--

**Q:** Contextualize it.

**A:** --Contextualize it, yeah. But anyway, back to incidental, yeah, I wouldn’t—I mean is somebody reported like an infarct, subacute or acute—well, subacute I’d be more concerned, like a chronic, old, infarct, acute also obviously, I take it much more seriously. Old infarct, I would consider it and take action if I see that. Yeah.

**Q:** Just to get you to explore that a little bit more out loud, why would it matter to you more if something is acute or subacute versus [simultaneous conversation]

**A:** Oh, just because I feel like if it’s acute then we always wonder at what stage, with a stroke, is it still going to be more, is there still going to be another one, right, if this is something that is happening. If it’s chronic, if it looks really old, it’s probably not related to what we’re doing, what the complaint is, right? And it may not be as urgent to get an evaluation done, is what I would say.

**Q:** When you see the reporting of an infarct that doesn’t seem to have any symptoms with it, do you feel obligated to respond to that?

**A:** Yes.

**Q:** And if so, how? Like what sort of things do you [simultaneous conversation]

**A:** Again, it depends. It depends on the chronicity. I would probably like get neurology involved if it was like acute, or get them in the ER.

**Q:** Sure. Sure. Okay.

**A:** And subacute, even, I would be like, we should do a work up. If it’s an old stroke I have to admit, you know, I would check their labs and do like a lab workup, maybe an ECHO, maybe put a monitor on them. But, yeah—but more, maybe myself--

**Q:** Sure. Which labs?

**A:** I would do CBCE, lights, BUN, creatinine, lipid profile, A1c, obviously. And yeah, just the standard vascular. I know sometimes you do ESR and CRP. I don’t usually. [Laughter]

**Q:** Some people do.

**A:** I know. I know. You people used to do. I don’t

**Q:** Fair enough. And then I’ll ask you a little bit about the ECHO cardiography and the event monitoring but what type of ECHO cardiography?

**A:** If it’s a stroke we usually do and ECHO with bubble study. And do you want to know about the monitoring?

**Q:** Yeah. Yeah.

**A:** Okay.

**Q:** Just because there are a lot of different ones.

**A:** I know. Usually like a Holter but I’ve started to do, if I’m really suspicious, the 30-day event. So.

**Q:** Is there anything in particular that would make you more suspicious?

**A:** More suspicious? [Laughter]

**Q:** [Laughter]

**A:** I think they are really having strokes or they are having A-fib or we can’t explain their stroke. I know I sound really flaky. I probably would send them to neurology, too.

**Q:** I can assure you neurologists are just as flaky.

[Laughter]

**A:** But I’m just trying to—I’m trying to think of specific examples, but again, these are not necessarily like silent strokes. These are—well, they are like question strokes, well, question TIAs, is I guess I should say. You know, I guess it depends on my suspicion that it is that or not. I’ve generally just done the 48-hour Holter generally, if I’m just doing it. Yeah.

**Q:** Do you modify any treatments?

**A:** I put them on aspirin and then make sure they’re on a statin, make sure their blood pressure is controlled. And I think that is all I would do.

**Q:** Do you talk to people about any behavioral changes or life style modifications?

**A:** Yes. But like I talk to people about that all the time [laughter] regardless if there is a stroke. But, yeah, I mean--

**Q:** I was just wondering if this end up being a trigger for them.

**A:** A trigger to do that. Yeah, I would think so. Yeah. Yeah.

**Q:** Okay. What sort of things?

**A:** Exercise, smoking cessation, making sure that their weight is, you know, appropriate weight control, and—yeah.

**Q:** Okay. Do you think that these asymptomatic or silent strokes, have they caused direct harm to patients?

**A:** Some.

[Laughter]

But most no because they are silent.

**Q:** You mentioned they are kind of, affecting a part of the brain that we’re not really using.

**A:** Correct. If they don’t it’s silent, they probably haven’t caused direct harm.

**Q:** When you say some, what do you mean by that?

**A:** Well, I have like a—I have a patient with some memory loss. And so it’s mainly it’s the memory stuff, like multi-infarct dementia.

**Q:** Sure.

**A:** And vascular dementia that sometimes I think is related to their strokes.

**Q:** So you’re inferring that--

**A:** That is a direct--

**Q:** --If there is an accrual of silent strokes, it might be leading to their cognitive--

**A:** Their cognitive dysfunction. Exactly.

**Q:** Fair enough. Do you think that—well, actually, let me frame it this way. From your perspective, so what’s the—how much risk, at risk do silent strokes put your patients? Like is it a small amount of risk? Is it a large amount of risk?

**A:** Risk for what?

**Q:** Health consequences?

**A:** Consequences? Yeah. I would say it’s a large amount of risk.

**Q:** Okay.

**A:** Yeah, I mean I would put them in the bucket of people who now have ASCVD, right? They’d be in that risk category, high risk or with disease.

**Q:** Sure.

**A:** Like they are not even at risk. They have a disease now.

**Q:** Sure. You say ASCVD, you mean--?

**A:** Atherosclerotic cardiovascular disease, like when we do our lipid profile and talk about risks to patients, is the 10-year risk of ASCVD, right? But they have it. It’s not even a risk.

**Q:** Right.

**A:** They’ve got the disease now. So I’d say high risk for that.

**Q:** Okay. So I think you have already kind of answered the next question that I was going to ask you, what sort of things do silent strokes put your patients at risk for. So you kind of mentioned atherosclerosis.

**A:** Atherosclerotic disease, heart disease, dementia, falls, gait disturbances, you know, cognitive decline, memory, all that stuff.

**Q:** Fair enough. I’m going to explore that question just a little bit more. I’m actually going to give you a little bit of a laundry list of different conditions, symptoms, other things. And to frame-up word [?] of the questions, do you think silent strokes put your patients at risk for any of the following.

**A:** And then I have to say yes to all of that.

**Q:** You can say yes, no or give some qualified answer.

**A:** Maybe. Okay.

**Q:** The first one is stroke.

**A:** Yes.

**Q:** The second is intracerebral hemorrhage.

**A:** Maybe.

**Q:** The third is myocardial infarction.

**A:** Yes.

**Q:** The next is heart failure.

**A:** Yes.

**Q:** The next is high blood pressure.

**A:** Yes.

**Q:** The next is high cholesterol.

**A:** Yes.

**Q:** Diabetes.

**A:** Yes.

**Q:** Headache.

**A:** Yes.

**Q:** Vertigo.

**A:** Yes.

**Q:** Seizures.

**A:** Yes.

**Q:** Falls.

**A:** Yes.

**Q:** Memory loss.

**A:** Yes.

**Q:** Dementia.

**A:** Yes.

**Q:** Anxiety.

**A:** Maybe.

**Q:** Depression.

**A:** Yes.

**Q:** Schizophrenia.

**A:** No.

**Q:** Okay.

[Laughter]

Fair enough. So you weren’t just saying yes to the whole thing.

**A:** I know. I don’t think so. [Laughter]

**Q:** Fair enough.

**A:** I know, right? I was listening.

**Q:** How comfortable do you feel treating patients with silent strokes?

**A:** Silent strokes?

**Q:** Yeah or asymptomatic strokes.

**A:** Pretty comfortable.

**Q:** Okay. Are there any particular practice guidelines that you follow? And if so, which ones?

**A:** No.

**Q:** You just kind of go based off of your experience or--

**A:** Yeah. Yeah.

**Q:** What do you think are the major knowledge gaps, the things that you would want to know about that would help you?

**A:** With silent strokes?

**Q:** Yeah.

**A:** Well, I think I would—the workup, a little bit more, even though I don’t follow a practice guideline that I can think of. I probably do because I look them up. But I couldn’t even think of them. What would be the knowledge gaps? I mean I’m not—like when people—so I guess I’m trying to think if a person—it depends, right? It depends. Like why do I send people with actual strokes to a neurologist, right, versus why I would manage someone with a silent stroke, which is an actual stroke?

And probably because it is not symptomatic I may not be as—I would think it’s okay for me to manage it. But I guess it would be good to get a review of just the management guidelines. Yeah.

**Q:** Do you talk to your patients about these, like when you find them?

**A:** Yes.

**Q:** How do you frame that conversation with them?

**A:** I probably—I would say I probably tell them, and again I can’t, I wish I remember like a specific example. But in general it’s similar to when we get a silent MI we pick up like Q waves or, you know, I would say you, “It looks like you has a stroke at some point in the past. And we need to make sure that another one doesn’t happen. And we need to manage all of the issues, we need to treat you to prevent another one.”

**Q:** Do you recall any of the responses from your patients in terms of how, like what questions they asked or how they reacted to that news?

**A:** I don’t. But I think most people, nothing super stressful. Most, you know, probably just have the usual questions about how did it happen, you know, what does it mean going forward. But I can’t remember anything specific.

**Q:** So we’re coming close to the end, actually.

**A:** Oh, good.

**Q:** But the last part is, it’s actually a little bit of a thought experiment.

**A:** Okay. Great.

**Q:** The idea is to see how your practice might change given some information from new studies--

**A:** Oh, great.

**Q:** --Which haven’t done yet.

**A:** Oh!

[Laughter]

**Q:** But some of this information is actually real information. So the first part is like, let’s imagine as a type of study, that there is a sort of large, rigorous observational, comparative effectiveness study that was looking at the effectiveness of prevention therapies after someone is discovered to have an asymptomatic stroke. And let’s say the idea is to try and prevent a symptomatic stroke from happening in the near future.

**A:** Correct.

**Q:** If the study found the opposite of what you might normally do, let’s say you often start patients on an aspirin and it turns out that the risks of aspirin, GI hemorrhage or something, intracerebral hemorrhage, actually outweighs the benefits of preventing ischemic stroke. Would that type of study or evidence be enough to alter your decision or alter your practice?

**A:** Yes.

**Q:** Okay. Do you think you would need, like would you need to go, need other things like a randomized trial or other things to sort of bolster that information or do think sort of a good, comparative effectiveness study would be sufficient?

**A:** I think it would be sufficient.

**Q:** Okay. Fair enough. What do you think would be the important outcomes to look for?

**A:** For--?

**Q:** For sort of a prevention study, where somebody—where we’re looking at people who have silent strokes.

**A:** Right. I see, like outcomes of whether it would be like—that’s a good question. [Laughter] It’s a really good question. I mean subsequent stroke, you know, obviously death is like another outcome. But subsequent stroke, quality of life, you know, markers would be a good outcome to look at, falls, dementia, things like that.

**Q:** Okay. I’m going to give you a few known statistics. So there have been several large, cohort studies mostly looking at individuals over age 50. And there have been some aggregation of this data. This is from the Framingham Heart Study, the Cardiovascular Health Study, the Northern Manhattan Study, Rotterdam, ERIC, and so on. And they’ve found that the prevalence of silent brain infarcts is about 20 percent in people over age 50. And it rises with age. So Framingham is somewhere right in the middle.

**A:** How do they define silent infarct?

**Q:** No prior history--

**A:** And then seeing—did they count the white matter changes?

**Q:** They actually used an imaging definition where they found sort of discrete lesions that were generally greater than 3 mm. That was their size cutoff. And then, especially if it was MRI, they would look at different sequences to see like if there sort of irregular edge, is there volume loss in it. And so, of course, like the numbers start lower at the younger ages. So in the 50- 60 range, it is more like 7.5 percent. Go a decade later it is like 15 percent. A decade later it’s 20, 25 percent and it keeps increasing.

So there’s that. Another thing that we know is that in these studies, which actually were a screened populations, they just did these MRIs at fixed time intervals. They found that silent strokes were associated with a two to fourfold increase in symptomatic stroke and a two to fourfold increase, or two to threefold increase in dementia, which includes Alzheimer's, vascular and vascular Alzheimer's mixed dementia.

So with that information about outcomes and also the general prevalence, if we were to have, to figure out with some studies, some sort of effective prevention strategy for let’s say stroke or even cognitive decline, knowing that there are that many people, do you think it worthwhile implementing some type of screening strategy?

**A:** For stroke, screening for stroke?

**Q:** For silent stroke.

**A:** For silent stroke? Wow! That’s a good question. Yeah, I would think so.

**Q:** How would you do that?

**A:** How would you do that? You’d have to do an MRI, right? You’d have to—I mean obviously it hasn’t been studied, but I think you’d have to do an MR, some kind of—I mean it would have to be a CT or an MR but I don’t think CT is very good at picking up small strokes, right?

**Q:** Right.

**A:** And so you’d probably have to do like a baseline MR at 50 and then see what happens and then go from there. But it’s interesting question.

**Q:** I guess MRIs are pretty expensive.

**A:** Yes! The MRIs are pretty expensive. It’s a huge number you are talking about.

**Q:** Yeah.

**A:** Yeah. It could be like lung cancer screening. You’d have to maybe look at, you know, criteria. Maybe screening people with, you know, cardiovascular risk greater than 10 or something like that would be another way to do it. But, yeah, I think that might be an interesting, a reasonable thing.

**Q:** Like identifying a high risk group.

**A:** A high risk group and then screen. Yeah.

**Q:** Great. So that’s actually it. You went really fast.

**A:** My God. I didn’t really--

**Q:** Do you have any additional thoughts or questions?

**A:** No, it’s really interesting. It’s great. It’s great.

**Q:** Well, I’ll end the recording right here.

**A:** Okay.

**Q:** I’m sorry, what were you saying about lacunar strokes?

[Laughter]

**A:** Sorry I categorized lacunar strokes and I didn’t really discuss them. But I think of them as—I mean I don’t think of them as different. But I guess I think like lacunar strokes are so small, right, usually associated with hypertension. And then the infarcts that are bigger I categorize differently.

**Q:** Okay.

**A:** That’s all.

**Q:** So do you pay attention to how the radiologist describe the infarcts by size?

**A:** Yeah.

**Q:** Okay.

**A:** And they call it—they call lacunar infarcts separately from other infarcts.

**Q:** They do. They will often, at least here they will often add lacunar as a--

**A:** Correct.

**Q:** --Adjective in front of infract.

**A:** Correct.

**Q:** But for you that, that may have a difference in terms of your aggressiveness of investigations or treatments?

**A:** Yeah. Because lacunar infarcts are rather common.

**Q:** They are.

[Laughter]

**A:** Just to let you know. [Laughter]

**Q:** [Laughter] Yeah, I know.

**A:** They are next to white matter changes.

**Q:** Sure.

**A:** Yeah.

**Q:** Can I ask you what you would do differently?

**A:** With lacunar?

**Q:** With like medications, referrals--

**A:** I wouldn’t do anything differently.

**Q:** --Testing?

**A:** No, I wouldn’t do anything differently. It’s just I was thinking about it, that there may be even more than ten, then.

**Q:** Oh, in terms of how many you see.

**A:** How many people I’ve seen. It’s probably still ten. I’m sorry. It’s so hard to guesstimate.

**Q:** No. No. No. That’s--

**A:** Yeah. Yeah. Sorry.

END OF INTERVIEW

Vascular Neurologist 3

Study ID: VN3

**Q:** So this is our interview our third vascular neurologist. And so the first question is just very general, just tell me what you know about silent or covert strokes so far, anything you would like to say about it.

**A:** Silence. What do I know about them? Well, I know that they are very common but I forgot the exact number. Boy! I mean what do you want me to say?

**Q:** Whatever comes to mind, whatever you think is important.

**A:** Well, I think they are under-studied and I think they are underestimated in terms of probably their role in predicting recurrent stroke and maybe even cognitive functions and stuff like that. I think, obviously, the more you do MRIs on people the more you are discovering that they are more prevalent than we knew or than what we thought. That’s really all I can say about them. The majority of them tend to be cerebella, lacunar, or in the right hemisphere.

[Laughter]

**Q:** Okay. I might need you just to speak just a tiny, little bit louder. I’m going to put the microphone as close to you as possible to make sure it is picking up your voice just because your voice is a little bit softer probably from talking to a lot of patients today. So this is a springboard from that question but there are a lot of terms that people have used in the clinical literature or used to describe these to colleagues or to patients. So some names that have been used include silent stroke, silent brain infarction, silent cerebral infarction, covert stroke, covert brain infarction, subclinical stroke, subtle stroke and asymptomatic stroke. So of these, which are terms that you’ve heard used?

**A:** Well, I heard of all of these terms.

**Q:** Which do you use most often?

**A:** Well, I usually say it’s a stroke that you didn’t know about, that you didn’t notice, because I really don’t know that silent. I don't know if it is asymptomatic. I mean it could be something very subtle and you noticed it but you didn’t pay attention to it. You just let it go.

**Q:** Okay.

**A:** Maybe clinically unrecognized.

**Q:** Okay. Clinically unrecognized stroke. Okay. Are there other terms besides the ones that you mentioned or the ones that I’ve mentioned or the ones that I’ve mentioned that you’ve heard people use?

**A:** Kaplan has a term for it but I can’t remember what it was.

**Q:** I think it is unexpected stroke.

**A:** Unexpected?

**Q:** Yeah.

**A:** Okay. Not a very perfect definition either but--

**Q:** Of these, whether it’s the term that you used or others, is there a term that you think is most appropriate or most useful, whether it’s describing it in the literature or describing it to patients?

**A:** I told you the term that I like to use it, just clinically undetectable or unrecognized.

**Q:** Clinically unrecognized.

**A:** Yeah, clinically unrecognized.

**Q:** Sure. So why do you think covert strokes occur.

**A:** Why do I think they occur? What do you mean? They occur just like any other stroke. It just happened to be. It’s like a TIA.

[Laughter]

So it’s the same thing. It’s just a different severity, I guess and different manifestation. It depends on the location of the stroke, too.

**Q:** So you think that they are unrecognized because of a lower severity.

**A:** Yeah. I think location and lower severity. I think yes. And part of it, I mean if you’re talking, digging into it and look at the patients, it might have something to do with the patients, too, maybe the level of sophistication, maybe level of education and kind of life style in general. So that might be a factor in there, too.

**Q:** What do you mean by life style?

**A:** Well, I mean if you’re drinking all the time and you are a little bit slurred and a little bit clumsy--

**Q:** So that might impair the recognition of the [simultaneous conversation]

**A:** Something subtle like that you just blame it on--

**Q:** Okay. Fair enough. No, thank you for making that a little bit more explicit. So that was, you mentioned impaired recognition. You mentioned severity. You also mentioned location. And I think previously you had mentioned that you thought that these might cerebella or right hemisphere or lacunar. Is the idea that you think that these are--

**A:** And of course, frontal.

**Q:** --The parts of the brain that are less likely to produce symptoms or produce deficits?

**A:** Yeah. Or if they produce deficits, they are not the ones that are actually easy to detect.

**Q:** Okay. So if you think that these are—this is a similar question but worded slightly differently. In your perspective are there ways in which covert strokes are similar or different in terms of their pathophysiology as compared to more typical ischemic strokes that--

**A:** No. I don’t think so.

**Q:** You think they’re the same?

**A:** I think they are probably the same.

**Q:** Okay. Do you think there are any differences in terms of risk factors or--?

**A:** Probably not.

**Q:** Okay.

**A:** The only thing I can think of that might be a little bit different is that these little white things you see in migraine patients, whether they are infarcts or not infarcts. So that might be the one that is a little bit, the pathophysiology is a little bit different.

**Q:** What do you think those are?

**A:** I think they are probably little infarcts.

**Q:** Okay. So would that lead you to think that migraine has a differential risk between producing covert lesions or covert strokes as opposed to more clinically apparent strokes?

**A:** Probably. Yeah, probably.

**Q:** Do you think there are any other risk factors that are more likely to produce a covert stroke as opposed to a less clinically apparent or one that is recognized?

**A:** I’m guessing here. I’m not sure.

**Q:** That’s fine.

**A:** Maybe antiphospholipid antibody syndrome. You see a lot more on the imaging than clinically sometimes.

**Q:** Okay.

**A:** That’s a guess. I don’t have anything to support this. [Laughter]

**Q:** Okay. Fair enough. Let me check the sound. Okay. So just to sort of make it more it more explicit, it sounds like you think that the risk factors are essentially the same. And so what would you think are the major risk factors for covert strokes or silent strokes?

**A:** Well, I mean it’s the same thing, smoking, hypertension, hyperlipidemia, something cardiac. I think these are probably the main things.

**Q:** Okay. Moving to a different topic, what are the settings or the scenarios in which you have encountered people with this diagnosis or with these findings on scans?

**A:** Well, I mean we just talked about that. So one of them is that they just get imaged for whatever reason and they find the strokes. So the common thing is that they get imaged for headaches. That they are light headed, they get imaged. But the other really is they are just imaged because they actually have a stroke and then you find that they have an old stroke that they didn’t know about. I think these are the commonest scenarios that I see.

**Q:** My guess is as a stroke neurologist, when you see the old stroke or the covert stroke in the setting of one that brought someone to the hospital and brought them to medical attention, my guess is that you think of those as being part of the same disease process or at least being somehow related.

**A:** Well, not necessarily. I mean sometimes they just help you to explain the disease process but sometimes they don’t. So It could be a different mechanism; not always.

**Q:** Okay. In other scenarios, if you see these patients as a consultant when they’ve received a scan for lightheadedness or headache, do you end up connecting the finding to the scans? Or do you think that they’re completely separate?

**A:** No. Most of the time it has nothing to do with the scan. So most of the time it is really incidental. Because most of the time the imaging is really for a headache or lightheaded.

**Q:** Sure. So just exploring that idea of something being incidental a bit more, what does it mean for you for something to be incidental?

**A:** Well, that you can’t explain it by the location and the size of the infarct when you talk about the silent strokes or a symptom that you think, based on your knowledge I guess that it just, it is not something that this lesion would cause.

**Q:** Sure. When you encounter these, even as incidental findings, do you feel an obligation to respond to them?

**A:** So, yeah. Most of the time, especially like in someone—I mean most of the time I really—it depends on what they look like, if they look like they’re embolic, that I do respond to them. I look at their heart and I do the work up for a stroke basically, no matter how old it looks.

**Q:** Any particular tests?

**A:** So I would look for A-fib. I would do an ECHO. I would do the vascular studies if they were not done. I really try to understand why they had the stroke.

**Q:** Kind of building a little bit on that. So you mentioned atrial fibrillation. How far would you take that investigation knowing that we now have multiple different methods of doing cardiac rhythm monitoring, anything from an EKG to a 24-hour Holter monitor to 30 days of surface monitoring to an implantable cardiac monitor?

**A:** I do the 30 days surface monitoring. That’s what I do most of the time. Then the implantable I only push it if I really have very high suspicion for it. But if someone has really and old stroke, it looks like years ago, I probably, I’m satisfied with just the 30-day that shows you that there is nothing.

**Q:** Okay. Are there any other scenarios besides if somebody had a cardioembolic [simultaneous conversation]

**A:** No, I mean I mostly look for the things that you probably can treat to prevent another one. So I mean it depends really on kind of what they look like, the location, your overall impression. In someone young maybe there is a suspicion for drug use. Maybe I don’t push it too hard. So it depends.

**Q:** What sort of treatment changes might you implement if [simultaneous conversation]

**A:** So anticoagulation would be—what do you mean, without doing the work up or just based on--?

**Q:** With doing the work-up, yeah, if you find some things.

**A:** So, yeah, I mean I’m looking. I think the big question is, does this person need to be anticoagulated or not. So that’s I think the main thing. Because other than that, most of the time I just tell them, “Take a baby aspirin,” whether it helps or not but that’s what I tell them.

**Q:** Okay. Besides echocardiography and cardiac rhythm monitoring, are there any other tests that you might do if you encountered an incidentally found stroke?

**A:** Again, I mean nothing routinely. It really depends on kind of the specifics of the case on how I want to, how hard I want to look into it and how hard I want to push it. But I’m less aggressive than when someone comes with an acute stroke and it’s cryptogenic and you want to figure out why it happened.

**Q:** So why are you less aggressive in this setting than somebody who presents with a symptomatic stroke?

**A:** Because sometimes it really gets a little bit complicated when you ask them about the history. So they’ll tell you, “Oh, when I was little I fell off the tree and I was in a coma for a”—so you start not really sure. I this just like a scar from a trauma? Is this really an old infarct? As I said, a lot of them you will figure out some period in time in their life when they were doing drugs. So I feel like almost kind of my own way of doing kind of cost effective treatment. So if I don’t know, I do kind of the common things.

But if it is something right in front of my eyes and it’s all brand new and I’m trying to figure out then, yes, it is more aggressive. It may not be the right way to do it but that’s my own kind of way of doing it.

**Q:** Do you ever get any other specialists involved during the investigations or the management of patients with these conditions?

**A:** So like what? So cardiologists, of course, might get involved. Sometimes hematology but not really a lot. I find their input confusing.

**Q:** [Laughter] Okay.

**A:** :[Laughter] You didn’t say who I was, right? [Laughter]

**Q:** This is anonymous. [Laughter]

**A:** Good. No. Not really. I mean I think that’s probably about it. We don’t see a lot of young people to say I should get someone from pediatrics to get their opinion on them.

**Q:** Do you ever recommend any life style changes, behavioral modifications after these are discovered?

**A:** Yeah. I mean of course, sleep apnea, weight loss, if there are any risk factors, you ask them to kind of optimize it. Smoking. I mean you do the same thing like you do for the regular stroke.

**Q:** Do you think these covert strokes have caused some kind of harm to the patient?

**A:** Well, that’s what I was saying. They probably do. I mean I think with our little exam, you really don’t catch on these things. I mean they might cause some subtle, cognitive problems that you don’t know, you don’t realize. But for the person, maybe at work they are not as sharp as they could have been. Maybe as students they are not as sharp as they could have been. So I think, yeah, they probably cause something but not physically kind of evident. But if you do [00:16:28], probably psych testing on them you probably will find that they are not 100 percent normal.

**Q:** So cognitive is the big thing in terms of direct effects--

**A:** Yes.

**Q:** --That these lesions might have on somebody’s function.

**A:** And sometimes they have good effect. Sometimes they have good benefit because people really realize they had a stroke. And if they have some risk factors or if there are life style changes that can be done, so it’s almost like a wake-up call. They really realize that, well, at least I’m still intact. So I better--

**Q:** So you think it can be a motivating factor when it’s discovered--

**A:** Yes, sometimes

**Q:** --To help people become healthier.

**A:** Yes.

**Q:** Okay. Moving on from direct harm, in your perspective do you think that covert strokes put patients at risk for other health issues? And if so, how much risk do they put them at?

**A:** For other health issues.

**Q:** Yeah.

**A:** I don't know the answer to that. I would think they are probably at higher risk for having another stroke. But I don't know if—I mean and probably just having strokes, that could increase the risk for having sleep apnea. So, yeah, I guess probably in general it could, I guess, same thing like a stroke, yeah.

**Q:** In terms of magnitude for these things, do you think it’s a big risk, a small risk, somewhere in between?

**A:** You know, I really don’t know the exact answer because that’s the problem. I don’t think we follow them closely enough to know, kind of the other problems that they run into. And we just focused on the stroke and that’s it. So I’m not sure. I don't know. I don’t see why it would be different.

**Q:** So as an extension of that question, thinking about what silent strokes or unrecognized strokes--

**A:** Covert.

[Laughter]

**Q:** Covert is supposed to get a the idea of things being unrecognized. But thinking about what they put people at risk for, I’m actually going to give you essentially a laundry list of different conditions or symptoms. And I want you just to tell me if you think, yes, covert strokes might put patients at a risk of these, no, or you can give some qualified answer in between that.

**A:** Okay.

**Q:** I’ll start with, again, the question is do you think covert strokes place your patients at risk for any of the following. So the first is stroke.

**A:** Yes.

**Q:** The second is intracerebral hemorrhage.

**A:** Well, it depends on the etiology I guess for them. Maybe.

**Q:** The third is heart attack.

**A:** Yes.

**Q:** The next is high blood pressure.

**A:** Yes.

**Q:** High cholesterol.

**A:** Well, that’s a tricky question. So are you trying to say that the stroke is the reason why they develop hypertension? Or it puts them at high risk that you are going to be able to detect that they are hypertensive later on?

**Q:** How would you interpret it?

**A:** Oh, I think that probably the start was maybe they were hypertensive but maybe intermittently hypertensive or not detected that they were hypertensive. And later on you discovered that they are hypertensive.

**Q:** So you doubt that covert stroke would actually be causal in terms of increasing the risk of hypertension. But do you actually think that hypertension is the cause of the covert stroke.

**A:** Probably. Yes.

**Q:** But we just discover it later on.

**A:** Yes.

**Q:** The diagnosis comes—

**A:** The chicken or the egg, kind of thing.

**Q:** Okay. No, I think that in asking these questions I’ve left it open for people to interpret it how they want to. But it sounds like what you are saying is, for high blood pressure you think that it is probably the risk factor for covert stroke and might be discovered later? And high cholesterol?

**A:** No, I don’t think stroke would give you high cholesterol.

[Laughter]

**Q:** Okay.

**A:** Everything is possible but--

**Q:** How about diabetes.

**A:** No. It is the same like hypertension.

**Q:** Okay. Headache.

**A:** I mean that’s a tough one because headache is very common. And, again, if you define these little things you see with migraine as silent strokes then you are linking—so I would say maybe, but not a strong maybe.

**Q:** How about vertigo?

**A:** Well, probably not but again, it depends on the location. But I doubt it.

**Q:** How about seizures.

**A:** It’s possible.

**Q:** Falls.

**A:** Yes.

**Q:** Memory loss.

**A:** Yes.

**Q:** Dementia.

**A:** It is the same line, yes.

**Q:** Anxiety.

**A:** Once they know about it, yes.

**Q:** Depression.

**A:** Yes.

**Q:** And schizophrenia.

**A:** Schizophrenia. I doubt it.

**Q:** How comfortable to you feel treating patients with covert strokes?

**A:** What do you mean how comfortable?

**Q:** How do you feel about it? Do you think that--

**A:** I mean they are not different than any other stroke, I guess.

**Q:** Okay. Are you implying that you feel comfortable then being a stroke specialist?

**A:** [Laughter] Well, what’s so special about them? [Laughter]

**Q:** Fair enough.

**A:** That they don’t recognize that they had a stroke.

**Q:** Okay. Do you follow any particular practice guidelines for the management of patients with covert strokes? And if so, which ones?

**A:** I was not aware that there are guidelines for—there are guidelines for that?

**Q:** No, actually.

**A:** [Laughter]

**Q:** But do you follow any guidelines?

**A:** But that’s a good idea. We can propose to the American Heart. [Laughter]

**Q:** Do you follow any guidelines though that help you? Like do you follow stroke guidelines or--

**A:** Yeah. I mean we all follow the same guidelines like in terms of working up a stroke patient and the treatment and—yeah.

**Q:** So maybe a refinement of that question is, do you follow secondary prevention guidelines or primary prevention guidelines?

**A:** No, secondary.

**Q:** Okay. Stepping back a little bit, what do you see—and I think you’ve already alluded to some of these already. But what do you think are the major knowledge gaps about this topic? And it could be for physicians or for patients.

**A:** Wow! I remember I talked about this but now my head is not—[Laughter] Well, I guess the first thing really is, are there a specific characteristic, whether it’s patient characteristic that help you to know which people would have more of the silent strokes than actually not silent strokes? I think the characteristic of the stroke itself I think, maybe not a lot published about this but I think you can tell just from clinical experience which one. It is probably as I said, just based on location and size. I think that is probably it. The patient really characteristic, that is probably the most important thing that we don’t know.

And maybe some of the questions that you asked, that we really don’t know now if they studied like systematically, what’s the impact of this silent strokes on all of these conditions that you asked on the cardiovascular risk factors. I think there is some data about like cognitive decline and dementia and stuff. But more in the systematic way, I think. That would be helpful.

**Q:** And just to make sure I understood that, the first thing was, you think that there is not a lot known as to what are the individual patient characteristics that would make someone more likely to have unrecognized strokes or silent strokes as opposed to ones that are more symptomatic or more obvious.

**A:** Yeah. You can ask it this. You can ask what are the features that make someone to not recognize a stroke as compared to someone who would recognize it.

**Q:** Okay. What would you want to know more about? Just those things or are there other things you would want to learn more or what things should be studied that might help you in terms of your practice?

**A:** I mean it would be good to, if there are really large kind of database to look what are the outcomes of the tests that we do. What’s the yield from the test that we do when you see a silent stroke? I mean if you find that 100 percent of them, you’re doing echo and Teli [?] and all this stuff and you don’t find anything, maybe we shouldn’t be doing it. I’ll be honest with you, I don’t remember that I ever found anything when I do these things. [Laughter] But you feel like you have to do it. But I’m not sure that there are data out there on this that would actually be interesting.

**Q:** So the last couple of question, this is a little bit of a thought experiment. And so thinking a little bit about how we might study this topic in the future, let’s imagine that the best evidence or like the best, new study that came out in a journal, like say next month is sort of a big, rigorous, observational comparative effectiveness study that was done to see if there are effective prevention therapies for these covert strokes.

And let’s say that that study actually found that the opposite of your usual practice is actually true. You mentioned that you might start a lot of these people on an aspirin. And let’s say they actually bleed more often than have benefit in terms of preventing future ischemic stroke. Would that type of study be enough to convince you to change your practice?

**A:** An observational study?

**Q:** Like a well-done, rigorous but observational comparative study.

**A:** I mean it’s a difficult question to answer because you really have to look at the study and then decide for yourself. Did it cover all the things that you would consider could cause a hemorrhage or not. Is it really the aspirin? Is it something else? Is it the underlying pathology in these people? So I would say maybe. I’m not sure. It depends. Really you have to look at the actual data and the study design itself.

**Q:** It sounds like you would want to know about the patient population and their characteristics--

**A:** Yes.

**Q:** Competing causes of--

**A:** Yeah. I don’t want just like the abstract kind of at the end of, [Laughter] the conclusion because it is always wrong [?][00:28:30]

**Q:** Fair enough. So you would really have to sort of pour over it in great detail and get a sense for whether or not it was convincing. You wouldn’t take it at face value. Not that I would expect you to take it at face value. So if that’s sort of a major--

**A:** In general, I mean I’ll have to say that it’s really difficult to convince people to do something, just based on observational data if they data were not randomized. So I think it will always be a challenge.

**Q:** So you think a randomized control trial would be more convincing in terms of guiding your therapy or changing what you do?

**A:** Probably. Yeah.

**Q:** If a randomized control trial were performed, just thinking about this type of scenario, where these findings are often found on studies incidentally, if you were sort of involved in the design of that type of RCT how would you recruit patients into the study? Or if you heard that people were designing that type of study, how would you suggest--?

**A:** Well, it’s a difficult study to design. That’s the problem with it because—so do you follow them for how long. [Laughter] You keep them on aspirin for how long? Two years? Three years? I mean I think you start running into what’s practical to do a trial and what’s—so you probably are not going to be able to follow these people for more than a couple of years to get out data from it.

**Q:** Are you implying that you think that the outcomes are probably beyond that time point?

**A:** You don’t know. Probably. I mean it’s going to depend on a lot of things. It is going to depend on their age. It’s going to depend on the extent of white matter disease. I mean it’s going to be very difficult to perform. I think it is actually impossible. [Laughter]

**Q:** Why do you think the extent of white matter disease is important? That’s the first time you mentioned that.

**A:** Well, I mean it’s a sign that there is an underlying vessel pathology I guess. And they tend to be more susceptible to bleed. So I guess if you have it, that might be a reason for increased bleeding as opposed to someone who just has a little, tiny maybe cortical infarct that you didn’t see before. So.

**Q:** Okay. So we talked a little bit about outcomes maybe being later than what a randomized control trial could account for. But let’s say that they were able to be detected within the time of an RCT. Actually, let me scratch that question. Let me rephrase that question, actually. If a randomized trial were done, what would be the outcomes that you think would be important to assess?

**A:** For patients with silent stroke?

**Q:** For patients with silent strokes.

**A:** I think would be imaging, are you seeing more silent strokes or not. And maybe some cognitive outcomes I think [00:31:56].

**Q:** Okay. Finally, let’s say some type of study were done or maybe a combination of studies, some comparative effectiveness studies, some clinical trial, maybe a few of them that found that there were effective means of preventing stroke, let’s say, after a silent stroke. How would you go about, in your own practice as well as sort of on a more broad scale, how would you propose going about making, implementing those changes? Like let’s say maybe everyone should be put on an aspirin and a statin, how would you sort of approach that?

**A:** Well, I’m not sure what you mean, how would you approach that? I think you just have to spread the word out that if you see a silent stroke, it is really, they’re treated as secondary prevention.

**Q:** You’re just telling me like that you’d want to spread the word in terms of--

**A:** Yeah. I mean I’m not sure what else. I guess the question is not really clear.

**Q:** Maybe let me rephrase it because I think one thing that can be useful in terms of thinking about this question is actually hearing a statistic. So there have been a number of prospective cohorts where they’ve looked, for example, the Framingham Heart Study, the Cardiovascular Health Study, the Rotterdam Study, NOMAS, some other ones—where they’ve actually done routine MRIs at a pre-specified time points and look to see what is the incidence of silent strokes or I guess the prevalence per se. And they found that above, for people above age 50, if you take that whole group, it might be as much as 20 percent.

And it might start on the lower end. At the lower end of the age spectrum, like in the fifties, sixties, it might be more like six, seven percent. But then, about ten years later it might be 15 percent. Ten years later it might be 25 percent, 30 percent. So it is something that seems very common in a screened population and those types of studies. So those are the best estimates that we have so far in terms of how common an issue this is. So that’s maybe as much as 20 percent for covert stroke as compared to somewhere between two to14 percent for clinically apparent stroke.

So the nature of the question I think is kind of like what you’re getting at. It’s like how would you approach in your individual practice but also as an expert, how would you suggest the country or the world, the field approach an issue that’s this common.

**A:** Well, I mean, I guess unless you understand their etiology, it’s going to vary. You go back to the same thing about the super pill, where everyone should be taking antiplatelet, a statin, and an ACE inhibitor. I’m not sure how you can really impose this. You can’t. And I don’t think it’s practical to just say because the odds of finding a silent stroke as you get older then, instead of just taking a baby aspirin as you tell someone in their fifties, then take an aspirin, a statin and something for the blood pressure.

If you try to apply this across the entire world I think economically it is probably not do-able. I don't know.

**Q:** What do you think about screening? Do you think people should be screened for this?

**A:** I’m not sure that—so screening like everyone at a certain age you get an MRI, I’m certain that this would be cost ineffective. Because I mean people, it’s the same thing, when this was done for aneurisms and stuff like that. It’s never cost effective when you do that. And at least aneurism is probably, there is a more definite kind of intervention for it that you would do than this that would be--

**Q:** So you need to have an effective intervention. You also seem to--

**A:** You have to target the population. I think if you just mass kind of, just everyone does that, I think it’s difficult.

**Q:** Just because I think you’re a thoughtful guy, what do you think are—if you were just to guess right now, what do you think are the things that would help you predict who is likely to have a clinically unrecognized stroke or a silent stroke on scan?

**A:** Oh, the people the risk factors. I think a lot of the people with the risk factors that--

**Q:** People with traditional stroke risk factors?

**A:** Yeah, I think so.

**Q:** Okay.

**A:** That would be really interesting to see, which I don't know is anyone like—is there a difference in the percentage or in the prevalence of silent strokes in people who actually you’re imaging because they had a stroke and you see that they have silent strokes on top of them, all silent strokes compared to just imaging what you think are healthy people. Is it more common? I don't know.

**Q:** It would be interesting to find out.

**A:** I don't know the answer to that. My guess is that is probably not more common but I don't know.

**Q:** Well, that’s actually the end of the interview. Do you have--

**A:** I mean one way to look at them I think is that you’re like, like you see someone now has an acute stroke and you image them and you find an old stroke. The ones that you get with silent strokes, it just you got them early a little bit, I think, in the curve, before they have the symptomatic stroke.

**Q:** Yeah.

**A:** That’s it? We survived?

**Q:** You survived. Do you have any additional questions or thoughts that you didn’t express?

**A:** No. I’m interested. It will be interesting to see--

**Q:** Sounds good. We’ll end the interview right now. I’ll stop the recording.

END OF INTERVIEW

Internist 5

Study ID: IN5

**Q:** So this is going to be the interview with our fifth internist. Can you say hello?

**A:** Hello.

**Q:** Okay, so we’ll get started. There's 13 questions, but it really just depends on how talkative you feel and how much detail you want to go into in the beginning because it might be like you answer several questions right off the bat. But the first question is just to tell me what you know about silent strokes or what are sometimes called covert strokes?

**A:** I guess I would see that-- I tend to think about them when I have patients who are hypertensive or diabetic and then-- yeah, then my-- if I got like kind of CT or MRI or someone else does it in the ER, I might see a finding that suggests an old stroke, or they say something like that. Yeah, and I think of them as mostly kind of diseases of hypertensive-- older hypertensive patients or older diabetic patients.

**Q:** And I think you're starting to talk a little bit about the risk factors and things that might predispose people to this, but why do you think they occur?

**A:** I just presume it’s kind of vascular paths [?] who have those risk factors, have atherosclerotic development over years and that can lead to either kind of occlusive disease that leads to stroke or a lacunar kind of infarct that's sub clinical, or-- yeah, so I'd say that. And I guess I wouldn't think they’d be embolic in nature typically just because you'd expect those to be more symptomatic.

**Q:** I think maybe you were starting to talk about that a little bit in terms of differences between stroke and embolic strokes, but I want to see if you have any thoughts about the pathophysiology of strokes that don’t produce symptoms. So if you think that they're similar or different to some strokes that are more overt or obvious?

**A:** Yeah, I think that would be my take on it, would be that they're typically more thrombotic or not typically the embolic strokes from the heart or the aorta, but are even paradoxical emboli, but more just these kind of chronic, slow processes that lead to progressive atherosclerosis. And most likely, these are small vessels like penetrating vessels or those would be more typical, I think.

**Q:** Do you think there are, just like there are different types of symptomatic strokes, do you think that there are different types of silent strokes?

**A:** I never thought about it, but I guess it would depend on the location. I mean, obviously depending on where it affects the brain, and there are probably subtle manifestations that a person doesn’t notice that they might attribute to aging or might attribute to some decline in function related to some other co morbidities they might have and they don’t recognize it’s actually a silent-- it’s not really a silent stroke, but it’s a sub clinical stroke that isn't that obvious to either the doctor or the patient. But I haven't really thought about it.

**Q:** Okay. So you mentioned that you don’t encounter a whole lot of these in a given year. But, if you were to encounter more, how do you-- if it made sense to break it down, and it might not, but if you had to kind of group them into categories, how would you differentiate them in terms of what's meaningful to you?

**A:** I mean, that’s tough to say. I guess the biggest breakdown to me would be is it truly aclinical or is it sub clinical? Like if I could distinguish that, I don't know that it would change my management of a patient, per se. I think the treatment of a patient like that often I feel like they’ll already be on therapy. So if they weren't on therapy, I think I might treat them anyway, whether it’s clinical or sub clinical or aclinical. So I don't know that any category I could come up with, and I can't come up with right now, would change the way I would treat them. So I might not-- in general, my practice is I don’t really care about stuff if it won't change my management if it’s not within some specific passion of mine that I really want to know the subtleties.

I think in stroke, I tend not to go too crazy with categorization because I feel like the treatment’s going to be quite similar for most cases with the exception of embolic disease, which I think, again I could be way off, but if that's correct, that these are often embolic, then you wouldn't necessarily think about kind of different therapeutics like coumadin or something like that.

**Q:** Sure.

[side remarks]

**Q:** Okay, that’s helpful to know. I think one thing that we often run into, especially when we're looking at reports from the radiology scans is the radiologist will often use different language and oftentimes they're referring to what's called white matter disease or microangiopathic changes. And for you, do you think that there's any-- are they the same thing in terms of silent strokes and white matter disease? Or are they different?

**A:** I actually struggle with that a lot, like when I see small vessel ischemic disease, what they report that as. I don’t treat it as a stroke, which it would be helpful if the language were different. To me personally, I think it would change my management of patients if someone said this is a stroke and this puts a person at high risk for further strokes or more clinically apparent strokes. So the language definitely affects me. Like if I see old stroke, I would think very differently than if I saw small vessel ischemic disease. Because I think those things are below my threshold for treatment and again, that may be wrong, but that's definitely how I viewed it.

Like, I think of it as hypertensive, kind of white matter changes or whatever, small vessel ischemic disease, I do think of it as oh, this is kind of chronic stuff that doesn't merit that level of aggression that someone-- they say old infarct.

**Q:** Yeah. So it’s sort of like they may have similar risk factors, but they're not quite-- they don’t have quite the same clinical significance?

**A:** Yeah.

**Q:** Okay. This is sort of a similar question kind of circling around what we're talking about. And I know you mentioned hypertension and diabetes, but what do you think are the major risk factors for silent strokes?

**A:** I haven't really distinguished it much from what I would say is traditional cardiovascular risk factors. I'd as smoking, hypercholesterolemia, hypertension, diabetes. I would probably put, I would think, hypertension and diabetes are higher on the list than some of the other ones. Smoking, I wouldn't know for sure. I'd just assume it’s a risk factor, but I wouldn't know if I'd put it higher or lower. I'd assume that any of the other things that kind of predisposes to hypertension chronically like end stage renal disease-- I'm trying to think what else would be big ones. I mean, when we talk about general stroke stuff, you could talk about more unusual things, like hyperhomocysteinemia, whether that would be-- or just homocysteinemia. But yeah, it would be kind of those traditional ones that would jump to my radar.

**Q:** Okay. [00:08:50] kind of warrants you-- what it’s like seeing these folks, what are the types of scenarios where you've encountered somebody with a silent stroke?

**A:** Yeah, I'd say the biggest one is kind of what you said, is the small vessel ischemic disease where I have a person I know is hypertensive and diabetic, or a smoker plus/minus and just they have-- it’s listed as small vessel ischemic disease. And again, that doesn’t typically kind of raise red flags for me. It’s like something I'm not surprised to see, and often these patients are being treated, so I think I don’t really think twice about it. I have read, basically, that these changes are-- they are a prognostic factor so that has kind of raised it a little more in my mind as being important. But I don't think, again, if any of these patients had strokes with or without the small vessel ischemic disease, I would be shocked. I'd be like, okay, yeah. I mean, they have the risk factors. So that's typically when I've seen it.

I'd try to think of a time where it wouldn't be those patients, but really nothing jumps to mind where I feel like the stuff that's in my head, that I recognize, are people who have those risk factors and that’s maybe why I'm noticing it or why I'm remembering it. And I may be missing cases where I actually-- I saw that, but they didn't have risk factors. But the only case I can recall right now are the cases where it was like pretty obvious that it would be there.

**Q:** Out of curiosity, did you read up on that because I asked you to do this interview or because you just came across--

**A:** Yeah, I came across it on Medscape a couple of months ago.

**Q:** Fair enough. Are there particular common indications for the scans that you run into? Are these scans you're ordering or that other people are ordering?

**A:** Could be a combination of stuff. Like I've ordered a few CT scans, MRIs for people who have early onset dementia and just trying to make sure there's nothing reversible. And then occasionally, it’s more my patients get seen in the ER for some reason and get a head CT for changing mental status or something along those lines. That would be a typical scenario.

**Q:** Yeah, definitely. And when people have those, whatever the indication is, change in mental status or if you're worried about their sort of long-term cognition, do you end up tying the infarct that’s seen on the scan to those sort of initial indications, or do you end up thinking of them as being separate?

**A:** Yeah. I would say that for small vessel ischemic disease, I don’t-- I mean, I think about vascular dementia, but I don't know exactly how small vessel ischemic disease would impact that, like whether that's a known correlation with it. So actually, I don't know if that’s related or not. If it's really dramatic, I would probably say, oh this is probably vascular dementia. If it's the typical readings where it says, “Oh, some small vessel ischemic disease,” I probably wouldn’t tie it to it. I'd just say it’s Alzheimer's, since just probabilistically speaking, that's the most likely cause of dementia in my population.

So, I guess it would depend on the extent. I have a guy who has had multiple strokes and certainly has had a bunch of sub clinical strokes, without a doubt. And for someone like him, I would-- but he has actually visible old infarcts that are clinical significant. So for him, it’s natural for me to call him vascular dementia. But for people not as obvious as him, I probably don’t make the link. I would still say Alzheimer's for those people.

**Q:** Okay. One of the things that often happens in sort of regular practices is we come across these incidental findings on scans or tests of various sorts. I'm sure, especially for you, you run into this a lot and in different organ systems and often there's a question as to are these meaningful, are they not meaningful. Just exploring the concept is something that's incidental in how you handle that. What does it mean for you for something to be an incidental finding in terms of how you approach it?

**A:** Yeah, I think you can't really define it as incidental until you do a workup because a pulmonary nodule is incidental, but if it’s cancer, it’s no longer incidental, it’s real.

**Q:** [00:13:35]

**A:** So, I mean, it’s really incidental is a term that’s used after a workup. I mean, it’s used in the literature as incidental, but to a primary care physician, or at least to me, you can't call something incidental until you know it’s incidental. Like, it could be something serious. And I understand it’s a bit semantical, but to me, all of those things need some attention. In pulmonary nodules, you know, the guidelines say if it’s less than four millimeters, don’t worry about it. So that's my guide there. For adrenal incidentaloma, the guidelines say you need to rule out pheochromocytoma and sub clinical Cushing’s Disease, so that's my threshold there.

In stroke, or in small vascular ischemic disease, no one has really told me that I know of, like okay, you need to do something about this. Like every one of these patients needs an echo and carotid studies or transcranial Dopplers.

Like if someone told me that, that would be my threshold. But because I don’t have that kind of expert guidelines in my head, then to me that's an incidental finding. Again, the issue there, to me, is how would it affect my management? Like, a pheochromocytoma would affect my management. A sub clinical Cushing’s would affect my management. But because I don't know that it would change anything I'd be doing, I'd have it-- I would view that as incidental.

**Q:** Certainly. I think you mentioned that a lot of these patients-- the ones that you can recall tend to-- you know that they have some cardiovascular cerebral vascular risk factors and so they already might be on some sort of treatment. Do you ever end up starting anything new, or can you recall ever having ordered a test?

**A:** I can't recall it, yeah. I was actually just thinking about it in preparation for this, but I can’t think of any cases where I could be definitive and say, like, “Oh my God, I saw that and I totally started something.” I really can't recall. I feel like most of the cases I've seen-- the only cases I can recall are those types of people and that's why I'm not sure.

**Q:** So no obvious change in medications or testing or referrals?

**A:** Yeah, nothing.

**Q:** Okay. Do you ever mention anything about lifestyle changes or dietary changes or anything to patients in response to this? Or is it just kind of a continuation of--?

**A:** Yeah, because most of that stuff’s already been done. I mean, so yeah. I wish I could tell you a case where it was a person without risk factors. And if I did something definitive, but I can't recall any--

**Q:** If you can't recall it, then--

**A:** I can't recall it, yeah.

**Q:** Which is fine, okay. I think you responded to my email, which is actually in relation to the patient study. But my next question was going to be-- was going to ask if you talked to patients about the imaging findings and how you do if you do?

**A:** I don’t usually talk about small vessel ischemic disease just because I feel like it’s something that I feel is not surprising. And to me, it’s a little tough to talk to them about what that means. And again, prior to this study, even after the study, I don't know, I was thinking would I say something differently to the patient? I already counsel them on their diet and exercise. I already give them antihypertensives. I mostly give them statins. I think where it would really affect my counseling would be in the setting where I'm not doing that. Yeah, to me--

**Q:** It’s sort of a surprise finding?

**A:** Yeah, like a good example was it’s not directly equivalent, but it is somewhat equivalent, was where-- again, if this had this kind of prognostic significance, it would affect the way I would think-- so, I had a guy who I kept pushing to do a statin because he’s diabetic and he kept saying no, no, no. Then I heard a bruit, a carotid bruit, on exam and we got a study and he had 50 to 79 stenosis of his left carotid. And then I said you have to be on a statin. And that changed the way I approached it.

So yeah, if someone told me, you know, these kind of findings merit very aggressive cardiovascular risk reduction, I think that's where it would affect me, where the patients weren't adequately medically managed. And then I would say, like, I would be very firm with my patients and I'd say this is critical to you. Or the way I would frame my approach would be different if I knew those facts.

**Q:** Okay. I think you may have-- well, actually, this is a little bit of a different question but in terms of sort of the question of clinical significance, but do you think that the silent strokes themselves have caused any harm to patients that have them?

**A:** I would say, again, it’s-- are these strokes truly silent or are they sub clinical and we just don’t pick them up as physicians because we're not aware of the subtleties, or do patients blow these things off as aging related phenomena? So I would say I would guess that silent strokes do hurt patients, but in subtle ways that they don’t detect because they're gradual or subtle. So I would say that would be-- can they hurt them in other ways? Nothing comes to mind other than the fact that we know that they're a setup for very bad things down the road potentially.

**Q:** If they have they caused any sort of subtle problems, what do you think those are?

**A:** I would guess subtle motor findings or subtle-- sensory findings could be subtle, too, like they might-- we always talk about multifactorial bases for gait disturbances, so could they have subtle cerebellar dysfunction that they're less coordinated than they are and going, “Oh, I'm just getting older,” and so they drop things or they're more clumsy, they fall more. So I would envision that you could imagine that all those things, gait disturbances and quality of life issues with being able to do things that they would normally do, like fumbling with buttons or things like that you could imagine could be an issue. So I assume that all those things could happen. But probably from a clinical standpoint, the biggest thing might be falls. I would worry about, especially in the elderly with the risk of hip fractures. And maybe subtle visual problems, too, depending on where the strokes are.

**Q:** In terms of other medical conditions or some other health issues, do you think that the silent strokes put people at risk for those? Like, anything in particular?

**A:** I mean, if they become sedentary, then they have all the kind of risks for sedentary people; obesity and worsening diabetes from a sedentary lifestyle, worsening hypertension. So I feel like it could be a vicious cycle that a silent stroke leads to.

**Q:** Okay. To kind of expand a little bit on the idea of whether or not silent strokes have an effect on someone’s health, what I'm actually going to do is I'm going to list a number of different medical conditions or risk factors or symptoms and just see if you think that they're connected, specifically in the direction of does a silent stroke lead to X, or blank. But if you think that it’s not or if it’s a reverse or something, you can specify that. You can say yes or no or some sort of qualified answer. So again, the framework is do you think that silent strokes place your patients at risk for any of the following? The first is stroke symptom?

**A:** I'd say yes.

**Q:** And then the second is any sort of intracerebral hemorrhage? Actually, I'll specify. Intracerebral hemorrhage into the brain tissue, not subdural or something?

**A:** Yeah, I guess. I'd even think of like grangiopathies [?] as maybe increasing risk for bleeding so I'd say sure.

**Q:** How about myocardial infarction?

**A:** I would qualify it by saying, like, they have risk factors so I'd want to see the data that shows how you can prove the chicken or the egg.

**Q:** Sure, so it’s more that they have shared risk factors rather than there being a causal relationship?

**A:** Yeah.

**Q:** Okay. How about heart failure?

**A:** I'd say the same for that. Associated, but not necessarily causally related.

**Q:** And high blood pressure?

**A:** I guess, again, causally. If it lead to changes in lifestyle, I'd say yes. But I couldn’t see the direct mechanism.

**Q:** Okay. How about high cholesterol?

**A:** Same thing, lifestyle related, but not necessarily. I can't see the causal relation.

**Q:** Diabetes?

**A:** Same thing.

**Q:** Headache?

**A:** I wouldn't know how it would do that.

**Q:** Vertigo?

**A:** Sure, sub clinical stuff.

**Q:** Seizures?

**A:** Sure.

**Q:** Falls?

**A:** Sure.

**Q:** And memory loss?

**A:** Sure.

**Q:** And dementia?

**A:** Sure.

**Q:** Anxiety?

**A:** I wouldn't think of that right away, so I'd say no.

**Q:** Depression?

**A:** Sure.

**Q:** And schizophrenia?

**A:** I would say no just because I'd be surprised at young ages they’d get those silent strokes.

**Q:** Okay. Next question is how comfortable do you feel treating patients with silent strokes?

**A:** Again, it would be if I'm missing the guidelines, then I may be doing the wrong thing. But I would feel like-- I know the literature okay for strokes, so I feel like I would just treat them with a statin and aspirin. So I'd say I'm pretty comfortable in blood pressure control.

**Q:** Uh-huh, I think you've answered my sub question to this, but I was going to ask if you follow any practice guidelines, but it sounds like you've kind of identified that you're not aware of any guidelines for this issue?

**A:** Right.

**Q:** From your perspective, what do you think are the major knowledge gaps in the things that you would want to know to help you with your practice?

**A:** I would want to know-- I think the big thing is should it change my management of my patients? Like, if I see silent strokes in patients, should I be giving them-- like if they're on aspirin, should I be adding Plavix? Should I be changing to a different agent? Those kind of things would be the big gaps. So for me, from a practical standpoint, it would be what should I do differently? Tell me what I should do differently if I see a silent stroke. So same thing, that would be the big gap in my knowledge.

And otherwise, I would say knowing the difference, if there is a difference between the radiological readings, like what radiological readings should concern me and which ones shouldn’t? So if you see white matter disease, is all white matter disease equivalent or is that-- should I assume all white matter disease is a silent stroke outside of people who have MS or something like that? But so those kind of things is a gap for me. Like, I don't know how to interpret the radiological findings in a subtle or nuanced way.

**Q:** So the last question is actually a-- it’s sort of a subset, it’s really kind of a thought experiment in a way. And I'll introduce some information to you and kind of put you in the-- put you in a sort of theoretical space a little bit. So the first is to-- let's say there aren't really necessarily proven treatments yet. Or let’s say that we know that people with silent strokes are at more risk for having stroke, but we don’t yet know what's the right way to prevent stroke after someone has one of these on a scan.

So let's imagine that in effort to address this that there's a large, sort of rigorously designed observational comparative effectiveness study that's trying to determine the effectiveness of prevention therapies for stroke after a silent stroke. And let's say that the study actually finds that patients should be going on these medications, like let's say they should be on an aspirin and a high dose statin and an ACE inhibitor or something. Would that type of study be enough to change your practice in terms of sort of setting up enough evidence for you to follow them?

**A:** I think in certain patients, it’s easy, right? Because again, in your diabetic, hypertensives, it’s not going to require much of a push to do that. And again, I would say that it would probably frame the conversation for me differently, just like I said before. I would be more aggressive. I would say, “Look, there are now observational data that suggests that you can benefit from this. It's not for sure, but when it comes to stroke, I wouldn’t mess around personally. So I'd strongly encourage you to take this medicine.”

So if they're already on the medicine, you know, I could envision being more aggressive with statins potentially with a study like that. Saying like, “Hey, I know you're on 40, but we just had this study, maybe we should go to 80,” if there's some evidence that that matters. So I would say that would be the biggest thing. It would probably frame my discussion differently. But yeah, but without definite randomized studies, I don't know that I would be hyper aggressive. Often, it would go down to patient preference and ideally, if things were working, ideally I would have a more probably vigorous discussion with patients who weren't on those meds and say, “You know, there is this data, there are these data out there now, that suggests you could be at high risk for stroke given the CT findings we saw in your scan a few months ago. How does that make you feel? Would that lead you to want to be more willing to take medication or not?” Something like that.

**Q:** If a randomized control trial were done, how do you think patients should be recruited into that type of study for this issue where they don’t-- they have symptoms and [00:29:05] were found incidentally?

**A:** I don't think it would be-- it could be awkward for primary care doctors for sure because their patients are going to be pissed and say, “Why didn't you tell me about this finding?” I think you could do it through radiology and just say that there are some new data that have come to light suggesting that this could be a problem. Your primary care doctor was not aware of this because something-- I might try to protect the primary care doctor just for my own personal interests. But I think that would be the best way to do it, to go through radiology because to primary care doctors, it would torture them to recruit patients and you probably wouldn't be very successful.

But I would just say, yeah, I would do it through the radiology department personally with that caveat.

**Q:** What do you think for you or for the patients, what do you think would be important outcomes to look for?

**A:** I mean, the important outcome would be stroke prevention, would be huge. I mean, but like you said, if there are a bunch of sub clinical things that we're not aware of, that these strokes are actually-- that we're not aware of that could be a big difference in patients’ quality of life, then that would matter a lot to patients, obviously. I mean, if gait disturbance and falls are significantly-- if you can prove a causal relationship with silent stroke, that would be enormous to our patients. I mean, so many of our patients have severe injuries related to falls, or they live lives in great fear, which totally diminishes their quality of life.

So, I would say for me, if you could improve gait and you could reduce strokes, that would be an enormous benefit to patients. I mean, obviously the other stuff would be big, too, just because you assume-- like you said, if there's diabetes, if OSA, if there's all these other things, hypertension that are worsened by silent strokes, presumably if you start controlling silent strokes in some way, then you should improve these risk factors which should obviously have the impact, same impact, of-- presumably, you're going to make the same interventions for those things as you would for silent stroke. So, I can't see a clear example, but let’s say there's drug X that has nothing to do with those traditional risk factors that reduces silent stroke but doesn’t reduce those traditional risk factors. But by improving silent stroke, you then-- like you said, that causal relation, drug X actually fixes silent strokes and then those risk factors get better from the more active lifestyle, from blah, blah, blah. So yeah, to me, that would be ideal.

**Q:** I should have prefaced that laundry list by saying as far as we know in the literature, we don’t know that all of those have a relationship.

**A:** No, I know. No, I'm just saying that if they did, yeah.

**Q:** If they did, yeah.

**A:** If they did, it’d be nice.

**Q:** Let me give you a couple of known statistics. So there have been a number of large cohort studies that have looked at this including the cardiovascular health study, the Framingham heart study, Rotterdam, the Northern Manhattan study. A lot of them have actually done serial MRIs as part of the study, so they're just done at fixed time points. And they’ve kind of accumulated that data and come to an estimate that for individuals over age 50, kind of like what you're talking about, the sort of older populations, the prevalence of silent strokes is about 20 percent. And that, of course, increases with age. Framingham is kind of right in the middle in the 50s, people are out around 7.5 percent in terms of the prevalence and then it just increases with time to 15 to 30 as you're sort of going up by each 10, 15 year interval.

And we also do know based on a couple of decades of epidemiology research that there's a strong association between silent strokes and both symptomatic stroke and dementia with a sort of two to four-fold increase, relative increase, for each of those.

And so knowing that in terms of how common it is for at least a couple of outcomes, if we were to find some prevention strategies that are modestly effective, do you think it would be worth implementing some type of screening strategy for this, or some other type of strategy for addressing that?

**A:** I would say yes. Dementia is the [00:34:02] factor for all those patients and for us and so, I mean, if you could find some screening study and some beneficial way to screen-- easy way to screen and say that you can actually get some reduction in dementia, either onset or at least delayed onset or a lower incidence of dementia, I think it would be really worth it. And stroke, of course. I mean, that's obvious.

**Q:** Even if it’s expensive, an MRI?

**A:** Yeah. I mean, that's the tough question. Then you're getting into cost effectiveness analysis and then you have to make kind of a decision from the priority of the nation and blah, blah, blah. But yeah, I would say it’s a no-brainer if it’s cheap and then obviously it would be a question mark if it’s really expensive.

**Q:** Yeah, okay. So we're at the end right now, but I just wanted to see if you had any additional thoughts or questions on your mind having talked about this for the last half hour?

**A:** I guess if there was any evidence out there other than kind of what you'd expect in terms of treating silent stroke.

**Q:** I can actually end the interview now and tell you as part of the debriefing what we know and what we don’t know.

**A:** Okay.

END OF INTERVIEW

General Neurologist 3

Study ID: GN3

**Q:** So this is going to be the interview with our third neurologist. And what I’m going to do is I’m going to ask you—there are currently 14 questions and they’re actually some sub-questions that go under that, just depending on how much you volunteer in terms of information.

**A:** Sure.

**Q:** But it’s a little bit, it’s organic in some ways so there’s not kind of right or wrong answers and you can just speak however you’d like about the topic. So, the first question is just to have you tell me what you know about silent strokes so far.

[00:00:31]

**A:** What I know about them?

**Q:** Yes, what your thought process is.

[00:00:34]

**A:** Yeah, well, my thought process. I mean I think silent strokes from my experience and the incidence increases as patients get older. And often you find them incidentally when you’re evaluating patients possibly for other symptoms or question of stroke-like symptoms. But perhaps you find things incidentally that don’t really necessarily fit their clinical syndrome or clearly look older that the onset of their symptoms.

From my experience it’s something we encounter fairly often, I think particularly in patients, like age is a factor. I think definitely patients who have a history of cardiovascular disease, diabetes, hypertension, too, I think from my experience also are patients that I tend to, you know, have these findings as well.

**Q:** Okay. Great. The diagnosis itself has been called a whole bunch of different things, both in the medical literature and also clinicians will convey it to themselves or to patients. And so some of the names that have cropped up include silent stroke, silent brain infarction, silent cerebral infarction, covert stroke, covert brain infarction, subclinical stroke, subtle stroke and asymptomatic stroke. Which of these terms have you heard used before?

[00:01:47]

**A:** I’ve heard silent stroke. I’ve heard covert stroke. I’ve heard the term covert used in these patients and asymptomatic stroke as well.

**Q:** Which do you use most often?

[00:01:57]

**A:** I used silent, yeah, clinically silent strokes.

**Q:** And are there other terms besides the ones that I mentioned that you’ve heard used before?

[00:02:07]

**A:** Nothing that comes to mind.

**Q:** Which do you think is the most useful or the most appropriate term to use?

[00:02:17]

**A:** I think clinically silent since—I’m biased, too, because I tend to use that one. I think that fits it pretty well. I think asymptomatic stroke I think is a good one, too. You know, I think emphasizing the likely mechanism of the finding on the imaging study, but understanding that there is any clinical correlate. So I think any term that encompasses those two aspects are certainly suitable.

**Q:** Why do you think silent stroke occur?

[00:02:43]

**A:** Yeah, well, you know, the brain, it’s really all location, I think, depending on where the lesion is, whether the patient has symptoms or not. So certainly, for example, frontal lobe for example, a small frontal stroke, I mean certainly can be clinically silent. Certainly brainstem stroke might be less likely. Just in terms of real estate, it’s really all location and density of pathways. So I think certainly you can have strokes in areas, which are just—fortunately for the patient, just don’t hit anything that’s clinically significant. So it doesn’t present with anything.

**Q:** From your perspective, do you think that silent strokes have similar or different pathophysiologies to symptomatic strokes?

[00:03:28]

**A:** Yeah. I would assume that the pathophysiology is quite similar. Again, it’s just the terms of location, at least that would be my thinking.

**Q:** Okay. Do you think there are different categories or types of silent strokes? And if so, will it—how do differentiate them and categorize them in your head?

[00:03:49]

**A:** Well, I think just like we categorize clinical strokes, I think, I think there are clinically silent strokes that may fit a more lacuna picture, again, reflecting the life of the mechanism, which from my experience is probably the more common presentation—versus, strokes that look maybe more embolic in nature, which are more cortical. Which may again be clinical. So I think just like with clinical strokes, I think you can have similar differences in mechanisms. Again, it is just all the matter of location. I think it is really their main distinguishing feature where it hits some sort of functional area.

**Q:** And in your perspective, how are silent strokes similar or different from white matter disease?

[00:04:37]

**A:** I think white matter disease tends to be more confluent, you know, very patchy. It is always, generally in the periventricular area. While I would view, what I would distinguish it between now is a clinically, silent stroke where it’s a discrete, which is, you know, quite consistent with the sort of focal, vascular injury that sort of stands alone—rather than the kind of confluence, you know, collection of small vessel disease that we see otherwise. So I think that is what would distinguish it from--

**Q:** Do you think they are similar or different in terms of their clinical significance?

[00:05:19]

**A:** I think clinically silent strokes, I think would be a little more clinically significant. Again, I think white matter disease often is a chronic accumulation—you know, again, risk factors are similar. But that is more of like a chronic, I wouldn’t say exactly a progressive process but it’s a chronic process that we kind of naturally find in patients as they get older.

A clinically silent stroke though, to me, could potentially suggest, again, and underlying etiology that needs to be addressed, particularly if it looks more like a embolic type of stroke. So I definitely think, you know, there are clinical implications.

**Q:** Would you—with respect to clinical significance and your approach, would you react to one but not the other? Or do you react similarly to both?

[00:06:08]

**A:** You mean in terms of white matter disease versus--

**Q:** The white matter disease versus silent strokes?

[00:06:13]

**A:** Yeah. I think clinically silent strokes, I think I probably would be a little bit more aggressive. I think I would—again, particularly if the stroke location is more of an embolic source, I think I would initiate, if it hadn’t been done for other reasons, at least some sort of baseline kind of cardiac work up, that sort of thing. I think if it looks more lacunar, though, in nature, again, just making a judgment, just based on location—I don’t think I would necessarily be as aggressive.

Certainly if a patient was not on anti-platelet I would initiate, again, looking at all the other risk factors that can be controlled, high blood pressure, diabetes control, things like that. I think I would pay a closer eye on. But I think that—I guess those types of strokes I probably would treat similar to what I do with kind of white matter disease that might come up incidentally as well. I think those patients as well, I would try to modify those stroke risk factors as well. So it is really the ones that look more cardio-embolic-y, more cortical type and location, I might be a little more aggressive.

**Q:** Let me do just a quick sound check real quickly. What do you—I think you’ve talked a little bit about this—I’m just going to have you kind of expand a little bit on this. But what do you think are the major risk factors for silent strokes?

[00:07:34]

**A:** Right. So, I think patients who have underlying cardiovascular disease, you know, high cholesterol, high blood pressure, particularly if it is uncontrolled, I think definitely diabetes I think are all risk factors, smoking. And I also think patients who also have, who do not interface with medical care, regularly, so patients who don’t follow with primary care physicians on a regular basis I think also are more likely to have clinically silent strokes.

**Q:** In what settings or scenarios have you encountered patients with this diagnosis?

[00:08:09]

**A:** Yeah. So, commonly in the outpatient setting where I may be getting MRI’s for other reasons, or maybe investigating, you know, unusual symptoms, that sort of thing. And then I think, usually, just based on MRI scans in those patients, you just find, incidentally, maybe a lacuna or, again, evidence of an old stroke or white matter change—which, when you ask the patient historically, from their history, from their standpoint that they don’t recall any corresponding symptoms.

**Q:** For these situations where you’re ordering scans, what are the types of issues or symptoms that the patients presented with?

[00:08:46]

**A:** Yeah. So headaches would probably be a common one. Or patients who may be having sensory symptoms, you know, maybe unilateral sensory symptoms or subtle motor symptoms, that sort of thing. Also patients for dementia evaluation, you know, cognitive, neurodegenerative disease workups, that sort of thing.

**Q:** In that context, did you end up kind of on average connecting the patient’s symptoms or issues to the findings on the scan or was it completely separate?

[00:09:21]

**A:** I generally view them separate. I guess if some of these patients, like with cognitive symptoms, possibly—again, if it tends to be particularly a subcortical type of stroke and in conjunction with other white matter disease, things like that, maybe there might be some correlation with it. But in the most cases, generally, I would probably view it independently.

**Q:** You mentioned the term incidentally. And the silent strokes often are encountered incidentally, just as you’re describing when clinicians are investigating a symptom or medical problem that doesn’t overtly seem to be related to stroke. And then they find this on the scan. So just for you in your general practice, what does it mean for something to be incidental?

[00:10:07]

**A:** I think when you find an abnormal finding at does not clinically correlate with the patient’s symptoms or their complaint. Yeah.

**Q:** When—it can maybe vary. But for silent strokes, when you encounter those, do you feel obligated to respond to them?

[00:10:33]

**A:** Yeah. Well, I think again—again, if I use my, if I think in terms of my clinical judgment, if I think the location—it really depends on the location of the stroke. So I alluded to this before. But If I think that the stroke may have an embolic type of evaluation, and the patient either has no history of any sort of cardio-embolic risk factor, like atrial fibrillation or is not on antiplatelet therapy or is not on or anticoagulation, that sort of thing—I think I probably would initiate a workup, almost close to, as if—you know, I would initiate a cardiac workup on those patients.

Now if it’s more of silent, subcortical lacunar-appearing stroke, I think if the patient was not on any antiplatelet, I certainly would recommend that they be on antiplatelet therapy. But those patients I probably would not chase as much. Like I wouldn’t feel strongly about doing an echo or anything on those patients, for example. Yeah.

**Q:** For these two categories, are there particular tests that you would pursue for them?

[00:11:37]

**A:** I think for a, for again, for the embolic type of stroke, I think definitely if they hadn’t had vascular imaging, I would do vascular imaging. And I think I would—I probably wouldn’t do a 30-day Holter, or a 30-day cardiac monitor. But I would probably do like a 48-hour Holter or something or at least refer them to their primary care physician for their evaluation that--

**Q:** What type of vascular imaging could you typically pursue?

[00:12:03]

**A:** Yeah. I probably would—I generally would probably do a—I probably would do an MRA of the head and neck.

**Q:** And, I guess you are a specialist but besides, within neurology, do you tend to refer these patients to anybody in terms of specialists or kind of ask for help?

[00:12:29]

**A:** I think probably—yeah, I generally do not, I think, unless I was worried about—either, unless the workup later on did have clinical findings, which I think might be more germane, be handled by a stroke neurologist or refer to a neurosurgeon or whatever for endo-vascular issues. Generally no. I probably would keep those patients. I guess the other exception, too—this hasn’t happened very often, but I guess young patients I might—now that we have an excellent, young stroke neurologist in the practice, I probably would refer those patients out as well.

**Q:** I forgot to ask you for the patients with the more subcortical or acute [00:13:10] appearing silent strokes, if you do any tests for them or if you just kind of-

[00:13:15]

**A:** I probably—yeah, those I probably—yeah. I probably let those go a little bit more. Again, if they weren’t on antiplatelet therapy I would put them on it. But I probably wouldn’t—yeah, I probably wouldn’t do any additional testing for those patients.

**Q:** Do you recommend any behavioral changes or life style modifications for patients when you identify silent strokes?

[00:13:33]

**A:** Definitely. Well, you know, definitely if they’re smokers, I strongly recommend they stop smoking. And I’ll use the finding of the silent stroke as addition impetus for them to, hopefully, motivate them to stop smoking. And also, just other, also, just in general, just be more compliant. If there are issues with high blood pressure, that still needs to be under control, just encouraging them to be more compliant with their primary care physician to address those issues.

**Q:** So it seems like what I’m hearing is from—in terms of your approach, oftentimes it depends on your assessment of what you think is the etiology or the mechanism is of these infarcts. Besides that mechanistic approach, are there other sort of, patient-specific factors that might influence your approach?

[00:14:26]

**A:** I guess age might also—I think age might also make be a little bit more aggressive or [00:14:33] me to refer out for further evaluation. So like, yeah. I can’t really recall an instance when I found an incidence of stroke in what I would describe in a young patient under the age of 50. I think that would--

**Q:** Oh, are you are saying if it’s a younger person that you might be a little more aggressive?

[00:14:50]

**A:** Yeah. I might be more aggressive. Yeah.

**Q:** You kind of expect it-

[00:14:53]

**A:** Yeah. There might be—right. Right.

**Q:** Moving to a little bit of a different topic about the radiologic imaging itself, how do you approach the radiologic imaging? For example, do you typically rely on the radiologist report or do you review them images directly or do you use a combination of the two?

[00:15:13]

**A:** I do a combination. I mean I always review all my scans whenever possible. So I’ll review it directly, also, with input from the radiology report. But I always look at my own scans.

**Q:** Are there particular ways in which the language that the radiologists use influence your decision making?

[00:15:36]

**A:** You know, I don’t—I don’t think so. I’m not saying that I’m not but I wouldn’t at least—I don’t consciously think, at least that it does. But there probably is some subconscious influence. I probably would expect that. But, at least I wouldn’t consciously make any changes based on what the report states.

**Q:** Okay. Do you speak with your patients about these imaging finding?

[00:16:03]

**A:** Yes, I do. Yeah. Yeah.

**Q:** How do you frame that discussion?

[00:16:08]

**A:** Well, what I do is I’ll mention that the scan that we did, looking, evaluating for the other problem found, incidentally, so not something that was expected but did show evidence that in the past they may have had a stroke. And I’ll go through with their history, just to make sure there wasn’t some correlation, like didn’t have some—maybe some TIA symptom that might clinically correlate it. But I’ll bring it up and I’ll say, “This might be something,” again, depending on, you know, depending on kind of what we talked about earlier, whether it looks kind of embolic or lacunar—but recommending that there might be some interventions. We do either additional testing or, for sure, if they weren’t on any sort of antiplatelet agent, I would certainly recommend that.

**Q:** Do you think that these silent strokes have caused harm to patients?

[00:17:06]

**A:** I guess probably I think it would be—I think it would be naïve to say no. So I guess probably it may have caused harm, either because they—even though they are clinically silent, but you have to rely on the patient’s recollection of symptoms. And quite possibly some of these strokes weren’t necessarily clinically silent. We just think they are. So then I guess you could say delayed evaluation. And it might contribute to, especially, if they have multiple, clinically silent strokes that might contribute.

And as we know, stroke burden can also lead to, you know, down the line cognitive issues and things like. And white matter changes certainly correlates with underlying neurological health in the long run. So I think probably in the long might potentially cause some harm. Yeah.

**Q:** I think you’re kind of getting into this idea of long-term issues. But in your perspective, do you—how much do you think silent strokes put patients at risk for other health issues?

[00:18:10]

**A:** Well, I think—I mean certainly I think a silent stroke that previously was not evaluated earlier, I think that delay in evaluation probably does put them at risk of future stroke. So I think there is some risk of that. In terms of health issues, probably nothing else that I can really think of more directly but definitely a delay in an evaluation, if they are someone who had a stroke and is at risk for—not on secondary prevention. So they are at risk of future strokes. I think that delay in evaluation could cause some problems down the line.

**Q:** Expanding a little bit on this topic of sort of risk and risk of other health consequences, I’m actually going to give you a list of health conditions and see what your thoughts are in terms of, if you think that silent strokes place your patients at risk for any of the following.

**A:** Okay.

**Q:** That is kind of the framework of the question.

**A:** Oh, okay.

**Q:** And again, there is no like—they can be yes or no some qualified answer in between that. So I think you already answered the first one, which is, do you think silent strokes put your patients at risk for stroke, symptomatic strokes.

[00:19:25]

**A:** Yeah, definitely.

**Q:** Do you think silent strokes put your patients at risk for intracerebral hemorrhage?

[00:19:34]

**A:** I don’t think so.

**Q:** How about heart attack?

[00:19:41]

**A:** I think in the sense that a clinically silent stroke, if they had no prior history of heart disease, you know, since the risk factors overlap, I think then that could indicate—not that the stroke itself would put them at risk but indicates that they have risk factors for heart disease.

**Q:** So there are sort of shared risk factors.

[00:19:58]

**A:** Yeah, it is like a correlation—more like a correlation rather than a causation.

**Q:** How about for heart failure?

[00:20:07]

**A:** I would say no.

**Q:** And how about high blood pressure?

[00:20:13]

**A:** Again, I think it is more of a, probably a correlation rather than a causation. But I would certainly imagine that patients with clinically silent strokes are far more likely to have also high blood pressure, uncontrolled blood pressure.

**Q:** How about high cholesterol?

[00:20:30]

**A:** Yeah, very—exactly similar to what I mentioned with high blood pressure. I think it indicates that they may have a shared risk factor.

**Q:** And diabetes?

[00:20:38]

**A:** I think. But I think probably less so compared to the other two.

**Q:** About them being a correlation?

[00:20:44]

**A:** Correlation. Right. Yeah.

**Q:** And then how about headache?

[00:20:51]

**A:** You know, I think it may be more that, since we do a lot of these scans, I think a lot of patients who have incidental findings of silent strokes, usually they have scans because of headache being a very common complaint. So again, I think there may be a correlation. But whether—I’m not sure there is a causation.

**Q:** How about seizures?

[00:21:11]

**A:** I think potentially there could be—again, if it’s more—particularly, these—particularly cortical silent strokes could put them at high risk for seizures. I think, yes.

**Q:** How about falls?

[00:21:22]

**A:** Yes.

**Q:** And memory loss?

[00:21:25]

**A:** Yes. I think so, too.

**Q:** Dementia?

[00:21:28]

**A:** Yes.

**Q:** Anxiety?

[00:21:33]

**A:** No, I don’t think so. Yeah.

**Q:** Depression?

[00:21:36]

**A:** No.

**Q:** And schizophrenia?

[00:21:41]

**A:** Well, I think, again, that I would also just qualify that. Just saying that there may be a correlation rather than a causation because those patients probably are less likely, like I said, interfacing with the medical establishment. So strokes would be missed or they may not be diagnosed, delayed diagnosis.

**Q:** Fair enough. How comfortable do you feel treating patients with silent strokes?

[00:22:06]

**A:** I think I’m reasonably comfortable. I mean I’m not a stroke neurologist but I feel like I have experience with managing patients with strokes and chronic, neurological illness. And I have an excellent—like I said, support system, colleagues that I can always refer to. So I’m pretty comfortable.

**Q:** Are there any practice guidelines that you follow for this issue?

[00:22:28]

**A:** Not specifically for clinically silent strokes I have to say. No.

**Q:** What do you think are the major knowledge gaps in this area?

[00:22:37]

**A:** Yeah. I guess, you know, I guess, you know there’s a lot of what I do, at least I try to, I correlate with patient—my experience when I’m treating symptomatic patients—so I guess there are some gaps in whether—one thing I ask, maybe I’m not being aggressive enough, for example, with certain types of patients. So maybe if there is some experience or clinical data that show that perhaps we should be more aggressive with these patients, in terms of working up clinically silent strokes—just like maybe how the paradigm shifted in terms of TIAs being, working them up more aggressively. Maybe we should be doing the same thing with clinically silent strokes. So I guess that needs to be determined.

**Q:** Are there other things that you would want to know more about besides kind of the approach, the aggressiveness of approach?

[00:23:25]

**A:** Yeah. Yeah, I mean I guess—I guess it’s also what we do, what about patients who have clinically silent strokes but who are already on secondary stroke treatments, I guess maybe. You know, do we start thinking about anticoagulation or something? I mean I don’t know. I’m just sort of throwing out there. But yeah, I guess what we would do in case of patients who have prior history of strokes but then have additional stroke findings like clinically silent strokes.

**Q:** So we’re getting pretty close to the end. This is the last sort of set of questions. And it’s a little bit of a thought experiment--

**A:** Okay.

**Q:** --To see how your practice might change given information from new studies that haven’t been done yet.

**A:** Sure.

**Q:** So I’ll present to you a few ideas. The first is, let’s imagine that there’s a big, sort of rigorous observational, comparative effectiveness study that is performed to determine the effectiveness of different prevention therapies for silent strokes. And let’s say actually the study found, actually, the opposite of your usual practice. So in terms of like effective or least harmful treatment decisions, for example, for let’s say the subcortical stroke patients you might start them on an aspirin. But it turns out that their bleeding risk is greater than the potential benefit of preventing strokes. Do you think that this type of study or a well-done, observational, comparative effectiveness study, would that be enough to convince you to change your practice?

[00:24:57]

**A:** Definitely would. Yeah. Absolutely.

**Q:** So do you think that would be enough in terms of action, without necessarily needing a randomized trial to do that?

[00:25:13]

**A:** I think so. I mean I think I would—I mean I think I would still love to see a more randomized trial. But I think that type of data, if there was a large enough observational study that demonstrated that, I think that would be enough for me to change my practice in that regard.

**Q:** Okay. If for some reason that type of study couldn’t be done or couldn’t be done well, and a randomized trial was performed comparing different treatments or looking at their efficacy and/or safety, how to you think patients should be recruited into that type of study, based on what you know in terms of how you encounter these patients?

[00:25:49]

**A:** Yeah. Well, you probably want to make sure that these patients—that, you know, that these patients with these clinical silent strokes are, you know, definitely clinically silent. It’s not just the patient just didn’t get evaluate for some symptom that they had, maybe. So I guess patients who had—I guess if you just had to recruit patients who—I guess for maybe—I guess if you just recruited them from a pool for patients with specific types of, you know, specific conditions like headache or something like that, you know, rather than dementia or things like where the recall might be as good. I don't know. That is sort of a weak answer.

**Q:** With regards to a randomized trial, would you have any concerns about feasibility of performing that type of study?

[00:26:43]

**A:** I don’t think so. Because I think probably it’s a pretty common problem. So I think you would have trouble recruiting patients for it.

**Q:** In terms of it, a randomized being a treatment trial, would you have any concerns about equipoise? For example, having some patients on aspirin and some patients not started on aspirin?

[00:27:02]

**A:** Yeah. Well, again, I think one—yeah, I think one—you mean concerned about just that some patients are not treated, you know?

**Q:** Yeah.

[00:27:11]

**A:** I guess no. I mean because we really don’t know. So I guess I wouldn’t. Yeah.

**Q:** What do you think would be the important outcomes to assess in either type of study?

[00:27:27]

**A:** I think I guess you just want and endpoint maybe until the patient’s next cardio, or the patient does have a clinical, you know, cerebral vascular event maybe—so maybe duration until the cerebral vascular event, something like that.

**Q:** Sort of like a survival analysis timetable?

[00:27:51]

**A:** Yeah. Yeah. Yeah. Time to event. Right.

**Q:** Okay. And the last part is, let me give you a few statistics that we do know so far. So there have been several large cohort studies, mostly in older individuals, like the Framingham Heart Study, the Cardiovascular Health Study, Northern Manhattan, Rotterdam, and so on. These are big, large prospective cohorts that have actually looked at the prevalence of silent strokes. And pooling the data together on average, in individuals over age 50, the prevalence of silent strokes is about 20 percent.

**A:** Oh, wow!

**Q:** And it starts out lower, in the sixth decade around—between 50 and 60 it’s somewhere—for example, in Framingham Heart Study it is about 7.5 percent. And after an additional decade it is 15 percent. After an additional decade it goes to 25 to 30 percent and it just increases from there. What we also know from these studies is that silent strokes are associated with a two to fourfold, relative increase in the risk of symptomatic stroke as well as dementia. So knowing those two things in terms of the prevalence and at least a couple of the outcomes, if we were to find a, at least a modestly effective prevention strategy for these two outcomes, or even just one of them—do you think it would be worth implementing some sort of screening strategy for silent strokes? And if so, how?

[00:29:26]

**A:** Yeah. Well, I mean I think a targeted screen strategy might makes sense. Like again, if you had patients who already had, you know, cardiovascular risk factor or stroke risk factors or reach a certain age, maybe, that could be a cutoff—yeah. I guess if you had a targeted type of approach—because presumably it would entail neuroimaging and things like that. It would be kind of cumbersome to neuroimage like every patient once they hit a certain age.

So I think if there was a targeted study that maybe within that 20 percent, whatever, if you found that there was a particularly high risk group, you know, or risk factors that would contribute to that, you know—like that would make you a more particularly at high risk—trying to break that down I think would be helpful.

**Q:** Okay. And do you have any additional thoughts or questions on your mind as we’re coming to the end of the interview?

[00:30:25]

**A:** Not really. I mean I think it’s, obviously, a very important topic that, yeah, it would be good to know, to have some additional guidelines for.

**Q:** Well, thanks. I’ll stop the interview and the recording right now.

**A:** Okay.

END OF INTERVIEW

Internist 6

Study ID: IN6

**Q:** This in our interview with our sixth internist. Can you say hello?

**A:** Hi.

**Q:** It’s just the sound check. So I’ll go through the questions. There are questions that I ask everybody. There are actually 13 of them. And depending on how talkative you feel I might kind of ask some more focused questions as well.

**A:** Okay.

**Q:** So the first question is just very open ended, it is, just tell me what you know or think about silent strokes so far.

[00:00:27]

**A:** What I know, so usually discover incidentally on brain imaging. So, you know, sometimes see a patient, review it. After I size up that where people never have a history of stroke or symptoms of stroke then I will categorize or diagnose that as a silent stroke.

**Q:** Are there particular terms that you’ve heard or that you’ve used to describe this? I know we are using the term silent stroke but have you heard any other terms?

[00:00:57]

**A:** Like by other people?

**Q:** Hm-hmm. Yes.

[00:01:00]

**A:** Not a specific term that I can think of right now.

**Q:** So I guess the literature often describes it in a number of different ways, which I think can be a little confusing. But sometimes people will describe these as silent strokes, silent brain infarction, silent cerebral infarction, covert stroke, covert brain infarction, subclinical stroke, subtle stroke or asymptomatic stroke. Have you heard any of these used before?

[00:01:28]

**A:** Yes, all of them.

**Q:** All of them at some point of time?

[00:01:30]

**A:** Yeah. Yeah. Yeah.

**Q:** Fair enough.

[00:01:33]

**A:** Right. And can I put like patients sometimes call it mini stroke. I don't know what that means but, yeah, that’s a very common term that you hear.

**Q:** Which do you use most often, if you had to describe this?

[00:01:48]

**A:** I want to say silent stroke or asymptomatic infarction. Yeah.

**Q:** Are those the terms that you think kind of fit best in terms of describing this or do you think there is another term that would be sort of most appropriate or most useful?

[00:02:04]

**A:** I mean like in terms of layman terms I think silent stroke is a pretty—not [?] everyone understands what silent stroke is. But for a professional level I feel like silent infarction is a pretty good term. Yeah.

**Q:** Why do you think silent strokes occur?

[00:02:25]

**A:** Why?

**Q:** Yeah.

[00:02:28]

**A:** I think most of the time is caused by—I mean people with a lot of vascular risk factors, caused by chronic vascular, poorly controlled vascular risk factor—yeah.

**Q:** Do you think, from your perspective do silent strokes have similar or different pathophysiologies to symptomatic strokes?

[00:02:57]

**A:** To be honest I’m not sure. I’m thinking of the same way that—for symptomatic stroke I’m thinking most of them are caused by kind of embolic, phenomenal embolic event compared to silent stroke, maybe more of a microvascular problem.

**Q:** Okay. So the mechanism you think might kind of sway in one direction or another.

[00:03:24]

**A:** Right. Yeah.

**Q:** Okay. Do you think there are different types of silent strokes? If so, like how do you categorize them?

[00:03:37]

**A:** Never think about that.

[Laughter]

Yeah. I mean the only thing is just the location, in terms of how to categorize by mechanism, no, not that I know of.

**Q:** Okay. So if you had to sort of kind of have some granularity you would just say where it is.

[00:03:55]

**A:** Right.

**Q:** Left side or right side.

[00:03:57]

**A:** Right. Right. Exactly.

**Q:** In your mind, in your perspective, how are silent strokes similar or different from white matter disease?

[00:04:10]

**A:** How are they similar--?

**Q:** Or different.

[00:04:12]

**A:** Or different? To me I feel that it’s kind of a different spectrum of the same, underlying mechanism, like white matter disease that are just kind of chronic, ongoing, micro [?] vascular problem, not to the extent that is causing an infarction. Right. But silent stroke is at the other extreme of, which is worsening microvascular event that is severe enough to cause an infarction.

**Q:** For you are they similar or different in terms of their clinical significance?

[00:04:51]

**A:** I feel like if I see a silent stroke I would probably be a little bit more aggressive in treating the potentially vascular risk factor compared to just seeing a white matter disease on a CT scan or brain imaging.

**Q:** What do you think are the major risk factors for silent strokes?

[00:05:14]

**A:** I would say all the typical risk factors including high blood pressure, hyperlipidemia, diabetes, smoking.

**Q:** And these are all things that you would address with your patients?

[00:05:29]

**A:** Well, if I see a silent stroke on top of that I would do an investigation just like how you treat a symptomatic stroke patient.

**Q:** Okay.

[00:05:42]

**A:** If it is as silent infarction that I see on a CT scan, I will probably do a full work up just like how I treat a symptomatic stroke.

**Q:** Would you do the same with people with white matter disease or not necessarily?

[00:05:55]

**A:** No.

**Q:** No. Okay.

**A:** Yeah.

**Q:** Fair enough.

[00:05:57]

**A:** White matter disease probably just focus on vascular risk factor, rather than do a full, like Carotid [?] or Echo or things like that. Right?

**Q:** Yeah. I might ask you a little bit about that afterwards.

[00:06:09]

**A:** Sure. Yeah.

**Q:** I was wondering, what sort of settings or scenarios have you encountered this diagnosis. Like what are the patients coming in with or like where are they when [simultaneous conversation]

[00:06:22]

**A:** Right. Yeah, I mean actually I just have a one patient recently who I saw with my resident, who came in with, just to establish care. And then when we go to the history there is no mention about stroke. But then, when you review their prior brain imaging there is signs of infarct—well, it was documented, saying there is infarction is the conclusion of the MRI brain. Yeah, that was one of the patients that we had just recently.

**Q:** What did you do for that patient?

[00:06:52]

**A:** So we basically tried to get more—because it is a relatively new patient to us we tried to get more record. And then we are going to do a full workup for him, actually. It depends on what kind of workup he has done and things like that.

**Q:** How did you communicate that to him?

[00:07:11]

**A:** Right. So basically we tell him that he, based on the brain imaging he had the stroke before and it can be caused by different reasons. And we are going to do a couple of tests to find out what is the reason and to see whether we can do more to prevent another stroke to happen. And then we are just going to aggressively treat all those vascular risk factors for him.

**Q:** Do you recall what his reaction was like to that message?

[00:07:35]

**A:** Well, he was pretty surprised about that because I don’t think anyone had told him about this. So, yeah, he was kind of like new information that he received.

**Q:** Besides this patient are there other sorts of situations in which you’ve encountered this?

[00:07:58]

**A:** Well, most of the cases I encountered in the clinic, where you see incidental, like brain imagining, many, many years ago that mentioned about infarction somewhere that the patient wasn’t aware of until you review it. And you told them and then, yeah. So I have a couple of patients.

**Q:** Okay.

[00:08:17]

**A:** Yeah, similar cases, yeah.

**Q:** Where you’ve gone back and reviewed the available data and the reports and then you’ve seen the reports that it mentions that.

[00:08:25]

**A:** Right. It’s most of the time happened during like physical exam, annual physical where you review some of the previous record. Or if they come in with a headache, then they review when was the last CT scan. Then you would see that. Yeah.

**Q:** Do the patients ever present with any symptoms and then you obtain a scan and the you find this incidentally? Or had it mostly just been this retrospective review of things that were done before?

[00:08:50]

**A:** Yeah. Most of the time it is retrospective review of—yeah.

**Q:** Okay. So that’s kind of informing this next question. But it’s more about the concept of incidental. So it’s kind of like taking a—going a little bit on an aside but oftentimes these silent strokes are encountered incidentally when physicians are trying to investigate some symptom or medical problem that doesn’t overtly seem related to stroke. But then they discover that a stroke has occurred at some point in time. For you, what does it mean for something to be incidental and how do you approach incidental findings?

[00:09:29]

**A:** Well, for incidental findings, you know, I will manage it in a non-urgent way. So, you know, instead of getting a full workup within a week, you know, I feel like there’s time that I can kind of take time, have patient to come by and have a test done one-by-one. So, yeah, that’s how I manage incidental finding or any kind of imaging.

**Q:** So it does affect the level of urgency.

[00:09:56]

**A:** Yes.

**Q:** It is usually something they can take time with.

[00:09:59]

**A:** Right. That’s what I do. Yeah.

**Q:** Going back to the question of the types of things you do for patient with silent strokes, what are the—do you usually start treatments of modify treatments for these patients?

[00:10:19]

**A:** Yes. So if they have signs of silent stroke and they are not on like anti-platelet and things like that, at least I’ll give them baby aspirin while doing the work-up for them—to find out any other potential causes for it. And definitely, risk modification depends on whether they have a underlying history of hyperlipidemia, diabetes, or high blood pressure. I might be more aggressive in terms of controlling the blood pressure and the lipids. But, yeah.

**Q:** Are there particular tests that you order?

[00:10:54]

**A:** I would do symptomatic stroke work-up.

**Q:** What sort of things are part of your—

[Laughter]

[00:11:02]

**A:** So depends on the area that is involved. You know, we will probably want to look at that vasculature in the neck and the brain. It depends on the underlying heart history. Might consider a Halter monitor, echocardiogram, what else, and then the routine blood tests, screen for lipids and diabetes and things like that. Yeah.

**Q:** Okay. Do you ever refer these patients or do you usually kind of embark on the investigation on your own? It sounds like you know that to do.

[00:11:42]

**A:** Fifty-fifty. Not necessarily all the time I’ll refer to a neurologist. If I have a question, I’m not sure that I’m doing the right thing, then yeah.

**Q:** What are those things that might call things into question? What are the situations where you might want to ask for help?

[00:11:58]

**A:** I would say if all the workup, everything is negative, and then he doesn’t have tons of risk factor for it, then I will probably send to a neurologist to figure out why he has this silent stroke, right? Or if there is a question about antiplatelets, for example, if patients are already on baby aspirin, so what would be the next step in terms of silent stroke.

**Q:** And do you ask your patients to make any life style changes or behavioral modifications?

[00:12:29]

**A:** Yeah, for sure.

**Q:** What sort of things?

[00:12:33]

**A:** So diet and exercise, those are the two components. Even with people with no silent stroke I would say the same thing. [Laughter] So, yeah, basically—yeah, so counseling on diet and exercise for 20 minutes every day, five days a week. Yeah.

**Q:** You mentioned the presence or absence of risk factors in terms of helping to kind of modify that approach. Are there any others, patient-specific factors that influence your approach or help to kind of individualize--?

[00:13:04]

**A:** Weight-wise is a big thing as well. What do you mean, in terms of--

**Q:** In terms of your approach and how you might select tests or who you might select to refer, who you might be more aggressive about medications or life style modifications.

[00:13:22]

**A:** Right. So I guess patients who are a little bit younger, good functional status, definitely would be more aggressive in terms of everything in terms of looking for a cause, like control all the risk factors really more tightly—than an elderly person who has kind of less functional, has less life expectancy, even with a silent stroke—I might not be as aggressive in younger people.

**Q:** How do you approach the radiologic imagining? And to kind of clarify that, do you tend to rely on the radiologist report or do you look at the pictures or do you do some combination of the two?

[00:14:04]

**A:** I don’t look at the picture. I just look at the report.

**Q:** And in terms of the radiologist’s language, how does that influence your decision making?

[00:14:17]

**A:** Well, if they say infarction in it [Laughter]—I mean if they just say microvascular or white matter disease or chronic microangiopathy, then, yeah.

**Q:** I think it’s great when they do put down their sort of five cents and say like, “Yeah, this is a brain infarct or this is a stroke or this is microvascular disease.”

[00:14:42]

**A:** Right.

**Q:** But sometimes, too, they’re a little bit more, they may say like probable infarct or possible infarct but could also be a myelinating disease versus migraine versus vasculitis. What do you do in those situations?

[00:14:58]

**A:** So I would say, it depends on the differential they gave me and the patient’s underlying history and clinical end story. I would try to definitely, to make sure there is nothing bad going on like in terms of acuity-wise like if it is a vasculitis or infection, things like that. So that, I would try to rule that out first. And then clinically not consistent with those, then I would focus more on to figure out if this is really just—is an infarction of that last acuity. So I do [00:15:32] the bad thing first. I mean infarction is bad, too. But if there [00:15:36] is something old then we have time to figure that out. And then a lot of times, if you are not sure, it is not going [?], then I will just send to a neurologist.

**Q:** Actually, out of curiosity, does this usually—have you mostly encountered these with head CTs or brain MRIs?

[00:15:57]

**A:** To be honest, I can’t—I want to say CT because most of the people—yeah. I want to say CT.

**Q:** You mentioned one example, which I think is really nice about how you would, how you approach this patient recently and how you communicated the findings. So you usually communicate it in a similar way for all patients? Or does it vary for some reason or another?

[00:16:22]

**A:** Similarly. Yeah. I mean I just really present the fact and I tell them what are the possibilities and what are the bad things that we don’t want to miss—and that we are just going to do all these tests to find out what happened. Yeah.

**Q:** Are there situations where you might not tell them about the finding or where, for some reason, it didn’t come up? No?

[00:16:41]

**A:** No.

**Q:** You feel pretty good about telling them about it?

[00:16:45]

**A:** Right. Yeah. And a lot of time when you tell them it is something that is seen [?] old, doesn’t look like acute, most of the people would—I mean it is a big diagnosis for them. But then, again, usually they are able to take it pretty well, as long as I explain to them clearly.

**Q:** Do you think that these silent strokes have caused harm to patients? And if so, how?

[00:17:13]

**A:** Well, I think it is definitely a red flag, that if we see a silent stroke in the brain it means that they have, you know, consider vascular disease that, you know, another stroke can happen or another major, vascular event can happen, like a heart attack because they are probably prone to that. So I think definitely is something that we have to focus on to make sure that we can reduce the risk, right?

**Q:** So having a silent stroke is a marker of all of these things brewing that might lead to--

[00:17:47]

**A:** Right.

**Q:** --Symptomatic stroke or a heart attack.

[00:17:49]

**A:** That’s what I think. Yeah.

**Q:** Are there other things that having silent stroke might put people at risk for?

[00:17:56]

**A:** I’m sorry?

**Q:** Are there other things that you think silent strokes might put people at risk for or increase the risk for?

[00:18:01]

**A:** Oh, well, it can definitely increase the risk of vascular dementia as well. So, yeah, is something to monitor if they have any memory complaint, yeah. Screen for dementia is probably important in those populations.

**Q:** I’m actually—so, I’m going to kind of spend a little bit on that part of the question.

[00:18:22]

**A:** Sure.

**Q:** I’m going to give you a little bit of a laundry list of just different conditions and symptoms and things. The framework of this question is, do you think silent strokes put patients at risk for any of the following?

[00:18:33]

**A:** Okay.

**Q:** So some of these you’ve already answered but, for example, do you think silent strokes put your patients at risk for stroke?

**A:** Yeah.

**Q:** How about intracerebral hemorrhage?

[00:18:45]

**A:** Yes.

**Q:** No right answer or wrong answer.

[00:18:48]

**A:** Yeah. Yeah.

**Q:** Just kind of best guess.

[00:18:49]

**A:** I was just thinking, actually.

**Q:** Heart attack or myocardial infarction?

[00:18:53]

**A:** Yes.

**Q:** How about heart failure?

**A:** No.

**Q:** High blood pressure?

[00:19:01]

**A:** I mean, you mean, the silent stroke associated with high blood pressure or put them at higher risk for high blood pressure?

**Q:** Either one. You can kind of--

[00:19:09]

**A:** Yes.

**Q:** You can give a conditional answer.

[00:19:10]

**A:** Yes. I think it is associated with—right.

**Q:** Associated with like a risk factor.

[00:19:15]

**A:** Yes.

**Q:** Okay. How about high cholesterol.

[00:19:18]

**A:** Yes, it’s associated.

**Q:** Same thing, associated.

**A:** Right.

**Q:** Diabetes?

[00:19:22]

**A:** Yes. Association.

**Q:** Associated. How about headache?

[00:19:29]

**A:** No.

**Q:** How about vertigo?

[00:19:33]

**A:** I guess it depends on the area, if it is not posterior, maybe?

**Q:** Okay.

[00:19:38]

**A:** Yeah.

**Q:** How about seizures?

[00:19:44]

**A:** I want to say it depends on the size of the infarction. I guess it is asymptomatic, most of the time it is small, I would say most likely not.

**Q:** Fair enough. How about falls?

[00:19:57]

**A:** I don't know. No.

**Q:** How about memory loss?

[00:20:00]

**A:** Yes.

**Q:** Dementia.

[00:20:02]

**A:** Yes.

**Q:** Anxiety.

[00:20:06]

**A:** No.

**Q:** Depression?

[00:20:08]

**A:** No.

**Q:** And schizophrenia?

[00:20:11]

**A:** No.

**Q:** Okay. And then, how comfortable do you feel treating patients with silent strokes?

[00:20:22]

**A:** Pretty comfortable.

**Q:** Are there any particular practice guidelines that you use? And if so, which ones?

[00:20:32]

**A:** No guidelines that I’ll pull it out. But to be honest, if I have any questions, I will first go to, up to the [00:20:41] to see what are the guidelines that, you know—but not that I can think of right now. I don’t go straight to a guideline.

**Q:** Sure.

[00:20:50]

**A:** Yeah.

**Q:** From your perspective, what do you think are the major knowledge gaps in this area?

[00:20:56]

**A:** Silent stroke?

**Q:** Yeah. So like what types of things would you want to know more about or what do you think we don’t really know much about yet?

[00:21:06]

**A:** Well, I’ve been doing like the work-ups of symptomatic stroke. I’m not sure if I’m doing the right way or am I overdoing it, because given that this is a silent stroke. But I’ve noticed that a lot of patients with all these findings that they are not aware of and they are not being informed by the previous doctors before. So I’m not sure whether we are undertreating these silent stroke patients.

**Q:** As a whole.

[00:21:35]

**A:** As a whole. Right. Yeah.

**Q:** And the last part of the interview, it’s a little bit of a thought experiment.

[00:21:45]

**A:** Sure.

**Q:** So the ideas is to get, to think a little bit about what sort of new data or new information would alter your practice or affect your practice.

[00:21:53]

**A:** Okay.

Q And so let’s imagine that a new study comes out. It’s a rigorous, observational, comparative effectiveness study, a big, sort of prospective study. And the purpose of it is to determine the effectiveness of prevention therapies for silent stroke as well as the benefit / risk ratio. And what if the study actually found that the opposite of your usual practice was true. So, for example, putting patients on an aspirin may actually increase the risk of hemorrhage more than preventing a subsequent stroke. If that were the case, would that type of study be enough to alter your practice?

[00:22:34]

**A:** Yes.

**Q:** It would be. Okay.

[00:22:35]

**A:** Yes.

**Q:** Do you think you would need a randomized trial to tell you that or would the comparative effectiveness study--

[00:22:41]

**A:** Well, you would make me think twice about aspirin. And then I might refer to a neurologist more if there is a study that comes up that says that. That actually does bring the risk probably more than the benefit, then. I would probably think twice and I’m actually referred to neurologist more.

**Q:** Okay.

[00:23:02]

**A:** Yeah.

**Q:** To see if there are individual patients--

[00:23:04]

**A:** Right. Exactly.

**Q:** --Who would benefit.

[00:23:06]

**A:** Yeah.

**Q:** Okay. Fair enough.

[00:23:11]

**A:** Where definitely I think there is always a need for more studies to verify on things that we do on a daily basis. But, yeah.

**Q:** Okay. So if I’m hearing you correctly, a comparative effectiveness study could be enough to kind of cast on your practice. And you might not necessarily need a randomized trial to sort of completely clarify whether or not--

[00:23:35]

**A:** Right. It’s not necessarily that I will stop giving aspirin to people. But I would just think twice. I would be hesitant and I would probably, you know—yeah, I mean it is always—we try to do evidence-based medicine as much as possible. So what are the best studies out there that will--

**Q:** Oh, okay. So for this type of study, whether it’s comparative effectiveness or a trial, what are the outcomes that you think would be most important?

[00:24:06]

**A:** I would say the incidence of recurring stroke and mortality.

**Q:** And then this last part, what I’m going to do is I’m going to give you a few facts, a few known statistics. So there have been several, large prospective cohort studies in a number of places, like the Framingham Heart Study, the Cardiovascular Health Study, Rotterdam, the Northern Manhattan Study. And they’ve actually followed patients and screened them. They have actually done MRIs at specific time points and established a little bit of information about how common this is.

And it turns out that over age 50 the sort of estimate of how prevalent this is, is about 20 percent have a silent brain infarct. And it starts off at the lower end. Sort of in the fifties it’s around seven and a half, eight percent. And then it climbs to 15 percent a decade later and the up to like 25 percent a decade later and so on. So that’s one thing. It’s a common issue. The studies also established that the risk of symptomatic stroke after the discovery of silent stroke is about a two to fourfold increased risk.

The same thing for dementia, for dementias that have a vascular component, whether Alzheimer's or vascular dementia. It is a sort of two to threefold increased risk. So knowing that, in terms of how the prevalence and also the outcomes or the consequences of it—if we were to find some sort of effective, or at least modestly effective prevention strategy, do you think it is actually worth screening people for this?

[Laughter] I can’t really express your facial expression.

[00:25:45]

**A:** No. [Laughter] Okay.

**Q:** It is a little bit of a complicated question.

[00:25:51]

**A:** Right. So I guess a question is just like a guideline. So let’s say you screen positive for silent stroke. So what are you going to do next?

**Q:** So let’s say there was a prevention therapy. Let’s say we found out that aspirin or something would actually prevent stroke and maybe prevent cognitive decline. Would that warrant then kind of looking to see if we’re undertreating people with silent stroke, kind of like what you alluded earlier.

[00:26:22]

**A:** Right. Yeah. I think, you know, if we are going to change what we are going to do and it’s going to give a positive outcome, then we have to think about the cost versus, you know--

**Q:** Is that what made you a little hesitant, the cost?

[00:26:39]

**A:** Yes. And also, you know, the interval, when you are going to screen how, you know, do you do every year or do you do it, you know—or should you just put everyone have, you know, have high risk on aspirin and then just don’t screen them if they have no, big contraindication for baby aspirin.

**Q:** If it were up to you, what do you think you would do?

[00:27:01]

**A:** I would—so I guess it’s important to look at those, you know, those studies, those people who screen positive for it. I mean if they are underlying, obviously, they have a lot of vascular risk factors. By just treating those, even without the [00:27:20] infarct in the brain imaging, I mean if it is going to bring the same outcome, then there is no indication to really do a--

**Q:** so you think maybe you can—looking at somebody’s past medical history and their, kind of stroke risk factors--

[00:27:35]

**A:** Right. Right.

**Q:** You might be able to predict who is likely to have a silent stroke--

[00:27:38]

**A:** Right. Exactly.

**Q:** --And just put them on treatment--

[00:27:40]

**A:** Right. If we can use those [dose ?] data and come out with a scale calculator, that might be more feasible than screening everyone. Yeah.

**Q:** Sure. Okay. That makes sense. So that’s it. That’s actually the end of the interview. I want to see if you have any additional thoughts or questions that you would want to express.

[00:27:59]

**A:** No. [Laughter]

**Q:** Fair enough.

**A:** Let me stop the audio then.

END OF INTERVIEW

General Neurologist 4

Study ID: GN4

#### Q: I’m starting the recording right now with our fourth neurologist. Can you say hello?

#### A: Hello.

#### Q: Great. That was just the sound test. So, most of these questions are pretty open-ended, so feel free to answer them in whatever length, or detail you’d like, and then I’ll try, and fill in around that. The first question is just to have you tell me about what you know about silent or covert strokes so far?

#### [00:00:28]

#### A: Do you mean their affect on patients, or –

#### Q: Just like your general thought process about them.

#### A: Most people – a lot of strokes do not have any clinical manifestations. People don’t even know they had any deficits for some of these small strokes, but they do indicate risk of future strokes, so, if we do find them it is important to address the potential risk factors for having future strokes.

#### Q: There are a bunch of terms that are actually thrown around both in clinical practice, as well as in the medical literature to refer to this phenomenon. Some people have called them silent strokes, or silent brain infarction, silent cerebral infarction, covert stroke, covert brain infarction, subclinical stroke, subtle stroke, or asymptomatic stroke. Which of these terms have you heard used?

#### [00:01:26]

#### A: Silent stroke, I’ve heard, subclinical stroke I’ve heard used. I think that those are the only ones that you mentioned.

#### Q: Which do you use most often when you’re talking to people about them?

#### A: I don’t think I use a general term like that. I would just say, “You had what looks like a stroke on your imaging that did not have any clinical manifestations. You’ve probably not been aware that you had it.”

#### Q: Fair enough. Do you think, kind of going forward, just to simplify things, do you think there’s a most appropriate, or useful term?

#### [00:02:03]

#### A: I think the term “subclinical stroke” is pretty accurate. We use the term “subclinical” a lot when there’s no very critical manifestations. I’m not sure how much patients would understand the term “subclinical.” I think for patient care “silent stroke” probably is the most accurate, so that they would be able to understand.

#### Q: Why do you think silent strokes occur?

#### [00:02:29]

#### A: I would assume that they’re due to small vessel disease. They’re deep strokes that don’t have any clear clinical manifestations. They’re usually in parts of the brain that there’s not much affect on clinical function, but they’re too small, and too deep, or affecting parts of the brain that aren’t really able to manifest clinically.

#### Q: You’ve kind of answered this question, I think, but just to kind of expand on that a little bit, from your perspective, do you think that they have similar, or different pathophysiologies from symptomatic strokes? And in what way are they similar or different?

#### A: Well, I think they’re probably similar to a lot of the small vessel strokes, and it depends on just where on the brain the stroke occurs. If it’s the limbic, or the basal ganglia, those have a lot more clinical manifestations, potentially, than strokes, parts of the [front area 00:03:30] that don’t have much clinical output. But I think a lot of it is based on location, because the pathophysiology is probably similar to a lot of the symptomatic strokes.

#### Q: Do you think there are different types of silent strokes, and if so, how do you organize them, or categorize them in your head?

#### [00:03:54]

#### A: Subtypes? I would think most of them probably are small vessel related. I would find it hard to have an embolic stroke not causing any deficits, usually because of the amount of territory that embolic strokes involve. But it’s certainly possible. If I saw one, I would apply a big workup tool without any other potential etiologies, and not just small vessel disease. Did that answer the question?

#### Q: It does. It does. Yes, it sounds like you’re kind of thinking about the mechanisms underlying the strokes, as your way of kind of categorizing them?

#### [00:04:28]

#### A: Right.

#### Q: From your perspective, how are silent strokes similar, or different from white matter disease?

#### A: There’s probably not much difference. When you say “white matter disease,” you mean microvascular disease? Or do you mean like any kind of white matter disease that’s related to that?

#### Q: Yeah. White matter disease that assumes to be related to some sort of vascular issue?

#### A: I think they’re probably along the same pathophysiological realm. And I think that sometimes we do these MRIs on patients, and you see extremely small areas of restricted diffusion. And some people aren’t sure if they’re acute strokes, or they’re artifacts. In my opinion, probably a long list of those same kind of a continuum between small vessel disease, and [micro plate managers 00:05:26] used in stroke.

#### Q: Do you think they’re similar, or different in terms of their clinical significance?

#### [00:05:36]

#### A: Probably not.

#### Q: For you, do you tend to react to one, but not the other? Or do you react more or less the same?

#### [00:05:47]

#### A: So, if you do an MRI, and you see there’s such a diffusion in an area, I probably would complete more so a short workup, even if the exact location didn’t really correspond to any of the patient’s symptoms for getting the MRI, versus if we do a CT scan/MRI, you see old white matter diffused, I probably would make sure that they were on an aspirin, and a statin, but I wouldn’t necessarily do an echocardiogram, or more so, a [certain image 00:06:15].

#### I would probably get echo imaging just to make sure there’s no vascular disease that’s contributing. If there’s a lot of white matter disease, I would do that, but if not, that was just a little help with a few slots.

#### Q: So, it sounds like the acuity of the lesion on an MRI scan can maybe affect how aggressively you would do testing for this?

#### A: Right. So, definitely if there was something appeared acute with the certain diffusion change, and I would do more workup, just because there’s obviously if they’ve had a stroke, then it’s higher risk, potentially, of having a larger stroke within a few days or weeks of having a single event.

#### But I would want to be more aggressive, versus finding a lot of old disease on an MRI, but I would still probably do some workup, and recommend treatment, but I wouldn’t be more aggressive in at least in the initial stages.

#### Q: What do you think are the major risk factors for silent strokes?

#### [00:07:20]

#### A: So, I think it’s a cerebral vascular risk factor, so high blood pressure, or diabetes, high cholesterol, smoking.

#### Q: In was sort of settings, or scenarios have you encountered people with this?

#### A: With the silent strokes?

#### Q: Yeah.

#### [00:07:40]

#### A: A lot of times they’re in either the inpatient setting, where they came in with some vague symptoms, that I don’t think corresponded to any vascular territory. You do an MRI, and you see a small stroke that probably doesn’t explain anything. I don’t think it was resulting in their symptoms.

#### Sometimes I’ve seen it in the outpatient setting when you do an MRI, or a CT scan for dementia, or some other headache, something nonspecific. And you get the MRI, and you see that they’ve had old strokes that they were never aware of.

#### Q: Yeah. For those inpatients are there particular symptoms that they do present with that you think are unrelated to the strokes?

#### A: Sometimes like people would present with ataxia, or they’ll present with like a visual field issue that not like it could have been an occipital stroke, and they have small, old strokes, and usually there will be white matter that would not be related to what the current symptomatology is.

#### Q: So, you end up attributing the ataxia, or the vision problem to some other non-stroke pathology irregardless of –

#### [00:08:56]

#### A: I think most of that, yeah. Well, a lot of time it could also be TIAs, and the symptoms are resolved by the time that they have the scan. And I’ve answered your question a few times. They come in with those symptoms. The symptom resolves in 24 hours. You do the MRI, something shows up in an area that’s not related to what I think the symptoms should be localized to. And I think it’s just an incidental finding.

#### Q: That actually leads me into the next question, which is that these silent strokes are often encountered incidentally when physicians are investigating a symptom, or medical problem that doesn’t overtly seem to be related to the stroke. Just kind of taking a step back, what does it mean to you for something to be incidental?

#### [00:09:50]

#### A: Incidental just means that I think it’s not related to the patient’s symptomatology. You were looking for your lesion in the occipital cortex, or the cerebellum, and you happen to find something in an area of the brain that would not produce the clinical symptoms that the patient is presenting with.

#### Q: You talked a little bit about this in terms of like how you respond to the findings, and kind of mentioned that there might be a little bit in terms of differences of approaches. In general, though, like do you end up starting, or modifying treatments for patients?

#### [00:10:31]

#### A: Who are set for these silent infarcts?

#### Q: Yeah.

#### A: Potentially, so if they have something. I think it kind of depends on what their initial presentation was, and what their initial symptoms were, and what they were doing before this was found. So, if they came in with a headache, and we found a small stroke in the deep white matter, yeah, I would probably just recommend that he take an aspirin.

#### And if they were taking aspirin already, I would probably either recommend increasing it, probably not switching to something else at that point, unless additional studies warranted management changes. But it would affect some decisions that I would make, mainly with regards to antiplatelet therapy, and statins, and then if additional studies are warranted.

#### Q: What sort of tests do you tend to order? What sort of is the range of tests?

#### [00:11:34]

#### A: It ranges usually from vessel imaging, if they haven’t had that already of the head and neck, and then if they’ve never had an echocardiogram, I might do one, just to get a baseline at that point to see if they have any cardioemboli risk factors.

#### So, my suspicion is that a lot of these silent infarcts are not cardioembolic in nature. And then, obviously, I’ll check a hemoglobin A1c, and I’ll look at that one, as well.

#### Q: For you, as a neurologist, you’re already specialist, but for these types of patients, do you ever get any other types of specialists involved?

#### A: Not unless I find significant carotid disease on their scan, and I see data on the ultrasound they need to see a vascular surgeon for the generation of surgery.

#### Q: If you found actually somebody with a silent stroke, and on the same side they had a pretty bad looking extracranial carotid artery, would you send them to a vascular surgeon for consideration for surgery?

#### [00:12:49]

#### A: I would probably have a detailed discussion with the patients about what the highly stenosed carotid would be, and their risk factors, and that the MRI, or some imaging already show that there is some risk for vascular disease, so I’ll make a decision about whether to proceed or not with the surgery, but I would consider implication as well. Yeah. It’s just a set up that has potential larger events in the future.

#### Q: Right. Do you ask your patients to make any lifestyle changes, or behavioral modifications?

#### [00:13:24]

#### A: Yes. Oh, yeah. If they are smoking I always tell them to stop smoking, and we talk about diet and exercise. But again, if they already have an 80 percent stenosed carotid, there’s only so much that that could probably do at that point.

#### Q: I think you mentioned a few of these things, but with regards to sort of individualizing care, it sounds like you may take an approach may vary a little bit, depending on some factors, especially like on the scans. Are there patient-specific factors that influence your approach, besides the scans?

#### A: Patient-specific factors for what? For management strategies?

#### Q: Yeah, exactly.

#### [00:14:11]

#### A: Obviously, it depends. If they have any medical comorbidities, what other risks might they have for strokes? Would they be at risk for having been started on antiplatelet, and [anticoagulants to stop 00:14:23] bleeding. But obviously, their medical comorbidities are a big factor in determining management after we find one, a silent stroke on imaging. That would probably be the main thing.

#### Q: How do you approach the radiologic imaging? So, for example, would you tend to rely on the radiologist report, or do you review the images directly, or do some combination of the two?

#### [00:14:54]

#### A: Well, I always review it myself, and then I review the report to make sure, (A), that there’s no errors in it. If the patient gets a hold of it there’s nothing incorrect on the report. But we also just see if there’s some agreement about what the images show, and pretty much my interpretation is, and what the radiologist’s interpretation is.

#### Q: Assuming that there aren’t any errors, how does the radiologist’s language influence your decision-making?

#### A: It usually doesn’t. The only time where it would influence it is if they specifically write in their report, “Repeat study should be done, or recommended in six months.” Then I’m legally bound to do a study at that point.” If something should come up, and the radiologist wrote in that report to do something, then I’d feel pressured to do it.

#### But other than if they specifically write that, I don’t tend to take much stock into their differential diagnoses, or other map, and report’s details.

#### Q: I think you’ve mentioned that you do describe these to some of your patients. Do you always describe them, or are there some situations where you might not immediately report them?

#### [00:16:09]

#### A: Explain what?

#### Q: It sounds like you do describe the silent strokes to your patients?

#### A: Yes.

#### Q: Or at least to some of them?

#### A: Yes.

#### Q: Do you generally report them to all patients, or are there sometimes reasons that you don’t?

#### [00:16:23]

#### A: No. If I find it on the imaging, I usually let them know that they’ve had a stroke in the past, or acutely depending on what the imaging shows.

#### Q: How do you frame that discussion with the patients?

#### A: I tell them that we looked at the imaging, and it looks like in the past they had a small stroke that they were likely unaware of. I let them know that not all parts of the brain have clear clinical manifestations, if there’s a stroke there, so that’s probably what happened. But that we should work this up as if it was something more significant, then we can prevent having larger strokes in the future.

#### Q: How do the patients, and their families typically respond to that?

#### [00:17:08]

#### A: Usually, they’re surprised. He never had any symptoms of a stroke, or, “I can’t believe he had a stroke.” But I think they’re usually pretty reasonable to try to do things to make sure that nothing happens in the future.

#### Q: Fair enough. In terms of the kind of idea of harm, and what these lesions might do, do you think that these silent strokes have caused harm to the patients? And if so, what sort of harm do you think they’ve caused?

#### A: If the silent strokes caused harm to the patient?

#### Q: Yeah.

#### [00:17:47]

#### A: Anytime that you damage brain tissue there’s a risk of other clinical issues in the future, and if you accumulate them they can lead to mood changes. They can lead to cognitive changes over time. So, it’s usually the buildup on these events that can lead to problems over time. A lot of times if there’s a silent stroke at that moment there’s not much immediate concern or damage. It’s usually the long-term that’s going to be [best 00:18:14].

#### Q: From your perspective, how much do you think silent strokes put patients at greater risk for other health issues?

#### A: Well, I think it’s a marker of vascular disease, whether it be cerebral vascular, or whether it’s cardiovascular disease in these patients, as well. So, again, I don’t think it’s a direct indicator of other disease, but it does show that an additional workup is warranted to make sure that there’s no significant cerebral vascular, or cardiovascular issues.

#### Q: I’m actually going to expand a little bit on that idea. So, what I’m going to do is I’m actually going to give you a list of different medical conditions, as well as symptoms to see if you think that silent strokes put your patients at risk for any of these? So, that’s the framework for the question. Do you think silent strokes place your patients at risk for any of the following? And you can say, “Yes,” or, “No,” or give some qualified answer in between that. So, the first is stroke.

#### [00:19:23]

#### A: So, yes, I think if you’ve had any kind of stroke in the past then you’re at high risk of having a future stroke.

#### Q: And then next is intracerebral hemorrhage?

#### [00:19:37]

#### A: No, I don’t think – the only way it would be related to intracerebral hemorrhage, so I would say, “Yes,” is that sometimes intracerebral hemorrhage, and strokes are obviously significant.

#### There are hypertensive issues, and so, if your blood pressures are elevated, and uncontrolled, you can get silent strokes, and you can also get intracerebral hemorrhages in the future. So, you don’t want to wait so that their blood pressures are well controlled.

#### Q: So, they have sort of shared risk factors?

#### A: Yes, exactly.

#### Q: How about myocardial infarction?

#### [00:20:08]

#### A: The same thing with shared risk factors. A lot of times, like I said before, the cerebral – the silent strokes are acting through the small vessel disease, which are due to high blood pressure, diabetes, a lot of times high cholesterol, which are the same risk factors for certain cardiovascular disease.

#### Q: How about heart failure?

#### A: Probably not directly that I’m aware of. I get heart failure lists over time, tapping into the high blood pressure, that’s some risk factor disease, risk factor similarities, and stenosis can lead to heart failure, and myocardial infarctions can lead to heart failure.

#### [00:20:52]

#### So, again, on some continuum there is a probably some shared risk factors between the two.

#### Q: And then high blood pressure?

#### A: I think high pressures are a potential risk factor for silent stroke, so I also think silent stroke is directly an indicator of high blood pressure, but it is something that usually is associated with it, and I would definitely make sure that blood pressures are managed well.

#### Q: Yep. How about high cholesterol?

#### A: Same thing. Yes. I think there is an association between the two.

#### Q: Diabetes?

#### A: Again, yes. So, not everyone with diabetes has silent strokes; not everyone with silent strokes has diabetes, but I do think that it is something that needs to be checked for, because of the association between the two.

#### Q: How about headache?

#### [00:21:44]

#### A: That’s an interesting question. And I think headache obviously can cause these T2-hyperintensities, and lead to a lot of migraine headaches, specifically, and we don’t really know what those T2-hyperintensities are in migraine. Are they vascular-related, or are they some other type of physiology we’re not aware of yet?

#### So, I would say it’s potentially undermined at this point. There may be some definite association in the future that’s realized, but at this point I don’t think there’s any direct association between the two.

#### Q: How about vertigo?

#### [00:22:20]

#### A: Yeah. Probably no direct association at this point, unless you have the silent strokes obviously in the [consideration 00:22:27] circulation. And in general, I would say there’s probably no relation between the two. But they’re potentially can be.

#### Q: How about seizures?

#### A: So, most seizures don’t come from deep in the brain, but it’s theoretically possible. But unless the silent stroke is in a [cortical 00:22:46] region, and again, most silent strikes are not, I don’t think there’s any direct association between the two.

#### Q: How about falls?

#### [00:22:58]

#### A: Falls? Sure, every time you can get – again, it’s kind of the number of silent strokes. If it’s an accumulation of these strokes in the deep, white matter that can lead to gait instability, and can lead to falling issues. But a single silent stroke, they’re probably not directly related.

#### Q: How about memory loss?

#### A: So, it’s again along those same lines. If you download accumulated silent strokes, accumulated white matter issues, then you can develop vascular dementia from that.

#### Q: And then the next one is dementia?

#### [00:23:39]

#### A: What’s that?

#### Q: Dementia?

#### A: Not just the [memory loss 00:23:42], but dementia? It’s all on the same continuum, I would say, so there can be a relationship there.

#### Q: How about anxiety?

#### A: Probably again, a single, silent stroke wouldn’t lead to anxiety issues. If there’s a lot of frontal disease sometimes, people do have anxiety as a manifestation of strokes, or probably more so depression, but I would say anxiety, as well. Again, I don’t think a single silent stroke would lead to this issue, but accumulations can potentially be contributing to it.

#### Q: The next one, actually, is depression.

#### [00:24:19]

#### A: Depression? Yeah, so it’s the same thing, I would say. And again, one single, silent stroke shouldn’t cause depression, but we do know there is an association between cerebral vascular disease, and depression, so I would say there probably is a relation there, or can be there.

#### Q: The last one is schizophrenia.

#### [00:24:40]

#### A: Schizophrenia?

#### Q: Yeah.

#### A: Yeah. At this point in time I would say no. We do know that if there is frontal disease, again, it can lead to schizophrenia. But I would say at this point in time there is no known single silent stroke, or even a few that should lead to that manifestation.

#### Q: Sure. The next question is how comfortable do you feel treating patients with silent strokes?

#### A: Well, I feel comfortable discussing it with patients about what it means, and then what official testing, or medications are necessary to try to help prevent this from happening again.

#### Q: Do you follow any particular practice guidelines, and if so, which ones?

#### A: I’m sorry? There are no known practice guidelines for silent strokes. I don’t follow any specific guidelines. I usually follow, if anything, guidelines for general stroke, and what the workup should be for those patients.

#### Q: So, like the secondary prevention guidelines?

#### [00:25:47]

#### A: The secondary ones, yeah.

#### Q: From your perspective, what do you think are the major knowledge gaps in this area?

#### A: Well, I think, (A), we don’t know for certain what the pathophysiology is on silent strokes, and we don’t know what the clinical outcome is of the accumulation of the silent strokes, and how closely they are associated with this regular white matter disease. And if there is a difference in outcomes between patients with white matter disease, and silent strokes.

#### Q: Are there things that you’d want to know more about, or things that you feel uncertain about?

#### [00:26:29]

#### A: I want to know what long-term outcomes are for people who have silent strokes now. I want to know what medications are optimal for creating silent strokes, and preventing reoccurrence, and what risk factors in addition to the ones we know about already, such as smoking, and unless they’re on diet. Is there anything else that we should be aware of?

#### Q: So, we’re coming pretty close to the end of the interview. There’s just like one last section, which is a little bit of a thought experiment to see how your practice might change, given information from new studies?

#### A: From new studies?

#### Q: From new studies. So, this is kind of hypothetical. So, let’s imagine that there’s kind of a big rigorous observational comparative effectiveness study that was performed to determine the effectiveness of prevention therapies for silent strokes.

#### [00:27:25]

#### And let’s imagine, actually, that the study finds that the opposite of your usual practice was either the most effective, or the least harmful treatment decision. So, let’s say that you’re describing that you think most of these strokes are probably small vessel disease. You might put them on an aspirin, or like make sure that they’re not on an aspirin.

#### But let’s say it turns out that actually the bleeding risk exceeds the benefit from preventing further symptomatic, or ischemic strokes. So, would this type of study be enough to convince you to change your practice, or would you need more than that?

#### [00:28:03]

#### A: It’s hard to make a large-scale practice decision from a single study, usually you’d want to see replication of data to make sure that before you make any large decisions at this point we know that the aspirin is effective for strokes, and small vessel disease.

#### So, I wouldn’t make any major decisions off of one study, but if repeat studies did show that the bleeding risk with aspirin outweighs the benefits of it, then obviously I would consider that.

#### Q: Do you think it would matter? Let’s say if there are multiple studies, would it matter if they were observational studies, versus clinical trials? Do you feel very strongly like there has to be clinical trials, or would like a number of observational studies that are all showing the same thing, would that be enough to convince you to make a change?

#### A: Yeah. If enough observational studies show the same data, a lot of time clinical studies just can’t be done, because of risk, or regulations. But if a lot of clinical, valid observational studies were done, and its data was implicated, then I would consider it.

#### Q: Just to kind of extend that thought a little further, let’s imagine that instead of a comparative effectiveness study, somebody was doing a randomized trial. How would you try and recruit patients into such a study, or how do you think they would be best recruited?

#### [00:29:42]

#### A: [Say that over again 00:29:42]?

#### Q: How do you think patients could be recruited into a randomized clinical trial for this particular issue.

#### A: How would they?

#### Q: Yeah.

#### [00:29:51]

#### A: Were they at finish, or just for the silent strokes?

#### Q: For silent strokes. Like let’s say you’re trying –

#### A: For silent strokes?

#### Q: Yeah.

#### A: Oh, I would definitely refer people to our tertiary care center where the studies are taking place to start with. And I would tell them about the risks of having future strokes, potentially, and the studies that we’ve done to try to limit their future risks. Would they be willing to participate in that kind of study?

#### But I think, obviously that would be done at a tertiary center, and we would look forward to your support staff. I wouldn’t be able to do that from my office, yeah.

#### Q: Fair enough. Fair enough. With regards to NRCT, do you have any concerns about feasibility, or about –

#### [00:30:44]

#### A: Feasibility?

#### Q: Yeah.

#### A: No. About feasibility? Not at this point. Obviously, they’re getting harder and harder to do, as a result of the regulations. But I think it’s still feasible to do randomized trials with these patients.

#### [00:31:05]

#### Obviously, the main risk would be – if you are studying something like using an aspirin and a statin for some of the medications, you’re potentially putting at risk, when they don’t receive those medications to have future events. That would be one potential risk. If you’re properly treating some patients, potentially.

#### Q: Yeah. So, that leads me to my next question, but do you think that there are any concerns about equipoise in doing a randomized trial? For example if you’re randomizing patients with silent stroke to aspirin, or no aspirin, do you think that’s reasonable, or perhaps not reasonable?

#### A: Yeah. That’s why say I don’t think it’s reasonable at this point in time. I think if they have known that that’s the risk factors to not give an aspirin is probably malpractice, but I wouldn’t recommend doing that in the study.

#### Q: That sounds good. What do you think are the important outcomes to assess in either type of study?

#### [00:32:05]

#### A: In a theoretical study? It would be recurrent silent strokes on imaging, obviously any clinical events. [Of a price 00:32:17], obviously clinical outcome of morbidity, and mortality, obvious cardiac endpoints. So, it will cerebral vascular endpoints, cardiac endpoints, stroke, MI. Those would probably be the main things.

#### Q: And the last thing I’m going to mention, I’m actually going to give you a few known statistics, so there have actually been several large cohort studies due to prospective cohorts, and in those studies, they actually did some screening of brain MRIs, so they just picked a time point in year X of the study, and in year Y, they just scheduled people for a brain MRI.

#### [00:32:52]

#### So, these are studies like the Framingham Heart Study, the Cardiovascular Health Study in North Manhattan, Rotterdam, and so on, all with like several thousand patients . And what they found pooling all that data together is that in individuals over age 50, the prevalence of silent brain infarcts is about 20 percent.

#### And they also found that in terms of clinical outcomes that silent brain infarcts are associated with about a two- to four-fold increase in the risk of symptomatic stroke. And a two- to three-fold increase in the risk of dementia, the types that have some sort of vascular component related to them, vascular or the Alzheimer’s, or the mix.

#### So, knowing that, if we were to find some sort of prevention strategy, even if it was just modestly effective, do you think it would actually be worth screening for silent infarcts?

#### A: Yes. If you can prevent accumulation of these infarcts, and potentially prevent vascular dementia, or other future strokes, then certainly it’s something that can improve morbidity in the future, and obviously that would help with patients, or help with saving healthcare dollars in the future. So, I think it is worthwhile.

#### Q: If so, so in terms of this type of screening, how would you imagine it being done?

#### [00:34:15]

#### A: I would imagine people getting at least an MRI, probably, at age 40 or 50 to see if they have any white matter burden, or have any history of silent strokes. And also, obviously, checking risk factors for these kind of strokes, if they’re looking at their vessels, checking their hemoglobin A1c, and checking their blood pressures, and cholesterol levels, and seeing if they’re at risk for these events.

#### Q: Sounds good. So, we’re actually at the end. Do you have any additional questions or thoughts about this issue?

#### [00:34:49]

#### A: I think it’s an important issue that you’re looking at. There are no clear practice guidelines at this point, and no clear knowledge of what these incidents may needs in the future. So, I think it’s a good study that you’re doing. I think that’s great.

#### Q: Thanks. I’m actually going to stop the recording right now, and then just mention a couple of more things to debrief.

#### [00:35:12]

#### A: Sure.

#### Q: Give me one second.

#### END OF INTERVIEW

Internist 7

Study ID: IN7

#### Q: So, this is our interview with our seventh internist. Can you say hello?

#### A: Hello.

#### Q: So, I’m just going to put this a little bit closer to you, just to make sure that it picks up on the audio. Most of these questions are pretty open-ended, so feel free to answer in as much depth as you like.

#### And if I need a little bit more information I’ll kind of add some additional questions onto that, to get a broader sense for how you think about this, and how you approach this. So, the first question is just for you to tell me what you know about silent stroke so far?

#### [00:00:36]

#### A: I guess not much. It can be linked to other vascular disease, so hyperlipidemia, diabetes, and it varies for MIs, or other vascular disease indicators for silent strokes. It may be asymptomatic.

#### Q: And in terms of how people describe this in clinical practice, or in the literature, there are actually a bunch of different names that people have used. Some people have called them silent strokes, silent brain infarcts, silent cerebral infarction, covert strokes, covert brain infarction, subclinical stroke, subtle stroke, and asymptomatic stroke. Which of these terms have you heard used?

#### [00:01:19]

#### A: Sorry, say those again?

#### Q: There are a bunch of them. There’s silent stroke, silent brain infarction, silent cerebral infarction, covert stroke, covert brain infarction, subclinical stroke, subtle stroke, and asymptomatic stroke.

#### A: I think silent stroke, and asymptomatic stroke.

#### Q: Good. And are there particular terms –

#### A: Sorry. One quick question.

#### Q: Sure.

#### [00:01:44]

#### A: Just semantics. You mentioned chronic ischemic changes. Are you counting that as –

#### Q: I’m actually going to get to that.

#### A: Oh, okay, good.

#### Q: I’m going to ask you about that, but that is a common question that people have in terms of trying to figure out what you see on a radiology report, and how that fits in.

#### A: Okay.

#### Q: Are there actually any other terms besides the ones that I mentioned that you’ve heard used? And you mentioned chronic ischemic changes, and wondered about that?

#### [00:02:08]

#### A: That’s probably the only other.

#### Q: Okay. Are there particular terms that you use most often when describing these to others?

#### A: Silent stroke.

#### Q: Do you think amongst these terms there’s a most appropriate, or most useful term to use?

#### A: I think silent stroke probably makes the most sense, something that wasn’t picked up that happened.

#### Q: Sure. Why do you think silent strokes occur?

#### [00:02:45]

#### A: I think, like I said before, I think anything that puts you at risk for atheroslerotic disease, or vascular disease can increase your risk for a stroke. So, I think either increased risk with smoking, or diet, or obesity, diabetes, metabolic syndrome.

#### Q: So, do you think they’re more or less similar in terms of pathophysiology to symptomatic stroke, or do you think there are any differences?

#### [00:03:14]

#### A: I think you can probably say that they’re more likely in the setting of hypertension, or smaller blood vessels that don’t cause – not big distributions.

#### Q: Larger sort of arterial territories that are affected?

#### A: Yeah.

#### Q: Do you think there are different types or categories of silent strokes, and if so, how would you organize that yourself?

#### A: I have no idea. [Laughter]

#### Q: Do you differentiate between them in any way in terms of how you approach them?

#### A: No.

#### Q: This is kind of getting to your prior question, but from your perspective, how are silent strokes similar or different from white matter disease?

#### [00:03:59]

#### A: I have no idea. I think white matter disease is more nonspecific, so I think it could refer to a lot of different things. So, I guess that would be the only thing. If it says “white matter disease,” I don’t think I would interpret that, necessarily, as stroke.

#### Q: Fair enough. Do you think that white matter disease, let’s say it’s described by the radiologist as being chronic microangiopathic changes, or something along those lines. Do you think that that has a similar, or different clinical significance to silent stroke?

#### [00:04:36]

#### A: Yeah. If it says chronic microangiopathic disease, I’m thinking more along the lines of blood flow issues, or hypertension, or something that is leading to blood flow issues.

#### Q: Would you react to one but not the other?

#### A: I’d probably more likely react to chronic microangiopathic changes.

#### Q: Oh, really? Okay.

#### A: Yeah.

#### Q: Fair enough. Why is that?

#### A: Again, like I said, the white matter disease is so nonspecific, so, when I see it I’m never sure how to interpret that.

#### Q: Oh, okay. So, I’m sorry, so you would react more to the language of chronic microangiopathic changes, but not if it just says “white matter disease?” Is that what you’re saying?

#### A: Yes, yes.

#### Q: Sorry, I wasn’t specific enough with my question, but what I meant was let’s say you’re comparing one person who has something that says that there’s an infarct?

#### [00:05:29]

#### A: Oh, yeah, I would react to that.

#### Q: But there’s no like clinical connection to that, like a clinical syndrome or symptom, versus someone who has a scan, again no clinical symptoms, but the report says something about chronic microangiopathic changes? Would you respond differently to those two?

#### [00:05:47]

#### A: Yeah, I think the infarct would be more concerning to me.

#### Q: Fair enough.

#### A: I don’t know if it’s meant to be, but I think that terminology would be more concerning.

#### Q: Sure. Fair enough.

#### A: I don’t know. When I’ve talked to radiologists before I’m like, “What does that mean “chronic microangiopathic change?” A lot of them cannot give me a straight answer, like, “Oh, this is this, versus this.” So, I don’t know if it’s like a bucket term for them, either.

#### Q: What are some of the things that you’ve heard them say?

#### [00:06:17]

#### A: Other than that? Or like when I ask them to explain what it is?

#### Q: Yeah. When you ask them to explain.

#### A: A lot of like, “Oh, nonspecific change,” or, like, “Not territorial,” so nothing specific that they can give me.

#### Q: Fair enough.

#### [00:06:31]

#### A: So, I think the infarct is like more committing to something, more of a diagnosis.

#### Q: Fair enough. It sounds like you have maybe in mind some things that you might do if it says “infarct,” but do you ever respond if it says “chronic microangiopathic changes,” or just kind of leave it alone?

#### A: I make sure that their neuro exam is okay. I make sure that if they haven’t had a lipid panel checked, to check that. This is not evidence-based, but if they’re not on aspirin, and there’s hypertension, or another comorbidity, I might consider aspirin, but I don’t think it dramatically changes how I manage them.

#### Q: Sure, sure. Fair enough. I think you kind of talked about this already a little bit, but just in case anything else comes up, what do you think are the major risk factors for silent strokes?

#### A: I think smoking, diabetes, other vascular disease, in other places peripheral vascular disease, and coronary disease, hypertension.

#### Q: Fair enough. What are the types of settings or scenarios in which you’ve encountered people with this?

#### [00:07:56]

#### A: A head scan done for another reason, like a headache, or migraine, or some sort of other vague symptom, and it’s mentioned incidentally, or dementia workup.

#### Q: Dementia?

#### A: Um hmm. [Indicates, "Yes."]

#### Q: Do you encounter this both in the hospital, as well as in the clinic?

#### [00:08:13]

#### A: Yes.

#### Q: Do the reasons differ from one to the other?

#### A: On why they were scanned?

#### Q: Yeah.

#### A: No, not really.

#### Q: Not really? It could be any [simultaneous conversation 00:08:21]?

#### A: I guess coming into the hospital, they’re more likely to have had trauma, or something along those lines. But not really.

#### Q: Fair enough. And do you ever end up connecting what they came in with to the finding on the scan, or is it always completely separate?

#### [00:08:41]

#### A: No.

#### Q: It doesn’t really explain it? Okay. So, taking a step back, thinking about the idea of incidental findings, which we run into all the time, and with different types of tests, what does it mean for something to be incidental for you? Like how does that –

#### A: You weren’t looking for it, and you found it.

#### Q: Does that affect like how –

#### [00:09:02]

#### A: And it doesn’t relate to the presenting symptom that the person had.

#### Q: Right. Does the fact of something being found incidentally affect your thinking about it, or your approach to it?

#### A: It depends on what it is. If it’s an incidental finding of like a lung mass, that would be a lot different, but I guess if it’s not causing symptoms, and if it’s a small incidental finding I would be less likely to jump on it right away.

#### Q: For silent strokes, as a specific type of incidental finding, do you feel some sort of obligation to respond to them?

#### A: Not really.

#### Q: Not really? Okay. Fair enough.

#### A: Yeah. I mean, again, making sure that they’re being adequately treated for vascular disease, but –

#### Q: Okay. I’m going to kind of explore that idea just a little bit more, like even if it’s not for everybody, do you ever end up changing medications, or adjusting them?

#### [00:10:00]

#### A: I think it would mostly be adding an aspirin, or a statin if they need it, and other indications for it. It’s never prompted me to get carotids, or an echo.

#### Q: Fair enough. Are there any tests that you do, or not really?

#### A: For –

#### Q: If you see someone with a silent stroke?

#### [00:10:23]

#### A: They hardly ever use the terminology of silent stroke on that.

#### Q: Sure, sure.

#### A: So, I guess, if it says “chronic microangiopathic disease,” no not really other than making sure.

#### Q: If it says “infarct” on that? Let’s say it says chronic lacunar infarct in the thalamus, or something. Would that –

#### A: Yeah. I would probably be more likely to make sure that they’ve had things up-to-date, and listened to carotids, and consider an echo, and look for other causes.

#### Q: I’m sorry, when you say carotids, do you mean auscultating them?

#### [00:10:54]

#### A: Dopplers.

#### Q: Or doing an ultrasound?

#### A: Oh, auscultating. Yeah.

#### Q: So, listening to the carotids? You mentioned – sorry, what was the other thing that you mentioned?

#### [00:11:06]

#### A: Making sure they’ve had an echo.

#### Q: Having an echo?

#### A: Um hmm. [Indicates, "Yes."]

#### Q: And for these patients, do you ever end up getting specialists involved in their care?

#### A: We’ve had people who had other neurological symptoms that were not nonspecific, who’ve had these findings [whatever 00:11:27] for neurology, just in case, I guess. Yeah, if anything neurology.

#### Q: What sort of symptoms?

#### [00:11:39]

#### A: I’ve had people have like vague numbness, tingling, not in a set distribution that really isn’t explained by the findings, but considering they have that consideration.

#### Q: And you look for some input to see what to do about that?

#### A: Yeah.

#### Q: And then, do you ask your patients to make any lifestyle changes, or behavioral modifications when you run into this?

#### [00:12:04]

#### A: I make sure that their blood pressure is under control, and to talk to them about medication compliance, and that sort of thing. I just make sure that all of their lifestyle things are under control.

#### Q: Are there any patient-specific factors, sort of irregardless of what the radiology report says, if you find that there’s a an infarct of some sort? Are there any patient-specific factors that kind of modify your approach?

#### A: Yes. If I think they’re high-risk for vascular disease, then maybe a little bit more aggressive.

#### Q: What sort of things?

#### A: If they’re otherwise healthy, then maybe not as aggressive.

#### Q: Sure, sure. At risk for vascular disease, meaning already having a history of –

#### A: Meaning a history of other atherosclerotic disease, or smoking, or diabetes, or something along those lines.

#### Q: Fair enough. I think you’ve alluded to this already, but about the radiologic imaging, and the reports? How do you approach that? So, do you tend to rely on the radiologist report, or do you review the images directly?

#### [00:13:12]

#### A: I do not review the images directly, no. [Laughter]

#### Q: Fair enough. Fair enough.

#### A: Sometimes I’ll call the radiologist if I’m concerned, or confused, or what not, but no.

#### Q: And I think you’ve kind of talked a little bit about this, but I was going to ask you about how the radiologist’s language influences your decision-making, and it sounds like –

#### [00:13:31]

#### A: Yeah, again, if they commit to something more specific, then it’s more concerning to me than just chronic changes.

#### Q: So, it sounds like you said if they say “infarct,” then you might actually –

#### A: Yes.

#### Q: – think of it as being more meaningful?

#### A: Yes.

#### Q: What if they have qualifiers? Like what if they say it could be a possible infarct, or a probable infarct?

#### [00:13:54]

#### A: Yeah. That would be more concerning to me.

#### Q: It would be? Even if –

#### A: And if they gave me a location, like a distribution, rather than just, oftentimes it says like “scattered changes.” It doesn’t really give you a specific location, or it’s harder to interpret for me.

#### Q: So, the more definitive their language is, the more it would make you convinced that there is actually something going on?

#### [00:14:18]

#### A: Yes. Um hmm. [Indicates, "Yes."]

#### Q: Okay. Fair enough. Do you speak with your patients about these when you find them?

#### A: Yes.

#### Q: How do you frame that discussion?

#### A: I say, “There is nonspecific changes that could reflect maybe old areas. I don’t even know how to word this properly, too but, “There’s nothing like acutely going on in your brain right now, but there is a suggestion that maybe you’ve had disease before, but nothing that we need to acutely do right now.

#### Q: How have people responded to that?

#### [00:14:59]

#### A: They don’t say much. They’re like, “Okay.”

#### Q: They just kind of let it –

#### A: Uh huh. [Indicates, "Yes."] Roll with it, because I’m vague about it. So, they’re vague about it, I guess.

#### Q: Fair enough. Have you ever had people ask questions about that in response to delivering that news?

#### [00:15:16]

#### A: I’ve had people ask, “Oh, do I need to do anything in terms of medications, or take anything?” And my response is usually just, “You should keep your blood pressure in control, make sure that your cholesterol is okay, and just monitor your neuro exam closely, and make sure that everything seems okay. Just let me know if there’s any symptoms.”

#### Q: Sure. Fair enough. I always admire the work that you guys do, because I think it’s actually a lot harder than being a specialist of all this, because there’s so many things that you have to tackle.

#### A: There’s so many random things, yeah.

#### Q: And so, I can just imagine this just being like one out of eight different things that you’d have to approach in a visit, or if someone comes into the hospital?

#### A: Yeah.

#### Q: It’s just like a problem, some number of like six different things. Do you feel like you end up like on average telling people they’ve had this conversation most of the time, some of the time, all the time, sometimes but not all the time?

#### [00:16:14]

#### A: Usually, this is in the setting of getting a scan for something else, so I think more of the discussion revolves along the lines of, “You’re negative for a bleed, or an acute stroke right now,” so, rather than, like, “You have these little changes that …” –

#### Q: Yeah, fair enough. So, kind of is incorporated into that discussion?

#### A: Yeah.

#### Q: So, the negative result from the scan in terms of it being relatively normal that comes first?

#### [00:16:40]

#### A: Yes.

#### Q: And then you mentioned, “Oh, but there’s this finding.”

#### A: Um hmm. [Indicates, "Yes."]

#### Q: Fair enough. Kind of moving to a little bit of different subject in terms of clinical significance, do you think that these silent infarcts have caused any harm to your patients?

#### A: That’s a good question. None that I can definitively say. I’ve not had anybody go on to have a massive stroke later that I can definitely say, “Oh, they had this chronic [case 00:17:10] before.

#### Q: Is stroke the main thing that you’d be worried about?

#### [00:17:16]

#### A: Um hmm. [Indicates, "Yes."]

#### Q: Sort of a risk of having stroke afterwards?

#### A: Yeah.

#### Q: Are there other things that you think silent infarcts might put patients at risk for?

#### [00:17:32]

#### A: No. I mean, maybe dementia, or –

#### Q: Out of curiosity, in terms of putting people at risk for, let’s say, stroke or dementia, how big a risk do you think that is? Is it a little bit? Is it a lot?

#### A: I’d probably say mild to moderate, not –

#### Q: Fair enough. [Laughter] So, not as usual, but there’s –

#### A: Not severe, yeah.

#### Q: I’m going to spend a little bit on that. So, actually, what I’m going to do is I’m going to give you a list of different conditions. And some of them are medical conditions, some of them are symptoms, and I’m going to ask you sort of for each one do you think that silent infarcts place your patients at risk for X?

#### A: Okay.

#### Q: So, you’ve answered this already, but do you think it silent strokes put your patients at risk for symptomatic stroke?

#### [00:18:27]

#### A: Um hmm. [Indicates, "Yes."]

#### Q: How about intracerebral hemorrhage?

#### A: Yes.

#### Q: I should have specified. You can say, “Yes,” “No,” or give some qualified answer in between.

#### [00:18:38]

#### A: Okay.

#### Q: How about heart attacks?

#### A: Yes, just because of association with other vascular disease.

#### Q: Heart failure?

#### A: Yes.

#### Q: How about high blood pressure?

#### A: Yes.

#### Q: High cholesterol?

#### [00:18:56]

#### A: Yes. I mean, are you talking causal, or association?

#### Q: You can describe it if you think it’s an association.

#### A: Yeah. I think they’re more likely to have hypolipidemia, but not because they had that sense, or could because they’re more likely to be that picture.

#### Q: So, hypolipidemia is more likely to cause someone to have a silent stroke, or at least be associated with that?

#### A: Um hmm. [Indicates, "Yes."]

#### Q: And did you mean the same for high blood pressure, too?

#### [00:19:20]

#### A: Yes.

#### Q: And diabetes?

#### A: Yes.

#### Q: How about headache?

#### A: No.

#### Q: How about seizures?

#### A: I guess no.

#### Q: How about vertigo?

#### [00:19:34]

#### A: No.

#### Q: Falls?

#### A: No.

#### Q: How about memory loss?

#### [00:19:43]

#### A: Yes, potentially.

#### Q: How about dementia?

#### A: Potentially, yes.

#### Q: Anxiety?

#### A: Yeah. If the diagnosis is distressing to them.

#### Q: Fair enough. And how about depression?

#### A: No.

#### Q: And how about schizophrenia?

#### A: No.

#### Q: And then –

#### [00:20:01]

#### A: And so, have those been studied?

#### Q: Not all of them. I’ll tell you about that, though, afterwards.

#### A: Okay, yeah.

#### Q: In general, how comfortable do you feel treating patients with silent infarcts?

#### [00:20:13]

#### A: Not comfortable.

#### Q: [Laughter] Okay. Why don’t you feel comfortable?

#### A: Because I know what to do with an acute stroke, but again, sometimes depending on the terminology, sometimes I’ll be more aggressive than others. So, I don’t have like a clear other than that, “Oh, this is what I do every single time I see this.”

#### Q: Are there any for this scenario for silent brain infarcts, are there any practice guidelines that you follow?

#### A: No.

#### Q: And this maybe is kind of underlying some of the things that you’re describing in terms of feeling uncomfortable, but what do you think are the major knowledge gaps in the area?

#### [00:20:54]

#### A: I guess just semantics, in general, like what’s being referred to: How severe is it? How aggressive do we have to be?

#### Q: When you say “semantics,” are you referring to trying to figure out –

#### A: There are so many different ways. Yeah, it’s written as – I don’t know what it’s describing. Are they all the same? Are they different?

#### Q: And you’re talking about on the radiology report?

#### A: Yeah.

#### Q: So, the difference between an infarct, versus chronic microangiopathic changes?

#### [00:21:25]

#### A: Yeah.

#### Q: Versus white matter disease?

#### A: Yes.

#### Q: Or leukoaraiosis, or whatever they’re referring to there?

#### A: Yes.

#### Q: Fair enough.

#### A: And does it make a difference? I guess, are they all the same?

#### Q: Does it make a difference to distinguish between them?

#### [00:21:40]

#### A: Um hmm. [Indicates, "Yes."]

#### Q: Are there particular things that you would want to know about, or that you feel uncertain about, besides the differences between those?

#### [00:21:52]

#### A: I guess, what does it put the patient at risk for? Is it equivalent in risk to a TIA, or a stroke, or what’s the subsequent morbidity for these patients?

#### Q: We’re actually coming close to the end of the interview. This last section, though, it’s a little bit of a thought experiment. And so, the idea is to see how you’re practice might change if given new information from new studies, so studies that haven’t been done yet.

#### So, the first scenario is let’s imagine that there’s a rigorous observational, comparative effectiveness study that was performed to determine the effectiveness of prevention therapies for silent brain infarcts.

#### A: Say that again? Comparative?

#### Q: So, a rigorous observational comparative effectiveness study. So, it’s well done, but it’s an observational study. It’s comparing a couple of different options in terms of treatments, and the idea is that it’s looking to see what’s the effective, and safe prevention therapies for silent infarcts to, let’s say, prevent symptomatic strokes afterwards?

#### And let’s say the study actually finds that the opposite of your usual practice is the true thing, so let’s say maybe – well, actually, I’m not entirely sure where you are on the spectrum, but let’s say, maybe on average, 70 percent of the time you put people on an aspirin, but then you find out that actually it might cause more bleeding than actually help in terms of preventing ischemic stroke. Would this type of study actually be enough to change your practice?

#### [00:23:36]

#### A: Yes.

#### Q: It would be?

#### A: Um hmm. [Indicates, "Yes."]

#### Q: Do you think you would need like randomized clinical trials, or other things to help guide that decision?

#### [00:23:42]

#### A: It would be more ideal, but I think an observational study would be useful.

#### Q: If it’s well done?

#### A: Yeah.

#### Q: Fair enough. Just out of curiosity, if a randomized trial were done, how would you imagine patients could be recruited into that type of study, or how would you try to recruit patients into that study?

#### A: I guess any time they have an abnormal – and maybe the radiologist could e-mail the doctor.

#### Q: It’s like the radiology departments would sort of be put on alert that the study is available?

#### [00:24:20]

#### A: Yeah.

#### Q: And they would contact the doctor who ordered the study to talk to the patient about it?

#### A: Yeah. Because a lot of times it’s also done in the emergency room, or what not, and you don’t find out that that scan was done until later.

#### Q: With a randomized trial, do you think there’s any problems in terms of feasibility?

#### [00:24:47]

#### A: If there aren’t good practice guidelines, no, in terms of intervention versus none, I don’t think it’s unethical.

#### Q: That was actually going to be my next question, if you thought there was any problems in terms of equipoise during a randomized trial? So, you think it’s kind of fair game. It’s not entirely clear what’s the right path?

#### A: Yeah.

#### Q: Fair enough. For either type of study, what are the –

#### A: I think – oh, I think just getting patients. That might be difficult recruiting enough during an optimal time period, because it’s an incidental finding, so you have to have some sort of a scan before. I wouldn’t scan people just for the sake of –

#### Q: Sure, sure, sure. So, to find silent infarcts that are incidentally discovered, it just might not occur often enough in order to track them down, or complete a study in terms of enrollment.

#### [00:25:46]

#### A: Yeah. I mean, I hardly ever order CT or MRIs in clinic unless they’ve had a long-term headache, or something. It’s rare.

#### Q: Sure. Fair enough. For this type of study, what sort of outcomes do you think would be really important to look for?

#### [00:26:06]

#### A: I guess, obviously death, so mortality, but stroke, TIAs, MIs.

#### Q: So, the cardiovascular things?

#### A: Yeah.

#### Q: Symptomatic strokes.

#### A: Maybe dementia. I feel like dementia would be a long time period to be following up.

#### Q: Sure, fair enough. And the last thing I’ll mentioned: So, I’m actually going to give you a few known statistics, right now. So, most of what we know about silent infarcts comes from a number of large cohort studies: The Framingham Heart Study, Rotterdam, Northern Manhattan Cardiovascular Health Study, ERIC, and so on.

#### And they’ve given us a little bit of information about the prevalence, some of the clinical consequences, and risk factors. And the average estimate from these studies in terms of how come this is in individuals over age 50, for example, the estimated prevalence of silent infarcts is actually about 20 percent.

#### And it goes up with age, so it starts off, for example, at Framingham, which is right in the middle of these studies, starts off around 7.5 percent in people who are in their fifties, and then goes up to 15 percent in the sixties, 25 percent in the seventies, and so on.

#### [00:27:29]

#### What we also know from these studies is that silent infarcts are associated with a two- to four-fold increased risk of symptomatic stroke, and also an increased risk of dementia that about a two- to three-fold increased risk. And those are the main outcomes that people have found.

#### So, knowing that, if we were to find some sort of effective prevention strategy for, let’s say, stroke, and it’s maybe even just modestly effective, do you think it’s worth implementing some type of screening strategy, or trying to figure out who might benefit from prevention therapy?

#### [00:28:04]

#### A: Yeah. If it’s a 2 to 4 percent risk.

#### Q: Two- to four-fold increased risk, actually. So, that’s a relative – an increased risk. So –

#### A: Oh, sorry. Yeah, two- to four-fold, yeah. So, it’s pretty big?

#### Q: Yeah.

#### A: I think so then, yeah.

#### Q: How do you think that would be done, like a screening strategy? How would you try to accomplish that? Or how would you recommend that it be done?

#### [00:28:32]

#### A: That’s a good question.

#### Q: Or do you have any concerns about implementing screening of this type?

#### A: I think that people who are high-risk enough are probably already on preventative meds, so it’s really that intermediate class where you could consider starting in aspirin, or a statin. So, I guess it’s what level of risk is it worth it to screen?

#### Q: So, it sounds like there ought to be some way of stratifying risk, or figuring out who’s high risk?

#### [00:29:08]

#### A: Yeah.

#### Q: Who’s intermediate risk? Who’s low risk?

#### A: Um hmm. [Indicates, "Yes."]

#### Q: And I don’t want to put words in your mouth, but it sounds like if they’re high enough risk, they’re probably already on medications, or –

#### A: Already, yes.

#### Q: Or you might just go ahead, and start them on medications?

#### A: Yeah.

#### Q: But then there’s kind of a middle, kind of gray zone, where maybe we need some more information to of sway the decision-making?

#### [00:29:27]

#### A: Yeah, exactly.

#### Q: Yeah, that’s an interesting idea. I actually haven’t heard anyone mention that before. But that’s very interesting. Okay. Well, that’s it.

#### A: I don’t know, instituting screening, I don’t know in terms of scanning people, like at what level that would make sense.

#### Q: Yeah. A lot of people have expressed some concern about costs, of course, for doing brain MRIs on every person.

#### [00:29:57]

#### A: On every person, yeah.

#### Q: Is that something that would concern you?

#### A: Yeah.

#### Q: Do you have any other additional sort of thoughts or questions about this issue?

#### [00:30:08]

#### A: So, what do you know about it?

#### Q: Well, let me stop the interview, and I’ll stop the recording, and then I can tell you.

#### END OF INTERVIEW

Vascular Neurologist 4

Study ID: VN4

**Q:** So I'm starting the recording right now. Can you say hello?

**A:** Yep.

**Q:** Great. So this is our interview with our fourth stroke neurologist. And the questions are going to be relatively open-ended for the most part. So just feel free to answer with as much detail as you’d like. But if there's more stuff to explore, I might ask some more detailed questions.

The first question is just to tell me what you know about silent or covert strokes so far.

[00:00:38]

**A:** What I know about what? I'm sorry.

**Q:** About silent strokes.

[00:00:43]

**A:** Well, you know, we uncover them [?] uncommonly. I don’t have two years of data to quote on the actual incidents. I'm actually not even 100 percent sure how rigorously that’s been investigated. But I bet you would know better than I on that one. I know that we don’t have any guidelines on how to address these. I know that the scenario of inquiries, hence your study, is the one thing.

I know that there's some heterogeneity in how people approach these, and whether or not they're even willing to call them ischemic. Because you see, these abnormalities, which are often not specific and suggestive of ischemia, but I don’t know that that’s how everyone always concludes that.

[00:01:33]

And personally, I think I view it very differently in a context of somebody who has vascular risk factors or even subsequent vascular events than somebody who has a [00:01:47] finding.

**Q:** Great. So as you're describing, there's some heterogeneity, including actually heterogeneity in terms of how people talk about these, or how they refer to them. And so in clinical practice, and also in the literature, there are actually a number of names that this diagnosis has taken up, which include silent stroke, silent brain infarction, silent cerebral infarction, covert stroke, covert brain infarction, subclinical stroke, subtle stroke, and asymptomatic stroke. Which of these terms have you heard used before?

[00:02:31]

**A:** I mean [00:02:32] if I've heard all of them.

**Q:** Are there any in particular that you use, or that you think of?

[00:02:43]

**A:** Absolutely, probably silent strokes. I mean I know I never say covert strokes. If you said it I’d know what you were talking about. Yeah, probably silent stroke.

**Q:** Are there other terms that you’ve heard used to refer to this phenomenon?

[00:03:04]

**A:** Not that come to mind, other than what you’ve mentioned.

**Q:** And which do you think would be the most appropriate or useful term to use if we were trying to stick with just one?

[00:03:21]

**A:** I don’t have a strong opinion about that, to be honest.

**Q:** Okay.

[00:03:24]

**A:** I think if there's something that becomes so universally adopted, for simplicity’s sake, I would support unification[?] more than I feel strongly about any particular term. Does that make sense?

**Q:** Mm-hmm, fair enough.

[00:03:38]

**A:** But then I think that it’s always less helpful when we have a lot of terms for the exact same thing. But it’s kind of actually like, uh-oh, would you like to call it a sensory aphasia, word [00:03:49] aphasia, posterior aphasia, receptive aphasia, like they're all—I don’t know that one of them I’d favor. So I think of that as sort of analogous.

**Q:** Fair enough. You kind of alluded to this before, but I'm interested to hear your thoughts on this. But in your opinion, why do you think covert strokes or silent strokes occur?

[00:04:18]

**A:** I don’t think that there's a single answer to that. I think that it’s also, if somebody has an MRI, and then they have a sub-centimeter, but also not like just a single millimeter of white [00:04:38] T2 hyperintensity, I think that it’s very different than if I see, like, sort of a wedge-shaped atrophy in the parietal lobe. So I think of those as very different circumstances what people might call a silent stroke, if [?] they [?] didn’t have a clinical scenario with this.

[00:05:02]

So I think that there's a lot more uncertainty about what is causing, you know, white matter hyperintensities. Because we certainly know that, you know, many of them are nonspecific, but some radiologists may read them as strokes. Some of them may be related to [00:05:17] phenomena, some of them may be related to small vessel disease, versus, you know, you said you see territorial-looking hyperintensity or hypointensity, depending on the fidelity you talked about. Like you think a lot more about sort of other traditional circumstances, particularly in [00:05:35] or large vessels. So I don’t know. I think there's not an answer saying, “Oh, this is what causes silent strokes or covert strokes.”

**Q:** So it sounds like you think they might have multiple different types of causes.

[00:05:53]

**A:** Oh, definitely. And I don’t know if there's multiple types of causes.

**Q:** Just to kind of explore this a little bit further, from your perspective, do silent strokes have similar or different pathophysiologies from symptomatic strokes? And in what ways are they similar or different?

[00:06:15]

**A:** That’s a good question. I would say I'm honestly not sure. I would presume more alike than different, but I think that that is a question that I don’t know that we know the answer to.

**Q:** Why do you have doubts or uncertainty about that question? What gives you pause?

[00:06:50]

**A:** Well I'm not—So, to the best of my knowledge, all the major glucophiles[?] we’re looking at for most stroke treatments, and looking at [00:07:04] looking at etiologies, they're focused on clinically symptomatic strokes. So I am not aware of much of the literature on asymptomatic or silent strokes. I know there probably is more literature than what I'm aware of. But I would profess ignorance on that point, I guess.

**Q:** So just from not having seen this in the literature, that’s what gives you a little bit uncertainty as to whether or not they're the same or different?

[00:07:41]

**A:** Yeah. I mean the literature and also the more fuzzy—like I'm not sure that I've ever really been taught much about silent strokes. We talk about it, but not in any robust way or if anything, so this is how you work up or even considering the etiologies of strokes. So I know that not everybody does evaluate etiologies of patients that have strokes.

**Q:** So what if I was to tell you, just like we’re having a debate, that I think that silent strokes are all the same as symptomatic strokes? That a stroke is a stroke. There's no difference. It sounded like you feel like maybe there's a chance that they might be slightly different. How do you argue that point? Or what would you tell me to kind of dissuade me from being so certain about that?

[00:08:45]

**A:** Well, so rather than directly trying to dissuade you, I think I would just follow a question with a question to be annoying[?], ask, like, what are the rates of stroke recurrence in somebody who has a silent stroke versus a symptomatic stroke? Do they differ? Because I think that’s, as a bottom line person, I think that’s the biggest thing I would alter management of patients on how I view them down the line. It’s like, well how does this portend [?] future risk? Because by their definition, they're asymptomatic. I'm not so worried about how the patient is doing today, it’s how are they going to do in the future?

So that’s, is there a difference between them, is what I want to know. And I don’t know that I—I don’t feel that I know the answer on that.

**Q:** Fair enough. I think that’s a good answer. So this might be kind of hearkening back to what you said before about causes of silent strokes. But I was going to ask you if you think there are different types of silent strokes and how you would categorize them in your mind, or sort of sort them in a way that's meaningful to you.

[00:09:56]

**A:** I mean I think that there are. I mean I've sort of given examples of the peers, you know, peer total, which there's not as many places where you could put sort of a wedge-shaped hole and where you can put a little sub-centimeter white matter hyperintensity without having symptoms. But you know you talk about, especially not dominant temporal lobe, especially the more interior you get, you might not have a lot you notice there.

So I think the size and shape and location probably suggests that there's a difference between them. But again, I wouldn’t profess to have much certainty there.

**Q:** I'm sorry, you said size and location. Was there a third thing that you said?

[00:10:43]

**A:** Morphology.

**Q:** Morphology.

**A:** In addition to just the size. But I think that, you know, those are related issues.

**Q:** By morphology, you mean the shape?

**A:** Yes.

**Q:** Okay. Just as a follow-up to that, in your perspective, in your opinion, how are silent brain infarcts similar or different from what's sometimes referred to as white matter disease or leukoaraiosis?

[00:11:15]

**A:** [00:11:14] your question. Are they different? [laughter] I think that there's certainly more examples of each of them aren’t the same. But how do we tell what's different? So again, if I'm seeing somebody who has got some distinct white matter abnormalities that are asymmetric, I’d say that maybe suggest that it’s more likely to be ischemic, but not necessarily. Again, when you have these symmetric multifocal patterns, I think that you have some uncertainties that this could be ischemic or it could be other etiologies. So I think a lot of these patients are in leukoaraiosis, yeah, a lot of them have the chronic vascular risk factors and say, you know, [00:12:02] radiology or, you know, chronic, microvascular ischemia.

But I don’t know that we know that that’s the type of physiology. I think we [00:12:10] degenerative things. So I'm not sure, again.

**Q:** For you, do you think that silent brain infarcts and white matter disease, do you think they're similar or different clinical significance?

[00:12:32]

**A:** Say it again? I'm sorry?

**Q:** Sorry, I didn’t word that correctly. But do you think that silent brain infarcts and white matter disease are similar or different in clinical significance?

[00:12:47]

**A:** I think that we have a Venn Diagram issue here. Because some of those things are probably the same, and some of them aren’t. And some of them probably do have a clinical significant difference. And some of them are, you know, again, they're in that overlapped part of the Venn Diagram. But I don’t know how to tell those.

**Q:** Sure.

[00:13:08]

**A:** It’s not a helpful distinction, but it’s just a belief I have about something that I have probably have made.

**Q:** Okay. Would you react to one but not the other? Or would you react the same to both?

[00:13:25]

**A:** I think it depends a lot on clinical context. You know, if somebody has had other events concerning subacute [?] cerebral ischemia or certain cerebral ischemia, if somebody has vascular risk factors, I think your pretest probability comes into play strongly on how I would make any clinical decision absent any further knowledge about the technology which I don’t think I have.

**Q:** So if somebody has what you identify as cerebral vascular risk factors, would you tend to be—would you actually tend to do some sort of treatment or investigation for those patients versus not in the other situation if those were absent?

[00:14:12]

**A:** Yeah, I think if somebody has no traditional vascular risk factors and no history such as any other ischemia, because again, we’re saying that this particular lesion we don’t think is [00:14:21] to a clinical [00:14:24], right. That’s the condition[?] of an asymptomatic one. But if they come in with something that I think, “Oh hey, that sounds like TIA,” I don’t think [00:14:31] the first of that part of the brain at all. But this is something that points to things[?] that had other events, then yeah, I would. If they're coming in with potentially nothing, and I don’t know that I have a strong proclivity to do a workup or start treatments, solely based on your radiographic finding. [simultaneous conversation] Like some people have, like yeah, they have mild, easily treated hypertension in their late 40s versus somebody who had multiple MRIs, they had afib, they're smokers, you know, you don’t think of people as dichotomists. They have [00:15:13] or they don’t. People are different as to risk profiles.

**Q:** And you would take that into consideration for patients with either issue, either a silent infarct or white matter disease?

[00:15:36]

**A:** I think so. Again, I'm not sure how firmly I'm actually making this division. I think that, like, white matter disease, if I've made that declaration, I guess I'm saying I think it’s probably not a big deal. And if I'm saying it’s ischemia, I think it is. But, whether or not I'm even making that call is based on the patient’s clinical scenario.

**Q:** Fair enough, whether or not you even get to that stage of trying to really distinguish between the two.

[00:16:03]

**A:** Yeah, because I feel like as though, like, “Well, what do you think of this? Like [00:16:09] this scan, this scenario, this [00:16:10] this scenario?” Well, I'm calling the scan two different things, two different scenarios. I'm reading it not just as an image in isolation but an image in a clinical context, which the radiologists don’t have the advantage of doing most of the time. But that’s how I have to apply these things.

**Q:** Right. So you’ve actually kind of partly answered my next question, which is, how do you approach the radiologic imaging? And the clarification of that is, do you rely on the radiologists’ report? Or do you review the images directly? Or some combination of the two?

[00:16:44]

**A:** As you're aware, I always just review the images directly. I take the radiologist’s report into consideration, but happily and easily disagree, proportionate with my experience with that particular radiologist, and whether or not they're a general radiologist or a neuroradiologist. But I think, particularly when we’re talking about brain and ischemic changes in particular, that I should be able to think [00:17:07] assessments of an image.

**Q:** Fair enough. How does the radiologist’s language influence your decision-making, if it does at all?

[00:17:21]

**A:** Depends on the radiologist.

**Q:** Okay.

[00:17:23]

**A:** There is a neuroradiologist that I trust. And they use strong language that they declare ischemia. And I know that they don’t always make it declarative. But I take that differently than somebody saying ischemia. Let’s just say that if the general radiologist—and I don’t know anything about them, never read their [00:17:43] I pretty much toss it out. I just pretty much take my readouts entirely over theirs.

**Q:** Okay, fair enough. Taking a step back a little bit, what do you think are the major risk factors for silent strokes?

[00:18:00]

**A:** Again, I think that’s a good question. I would presume the risk factors are, you know, akin to traditional symptomatic stroke risk factors. But I think that it would be an interesting question to answer whether or not these people have different characteristics. I guess I would hypothesize maybe two risk factors in the silent folks, but I'm not sure.

**Q:** What do you mean by that, by fewer? Do you mean like in individual patients?

[00:18:32]

**A:** Again, a sort of off-the-cuff and not particularly robust hypothesis, basically, the more risk factors you have, the more likely if you're going to have an event, it’s going to be clinically apparent. But again, I don’t feel strongly about that.

**Q:** That’s an interesting idea. It’s kind of, if I'm hearing you correctly, it sounds like the number of, let’s say, vascular risk factors may indicate a certain risk profile or maybe have some effect on the—as you were kind of alluding to before, maybe like the risk of having recurrent events. And maybe if you have more risk factors, you're just more likely to have more events. And, probabilistically speaking, one of them is going to be symptomatic.

[00:19:23]

**A:** Or even that you don’t say that—Like even if you took the denominator question away from it, just it’s like, these people are going to have a single event. Who’s more likely to have it be silent? I think I can actually argue my way into both positions, where you say, “Oh, somebody with more risk factors, just [00:19:38] having larger and denser infarct, and therefore it’d be symptomatic.” Or I could, just in terms, say, “Well, they’ve been smoking for all these years,” and felt that it’s sort of probably [00:19:52] ischemia, they have a more robust collateralization to some. Therefore, they're more likely to have a silent infarct because they have a low density or low—or smaller sized infarct. It’s like, again, I think I’d consider arguing. A train is coming. This is very nice for an interview. [laughter]

**Q:** Sorry. You're near a train or something?

[00:20:16]

**A:** Yeah, I’m near some train tracks right now. So I'm up in Dade, but I'm not in the hotel room anymore.

**Q:** Oh, okay, I see. Fair enough.

**A:** I'm sorry. It’s a lovely day, other than the train.

**Q:** Just to kind of get it on the audio recording, so for those risk factors that you're describing, what are some of them that kind of come to mind as being most relevant for silent strokes? You mentioned smoking.

[00:20:44]

**A:** Well smoking, I think smoking and atrial fibrillation are the ones that move the needle pretty far. I think hypertension, as well, just [00:20:52] that stuff that we [00:20:55], likely to call the chronic small vessel disease. I guess I think of it less with sort of atherosclerosis or large vessel stuff. I don’t know. I have this belief that I don’t really have any foundation with. You know, that’s more likely to cause a symptomatic stroke. But I think that, you know, a-fib, you think about the scenario of just small infarcts that aren’t necessarily a place where you're going to have a lot of symptomatology, versus you're smoking and hypertensive, you think of a micro vascular side of things. So that comes immediately to mind.

**Q:** Okay. In what sort of settings or scenarios have you encountered this phenomenon? Like what did patients come in with, or what did they get scans for, or what were you seeing them for?

[00:21:54]

**A:** Well, I mean, it sort of [00:21:56] inpatient and outpatient settings, because all of a sudden, in the outpatient setting, somebody is getting referred for abnormal MRI and the setting of headache, or [00:22:07] And somebody says, “Well, maybe you could have [00:22:10] disease or neurology not otherwise specified,” and get to the MRI without a clear indication, and we find some abnormalities.

Versus, in the inpatient setting, it’s usually somebody comes into the emergency department and they have something that, you know, usually is more directed by myself, I think that they know they could have something. And often, that’s I'm concerned about stroke or TIA. That’s, again, one of the most common things [00:22:38] to the emergency department for neurology.

And then we find something that isn't related to whatever the reason for the emergency visit. So some of this incidental [00:22:47] scenario, I guess.

**Q:** I'm actually going to ask you about incidental findings, as sort of a general concept. So these silent strokes are often encountered incidentally when physicians are investigating a symptom or medical problem that doesn’t overtly seem to be related to stroke. But then they find them. So what does it mean for you for something to be incidental?

[00:23:16]

**A:** Well, I mean, if it’s asymptomatic, every premise on it is incidental, right. Like just because I think, by definition, I'm saying that I was looking for some problem. Somebody else was looking for something that they think was a problem. And I think that this particular finding on the image is not related to that problem. Otherwise, it’s got a clinical correlate. So I think that these are all necessarily incidental if it’s not what you scanned them for.

**Q:** Sure. In general, for silent strokes, do you feel obligated to respond to them?

[00:23:55]

**A:** I’ll go back to my [00:23:58] ability declaration. It depends on what's the total context? How many other things do I think are playing into this person having a high risk going forward of an ischemic event? And I think that yes, you can use something that you declare yourself uncertain [?] to do that. But I don’t know that necessarily everyone does. And again, that comes to the type of morphology of doing it.

And say that somebody’s got a wedge-shaped two and a half centimeter chunk out of their temporal lobe, and they're a 35-year old healthy woman. Like well yeah, I'm going to work that up. But if it’s just a sub centimeter white matter abnormality, you might say, “Well, we’ll do another scan. I'm not sure that I'm going to start doing a whole lot of vascular receptor evaluations for you if you [00:24:53] been on the radar beforehand. Does that make sense?

**Q:** It does, yeah. So it seems like you tried to individualize the care. A lot of it depends a little bit on their prior history or your clinical suspicion.

[00:25:06]

**A:** Absolutely. Like say that somebody comes in with, you know, right arm and face weakness and difficulty speaking for 30 minutes. And then I find some right hemisphere sub centimeter white matter abnormality, I'm not attributing those things to be related, left sided weakness, right, [00:25:33] It’s supposed to be—I’m trying to paint the picture of the left hemisphere [00:25:37] right hemisphere imaging abnormality. That scan is maybe suggesting that there's other things going on. But, you know, 30 minutes of an event may not leave any lasting marks on the MRI in the slope on the clinical scenario. Hopefully you follow me there, right?

**Q:** Yes, yes. Okay. Are there other patient-specific factors that influence your approach?

[00:26:12]

**A:** I think, just in general, like what is their baseline set of vascular risk, at least as whatever is apparent, in terms of known historical factors. And what specific clinical incident led to them being in my care? Or even I’d also want to [00:26:30] like say even, if they came in for headaches, you're like, “Have you ever had a period of time where there was a short vocal neurologic deficit?” And so I think that there's the—that’s the two elements, is a pseudo[?] clinical event and the general respecter profile. Those are the dimensions I'm looking at.

**Q:** Okay. And just to get a sense for putting aside kind of the decision on how aggressive you want to be in terms of doing treatments or testing, just thinking now about the range of things you might do, including like, for example, for the most—the people who you think are at greatest risk—I might ask you a few questions about what are the types of responses you might have. So, for example, would you start or modify treatments for patients with silent stroke?

[00:27:29]

**A:** Would I start what?

**Q:** Would you start or modify treatments for patients with silent strokes?

[00:27:39]

**A:** Assuming that I had made the attribution that the [00:27:42] finding is a silent stroke and not a non-specific finding, which again is, I think that the line is not clearly drawn. But if I said that this was [00:27:55] I would modify treatments in a fashion not dissimilar to how I would modify for a symptomatic stroke. And we’ve had other conversations about how—what is the evidence basis for treatment modification? And what's the evidence basis for upping an aspirin dose, or switching from aspirin to Plavix? And I don’t think that’s a robust thing.

So in some of the [00:28:16] that they had a, you know, a TIA or a symptomatic stroke. So, in most cases, I think I would—if I'm making the attribution yes, this is an ischemic event, I would probably treat it similarly to a [00:28:35] ischemic event.

**Q:** Okay.

**A:** All right. I kind of talked in circles.

**Q:** That’s fine. In terms of, again, like in the most extreme cases or in terms of the widest range of what you might do, are there particular tests that you might order when you encounter silent stroke?

[00:28:57]

**A:** I don’t think that silent versus symptomatic changes what type of tests I'm ordering. I think I would treat it, if it’s based on the patient, what are the appropriate tests for their clinical circumstances?

**Q:** Sure.

[00:29:10]

**A:** Symptomatic versus not symptomatic, so I don’t think it would dictate that unless I hear of something that I'm not aware of right now.

**Q:** And for you, you are a specialist. But there are some times other people that we get involved as well, would you consider referring these patients to any specialists for additional insight?

[00:29:35]

**A:** Yes and no. I mean yes and no, I'm a hospitalist[?] and I’ll have them follow up with a stroke doctor. But say they were seeing me in a stroke clinic, I don’t think that I would. You know, it comes back to the last question you were asking, which is like, are there different tests that you would order? And I think that that says that, do we know if these patients are any different than the other one? Well, I think that that’s an open question. So if I see evidence to suggest that there's different risk factors to these patients, then yeah, I’ll test for the risk factors appropriately. But I don’t know if that’s been identified as yet.

**Q:** Finally, do you ever ask your patients to make any lifestyle changes or behavioral modifications?

[00:30:19]

**A:** Yes.

**Q:** What sort of things? [laughter]

[00:30:25]

**A:** Usually I talk about smoking, avoiding sedentary lifestyle, diet, all the good stuff.

**Q:** Including for silent stroke, I should have clarified that.

[00:30:35]

**A:** Again, if I made the assessment that I think that this finding is truly ischemic, then yeah, then they're going to have—like I said, the treatment would be similar to symptomatic. And that would include lifestyle modification.

**Q:** Okay, fair enough. A little bit of a different topic, but do you speak with your patients about these imaging findings? And if so, how do you frame that discussion?

[00:31:05]

**A:** Well I always tell patients about any imaging findings, because, you know, if they don’t hear from me that their reports belong to that very [00:31:13] I’d rather have them have an intelligent guide through a report rather than them sort of left in the lurch. And again, it just depends on what's the assessment that I've made. If it’s present but I don’t think that it’s necessarily [00:31:30] more of an [00:31:31] say like, “Hey, we see this on the imagery.” Often I will show the patients the image. And I’ll say, “We’re not sure what this means. We’re not sure that it’s something that we need to be worried about. This is what the plan is going to be like. [00:31:45] I think that we have this finding, like this was a stroke. But we need to address it this way.”

But yeah, I think I would describe what there is, and then what I'm thinking about, because I'm just worried about—You know, they're going to find out, “Hey, there was something in my brain. [00:32:00]”

**Q:** I couldn’t quite hear the last 10 seconds of that. Can you repeat that?

[00:32:08]

**A:** So I was saying that if basically somebody has an abnormal imaging [00:32:14] I, even if there's no significance, I will tell the patient about it, and spread my impression of it, just because I’d rather—I'm always [00:32:22] finding out that there was something called abnormal. And I want them to know what I think, because that’s why they came to see me. But even if it’s not why they came to see me, I think that that’s a reasonable expectation people have of their physicians, is like if I'm seeing a brain doctor, and there's something on my brain, they should tell me what's going on, even if it’s nothing.

**Q:** Sure. Do you think, in terms of the way that you framed that discussion, do you tend to incorporate some degree of reference to the uncertainty about this? Or do you tend to present it with a fair amount of confidence? You know, this is a brain infarct. You just didn’t have symptoms, versus, well, this might be a brain infarct without any symptoms, but I'm not entirely sure?

[00:33:10]

**A:** Again, I think it depends on how have I attributed it? If I feel confident that it’s an infarct, that they didn’t have symptoms at all, I discuss it in frank terms of such. If I feel there's uncertainty, I'm happy to portray it as having an uncertainty. And again, I try to always relate it to, this is how it plays into the steps we’re taking next, whatever those may be.

**Q:** Just if you were going to come up with a rough estimate, do you think you’d tend to be more towards one side versus the other? Or half and half?

[00:33:47]

**A:** I tend to be or that we as a population of neurologists tend to be?

**Q:** You, specifically.

[00:33:57]

**A:** I would say I tend to lean towards not wanting to attribute things to ischemia once I feel very confident about it and a relatively high comfort with uncertainty, and a high comfort with telling my patients when I'm uncertain. I think patients do pretty well with that. And I basically say, “Well, these are the things that I'm thinking about. And this is how much I’m thinking of one or the other. And this is what we need to do.” But yeah, I think that that would happen more times than I’d say. This was definitely, you know, I think a stroke that happened. And this is what we’re doing with it, just because I just think that that attribution that I'm making happens less often.

**Q:** Okay. Just to make sure I heard that correctly, are you suggesting that more often than not you tend to be pretty confident in terms of deciding whether or not something’s a silent infarct, and then you’ll convey that accordingly to the patient?

[00:35:02]

**A:** No, I would actually say that I have a pretty—I think that there’s—of these imaging abnormalities that people might throw into the bucket of silent stroke, I would say that there's less of them that I feel, “Oh, this is definitely a stroke,” and more of them that, like, “Well, could be a stroke, could be other things.” And then I will represent to them I think it could be a stroke. And again, this is not a dichotomy either. I say, “Well, I'm fairly certain this is a stroke, but it could be something else.” Or, “No, it could be a stroke, but I think it’s probably something else.” And then everything in between on that spectrum.

But I think me feeling very confident about what the actual imaging finding is, is probably less often than me sensing some uncertainty.

**Q:** Okay. How have people responded when you’ve given them the diagnosis or told them about these findings?

[00:36:06]

**A:** So are we saying in the subset of people that I have a higher degree of certainty in?

**Q:** Actually, just any of them.

[00:36:19]

**A:** I’d say in general, barring a particularly anxious patient, I'm not sure that this necessarily resonates with patients because they don’t have something salient to connect it to. So I try to demonstrate that it’s important to me, and hopefully it will be important to them accordingly. But I'm not sure that it always comes through. Now, again, me being the talking[?] person, I think that I want them to understand. But I think the most important thing is, how do they behave? Or do they stick with the treatment plan? And that is a little tougher to assess.

I haven't encountered much in terms of, “Well, I don’t want to do these tests or change this treatment because this doesn’t seem—it’s seems fuzzy to me.” I've never had that. But it just, I get a very fuzzy sense that people aren’t taking it quite as seriously.

**Q:** Sure.

**A:** To quantify, though.

**Q:** Sure. Do you think that these silent strokes have caused harm to the patients? And if so, what harm do you think this has caused?

[00:37:37]

**A:** Yeah, potentially. I think defining something as silent or asymptomatic, that becomes an issue, a threshold, right. You know, if I put somebody through an [00:37:48] or cognitive battery before they had the abnormality on their brain and after, would I be able to detect something? I don’t know. Maybe. I would hypothesize that in of these cases, at least, yes. And I think that there is, you know, a lot of what we think of as symptomatic in neurology is very coarse, you know, even like a bedside, careful bedside neurologic testing and examination. I think there's a lot of neurologic dysfunction that you could miss there. I think this is really apparent when you’re examining about accumulation of neurologic issues. [?] MS comes to mind pretty strongly. You know, these patients have these nonspecific things that you don’t localize real well, like fatigue or cognitive slowing.

And I suspect that some of these people would—stuff that we’re saying is “asymptomatic” actually probably does have these symptoms. But then it also probably, a subset of patients that we are not able to detect any dysfunction. But I don’t know. It’s just hard for me to say that, if there's neurons gone and a measurable amount, that they didn’t have any purpose. But you know, hard to say.

**Q:** Do you think, I guess from your perspective, how much do silent strokes put patients at greater risk for other health issues?

[00:39:19]

**A:** I mean probably does, I'm unsure of the magnitude. I would suspect—The biggest thing I would suspect would probably be cognitive issues and dementia if you're doing more accumulated disease. I'm not sure. And also, I think, again coming way back in the conversation, it’s like there's probably heterogeneity in what we’re even talking about. So I imagine the effects are heterogeneous as well.

**Q:** Sure, sure. And this is just a clarification question. But do you think that a true silent infarct puts patients at greater risk for having symptomatic strokes?

[00:40:16]

**A:** If I'm going to say that this is, yeah, a truly a silent stroke, then like if it’s [00:40:23] in nature, then yes. I’d be surprised if it didn’t. And I will operate under the belief that it does until informed otherwise.

**Q:** Sure. How comfortable do you feel treating patients with silent strokes?

[00:40:39]

**A:** Proportionate to how confident I am that they are ischemic and [00:40:43] [laughter] If I feel that they are definitely, like [00:40:48] it’s definitely ischemic, then I feel very comfortable. The more uncertainty there is, the less comfort I have.

**Q:** You mentioned that you don’t think there are any guidelines. But even in the absence of guidelines, do you follow any particular practice guidelines? And if so, which ones?

[00:41:09]

**A:** Well, you know, in terms of working up, again, if I have made that strong attribution with certainty, then yeah, I stick with mostly [00:41:20] in terms of working out the—Look. But you diverge from that as appropriate for the individual patients. Guidelines are good, but that’s what they are, they're guidelines. And you always want to have individualized care.

**Q:** Sure. And those are the secondary prevention guidelines that you're referring to?

**A:** Yeah, secondary prevention. I think that, again, if I think that this is really ischemic, that takes them out of primary prevention, in my view. Something’s happened.

**Q:** Sure. You alluded to some of these earlier, but what do you think are the major knowledge gaps in this area? What are the things that you would want to know more about?

[00:41:59]

**A:** So I would want to know which findings truly represent ischemia, what are the—of people who have these imaging findings, what is their likelihood of having a symptomatic stroke in the future? What are the impact, if any, on the patient’s wellbeing if they're supposedly [00:42:22] cognitive effects? Are there different respective profiles for asymptomatic and symptomatic stroke? And should treatment options differ for symptomatic [00:42:33] asymptomatic strokes? Is that enough questions for you?

**Q:** Yeah, that’s great. So we’re coming pretty close to the end of the interview. The last part is a thought experiment to see how your practice might change, given information from new studies.

**A:** Okay.

**Q:** So I’ll present to you kind of two study designs. The first is, let’s imagine that there is a big rigorous observational comparative effectiveness study that’s performed to determine the effectiveness of prevention therapies in the setting of silent stroke. And let’s say the goal is to prevent symptomatic stroke. So what if this study found the opposite of what your usual practice might be? So these are all patients who are quite certain that they had a silent brain infarct. Normally you’d put them on an aspirin. But it turns out that putting them on an aspirin doesn’t seem to prevent ischemic strokes as much as it’s causing harm in terms of bleeding risk. Would this type of study in this result be enough to convince you to change your practice?

[00:43:38]

**A:** Probably. I think the main thing that I’d want to know about the methods section is how were patients selected for asymptomatic stroke. Because in the end, I think these people get scans for a reason. And those reasons are heterogeneous. So I think that there's a potential of them choosing [00:43:57] because, I don’t know, you’d have a lot of people with cognitive things, and you're having a higher representation of cerebral amyeloid and that’s the increased bleeding. All right, let me make this simpler. Yes, that would be enough to change my practice, barring a methodological complaint.

**Q:** That’s a good point.

**A:** I don’t think I'm making it too complicated.

**Q:** No, no, that’s great, because it sounded like you were getting a little bit worried about potential selection bias related to the indication of the original scan. Is that correct?

[00:44:28]

**A:** Yes. I mean you're going to have a population of people who have asymptomatic strokes and a population of people who have scans. And while I think it’s the scan is that uniform? And is there a potential for bias there? Because that’s always going to be an issue when you're studying this, because the other population, well we know what happened.

**Q:** Sure. So the second study design is a randomized controlled trial. And let’s say it was performed looking at the same treatments. Let’s say it was aspirin versus no aspirin for people with silent brain infarcts, with an aim to prevent symptomatic stroke, and also to look at safety outcomes. If a randomized trial would be done, how would you imagine patients would be recruited into such a study? Because you mentioned those concerns about selection bias.

[00:45:27]

**A:** Yeah, no. So I think you’d have to—That’s a good question, because I think you’d want to end up matching people from their risk profiles in the symptomatic or asymptomatic group. But then, again, I worry about that having validity, because most of these people, just in general [00:45:44] profiles. So I just worry about the person who has a white matter abnormality and a 25 year old who’s coming in with headaches versus other people. Would they even be included? I think you’d have to have—I think you would work hard to have intelligent inclusion criteria for the asymptomatic stroke people. And then maybe match the groups. I'm honestly not sure about that. That’s a good question.

**Q:** Would it make it any easier if you had sort of an age limit, like maybe people only over age 50 for example?

[00:46:27]

**A:** I think that’s a reasonable consideration. But I think one of the things I’d want to know is if we’re saying, what's the baseline frequency of people who have asymptomatic strokes, if there is a predilection for the young, then I think that you're not highly representative sample if you're making [00:46:44]. So I think it is a reasonable thing to think about, but I'm not sure I want to say that just flat out. There’d have to be a lot of thought going into how you design this trial, actually.

**Q:** Sure. Speaking of which, do you have any concerns about feasibility of a randomized trial?

**A:** Feasibility?

**Q:** Yeah.

[00:47:06]

**A:** Absolutely. Yeah. I mean I don’t think of silent stroke as something that I identify particularly often on an anecdotal basis. I definitely worry about recruitments. And also, like a spec[?] size, we’re talking about a treatment effect. Like I would imagine you’d have to have a pretty big end. And we know it’s hard thing to get these people enrolled in these big stroke trials. But absolutely, I have concerns about feasibility for it.

**Q:** Sure. Do you have any concerns about equipoise[?] in doing a randomized trial?

[00:47:56]

**A:** Yes. I think that that could be helped a little bit by if there were or if there are that I'm just not aware of, observational studies indicating risks of symptomatic strokes [00:48:16] asymptomatic stroke. If we thought that risk was high, then I think that’s my concerns about equipoise. But I think if there's more uncertainty about that, then the benefits of getting more knowledge may outweigh the risks of withholding an effective treatment. But I think that other studies, whether or not they exist right now, should inform that decision.

**Q:** I'm sorry, the audio cut out very slightly. But were you saying the thing that you would want to be more certain about is the rates or the risk of symptomatic stroke after silent stroke? Is that what you were asking about?

[00:48:59]

**A:** That’s correct. I think that would be the biggest thing. I know we’re not going to necessarily have perfect data on that. But I’d like to assess the knowledge that we have, and if that’s particularly weak, that they’d probably do some study into that. Retrospective would be fine, but—

**Q:** Okay. I’ll actually give you a little bit of that information, but I want to ask you one more question first. In either type of study, a treatment trial or a comparative effectiveness study, are there particular outcomes that you think would be most important to assess?

[00:49:45]

**A:** So I'm big into recurrent ischemic events plus all-cause mortality. I think then you’d want to also know about hemorrhages, particularly intracerebral hemorrhages for a safety effect as an endpoint, plus or minus whether or not you want other vascular events that aren’t in brain. And I think that introduces some more uncertainty with the [00:50:15].

**Q:** Sure. We’re almost at the end. Let me give you a few known statistics. It’s actually part of the debriefing process, but also there are a couple questions I want to ask you. So there have been several large cohort studies like the Framingham Heart Study, Rotterdam, Northern Manhattan Cardiovascular Health Study, these are all sort of big either single city or multi city or regional cohorts. Usually several thousand patients that actually have involved some degree of screening MRIs. So basically, they set up a time to do a brain MRI, irregardless of symptoms or anything else. There are different inclusion criteria for each of these cohorts. Some are healthy, some are less healthy.

But what they found is, so just in individuals over age 50, because they're mostly older cohorts, but like from middle age up, it turns out that the prevalence of silent brain infarcts is about 20 percent. And this is using some—there's a strive towards consensus criteria for imaging. So these could be acute or chronic lesions for the ones that are in subcortical areas, they have to meet a certain size criteria. Like they have to be at least three millimeters or greater. Usually have either cavitation or an irregular margin. So there's been some thought about this, in terms of coming up with a consensus.

So they found that. So they're fairly common, starting in the 50s. The middle of the ground study was the Framingham Heart Study which indicated, in the 50s, the prevalence is around 7 ½ percent. And then in the 60s, it goes up to 15 percent. In the 70s it goes up to 25 percent, and so on. Just up from there.

They also looked at outcomes. And so they found that silent brain infarcts are associated with a two to four-fold relative increased risk of symptomatic stroke and a two to three-fold increased risk of dementia. But this is between the scans, which were usually about a few years apart, five, six years apart.

So knowing that, going back to that question about equipoise, do you think that there would be equipoise, in terms of doing a treatment trial?

[00:52:47]

**A:** Good question. It’s also framework that I hadn’t been thinking about in before, because clinically I encounter these patients [00:52:59] You're talking about population spec sheet didn’t get a scan for some other reason.

**Q:** Exactly.

[00:53:04]

**A:** Which is very interesting. It hadn’t even occurred to me that people were doing that, although I should have thought of Framingham I guess. But I didn’t know they were scanning people in it. So it’s certainly concern for me. I'm honestly not familiar with how we necessarily did the calculus for what levels of risk have we sort of decided are examples or stories and guidelines. I suspect there are. So it’s something I would have to take into consideration. But I don’t know that I could do the decision right now if it was done.

**Q:** Sure. So it’s kind of an open question for you.

[00:53:52]

**A:** Yeah. I mean it bears considering, because I think, you know, that’s exactly what I was saying, if there is existing evidence that there's risk going forward, it makes you feel more concerned about withholding effective treatment from these patients. But I think that I’d lean still towards thinking that the knowledge benefit could potentially outweigh that risk, just assuming you have your criteria for early termination of a trial if you're showing a benefit at a certain, standard trial design stuff.

**Q:** Fair enough. And also, with these statistics in mind, or at least knowing that there's risk, knowing that this is fairly prevalent, if we were to find some sort of effective prevention strategy, even if it’s just sort of modestly effective, do you think it would actually be worth screening people for silent brain infarcts?

[00:54:59]

**A:** Yeah, potentially. I mean it depends on the magnitude of the effect with our [00:55:06] treats, how much benefit are we doing? What's the cost of doing these? And also, you know, when we can scan people, you know, just find stuff you're looking for. It’s like yeah, other, incidental [00:55:18] So there's a calculus to be done, but I think it would bear consideration.

**Q:** Do you think you would have any ideas in terms of how that would be done?

[00:55:37]

**A:** Sorry, how what would be done?

**Q:** How screening would be done? Like for example, let’s say it ends up being—like you're trying to figure out what’s cost-effective, right. You have to set some parameters. Like would you pick an age and then screen everyone at that age? Or would you try and somehow come up with a risk calculator and figure out who’s at risk?

[00:56:05]

**A:** Yeah, I'm leaning more towards the latter, a certain age could be that. I’d like to think that there should be data we can extrapolate who’s going to receive the most benefit from screening. And I admit that, you know, it could be a scenario that we talk about, like colon cancer, where we just say everyone over 50, you know, you get your colonoscopy. But I’d like it to be data-driven, just who is likely to benefit, and by how much. And I have to think we can do this in an intelligent fashion, probably based on a risk score. But I’d like that to be derived from [00:56:47] that suggests that people do get better [00:56:51] initial.

**Q:** Alternatively, I suppose some could also argue just put everybody on an aspirin and a statin, right?

[00:57:03]

**A:** I’d be inclined to think that the risks would outweigh the benefits of that, because those medicines are largely benign, but a big enough denominator, you're going to start to get adverse effects. So I think, you know, aspirin in the water probably isn't going to be the answer.

**Q:** Fair enough. Do you have any other questions or thoughts about this?

[00:57:28]

**A:** No, I just wish that I had known a little bit more about those other studies, because you educated me now. But I'm glad to know now. I think the other thing is, it’s always going to be interesting like how we define this, because I think that it probably came through in this interview that I feel a little shaky about what I'm deciding to call ischemic and not ischemic. And I wonder if I would find, you know, study definitions to be accurately convincing only or including other non-ischemic ideologies in there.

And then the fourth question would be, does it matter? If we’re catching enough of it, and it makes a difference in terms of outcomes, should we just say that it’s good enough and move forward, even if we don’t have certainty of the pathophysiology? So I guess that’s a comment, not a question.

**Q:** No, that’s good. Thanks.

END OF INTERVIEW
