# Supplementary material for: Clinicians’ perspectives on incidentally discovered silent brain infarcts – A qualitative study
Source: PLoS One. 2018 Mar 29;13(3):e0194971. doi: 10.1371/journal.pone.0194971 (PMC5875806; doi:10.1371/journal.pone.0194971)
Supplement: S2 File — The final version of the interview guide. (DOCX) [file pone.0194971.s002.docx]

**S2 Interview Guide – Final iteration.**

**Exploratory Questions (required)**

E1. Tell me what you know about silent or covert strokes so far. (Knowledge)

F1

E2. Why do you think covert strokes occur? (Nature, Pathophysiology, Risk Factor)

E3. From your perspective, do covert strokes have similar or different pathophysiologies from symptomatic strokes? In what ways are they similar or different? (Nature, Pathophysiology, Risk Factors)

E4. Do you think there are different types of covert strokes? What would be your categories?

F2

E5. What do you think are major risk factors for covert strokes? (Risk Factors)

E6. In what settings or scenarios have you encountered this diagnosis? (Experience)

F3

E7. These covert strokes are often encountered *incidentally* when physicians are investigating a symptom or medical problem that does not overtly seem to be related to stroke. What does it mean to you for something to be “incidental?” (Nature)

a) Do you feel obligated to respond? (Experience, Perception)

b) If so, how do you typically respond to these findings? (Practice)

F4

F5

F6

F7

c) With regards to individualizing care, what patient-specific factors

influence your approach?

E8. How do you approach the radiologic imaging?

1. Do you rely on the radiologist’s report, do you review the images directly, or a combination of the two?
2. How does the radiologist’s language influence your decision-making?

E9. Do you speak with your patients about these imaging findings? If so, how do you frame that discussion? (Patient education, communication)

E10. Do you think these covert strokes have caused harm to patients? (Risk)

a) If so, what harm do you think they have caused?

E11. From your perspective, how much do covert strokes put patients at greater risk for other health issues? (Risk)

a) If so, for what other health issues do they increase risk?

F8

E12. How comfortable do you feel treating patients with covert strokes? (Perception)

a) Do you follow any practice guidelines? If so, which ones?

b) What do you think are the major knowledge gaps in this area?

c) What do you want to know more about?

E13. This last part is a thought experiment to see how your practice might change given information from new studies.

1. Let’s imagine that a rigorous, observational, comparative effectiveness study was performed to determine the effectiveness of prevention therapies for covert stroke, and this study found that the opposite of your usual practice was the most effective or least harmful treatment decision. Would this convince you to change your practice? (Evidence synthesis, Study planning)
2. If not, what would convince you to change your practice?
3. If a randomized controlled trial were performed looking at the same treatments, how do you think patients should be recruited into such a study?
4. With regards to an RCT, do you have any concerns about feasibility? Any concerns about equipoise?
5. What do you think are the important outcomes to assess in either type of study?
6. Let me give you a few known statistics. In several large cohort studies, in individuals over age 50, the prevalence of covert brain infarcts is about 20%. Covert infarcts are associated with a two-to-four fold increase in the risk of symptomatic stroke as well as dementia. Knowing that, if we were to find an modestly effective prevention strategy, do you think it would be worth screening for covert infarcts? If so, how? If not, what do you think would be the best way to address this issue?

E14. Do you have any questions or additional thoughts on this issue?

**Focused Questions (as many as possible, time permitting)**

**After question E1**

F1. The diagnosis has several names: silent stroke, silent brain infarction, silent cerebral infarction, covert stroke, covert brain infarction, subclinical stroke, subtle stroke, asymptomatic stroke. (Language, Terminology)

a) Which of these terms have you heard used?

b) Which do you use most often?

c) Are there other terms that you have heard used?

d) Which do you think is the most appropriate or useful term?

**After question E4**

F2. How are covert brain infarcts similar or different from white matter disease?

1. Are they similar or different in clinical significance?
2. Would you react to one but not the other? Or would you react the same to both?

**After question E6**

F3. With what symptoms or issues did your patients present?

a) Did you think that your patients’ symptoms or issues were related to the silent stroke?

**After question E7**

F4. Do you start or modify treatments for patients with covert strokes? (Experience, Practice)

a) If so, what do you start or change?

F5. Do you order any additional tests when you encounter covert strokes? (Experience, Practice)

a) If so, what do you order?

F6. Do you refer patients with covert strokes to specialists? (Experience, Practice)

a) If so, to whom do you refer your patients?

F7. Do you ask your patients to make any lifestyle changes or behavioral modifications? (Experience, Practice)

a) If so, what do you ask them to do?

**After question E11**

F8. Do you think covert strokes place your patients at risk for any of the following? (Risk)

☐ Stroke

☐ Intracerebral hemorrhage

☐ Heart attack (myocardial infarction)

☐ Heart failure

☐ High blood pressure

☐ High cholesterol

☐ Diabetes

☐ Headache

☐ Vertigo

☐ Seizures

☐ Falls

☐ Memory loss

☐ Dementia

☐ Anxiety

☐ Depression

☐ Schizophrenia
